# Pan-cancer analysis of somatic mutations and transcriptomes reveals common functional gene clusters shared by multiple cancer types

**DOI:** 10.1038/s41598-018-24379-y

**Published:** 2018-04-16

**Authors:** Hyeongmin Kim, Yong-Min Kim

**Affiliations:** Korean Bioinformation Center, Korea Research Institute of Bioscience and Biotechnology, Daejeon, 34141 Korea

## Abstract

To discover functional gene clusters across cancers, we performed a systematic pan-cancer analysis of 33 cancer types. We identified genes that were associated with somatic mutations and were the cores of a co-expression network. We found that multiple cancer types have relatively exclusive hub genes individually; however, the hub genes cooperate with each other based on their functional relationship. When we built a protein-protein interaction network of hub genes and found nine functional gene clusters across cancer types, the gene clusters divided not only the region of the network map, but also the function of the network by their distinct roles related to the development and progression of cancer. This functional relationship between the clusters and cancers was underpinned by the high expression of module genes and enrichment of programmed cell death, and known candidate cancer genes. In addition to protein-coding hub genes, non-coding hub genes had a possible relationship with cancer. Overall, our approach of investigating cancer genes enabled finding pan-cancer hub genes and common functional gene clusters shared by multiple cancer types based on the expression status of the primary tumour and the functional relationship of genes in the biological network.

## Introduction

The Cancer Genome Atlas (TCGA, https://cancergenome.nih.gov/), which contains genome maps from more than 30 cancer types, has revealed thousands to hundreds of thousands of somatic mutations. Through decades of cancer research, we now know that cancer genes contain “driver” mutations that have causal roles in the development of cancer, and accumulated “passenger” mutations that are from cell differentiation and proliferation^1^. Driver mutations modify protein-coding sequences of genes and provide a selective growth advantage. Small differences in the rates of cell division and cell death that result from each driver mutation contribute to an enormous amount of cancer cells over years^2^. Thus, cells that have driver mutations tend to accumulate more mutations and proliferate uncontrollably. Passenger mutations that were not thought to be active contributors to cancer are now considered “dark matter” together with mutations in non-coding sequences^3^. These mutations consistently affect exonic motifs by altering the mRNA splicing pattern^4^, and changing the coding regions in DNA and RNA, which can affect gene regulation^3^.

To determine the effect of genomic variants on transcriptomic changes, integrated analysis using multi-omics data, including somatic mutations and transcriptomes, has been performed with liver and breast cancers^5,6^. The data showed cancer cells were transcriptionally more active than normal cells. In addition, many genomic variants of cancers show stable high expression that triggers transcriptional alterations such as over- or under-expression of genes and splicing aberrations. Other studies that integrated somatic mutation and transcriptome data were performed in breast cancer^7,8^. One report identified a potential driver gene mutation that was predictive of patient survival, and the other report used the data to stratify patients into groups with different clinical outcomes. In all studies referenced above, transcriptome data were used based on their important biological aspect, rather than as ancillary data.

Currently, the use of multi-omics data is not limited to a single type of cancer; rather, comprehensive analysis of multiple cancer types is becoming the new paradigm to understand cancer^9–11^. Hoadley *et al*. integrated six different omics datasets (mRNA-Seq, miRNA-Seq, reverse-phase protein arrays, structural copy number alterations, DNA methylation, and somatic mutations) from 12 cancer types, and found 11 subtypes based on expression profile that were informative and extended beyond tissue-of-origin cancer types in the classification^10^. Similarly, Liu *et al*. integrated three datasets (copy number alterations, somatic mutations, and DNA hyper-methylations) from 12 cancer types^11^; the authors found nine subgroups and reported cross-cancer similarities. Akbani *et al*. showed that the functional proteome gave independent knowledge of cancer that was not captured by genomic and transcriptomic data^9^. In these reports, different properties of omics data enabled complementary detection of different pathways and features across cancers.

Recently, pan-cancer transcriptome analyses that were based on differentially expressed genes (DEGs) between tumour and normal expression have been reported^12,13^. Cao *et al*. investigated co-expression networks using Pearson correlation of DEGs in 16 cancer types and showed that merged pan-cancer gene networks had pan-cancer subnetwork signatures of prognostic potential^12^. Cabanski *et al*. found differentially expressed long non-coding RNAs (lncRNAs) across eight solid tumour types, referred as onco-lncRNAs, which might have oncogenic and tumour suppressor roles^13^.

Thus, these successful pan-cancer studies have led to a broader understanding the nature of cancer. In this study, as part of these efforts, we aimed to identify functional gene clusters across cancer types. Using publicly available cancer data from the TCGA database that included somatic mutations and transcriptomes, we investigated the properties of somatic mutation-associated hub genes in the weighted gene co-expression networks of 33 TCGA cancer types. We focused on the expression status of primary tumours and functional relationships between hub genes in the biological network. Here, we report integrated hub gene sets from multiple cancer types, and nine common functional gene clusters shared by multiple cancer types. The clusters were functionally related to development and progression of cancer, and had high gene expression and enrichment of programmed cell death (PCD) genes and known cancer genes. Non-coding genes of the integrated hub gene set also showed a functional relationship with cancer.

## Results

### Research process

**Figure 1:**
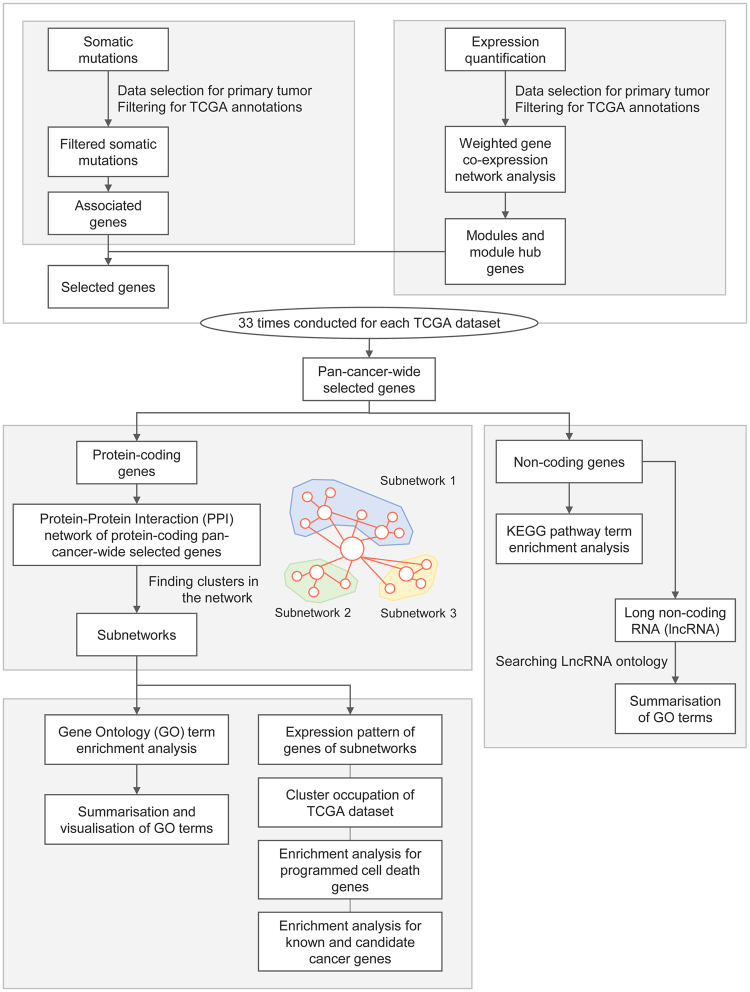
Schematic diagram of the research process.

We used somatic mutations from 10,425 cases and expression quantification data from 9,831 cases (Supplementary Tables S2 and S3, respectively), which encompassed 33 types of cancer (Supplementary Table S1). Following selection of the primary tumour and filtering based on TCGA annotations, weighted gene co-expression analysis (WGCNA^14^) for gene expression data was performed, and WGCNA modules and module hub genes were identified. Then, we selected genes at the intersection between module hub genes and somatic mutation-associated genes for each TCGA dataset for additional analysis. We integrated selected genes from each TCGA dataset into the pan-cancer-wide selected genes (PSGs) group. PSGs were categorised according to protein-coding status. For protein-coding PSGs (pcPSGs), a single-depth network of protein-protein interaction (PPI) was generated and subnetworks were discovered using the method developed by Bader^15^. Subsequently, we summarised the gene clusters of subnetworks and non-coding PSGs (ncPSGs) using gene ontology (GO) terms or Kyoto Encyclopaedia of Genes and Genomes (KEGG)^16^ pathway terms. To understand the characteristics of the gene clusters in subnetworks, we performed additional investigations of the expression patterns of genes and level of cluster occupation of the TCGA dataset, and enrichment analyses for known cancer genes and PCD genes. A schematic diagram of the research process is shown in Fig. 1.

For somatic mutations and expression, only data from the primary tumour type were used and filtered based on annotations. After weighted gene co-expression analyses were conducted, genes at the intersection of somatic mutations-associated genes and module hub genes were collected as selected genes for each TCGA dataset. Then, we integrated selected genes from each TCGA dataset into the pan-cancer-wide selected genes (PSGs) group. For protein-coding PSGs (pcPSGs), a single-depth network of protein-protein interaction (PPI) was generated and subnetworks were discovered. The subnetwork genes and non-coding genes were summarised using gene ontology (GO) terms or KEGG pathway terms. To investigate the characteristics of gene clusters of subnetworks, we investigated the expression pattern of genes and level of cluster occupation of the TCGA dataset, and conducted enrichment analyses for known cancer genes and programmed cell death (PCD) genes.

### Somatic mutation filtering and gene expression data

For somatic mutation data, from 0.4% (TCGA-PAAD) to 84.3% (TCGA-SKCM) of variants were filtered out (Supplementary Table S4). The number of variants per aliquot ranged from 7.4 (TCGA-PCPG) to 979.9 (TCGA-UCEC), and the number of variant-associated genes ranged from 780 (TCGA-UVM) to 26,042 (TCGA-UCEC) (Supplementary Table S5). The number of pan-cancer-wide genes with variants was 27,041. Normalised frequencies of somatic mutation calls from genes are shown in a heatmap (Supplementary Fig. S1). Except for TCGA-LUAD and -LUSC, we could not identify clustering of datasets of the same tissue based on mutation frequencies of genes. Furthermore, we did not observe a distinct cluster of genes that were dominant across all cancer types with the high number of variants. Only one gene, *TTN* was mutated in all 33 TCGA datasets, which had the highest average number of somatic mutations (0.56 mutations per aliquot). Including *TTN*, there were nine genes which had more than one mutation for every ten aliquots: *MUC16*, *LRP1B*, *CSMD3*, *RYR2*, *SYNE1*, *FAT4*, *USH2A*, and *PCLO* genes (Supplementary Data S1). When we took into account of exon length of genes, immunoglobulin genes and mitochondrially encoded genes such as *IGHV2-70*, *IGHD2-15*, *IGHD3-3*, *IGLC2*, *MT-CYB*, *IGHV1-69-2*, *IGHG2*, and *MT-CO3* were potentially having higher mutation burden in a pan-cancer context (Supplementary Data S1).

For quantification of gene expression data, up to 125 (TCGA-OV) cases were filtered out (Supplementary Table S6).

### Weighted gene co-expression network analysis

Before we conducted WGCNA for normalised expression values, we examined the data for excessive missing values and excluded outlying samples for each TCGA dataset (Supplementary Fig. S2). Then, we chose suitable parameters for calculation of connection strength that were required to generate the weighted network for WGCNA (Supplementary Fig. S3 and Supplementary Table S7). As a result, we identified an average of 122 WGCNA modules (from 37 of TCGA-CHOL to 243 of TCGA-LIHC); 81% of genes (from 49.8 of TCGA-BLCA to 97.7 of TCGA-UVM) were covered by the modules (Supplementary Fig. S4, Supplementary Data S2, and Supplementary Table S8). With the intention of assigning a similar number of genes to each dataset, we used an average of 2,852 genes (from 885 of TCGA-THCA to 4,938 of TCGA-DLBC) as module hub genes.

### Pan-cancer-wide selected genes (PSGs)

**Figure 2:**
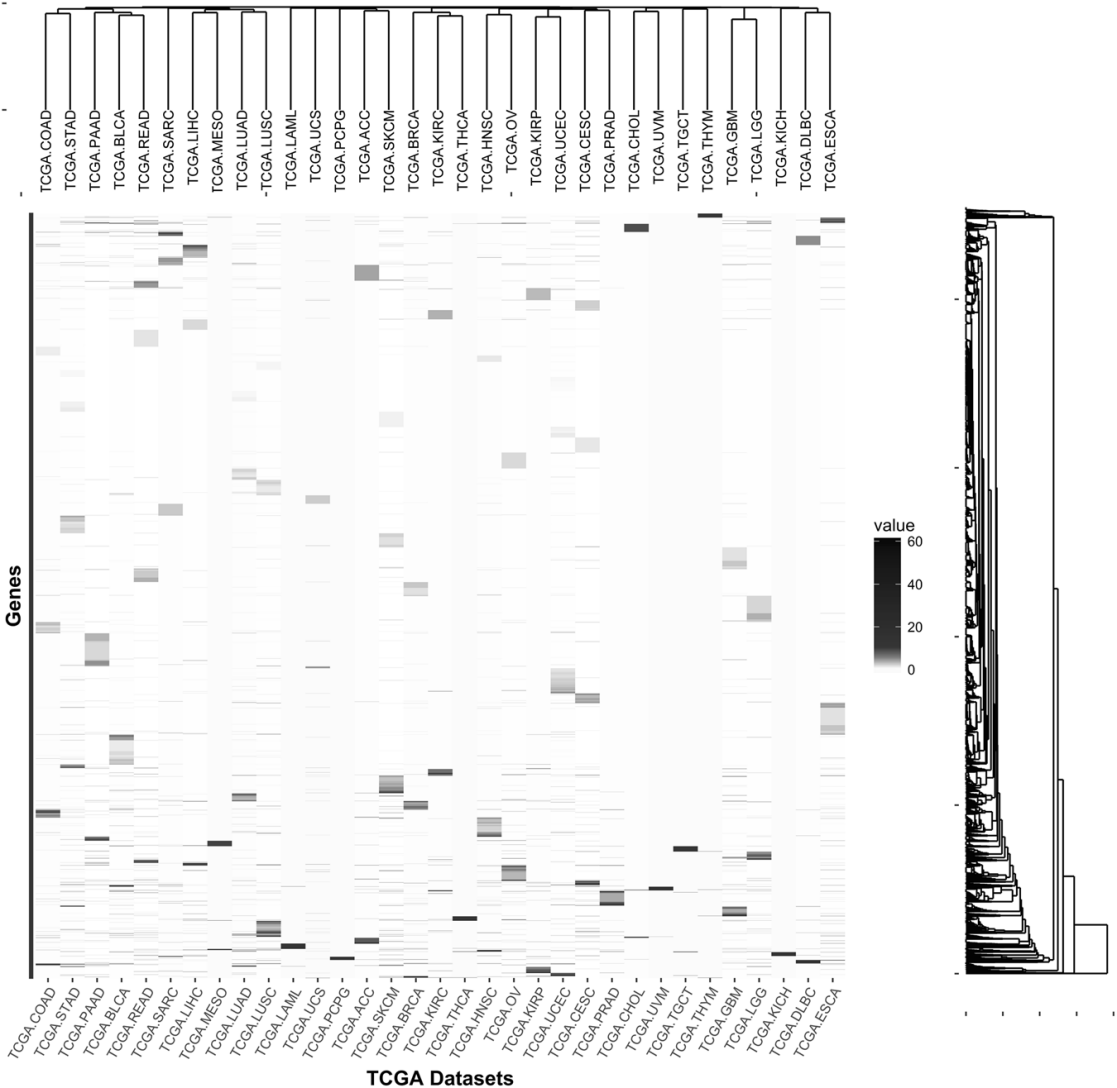
Number of variants of pan-cancer-wide selected genes (PSGs) per aliquot.

To identify genes that had somatic mutations in cancer and that were highly connected with other genes in the co-expression network, we selected genes at the intersection between genes associated with somatic mutations and genes that were module hubs. An average of 218 genes (from 19 of TCGA-PCPG to 536 of TCGA-UCEC) was selected (Supplementary Table S9). After integration, the number of PSGs was 4,546. When we observed genes that overlapped between TCGA datasets, 67.4% of PSGs (3,064 PSGs) belonged to only a single TCGA dataset (Supplementary Fig. S5). Regarding the frequencies of somatic mutations in PSGs, we did not identify dominant genes across all types of cancer, and did not find distinctive clusters of TCGA datasets (Fig. 2).

Heatmap of normalised values of somatic mutation calls of PSGs. TCGA datasets are shown on the x-axis and genes are on the y-axis. A dendrogram at the top of the heatmap show clustering of the datasets. A dendrogram on the right of the heatmap show clustering of the genes.

Among 4,546 PSGs, 3,299 genes were protein-coding genes and 1,247 genes were non-coding genes (Supplementary Fig. S6). Except for TCGA-CHOL, all other TCGA datasets had more pcPSGs than ncPSGs.

### Protein-Protein Interaction (PPI) network of protein-coding PSGs (pcPSGs)

**Figure 3:**
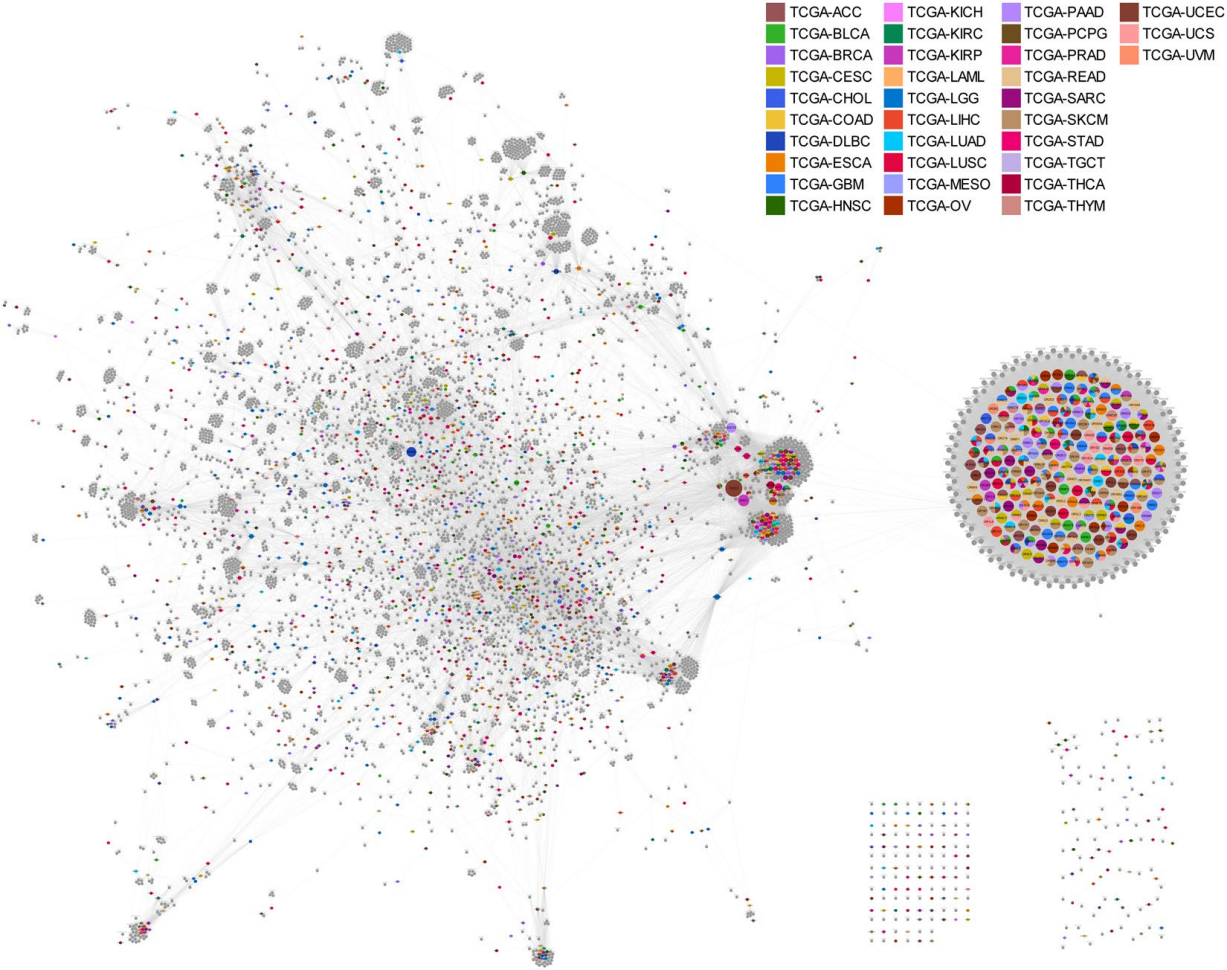
Single-depth protein-protein interaction (PPI) network of protein-coding pan-cancer-wide selected genes (pcPSGs).

Of 3,299 pcPSGs, 1,740 genes had PPI information in the STRING V10 database^17^, and another 5,072 genes had a PPI with pcPSGs (Supplementary Data S3). In this study, we referred to these genes as ‘representors’ and ‘interactors’, respectively. The single-depth PPI network of pcPSGs had 6,812 nodes and 84,128 edges (Fig. 3). All except for 230 genes connected into a large single network consisting of 6,582 genes. The nodes of the network followed a power-law distribution; several genes with a high degree of interaction (Supplementary Fig. S7 and Supplementary Data S3) were noted. They were 207 olfactory receptor genes, *ADCY8*, *EP300*, *GNG2*, *KNG1*, and *RP11-294C11.1* genes. In the network, we did not find notable spatial patterns of PSGs in specific TCGA datasets. PSGs in TCGA datasets were randomly distributed in the PPI network, and PSGs in different TCGA datasets were located closely to each other.

PSGs that have PPI information (representors) are coloured differently according to the TCGA dataset to which they belong. The colour indicator for the TCGA datasets is located to the top right corner. PSGs that belong to more than one TCGA dataset are presented like pie charts. The genes that interact with PSGs (interactors) are presented as grey colour. The node size and name reflect the number of connections. Except for genes of the two groups on the bottom right, all are connected to a large single network.

### Gene Ontology (GO) term enrichment analysis of subnetwork genes

**Figure 4:**
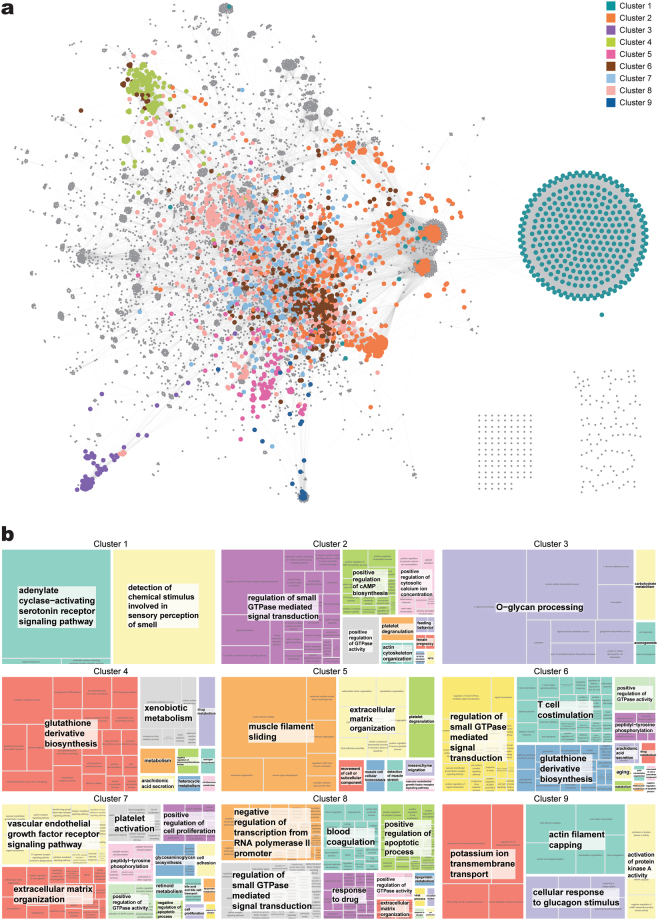
Gene clustering of single-depth PPI network of pcPSGs and gene ontology (GO) term summarisation of gene clusters. (**a**) Gene clusters of nine subnetworks are shown in different colours. The colour indicator is located in the top right corner. The genes that are not in subnetworks are grey colour. (**b**) GO term summary of gene clusters of subnetworks are presented as a tree map. The name of the subnetwork is on top of the tree map, and the relative size of blocks shows the significance of enrichment of the GO term. Similar GO terms were combined and grouped into a large block of the same colour. The term of most uniqueness in the large block is shown on the white box and located at the centre of the block.

To deliver functional annotation of the network, we found nine clusters of genes and created subnetworks based on inter-connection of nodes (Fig. 4a, Supplementary Fig. S8, and Supplementary Table S10). The clusters partitioned the regions of the network map properly. We used an additional program option for large subnetworks, and we obtained relatively large subnetworks compared to many small subnetworks. However, use of the option allowed detection of loosely interacting modules in the network; the clusters’ shared genes were not mutually exclusive (Supplementary Fig. S9). The number of genes shared by clusters 2, 6, 7, and 8 was high.

To summarise the functions of clusters, GO term enrichment analysis was performed for each subnetwork. Among 2,217 subnetwork genes, 2,209 were identified in DAVID Bioinformatics Resources 6.8^18^ (Supplementary Table S10). Then, we summarised the enriched biological GO terms as a tree map (Fig. 4b and Supplementary Fig. S10). For genes of cluster 1, GO terms related to ‘G-protein-coupled receptor signalling pathway’ and ‘sensory perception of smell’ were enriched. In cluster 2, genes of many signal transduction pathways including ‘cell surface receptor signalling pathway’ and ‘intracellular signal transduction’, and genes of ‘regulation of cell proliferation’, ‘regulation of apoptotic process’, and ‘inflammatory response’ were enriched. Cluster 3 had enriched GO terms: ‘O-glycan processing’ and ‘carbohydrate metabolic process’. Cluster 4 had enriched GO terms: ‘glutathione (GSH) derivative biosynthesis’, ‘xenobiotic metabolism’, and ‘drug metabolism’. In cluster 5, ‘muscle filament sliding’, ‘extracellular matrix organisation’, and ‘movement of cell or subcellular component’ were enriched. In cluster 6, GO terms related to signal transduction, immune response, and metabolism of GSH and steroids were enriched. In cluster 7, GO terms related to angiogenesis (‘vascular endothelial growth factor receptor signalling pathway’, ‘extracellular matrix organisation’, and’platelet activation’) and cell proliferation were enriched. In cluster 8, GO terms related to regulation of transcription (‘positive regulation of gene expression’ and ‘transcription from RNA polymerase II promoter’), cell growth (‘epidermal growth factor receptor signalling pathway’ and ‘regulation of cell cycle’), and apoptosis (‘positive regulation of apoptotic process’ and ‘regulation of cell proliferation’) were enriched. In cluster 9, GO terms of transport (‘ER to Golgi vesicle-mediated transport’) and cell movement (‘actin filament capping’ and ‘cellular response to glucagon stimulus’) were enriched.

### Characteristics of subnetworks

**Figure 5:**
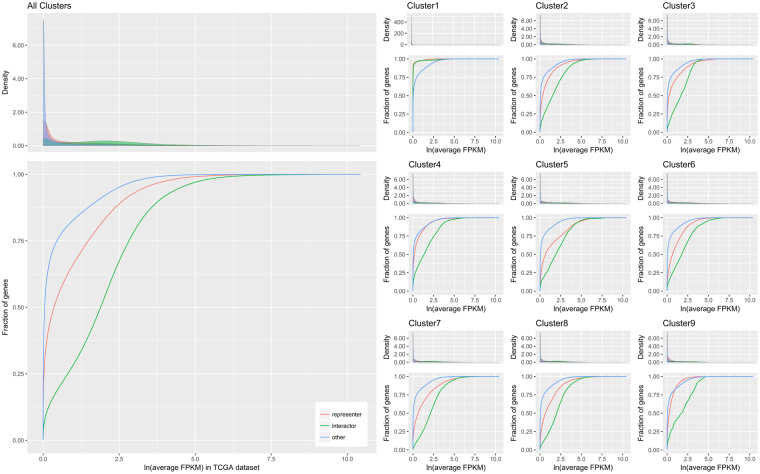
Expression patterns of subnetwork genes.

To identify the characteristics of subnetworks, we performed several analyses. First, we investigated the expression pattern of subnetwork genes (Fig. 5). Expression of genes that belonged to subnetworks was higher than genes not involved in subnetworks. Except for cluster 1, genes in clusters had higher expression levels than genes that were not in clusters, and interactors had higher expression than representors.

Ten panels are shown to present gene expression patterns. The large panel on the left includes genes in all clusters. Nine small panels on the right show genes in a specific cluster. The name of the cluster is on top of each panel. The representors, interactors, and other genes are presented as red, green, and blue, respectively. The panel shows the density and cumulative fraction of genes (y-axis) against log values of average FPKM (“fragments per kilobase of exon per million fragments”; a unit of expression quantification) in the TCGA dataset (x-axis). The expression values of the genes in the left panel were also used in the nine panels on the right.

**Figure 6:**
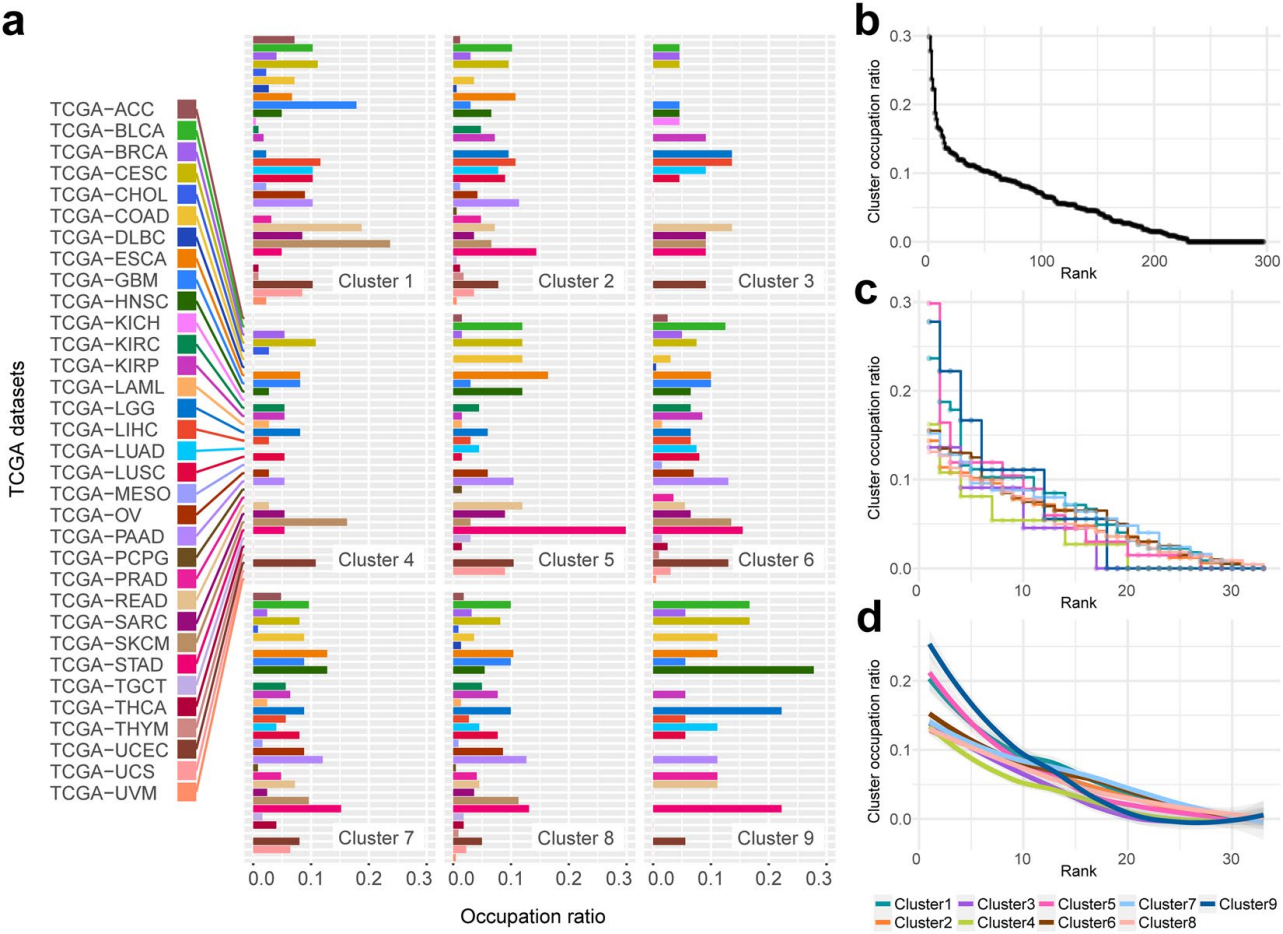
Level of cluster occupation of TCGA datasets in subnetworks and distribution of cluster occupation ratios. (**a**) Occupation ratio of TCGA dataset is shown for each cluster. The length of bar indicates occupation ratio of the dataset that takes the number of shared genes into account. (**b**) For all occupation ratio of (**a**), the ratio against their rank is shown. (**c**) A stair-step plot shows the occupation ratio against the rank for each cluster. (**d**) The smoothing plot using loess regression of (**c**). The indicator of colours for clusters is on the bottom right corner.

Second, we investigated the level of cluster occupation of TCGA datasets in subnetworks (Fig. 6 and Supplementary Fig. S11). We did not identify a dominant cancer type that occupied a cluster exclusively (Fig. 6a and Fig. 6b). The highest occupation ratio was 0.3 of TCGA-STAD in cluster 5. In clusters 3, 4, and 9, there were only 16, 19, and 17 cancer types, respectively. This finding indicated that these clusters had a relatively low level of cluster sharing. However, there were 30, 30, 29, and 32 cancer types in clusters 2, 6, 7, and 8, respectively. In addition, the range of fluctuation in occupation ratios was narrow in clusters 2, 6, 7, and 8 (Fig. 6c and d). This result indicated that these clusters had a relatively high level of cluster sharing.

When we generated a heatmap of cluster-cancer type relationships based on the number of PSGs in clusters, we identified discordance between clustering of cancer types and clustering based on primary site of cancer (Supplementary Fig. S12). The heatmap did not reflect tissue-of-origin-based classification when we stratified cancer types according to the number of PSGs of TCGA datasets in clusters. Clusters 2, 6, 7, and 8, which had a relatively high level of cluster sharing, showed a moderate correlation in hierarchical clustering.

Third, enrichment analysis for PCD genes was performed. We collected three types of PCD: apoptosis (type 1 PCD), autophagy (type 2 PCD), and programmed necrosis (type 3 PCD) (Supplementary Data S4). Then, enrichment of PCD genes in subnetworks, the whole network, and PSGs (Table 1) was investigated. PCD genes were significantly enriched in PSGs compared to genes that were not in PSGs, and were enriched in the PPI network of PSGs compared to genes were not in the network. Within the network, PCD genes were enriched in subnetworks. In subnetworks, PCD genes existed in all clusters except for clusters 1 and 4. Clusters 6, 7, 8, and 9 had enrichment of PCD genes compared to the other clusters and the other genes of the network. The genes of type 1 and 2 PCD were enriched in clusters 6, 7, and 8.

**Table 1:** The number of programmed cell death (PCD) genes and enrichment analysis.

	No. of genes	No. of PCD genes	No. of type 1 PCD genes	No. of type 2 PCD genes	No. of type 3 PCD genes
Cluster 1	342	0	0	0	0
Cluster 2	536	13	5	7	6
Cluster 3	69	1	0	0	1
Cluster 4	158	0	0	0	0
Cluster 5	151	6	6^***^	0	1
Cluster 6	521	28^**,***^	19^**,***^	16^**,***^	8
Cluster 7	399	29^**,***^	18^**,***^	18^**,***^	6
Cluster 8	857	49^**,***^	36^**,***^	28^**,***^	10
Cluster 9	38	4^**,***^	1	3^**,***^	0
Clusters	2,219	79^*^	50^*^	38^*^	21
Not in clusters	4,597	124	64	43	37
Network	6,816	203^*^	114^*^	81^*^	58^*^
Not in network	50,472	113	28	50	41
Pan-cancer-wide selected genes	4,546	43^*^	19^*^	15	20^*^
Non-pan-cancer-wide selected genes	52,742	273	123	116	79

^*^PCD genes were enriched in genes in clusters, networks, and PSGs compared to other genes not in clusters, networks, or PSGs.

**PCD genes were enriched compared to other genes in clusters.

***PCD genes were enriched compared to other genes in networks.

Fourth, we performed enrichment analysis for known and candidate cancer genes. Both known and candidate cancer genes were significantly enriched in PSGs, the PPI network, and clusters (Table 2). Clusters 6, 7, and 8 had enrichment of both known and candidate cancer genes compared to the other clusters and other genes of the network. Cancer-related genes existed in all clusters. Subsequently, we conducted additional enrichment analysis to evaluate PPI network membership and PSGs membership (Supplementary Table S11). Cancer-related genes were enriched in PSGs of the network compared to PSGs that were not in the network. For genes that were not PSGs, the interactors in the network had enrichment of cancer-related genes. For genes of the PPI network, candidate cancer genes were enriched in the representors, and known cancer genes were enriched in the interactors.

**Table 2:** The number of known and candidate cancer genes and enrichment analysis.

	No. of genes	No. of known cancer genes	No. of candidate cancer genes
Cluster 1	342	0	30
Cluster 2	536	21	40
Cluster 3	69	1	5
Cluster 4	158	2	6
Cluster 5	151	7	16
Cluster 6	521	41^**^	59^**^
Cluster 7	399	33^**^	47^**^
Cluster 8	857	89^**^	92^**^
Cluster 9	38	1	6
Clusters	2,219	131^*^	194^*^
Not in clusters	4,597	201	296
Network	6,816	332^*^	490^*^
Not in network	50,472	176	544
Pan-cancer-wide selected genes	4,546	67^*^	278^*^
Not in pan-cancer-wide selected genes	52,742	441	756

^*^Known and candidate cancer genes were enriched in clusters, networks, and PSGs compared to other genes.

^**^Known and candidate cancer genes were enriched in these clusters compared to other clusters and other genes in networks.

### Summarisation of non-coding PSGs (ncPSGs)

We categorised ncPSGs into four categories and 19 subcategories according to annotations in Ensembl (Supplementary Table S12). Pseudogenes composed 77.2% of ncPSGs, and two categories of short non-coding RNA and lncRNA occupied 16.1% and 6.5% of ncPSGs, respectively (Supplementary Figure S13). For each cancer type, occupation of gene type categories was investigated (Supplementary Figure S14). Overall, pseudogenes were a major component. Short non-coding RNA had high levels of cluster occupation in TCGA-LUAD, LUSC, HNSC, BLCA, and UCEC.

To summarise the function of ncPSGs, we performed KEGG pathway enrichment analysis. Among nsPSGs, 245 genes were identified in DAVID Bioinformatics Resources 6.8, and the KEGG pathway term ‘MicroRNAs in cancer’ was significantly enriched for 17 genes (Bonferroni adjusted *p*-value of 2.28∙10^−21^) (Supplementary Table S13). For lncRNAs of ncPSGs, functions were investigated using LncRNA Ontology^19^. Among 81 long ncPSGs, 59 genes (72.8%) were identified in LncRNA Ontology. GO terms related to RNA processing, transport system, signal transduction, and cell growth and death were enriched (Supplementary Figure S15). These findings indicated that ncPSGs also had a possible relationship with cancer.

## Discussion

To investigate the properties of somatic mutation-associated hub genes in the weighted gene co-expression networks of multiple cancer types, we used publicly available somatic variant calls and gene expression quantification values from the TCGA database. This approach ensured reproducibility of the study and reliability of data that were curated manually and extensively over the years by experts.

After applying filtering criteria, 84.3% of variants in the TCGA-SKCM dataset were filtered out (Supplementary Table S4). This might come from the characteristics of SKCM tumours in that primary skin cutaneous melanoma tumours are difficult to pinpoint and distinguish from melanocytic nevi^20,21^. Mutation frequencies varied across cancer types (Supplementary Table S5). The frequencies of variants were high in TCGA-UCEC, SKCM, COAD, LUAD, and LUSC, which were 979.9, 493.4, 327.1, 292.1, and 289.9, respectively. The variation in mutation frequency can be explained by cancer type. In melanoma and lung cancers, exposure to environmental mutagens is known as a major cause of increased mutations^22^. In uterine and colorectal cancers, DNA mismatch repair (MMR) and DNA replication are coupled together, the inactivation of MMR and the inactivation of the proofreading domain of DNA polymerase epsilon (PolE) results in high mutation frequencies^23^.

In identifying somatic mutation-associated genes, we used all types of mutations including synonymous mutations because “passenger” mutations can be contributors to cancer^3,4,24^. However, accumulated non-contributing mutations and non-contributing genes were identified. Therefore, by including genes at the intersection between genes associated with somatic mutations and WGCNA module hub genes, we intended to avoid considerable number of false hub genes that had only somatic mutations or importance in only WGCNA.

Using mutation frequencies of PSGs and the number of PSGs from clusters, we investigated TCGA datasets that could be stratified according to those data (Fig. 2 and Supplementary Fig. S12). However, we did not identify distinguishable clusters of datasets based on the number of variants, and we only identified clustering of datasets when we investigated the number of PSGs in clusters, which were similarly sorted in order of total number of PSGs in datasets. Unlike a network-based stratification (NBS) method^25^, high dimensional data of individual samples were lost in our procedures, which did not reflect tissue-of-origin-based classification and were not suitable to classify cancer types. Aspects of mutation frequencies of PSGs and the number of PSGs of clusters showed little commonality among datasets.

When we observed genes that overlapped between TCGA datasets, 67.4% of PSGs belonged to only a single TCGA dataset (Supplementary Fig. S5). For PSGs that belonged to most datasets, only two genes were found in 11 datasets. This finding indicated that PSGs were not common core genes shared by all cancer types, and each cancer type had relatively exclusive PSGs. In the PPI network of pcPSGs, we did not identify specific TCGA datasets that dominantly occupied clusters of specific sections in the network, and PSGs of different cancer types were mixed-up and closely located in the network (Fig. 3). This pattern also was observed in the subnetworks (Supplementary Fig. S8). This finding indicated that PSGs of different cancer types were functionally related to each other. Taken together, the data suggest that multiple cancer types have relatively exclusive hub genes individually; however, the hub genes tended to cooperate with each other based on their functional relationships. We noted that, however, this didn’t mean actual PPIs in individual clinical cases, which needed to be validated by further experimental studies.

In summarising PPI networks of pcPSGs, we found nine gene clusters and created subnetworks based on inter-connectedness of genes (Fig. 4a). Partitioning of the network map by gene clusters of subnetworks showed appropriate division of the regional distribution of network genes. In addition, functional annotations acquired from GO term enrichment analysis of subnetwork genes appropriately divided and explained the function of network genes (Fig. 4b and Supplementary Fig. S10). In detail, cluster 1 mainly consisted of olfactory receptors (ORs) that are members of G protein-coupled receptors and known to function in the sensory perception of smell. ORs were previously reported as implausible and false-positive genes in cancer because they had low expression and were late in replication timing^26^. Our result also showed relatively low expression of ORs. However, a recent review that summarised the effect of ectopic expression of ORs showed abundant evidence that expression of ORs was up-regulated in multiple cancer types^27^. In prostate cancer, upregulation of ORs induced a chronic inflammatory response, promoted tumour growth, and correlated with cancer progression.

Clusters 2, 6, 7, and 8 were closely located in the network map, and the number of shared genes was high (Supplementary Fig. S9). These findings indicated that these clusters were functionally more interrelated. Cluster 8 might have roles in transcriptional regulation of cancer, tumour growth, and altered PCDs. Cluster 7 might contribute to tumour-associated angiogenesis. Clusters 2 and 6 might reflect dysregulated signalling and unregulated inflammation in cancer^28,29^. When the delicate mechanisms of signalling networks are distorted, they accelerate cancer progression via changes in the tumour microenvironment, angiogenesis, and inflammation^28^. Inflammation is the first defence mechanism of innate immunity. However, unregulated chronic inflammation induces malignant transformation of cells and upregulation of cytokines that contribute to tumour growth. Cytokines are secreted from tumour-associated macrophages, tumour-infiltrating lymphocytes, and cancer-associated fibroblasts^29^. Because cytokines have multiple roles in many kinds of cell activity, unregulated inflammation is associated with generation of reactive oxygen species (ROS), reactive nitrogen species, tumour growth, angiogenesis, and epithelial mesenchymal transition that promote invasiveness and metastasis^29^.

Genes in cluster 3 were involved in glycosylation processes such as sialylation, fucosylation, O-glycan processing, keratan sulphate biosynthesis, and ganglioside biosynthesis. Glycosylation is the enzymatic process that attaches saccharides to proteins, lipids, or other saccharides via glycosidic linkages^30^. These saccharides are called glycans, and a molecule to which one or more glycan units are covalently linked to a non-carbohydrate entity is called a glycoconjugate^31^. There are three major classes of glycoconjugates: glycoproteins, proteoglycans, and glycosphingolipids. The glycoproteins carry covalently attached glycans via nitrogen or oxygen linkages, which are known as N-glycans and O-glycans, respectively^30^. Proteoglycans have one or more glycosaminoglycans, such as keratan sulphate. Glycosphingolipids consist of a glycan attached to the lipid ceramide; when it contains sialic acid, it is called a ganglioside. Gangliosides are associated with receptor tyrosine kinases such as epidermal growth factor and insulin receptors^31^. Glycoconjugates are present on the cell surface and mediate cell adhesion and motility, as well as intracellular signalling. Tumour cells show aberrant expression of various glycans due to a wide range of glycosylation alterations that regulate the development and progression of cancer via cell interactions, extracellular communication, and immune reactions^30^.

Cluster 4 genes were enriched for GSH derivative biosynthesis, xenobiotic metabolism, and drug metabolism. GSH plays a major role in intracellular redox homeostasis, and participates in many metabolic processes^32^. Because GSH has an antioxidant effect on cells, decreased or depleted GSH increases cytotoxicity via oxidative stress, which is implicated in the progression of cancer^33^. On the other hand, malignant tumours have higher GSH levels compared to normal tissues, which is associated with multidrug and radiation resistance^34^. Increased GSH contributes to drug resistance by interacting with drugs/ROS, damage protection of proteins/DNA, or affecting the DNA repair process^32,35^. In the tree map block of “GSH derivative biosynthesis”, there were enriched GO terms related to acyl-chain remodelling and phospholipids. The phospholipid class and acyl-chain homeostasis are crucial for normal membrane function^36^. Cluster 4 genes might contribute to survival of cancer cells in the tumour microenvironment.

Clusters 5 and 9 were neighbours in the PPI network of pcPSGs. Genes expressed in muscle cells and associated with membrane transport were enriched in clusters 5 and 9, respectively, and both showed similar enriched GO terms related to actin filaments. This finding indicated that genes in these clusters contribute to intracellular transport systems in cancer cells and cancer cell migration. The actin cytoskeleton regulates cell polarity, adhesion, and migration^37^. With actin filaments, the non-muscle myosin motors function in endocytic, exocytic, and recycling pathways^38^. These are tightly organised; disruption of myosin and the actin cytoskeleton may interfere with normally well-regulated pathways. For example, disruption of myosin activity results in aberrant receptor internalisation and recycling, which can alter growth factor receptor signaling^38^. Similarly, distortion of myosin, the actin cytoskeleton, and other molecular activities results in tumour cell migration and invasion^38,39^.

In summary, the gene clusters of subnetworks showed the spatial organisation of the PPI network map of pcPSGs, but also divided and explained the function of the network via their distinct roles related to development and progression of cancer.

Genes that belonged to subnetworks were more highly expressed than other genes (Fig. 5). Shilien *et al*. previously showed that cancer cells were more transcriptionally active than normal cells by using a fraction of transcripts derived from cancer cells and fractions of cancer cells^6^. The subnetwork genes might contribute to the transcriptionally active state of cancer cells. Zhang *et al*. showed a positive correlation between the importance of a gene and its expression level^40^. In that study, the word “important” was defined as a sequence’s relevance to the fitness of the organism bearing the sequence, and protein importance was measured as protein dispensability^41^. This finding indicated that high expression of subnetwork genes reflected their biological benefits to cancer cells in the tumour microenvironment.

Investigation of the cluster occupation ratio of TCGA datasets showed that clusters 2, 6, 7, and 8 were involved in most of cancer types, and showed a narrower range of fluctuation in cluster occupation ratio (Fig. 6). This finding indicated that these clusters had a relatively high level of cluster sharing, which was more essential to most cancer types. This result was underpinned by the enrichment analyses for PCD, known, and candidate cancer genes (Tables 1 and 2). The enrichment analyses showed that all PCD, known, and candidate cancer genes were enriched in clusters 6, 7, and 8. Many types of PCD in cancer are well known as key players in ultimate decisions of cancer cell fate^42^. The PCD is involved in cancer initiation and progression. Enrichment of known and candidate cancer genes means that cancer driver genes are more likely to exist in those clusters.

The enrichment analyses also showed that PCD, known, and candidate cancer genes were enriched in PSGs and the PPI networks of pcPSGs. This result supports the close relationship between pcPSGs and cancer. Besides pcPSGs, we found evidence that ncPSGs were also related to cancer. We identified the significantly enriched KEGG pathway term “MicroRNAs in cancer”, which included 17 ncPSGs (Supplementary Table S13). In the summary of lncRNAs of PSGs, there were GO terms that were possibly related to cancer such as “DNA damage response, signal transduction by p53 class mediator”, “multicellular organismal growth”, “cell cycle”, “cell death”, and “cell division” (Supplementary Fig. S15). Among ncPSGs, pseudogenes accounted for 77.2% (Supplementary Fig. S13). Pseudogenes were once thought to be ‘junk’ DNA; however, it is now known that they affect many physiological and pathological processes at the DNA, RNA, and/or protein levels, especially in cancer^43,44^. Pseudogenic DNAs can interact with gene loci based on their sequence homology, which result in alteration of target sequences and/or transcriptional efficiency. Pseudogenic RNAs such as antisense RNAs, endogenous small-interference RNAs, competing endogenous RNAs, and chimeric RNAs, can act as post-transcriptional regulators. Although most pseudogenes have lost their ability to encode proteins, a few pseudogenes have retained or regained protein-coding ability^43^. Pseudogenic proteins can act as fully functional proteins in the wrong place and time, as antigens recognised by the immune system, and as partially functional proteins that interact with parental proteins, which affect the functions of their parental counterparts. Recently, classification of the major histological subtypes of endometrial cancer was reported using pseudogene expression^45^.

Our experimental design had a limitation in that it did not stratify genes and cancer types like the NBS method; however, our different approach to investigating cancer genes enabled us to identify pan-cancer hub genes and pan-cancer functional gene clusters based on the expression status of the primary tumour itself and gene-to-gene relationships in the biological network. Our findings also had high relevance to understanding gene expression profiles and biological pathways that are in common among diverse types of cancer.

## Materials and Methods

### TCGA data (somatic mutation, gene expression quantification, and metadata)

The Cancer Genome Atlas (TCGA) data used in this study were from the Genomic Data Commons (GDC) (https://gdc-portal.nci.nih.gov/) Data Release 4.0. The reference gene annotations of the GDC were from GENECODE^46^ Release 22 (GRCh38.p2). This was an evidence-based annotation of the human genome GRCh38, which was based on the data of Ensembl^47^ release 79. We used publicly available data on 33 types of cancer (Supplementary Table S1) for analyses.

For somatic mutation data, we used somatic variant calls in mutation annotation format (MAF), which were MuSE^48^ variant aggregation and masking data. There was one file for each dataset, and 33 files with 10,425 cases were downloaded (Supplementary Table S2).

For gene expression quantification data, we used pre-calculated gene expression values in fragments per kilobase of exon per million fragments (FPKM). For this study, we selected TCGA datasets for primary cancers such as ‘Primary Blood Derived Cancer - Peripheral Blood’, ‘Primary Tumour’, and ‘Additional - New Primary’. There were 9,922 files with 9,831 cases downloaded (Supplementary Table S3).

We used two types of metadata, which were biospecimen and annotation data. The biospecimen data was used for ID mapping of hierarchical biospecimen elements (dataset-patient (or case) -sample-portion-analyte-aliquot), and the annotation data was used for filtering somatic mutation and gene expression quantification data. There were 11,353 files of biospecimen data, and 33 annotation tables of 1,013 items with annotations (one file for each dataset) for somatic mutation data and one annotation table of 1,952 items with annotations for gene expression quantification data.

### Data analysis

A schematic diagram of the research process is shown in Fig. 1.

### Filtering of somatic mutation data

For somatic mutation data, we applied three types of filtering criteria, which were based on sample type, TCGA annotations, and read depth. First, we selected aliquots of primary cancers such as ‘Primary Blood Derived Cancer - Peripheral Blood’, ‘Primary Tumour’, and ‘Additional - New Primary’. Second, we excluded results of aliquots that had annotations except those with an arbitrary filtered-in category such as ‘Acceptable treatment for TCGA tumour’, ‘Alternate sample pipeline’, ‘Item in special subset’, and ‘Item is noncanonical’. Filtered out categories included critical flaws such as ‘Item may not meet study protocol’, ‘Barcode incorrect’, ‘BCR notification’, ‘Prior malignancy’, and so on. Third, we removed MuSE somatic mutation calls with t_depth (read-depth in the tumour) < 14 or n_depth (read-depth in the normal) < 8 from MAF files according to previous research^26^. Then, we selected genes at the intersection between GRCh38 Ensembl releases 79 and 86 to exclude misannotated genes.

### Filtering of gene expression quantification data

For gene expression quantification data, we applied the same first two filtering criteria used in filtering of somatic mutation data, which were filtering based on sample type and filtering based on TCGA annotations. Then, we randomly selected one transcriptome profile when a TCGA case had more than one transcriptome profile. Finally, we selected genes at the intersection between GRCh38 Ensemble releases 79 and 86.

### Weighted gene co-expression network analysis

After filtering, we performed quantile normalisation on expression quantification data for each TCGA dataset. This method is based on the concept of a quantile-quantile plot extended to multi-dimensions, which results in the same distribution of expression values of transcriptomes^49^.

To identify relatively important gene expression in TCGA datasets, we used the R-package program, Weighted Gene Co-expression Network Analysis (WGCNA)^14^ version 1.51. This program uses a weighted network (matrix of connection strengths) calculated from a correlation matrix of expression, rather than unweighted networks produced by dichotomizing the Pearson correlation matrix. The program produces a topological overlap measure (TOM)^50^ from the weighted network, which is used to define gene modules (clusters of highly interconnected genes) of weighted gene co-expression networks based on their dissimilarities.

To explain the process, genes that had excessive missing values were removed, and outlier samples were discarded (Supplementary Fig. S2). Then, we chose parameters that were used in calculation of connection strengths based on the approximate scale-free topology criteria. Network topology analysis of possible candidate soft-thresholding powers was performed, and a suitable soft-thresholding power was chosen (Supplementary Fig. S3 and Supplementary Table S7). Following calculation of the degree of connectivity that is the sum of the connection strengths with other network genes, a gene co-expression network was generated, and the genes clustered onto a TOM, based on their dissimilarities. Gene modules were assigned with an option “minimum module size” of 30, and genes with high connectivity to each other clustered at the same module (Supplementary Fig. S4). In this study, we used signed TOM to make a distinction between positive and negative correlations, and to take anti-reinforcing into account^51^.

Module hub genes were defined to be highly connected genes inside co-expression modules. In selection of module hub genes, we intended to assign an arbitrary similar number of genes to each dataset (about 5% of genes), and within bounds we restricted the maximum rank of each module considering its size because the number of modules varied for each dataset. Then, we applied cut off criteria, which were module membership (eigengene-based connectivity kME) ≥ 0.9 and the *p*-value of module membership ≤ 0.01.

### Pan-cancer-wide selected genes (PSGs)

For each TCGA dataset, we found genes at the intersection between genes associated with somatic mutations and genes that were WGCNA module hubs. Then, we generated a set of PSGs by integrating these genes. PSGs were classified into protein-coding and non-coding genes according to categorisation of the Ensembl database. Non-coding genes were also classified into four categories (pseudogene, short non-coding, long non-coding, and problematic) and their sub categories (Supplementary Table S11).

### Protein-protein interaction (PPI) network of protein-coding PSGs (pcPSGs)

For pcPSGs, we generated a single-depth PPI network and discovered subnetworks. We used information of PPI from STRING V10^17^ with stringent cut off that was a combined interaction score ≥ 900. To find gene clusters and create subnetworks, we used MCODE^15^ plugin of Cytoscape^52^. The program options ‘fluff’ and ‘K-core = 10’ were used to increase the size of subnetworks and filter out clusters lacking a maximally interconnected node of at least 10 degrees of edges. Because the ‘fluff’ option was used, genes of clusters were partially overlapped.

### Gene Ontology (GO) term enrichment analysis and characteristics investigation of subnetworks

GO term enrichment analysis of subnetwork genes was performed using DAVID Bioinformatics Resources 6.8^18^. We used the results of ‘GOTERM_BP_DIRECT’ with Bonferroni’s adjusted *p*-value ≤ 0.05. Then we summarised GO terms with REVIGO^53^ that removed redundant GO terms and visualised remaining GO terms in a tree map. The *p*-values of enriched GO terms were used to determine the size of tree map block in REVIGO.

To investigate the characteristics of gene clusters of subnetworks, we performed several analyses. Using the empirical cumulative distribution function (ECDF) of ggplot2^54^, an R-package program, expression pattern was observed between genes of clusters (entire clusters or specific cluster) and genes that were not in clusters. We performed one-tailed Fisher’s exact test for assessing enrichment of known and candidate cancer genes and PCD genes in the gene clusters. We set the *p*-value < 0.05 as a criterion of significant enrichment. The list of known and candidate cancer genes was from NCG 5.0^55^, and the list of PCD genes was from KEGG pathway^16^ and previous studies^56,57^. We considered apoptosis (type 1 PCD), autophagy (type 2 PCD), and programmed necrosis (type 3 PCD) as PCD, and made programmed necrosis consist of necroptosis and pyroptosis according to a previous study^58^ (Supplementary Data S4).

### Summarisation of non-coding PSGs (ncPSGs)

Non-coding PSGs (ncPSGs) were categorised into four gene type categories and 19 gene types according to RNA annotations in the Ensemble database (Supplementary Table S12). To summarise ncPSGs, we performed KEGG pathway term enrichment analysis using DAVID Bioinformatics Resources 6.8, and the results of ‘KEGG_PATHWAY’ with Bonferroni adjusted *p*-value ≤ 0.05. For lncRNAs of PSGs, we used LncRNA Ontology^19^, which is a functional annotation database of lncRNA that shows the function as a GO term. The GO terms of lncRNAs were summarised with REVIGO. The frequency of GO terms presented in the results of LncRNA Ontology was used to determine the size of tree map block in REVIGO.

## Electronic supplementary material

Supplementary Information

Supplementary Data S1

Supplementary Data S2

Supplementary Data S3

Supplementary Data S4
