# Supplementary material for: Pan-cancer analysis of somatic mutations and transcriptomes reveals common functional gene clusters shared by multiple cancer types
Source: Sci Rep. 2018 Apr 16;8:6041. doi: 10.1038/s41598-018-24379-y (PMC5902616; doi:10.1038/s41598-018-24379-y)
Supplement: Supplementary file 1 — Supplementary Information [file 41598_2018_24379_MOESM1_ESM.pdf]

Supplementary Information

Pan-cancer analysis of somatic mutations and transcriptomes reveals common functional clusters shared by multiple caner types

Hyeongmin Kim and Yong-Min Kim*

Korean Bioinformation Center, Korea Research Institute of Bioscience and Biotechnology, Daejeon 34141, Korea

*To whom correspondence should be addressed, Tel. +82-42-870-8534 Email. ymkim@kribb.re.kr

Supplementary Figures

SUPPLEMENTARY FIGURE S1 3
SUPPLEMENTARY FIGURE S2 4
SUPPLEMENTARY FIGURE S3 37
SUPPLEMENTARY FIGURE S4 70
SUPPLEMENTARY FIGURE S5 103
SUPPLEMENTARY FIGURE S6 104
SUPPLEMENTARY FIGURE S7 105
SUPPLEMENTARY FIGURE S8 106
SUPPLEMENTARY FIGURE S9 115
SUPPLEMENTARY FIGURE S10 116
SUPPLEMENTARY FIGURE S11 125
SUPPLEMENTARY FIGURE S12 134
SUPPLEMENTARY FIGURE S13 135
SUPPLEMENTARY FIGURE S14 136
SUPPLEMENTARY FIGURE S15 137

Supplementary Tables

SUPPLEMENTARY TABLE S1 138
SUPPLEMENTARY TABLE S2 139
SUPPLEMENTARY TABLE S3 141
SUPPLEMENTARY TABLE S4 142
SUPPLEMENTARY TABLE S5 143
SUPPLEMENTARY TABLE S6 144
SUPPLEMENTARY TABLE S7 145
SUPPLEMENTARY TABLE S8 146
SUPPLEMENTARY TABLE S9 147
SUPPLEMENTARY TABLE S10 148
SUPPLEMENTARY TABLE S11 149
SUPPLEMENTARY TABLE S12 150
SUPPLEMENTARY TABLE S13 151

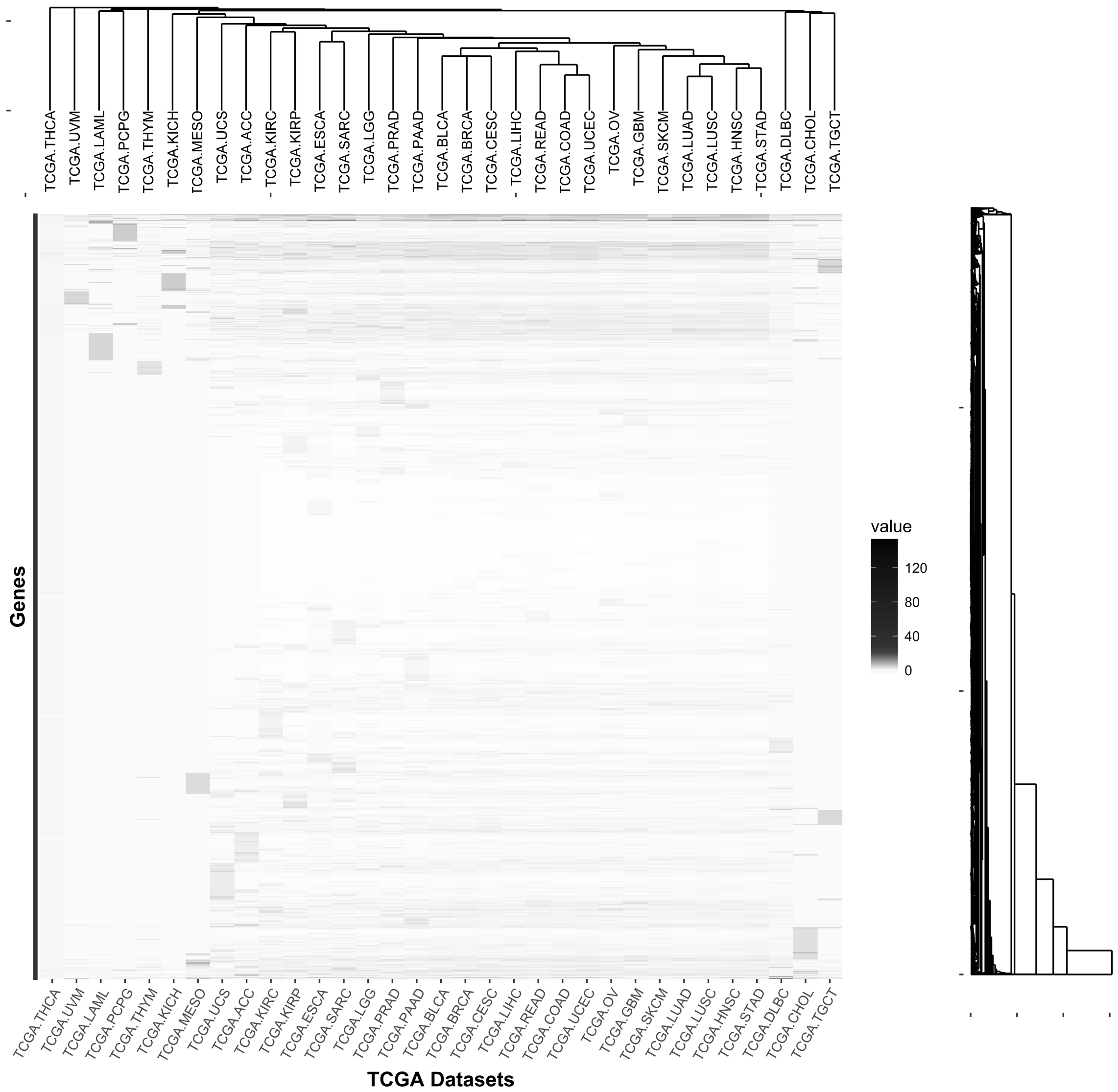

Supplementary Figure S1. Number of variants of gene per aliquot after filtering.

Heatmap of normalised values of somatic mutation calls of PSGs. TCGA datasets are shown on the x-axis and genes are on the y-axis. A dendrogram at the top of the heatmap show clustering of the datasets. A dendrogram on the right of the heatmap show clustering of the genes.

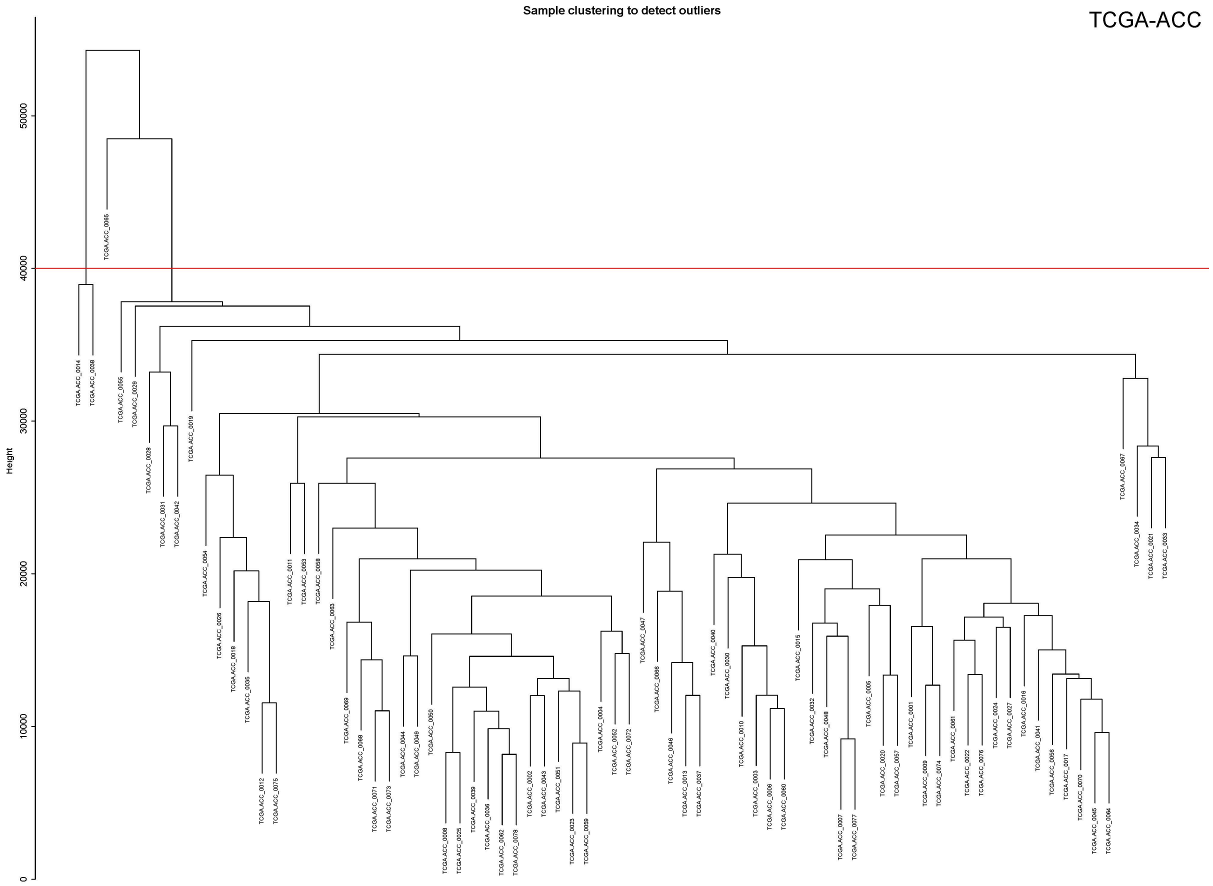

Supplementary Figure S2.1. Sample clustering to detect outliers.

Sample clustering is shown in a dendrogram. Arbitrary cut off value to exclude outlier is shown as red horizontal line. The name of TCGA dataset is located to the right top corner.

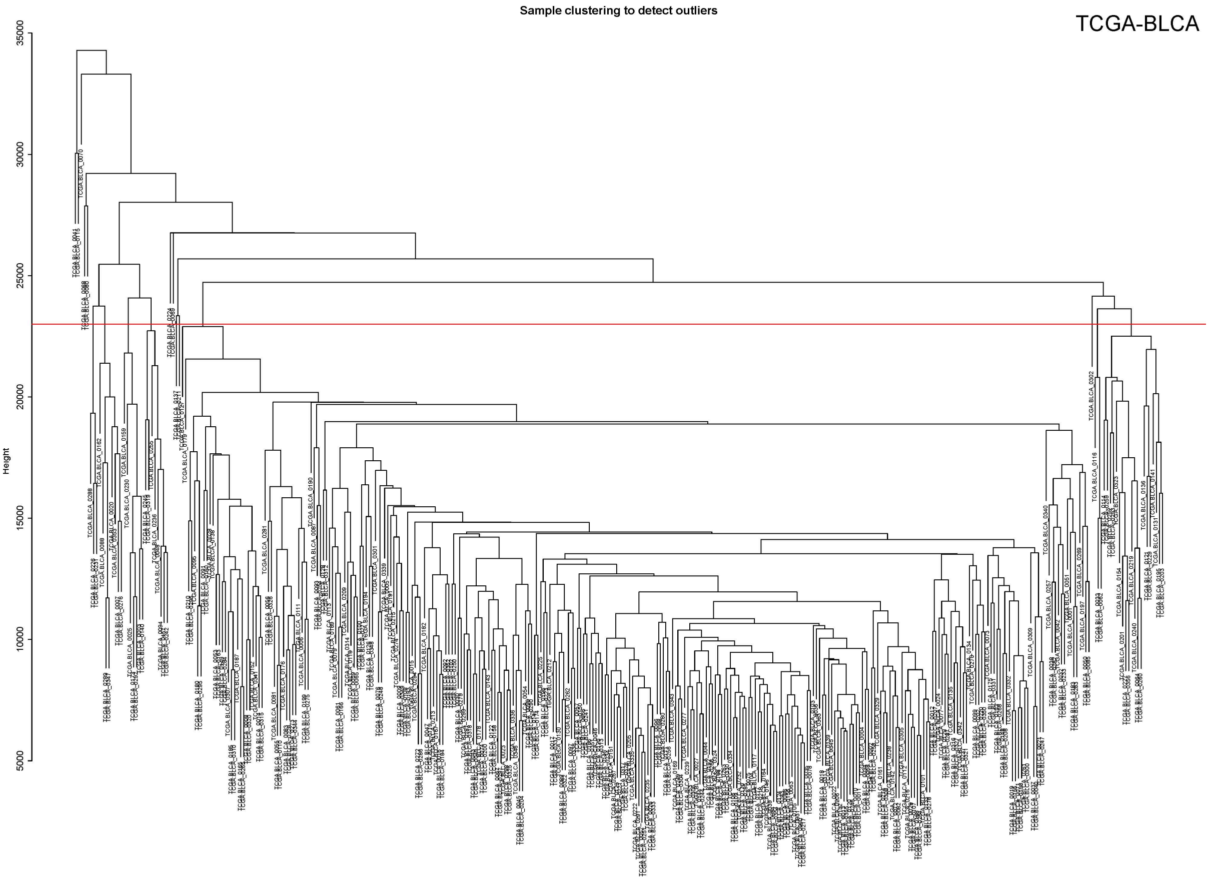

Supplementary Figure S2.2. Sample clustering to detect outliers.

Sample clustering is shown in a dendrogram. Arbitrary cut off value to exclude outlier is shown as red horizontal line. The name of TCGA dataset is located to the right top corner.

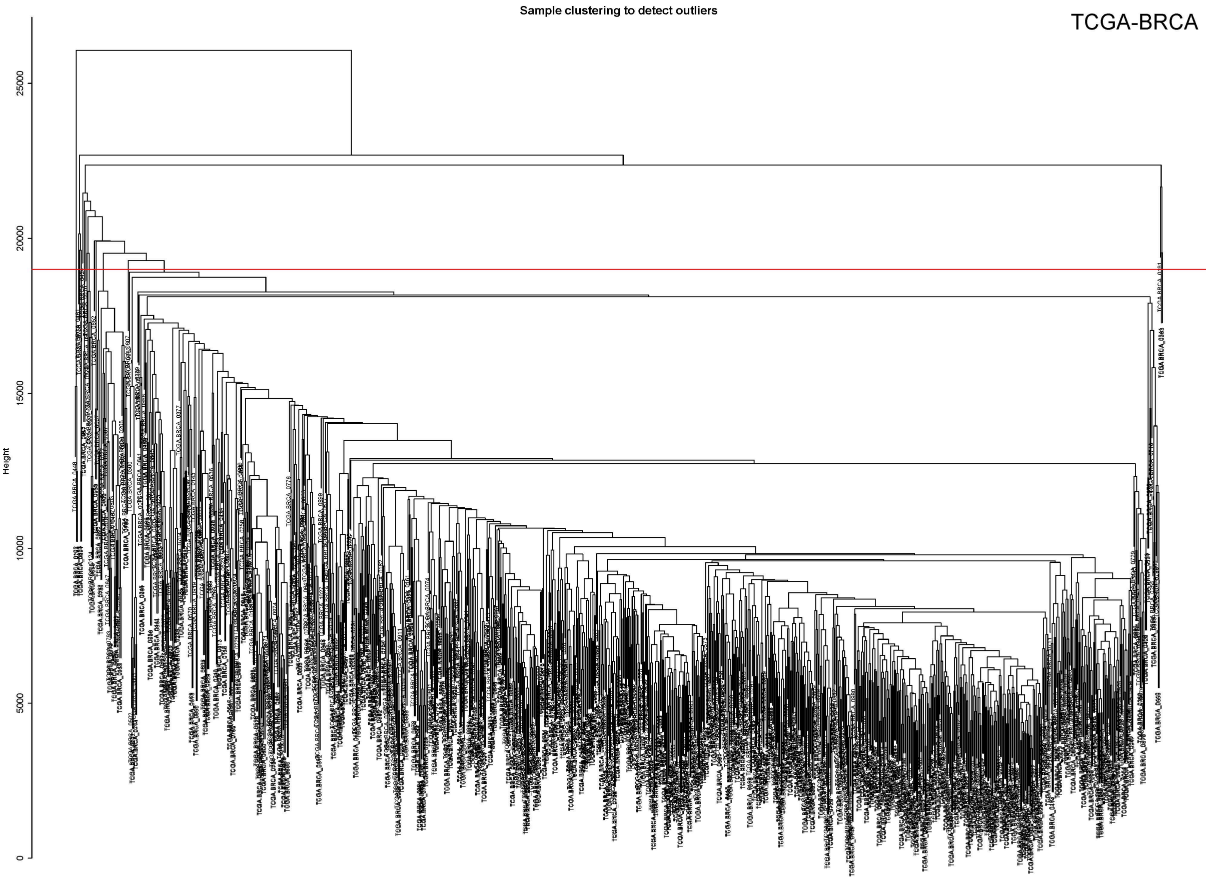

Supplementary Figure S2.3. Sample clustering to detect outliers.

Sample clustering is shown in a dendrogram. Arbitrary cut off value to exclude outlier is shown as red horizontal line. The name of TCGA dataset is located to the right top corner.

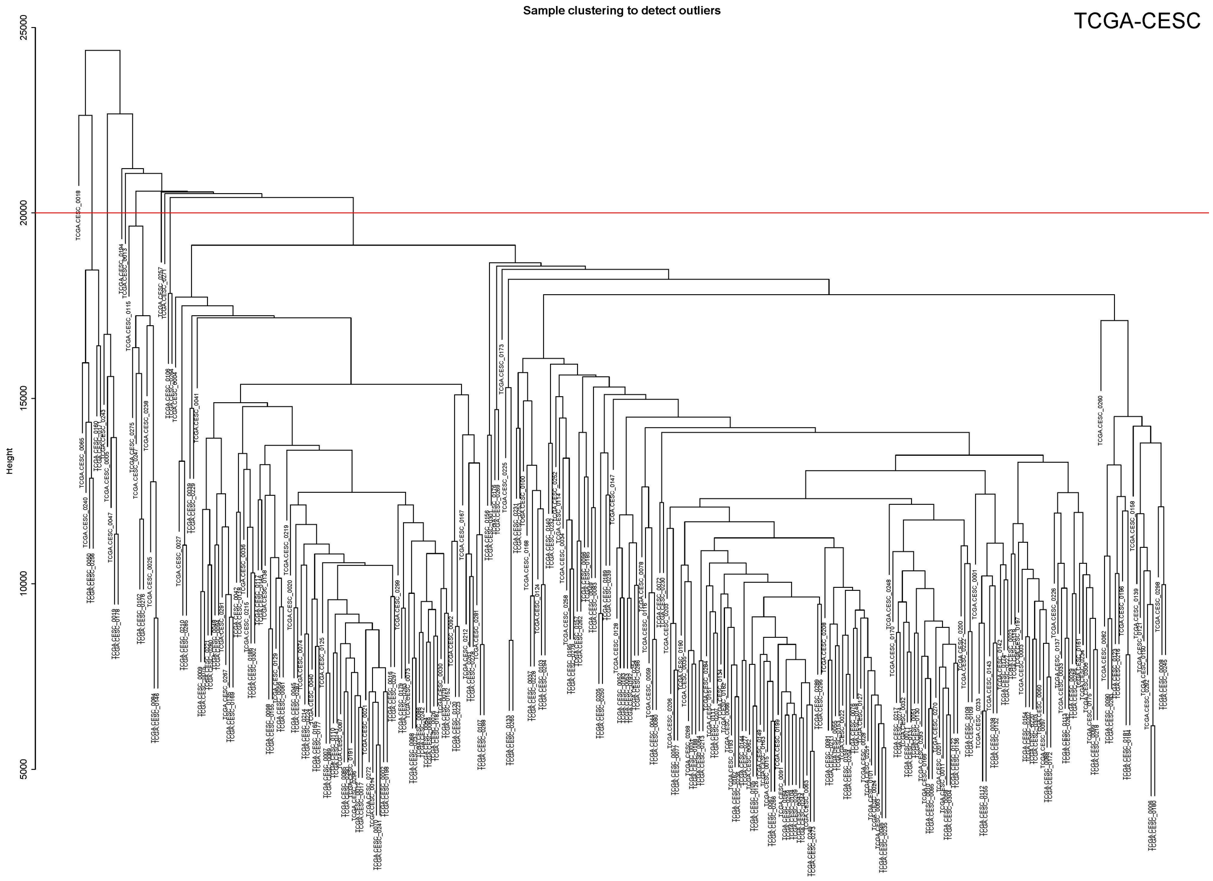

Supplementary Figure S2.4. Sample clustering to detect outliers.

Sample clustering is shown in a dendrogram. Arbitrary cut off value to exclude outlier is shown as red horizontal line. The name of TCGA dataset is located to the right top corner.

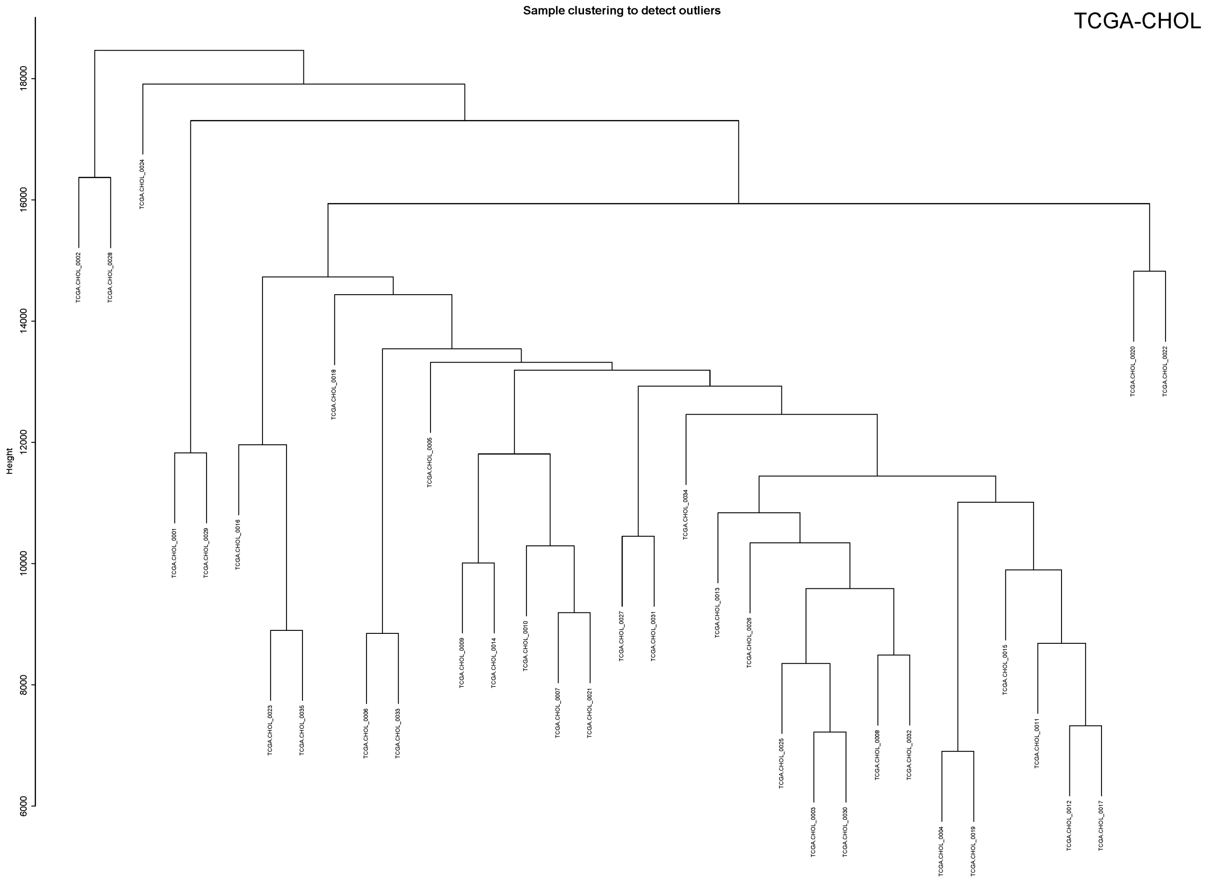

Supplementary Figure S2.5. Sample clustering to detect outliers.

Sample clustering is shown in a dendrogram. Arbitrary cut off value to exclude outlier is shown as red horizontal line. The name of TCGA dataset is located to the right top corner.

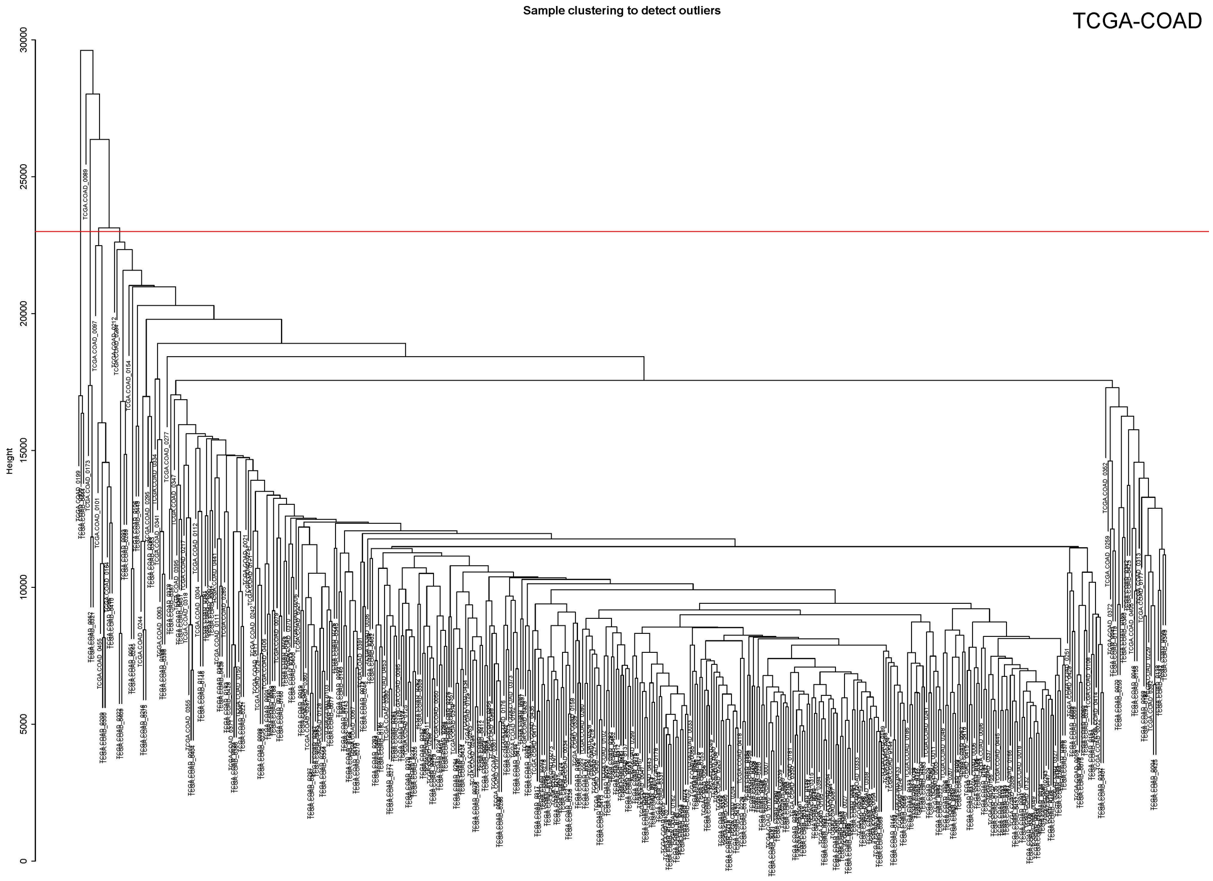

Supplementary Figure S2.6. Sample clustering to detect outliers.

Sample clustering is shown in a dendrogram. Arbitrary cut off value to exclude outlier is shown as red horizontal line. The name of TCGA dataset is located to the right top corner.

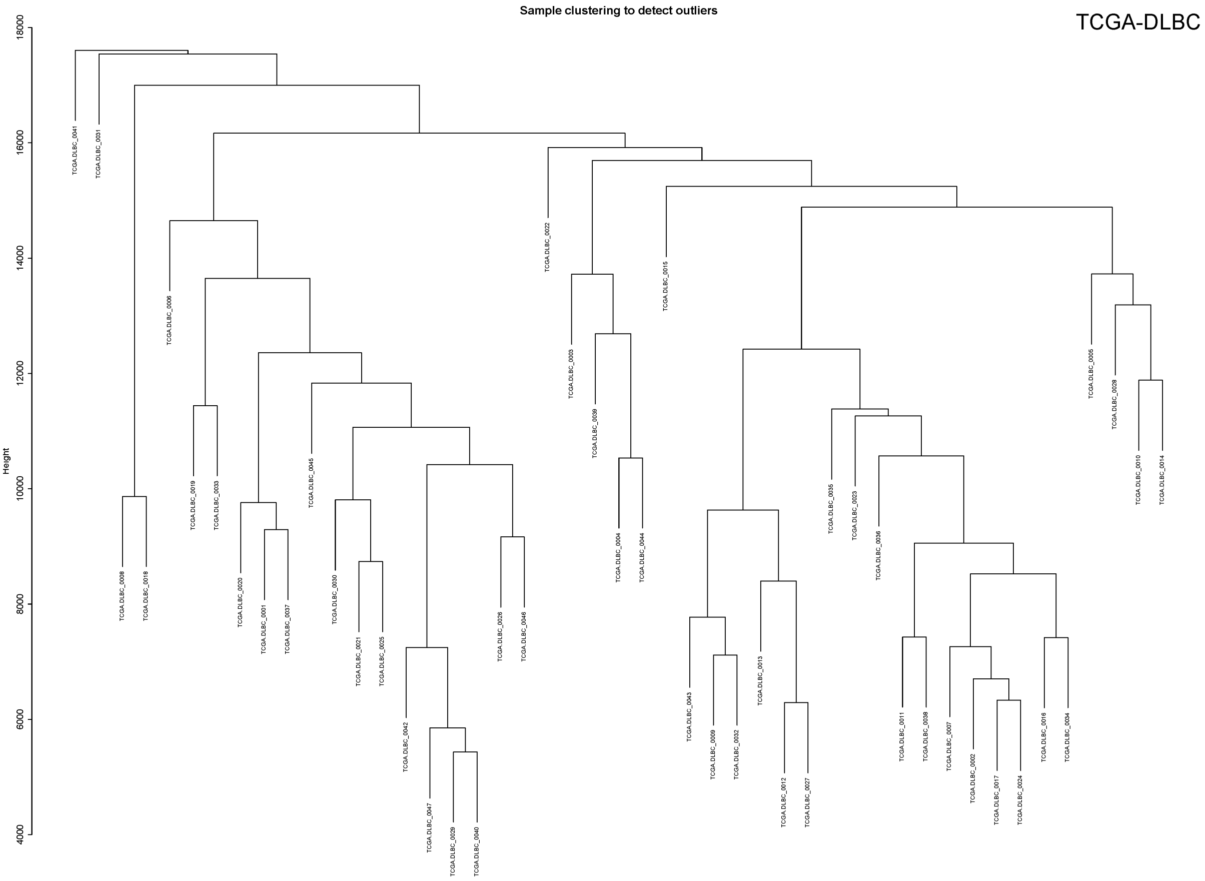

Supplementary Figure S2.7. Sample clustering to detect outliers.

Sample clustering is shown in a dendrogram. Arbitrary cut off value to exclude outlier is shown as red horizontal line. The name of TCGA dataset is located to the right top corner.

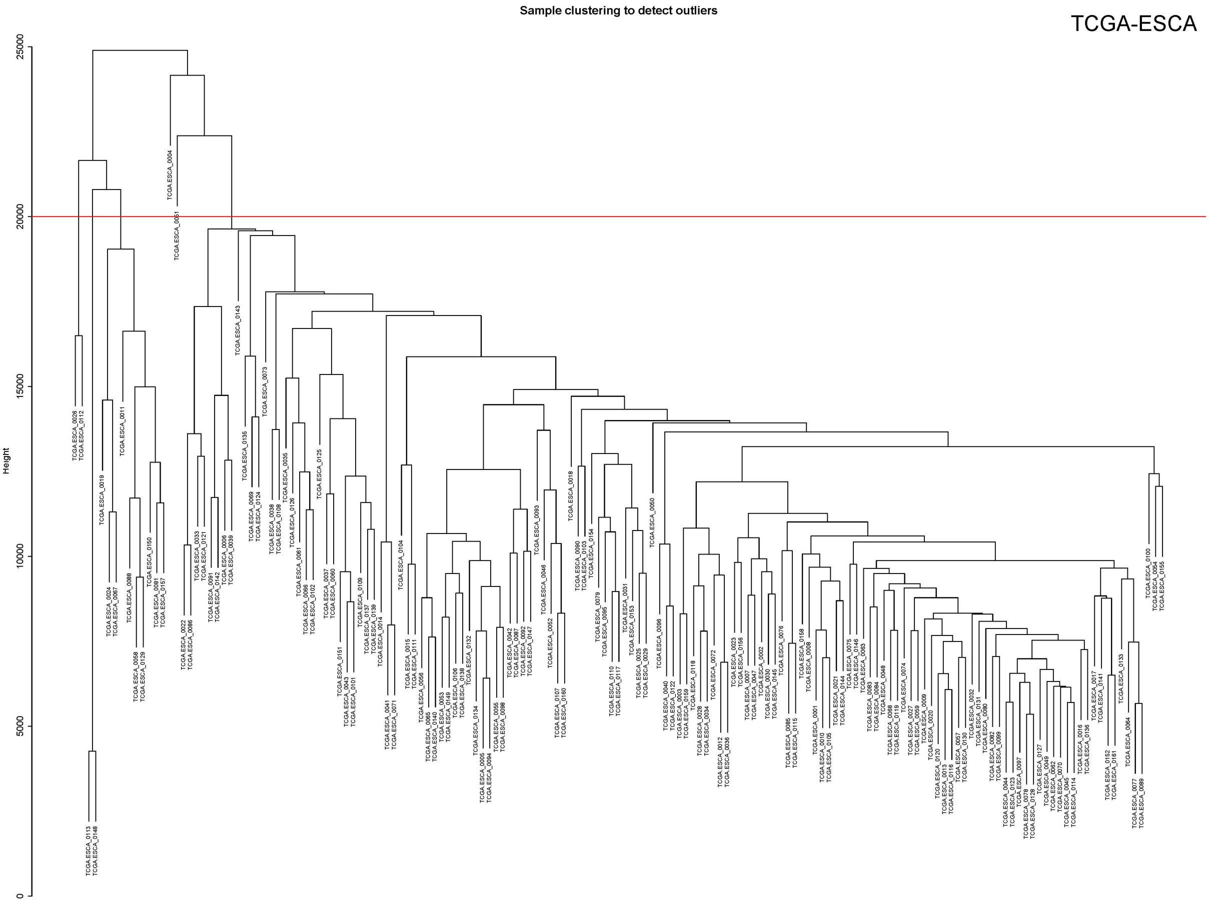

Supplementary Figure S2.8. Sample clustering to detect outliers.

Sample clustering is shown in a dendrogram. Arbitrary cut off value to exclude outlier is shown as red horizontal line. The name of TCGA dataset is located to the right top corner.

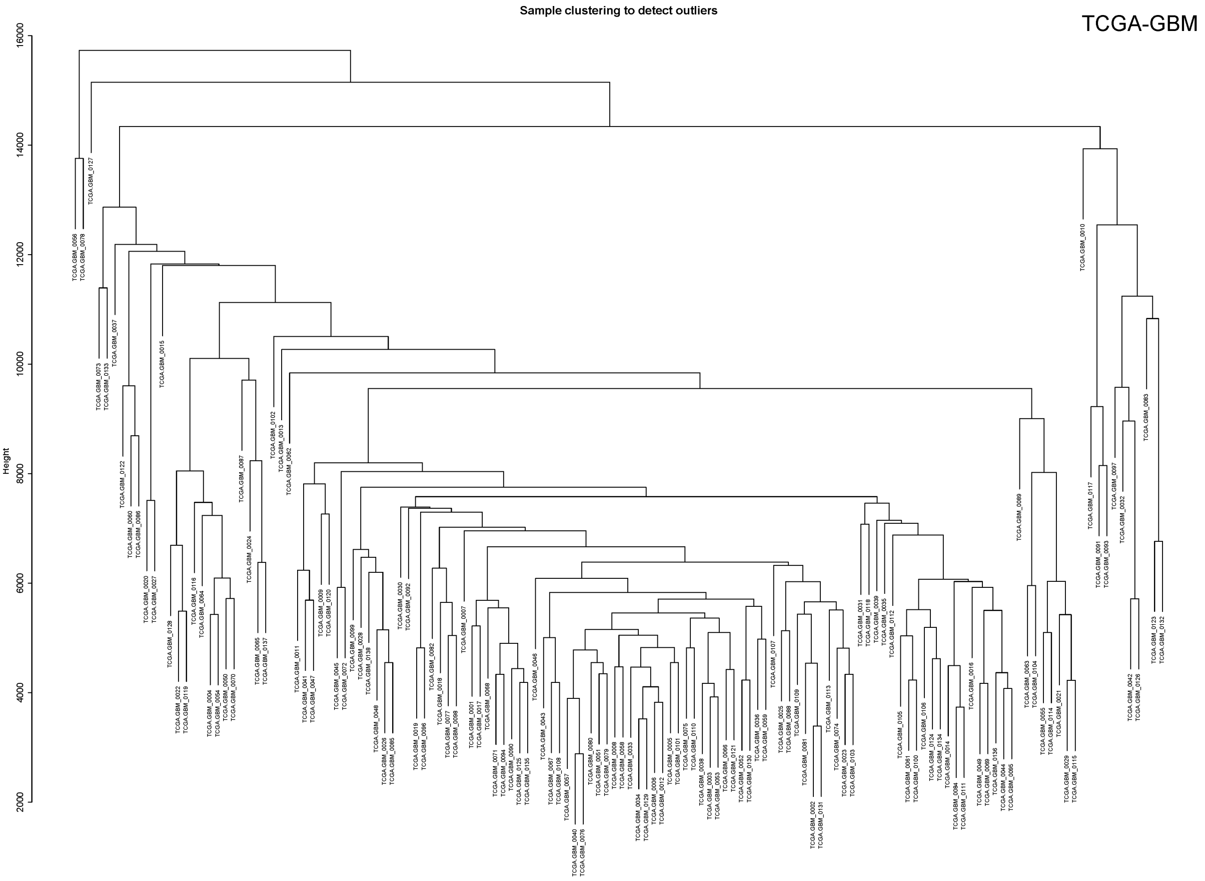

Supplementary Figure S2.9. Sample clustering to detect outliers.

Sample clustering is shown in a dendrogram. Arbitrary cut off value to exclude outlier is shown as red horizontal line. The name of TCGA dataset is located to the right top corner.

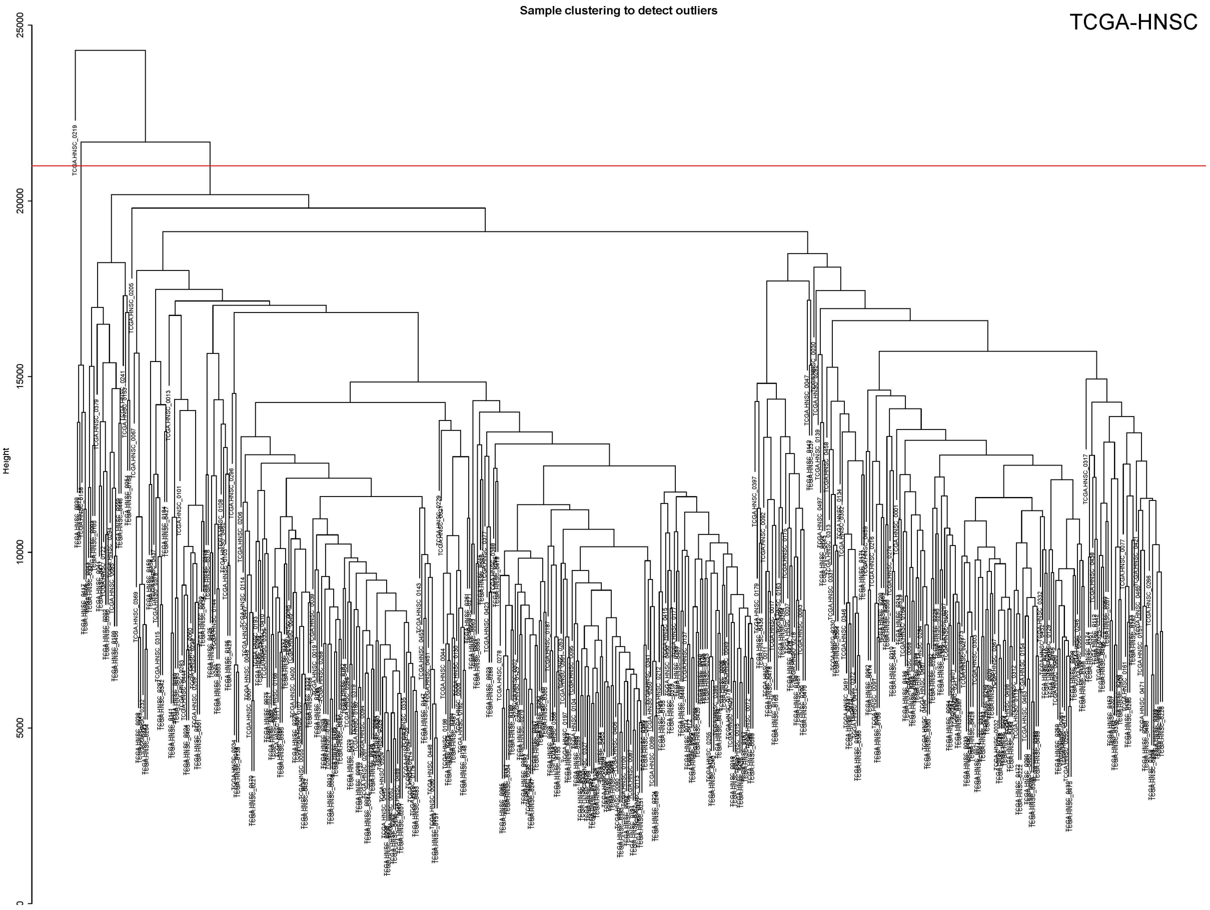

Supplementary Figure S2.10. Sample clustering to detect outliers.

Sample clustering is shown in a dendrogram. Arbitrary cut off value to exclude outlier is shown as red horizontal line. The name of TCGA dataset is located to the right top corner.

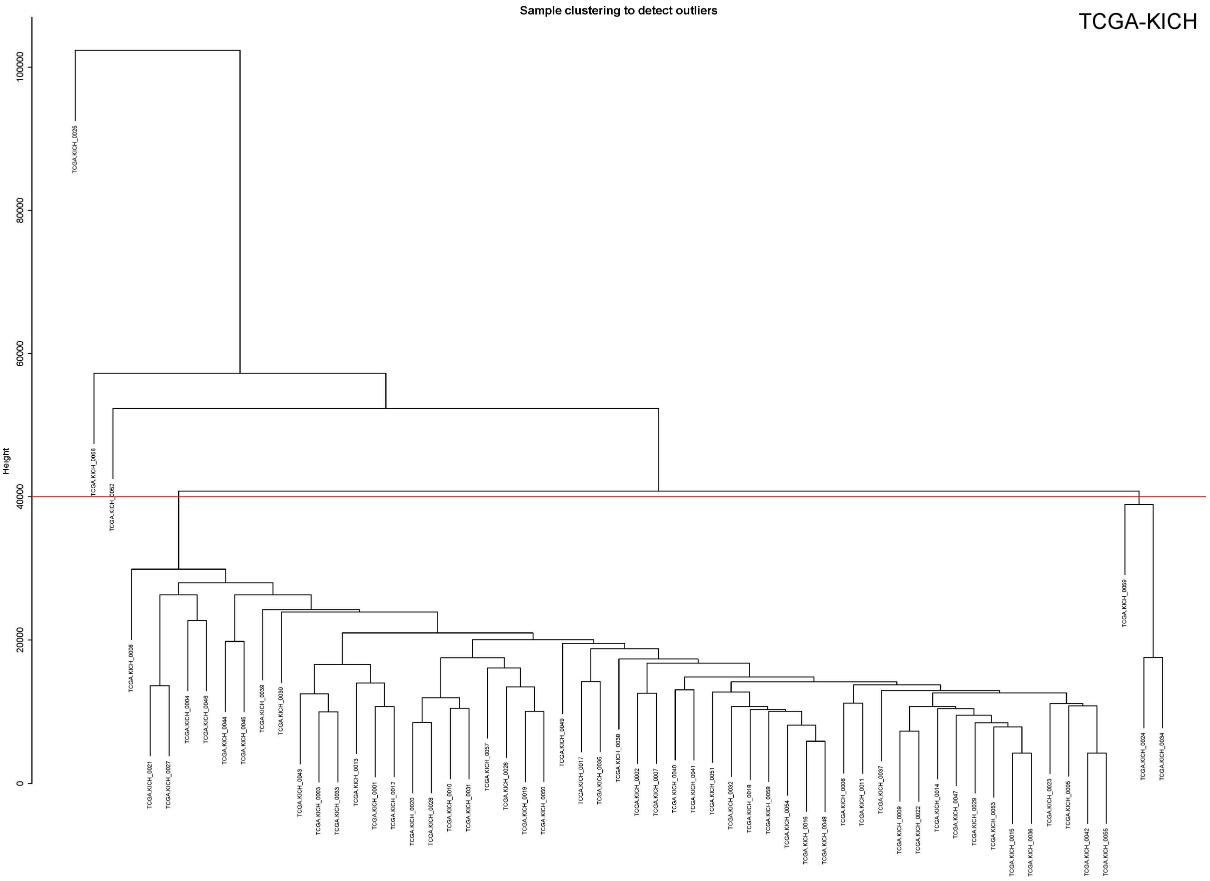

Supplementary Figure S2.11. Sample clustering to detect outliers.

Sample clustering is shown in a dendrogram. Arbitrary cut off value to exclude outlier is shown as red horizontal line. The name of TCGA dataset is located to the right top corner.

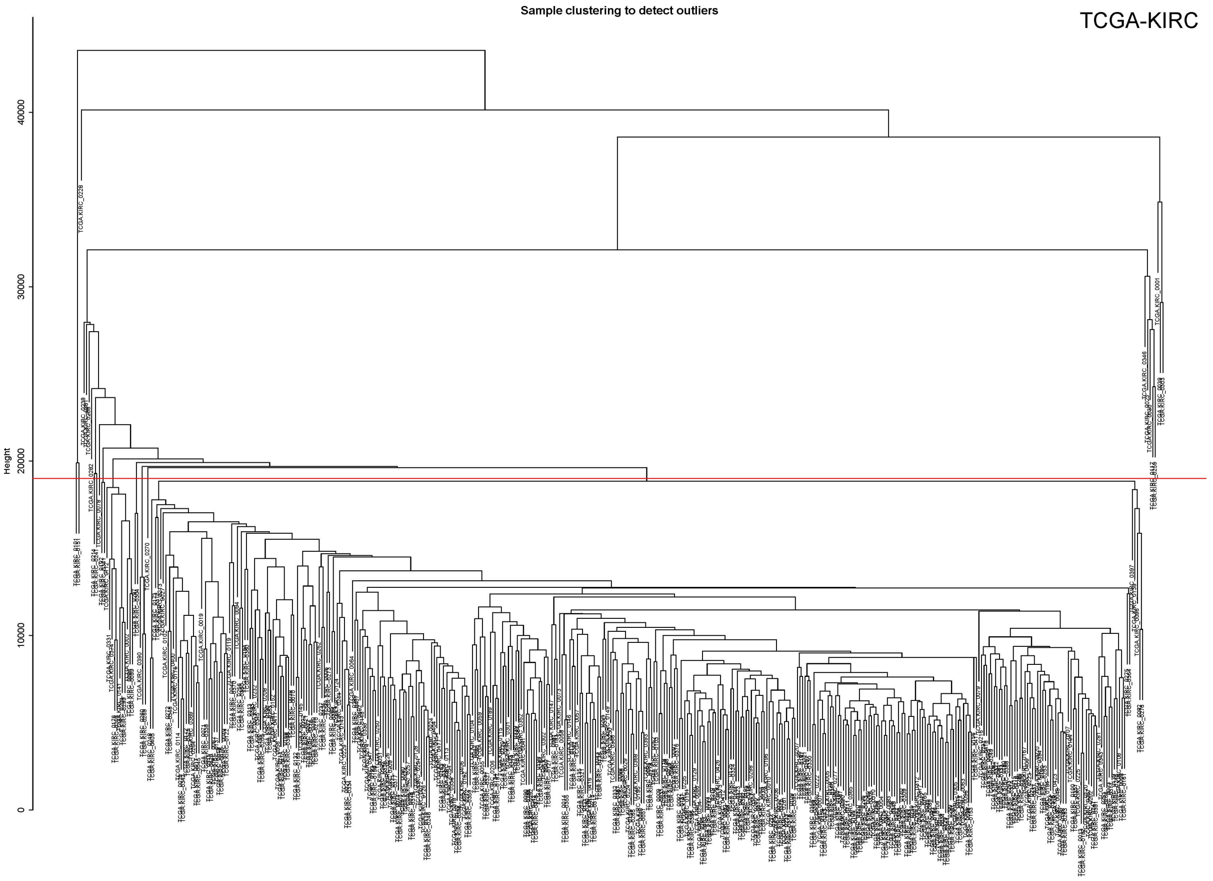

Supplementary Figure S2.12. Sample clustering to detect outliers.

Sample clustering is shown in a dendrogram. Arbitrary cut off value to exclude outlier is shown as red horizontal line. The name of TCGA dataset is located to the right top corner.

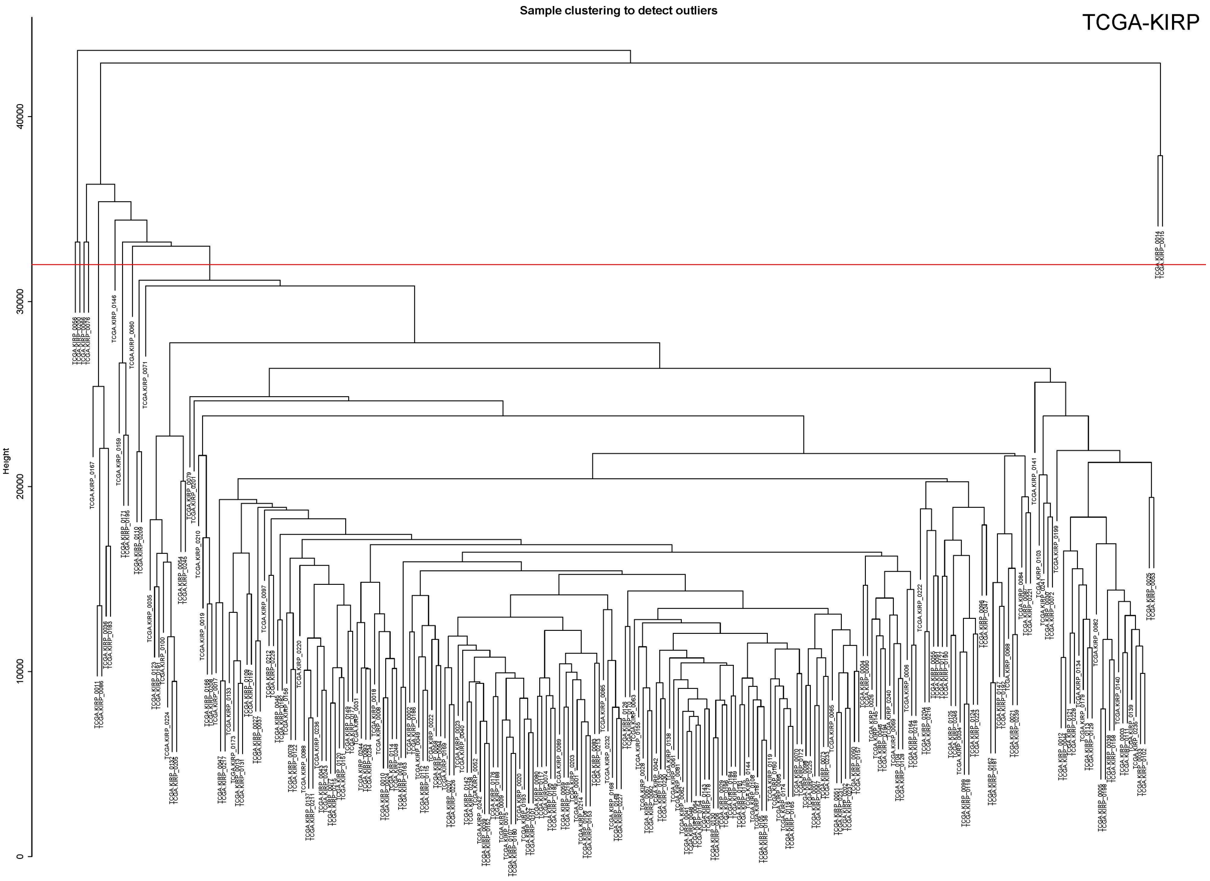

Supplementary Figure S2.13. Sample clustering to detect outliers.

Sample clustering is shown in a dendrogram. Arbitrary cut off value to exclude outlier is shown as red horizontal line. The name of TCGA dataset is located to the right top corner.

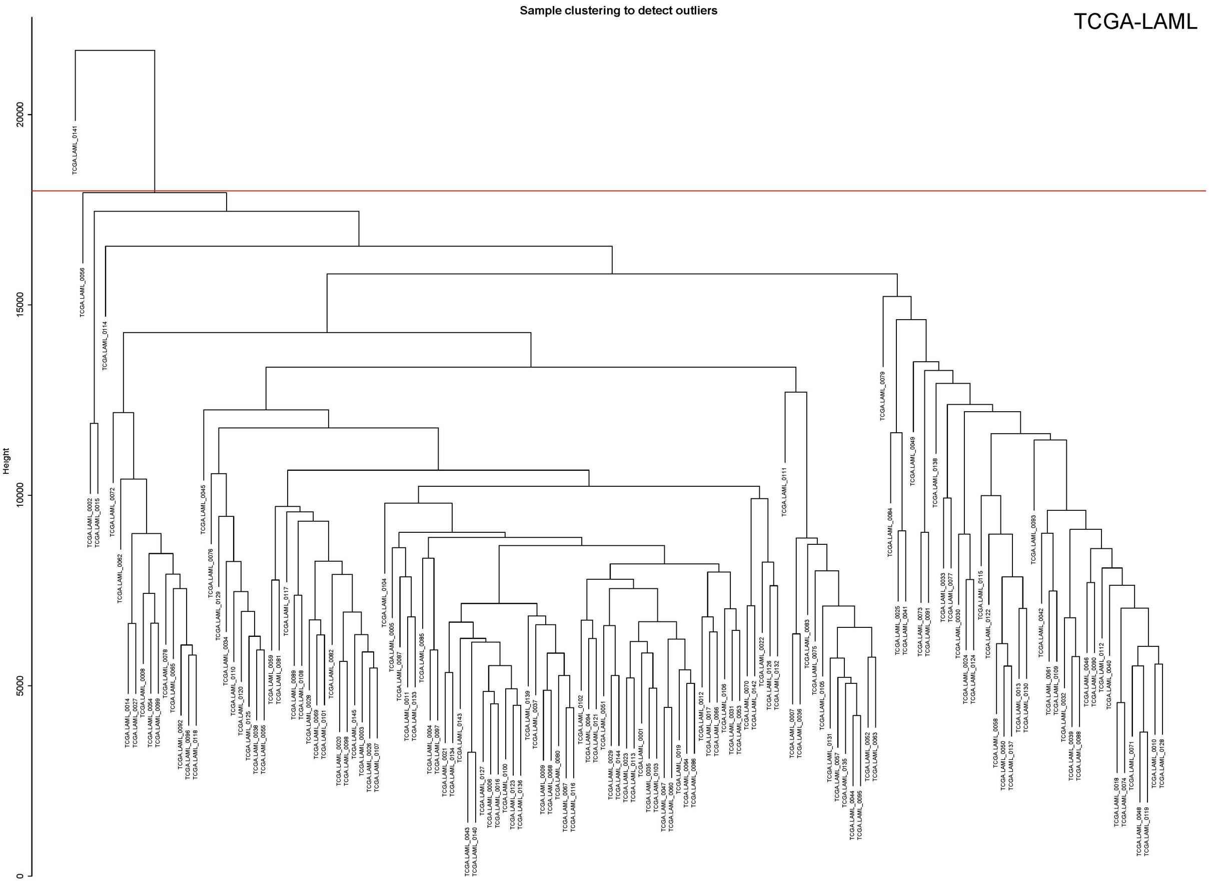

Supplementary Figure S2.14. Sample clustering to detect outliers.

Sample clustering is shown in a dendrogram. Arbitrary cut off value to exclude outlier is shown as red horizontal line. The name of TCGA dataset is located to the right top corner.

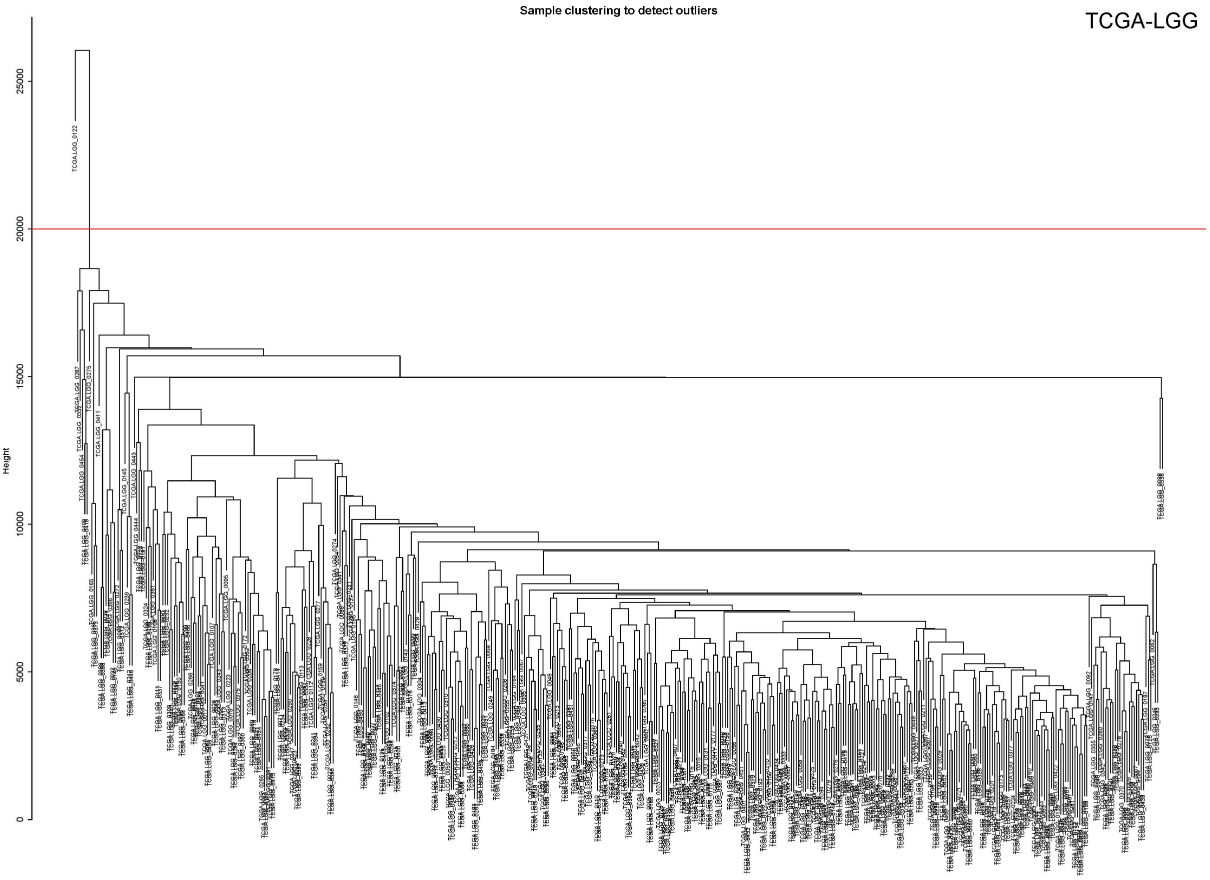

Supplementary Figure S2.15. Sample clustering to detect outliers.

Sample clustering is shown in a dendrogram. Arbitrary cut off value to exclude outlier is shown as red horizontal line. The name of TCGA dataset is located to the right top corner.

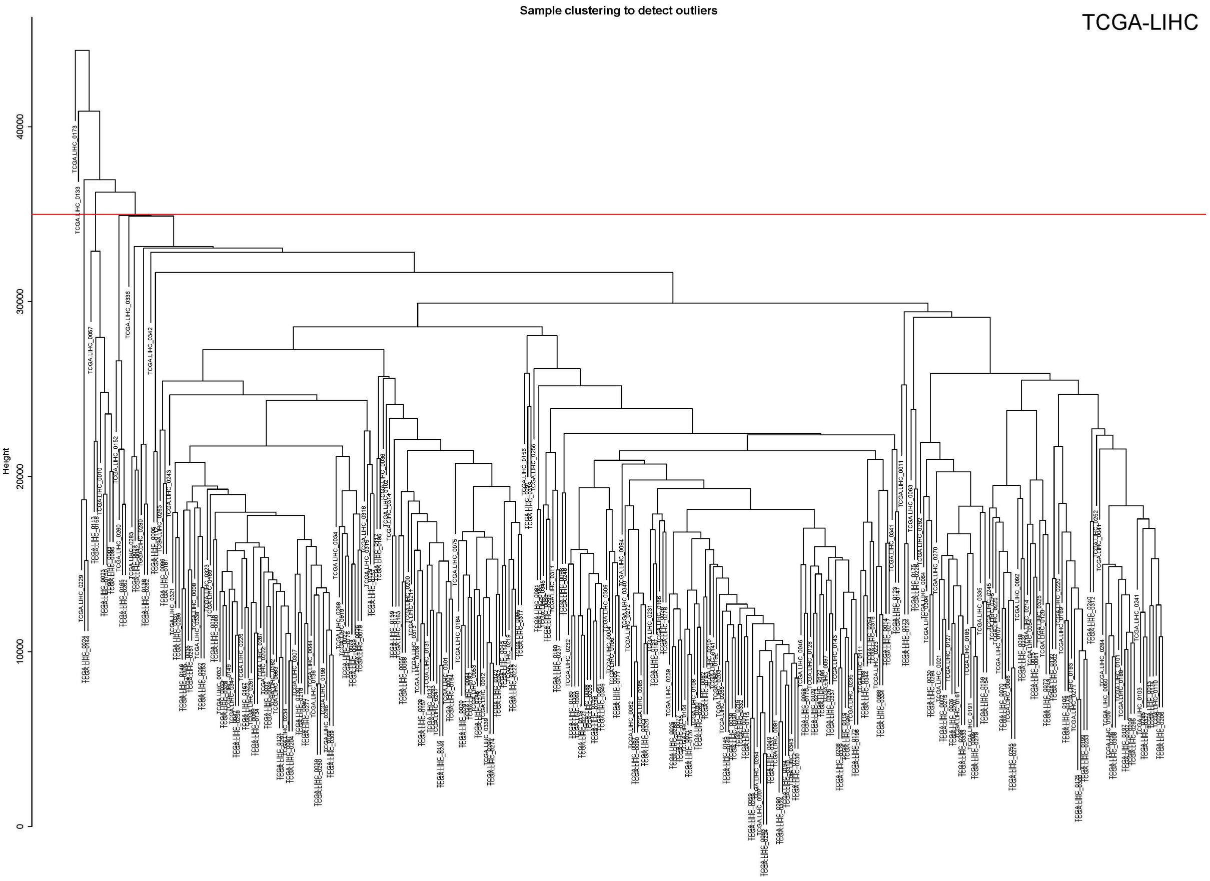

Supplementary Figure S2.16. Sample clustering to detect outliers.

Sample clustering is shown in a dendrogram. Arbitrary cut off value to exclude outlier is shown as red horizontal line. The name of TCGA dataset is located to the right top corner.

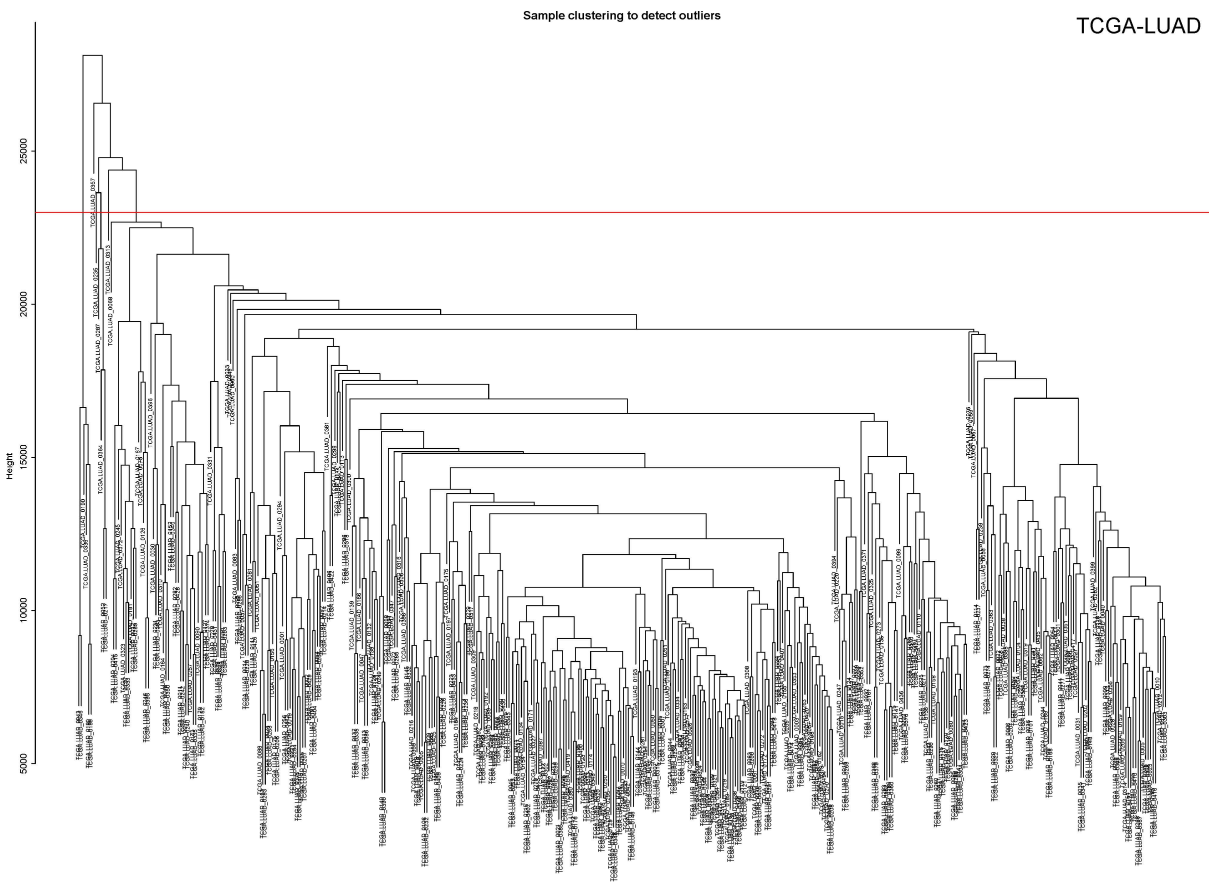

Supplementary Figure S2.17. Sample clustering to detect outliers.

Sample clustering is shown in a dendrogram. Arbitrary cut off value to exclude outlier is shown as red horizontal line. The name of TCGA dataset is located to the right top corner.

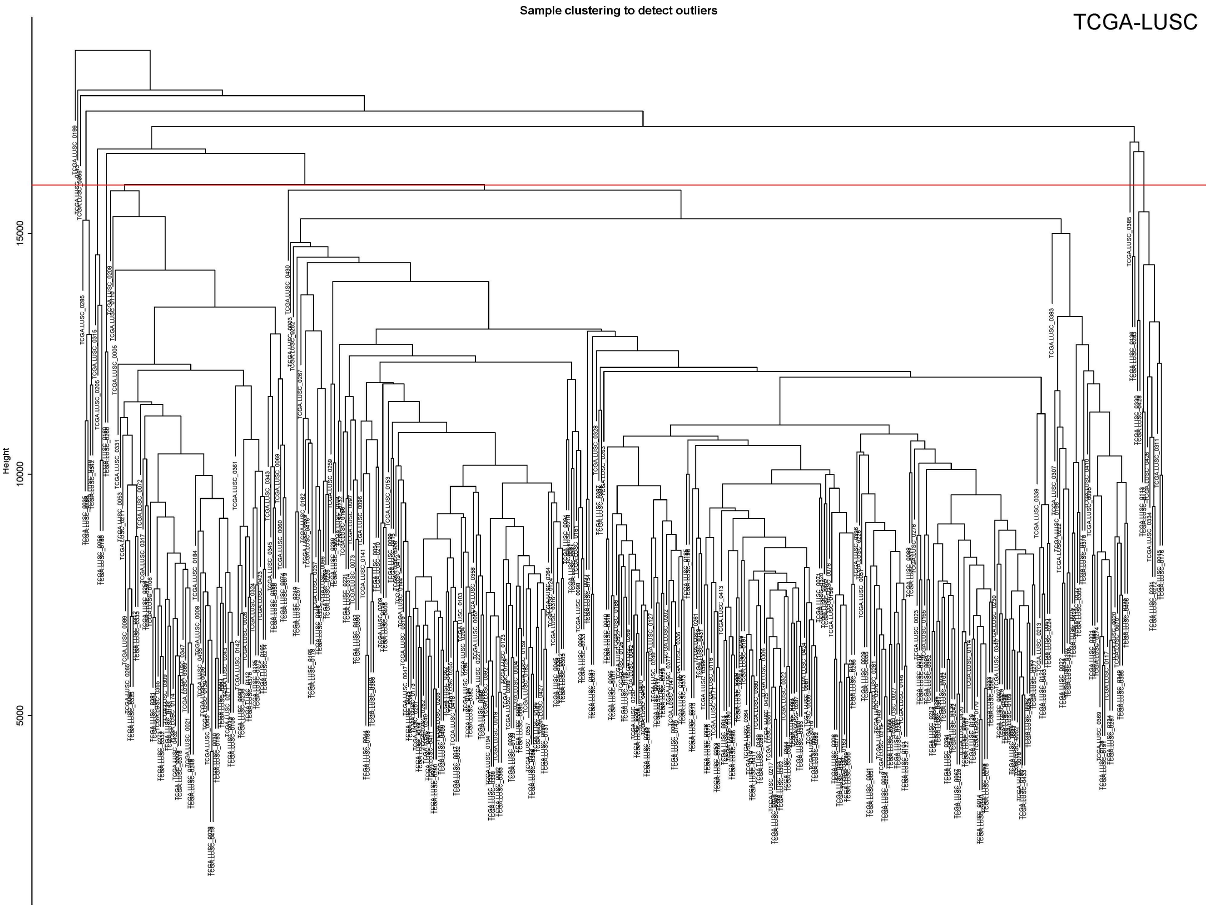

Supplementary Figure S2.18. Sample clustering to detect outliers.

Sample clustering is shown in a dendrogram. Arbitrary cut off value to exclude outlier is shown as red horizontal line. The name of TCGA dataset is located to the right top corner.

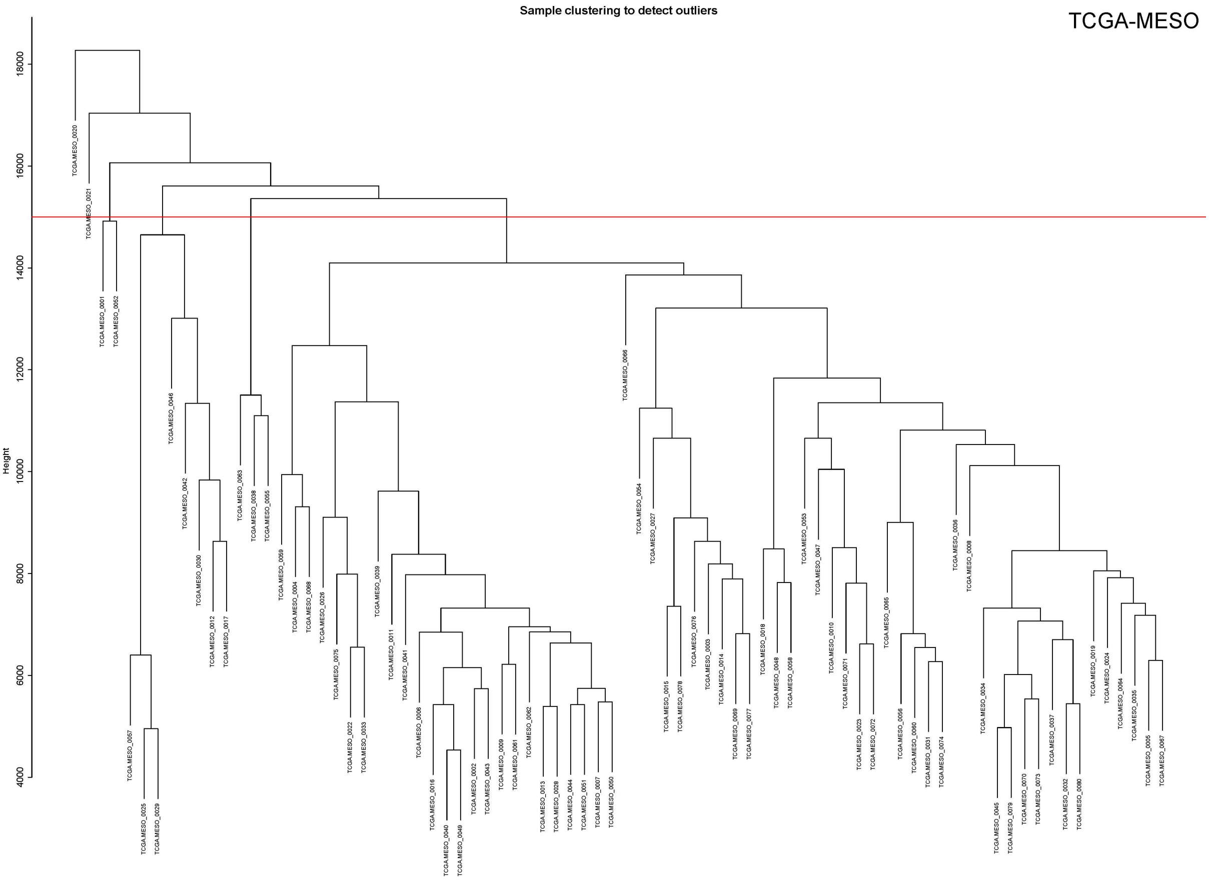

Supplementary Figure S2.19. Sample clustering to detect outliers.

Sample clustering is shown in a dendrogram. Arbitrary cut off value to exclude outlier is shown as red horizontal line. The name of TCGA dataset is located to the right top corner.

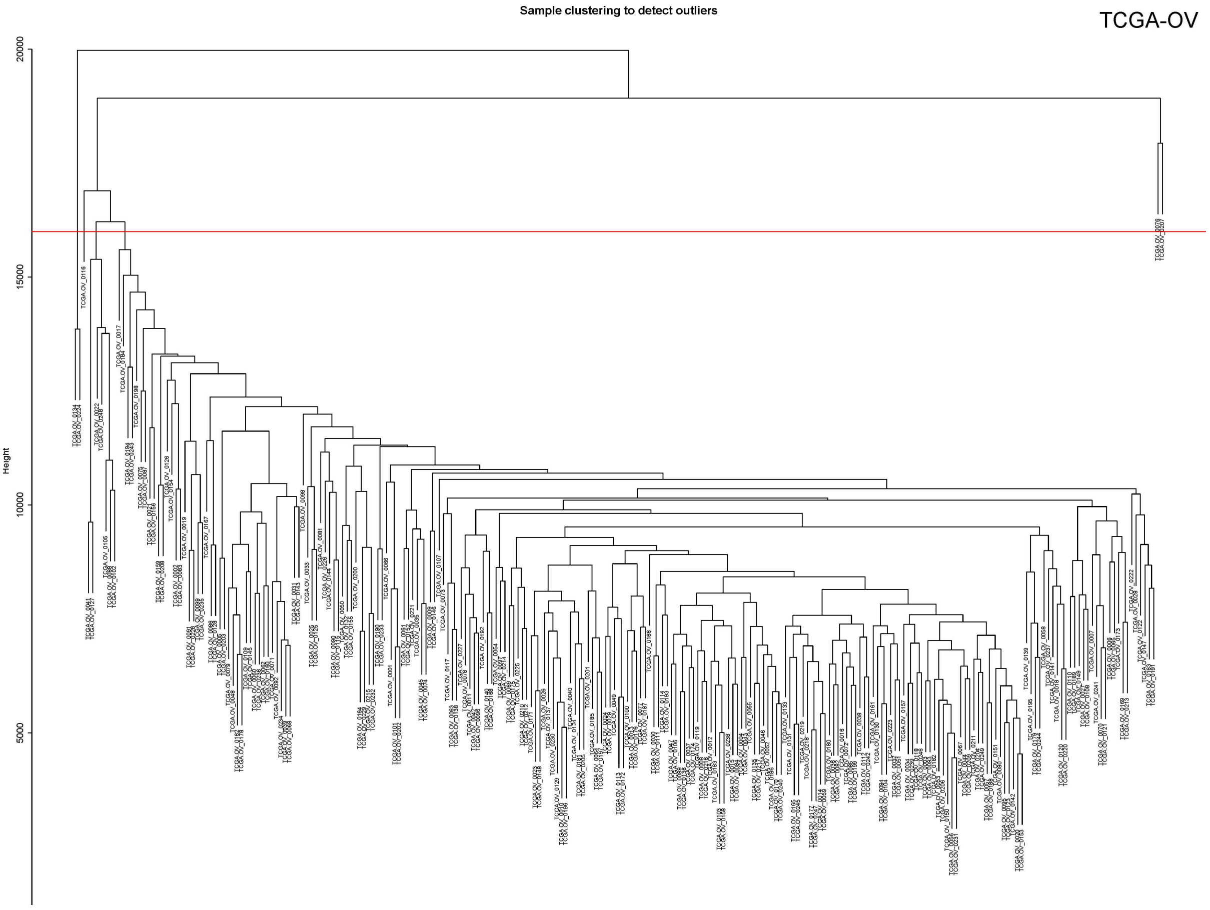

Supplementary Figure S2.20. Sample clustering to detect outliers.

Sample clustering is shown in a dendrogram. Arbitrary cut off value to exclude outlier is shown as red horizontal line. The name of TCGA dataset is located to the right top corner.

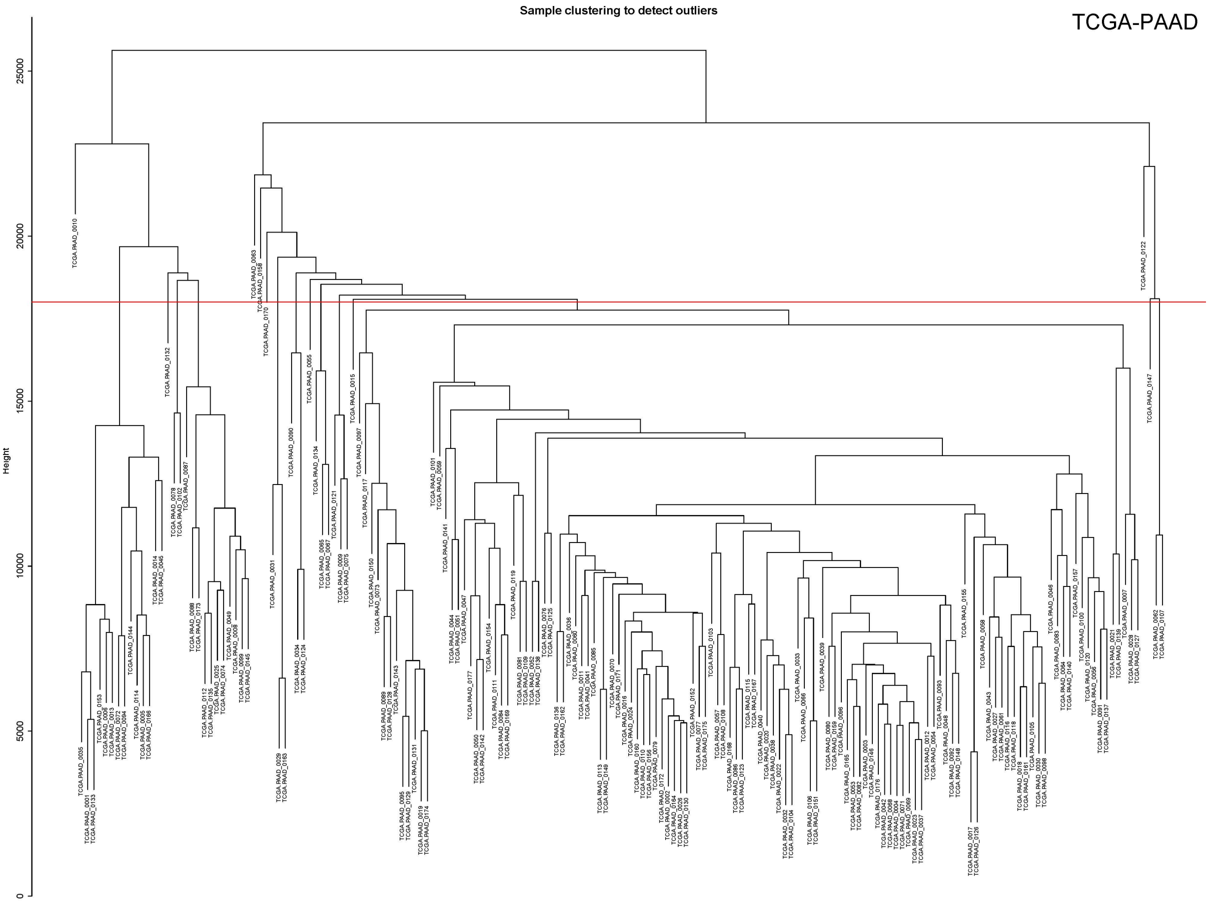

Supplementary Figure S2.21. Sample clustering to detect outliers.

Sample clustering is shown in a dendrogram. Arbitrary cut off value to exclude outlier is shown as red horizontal line. The name of TCGA dataset is located to the right top corner.

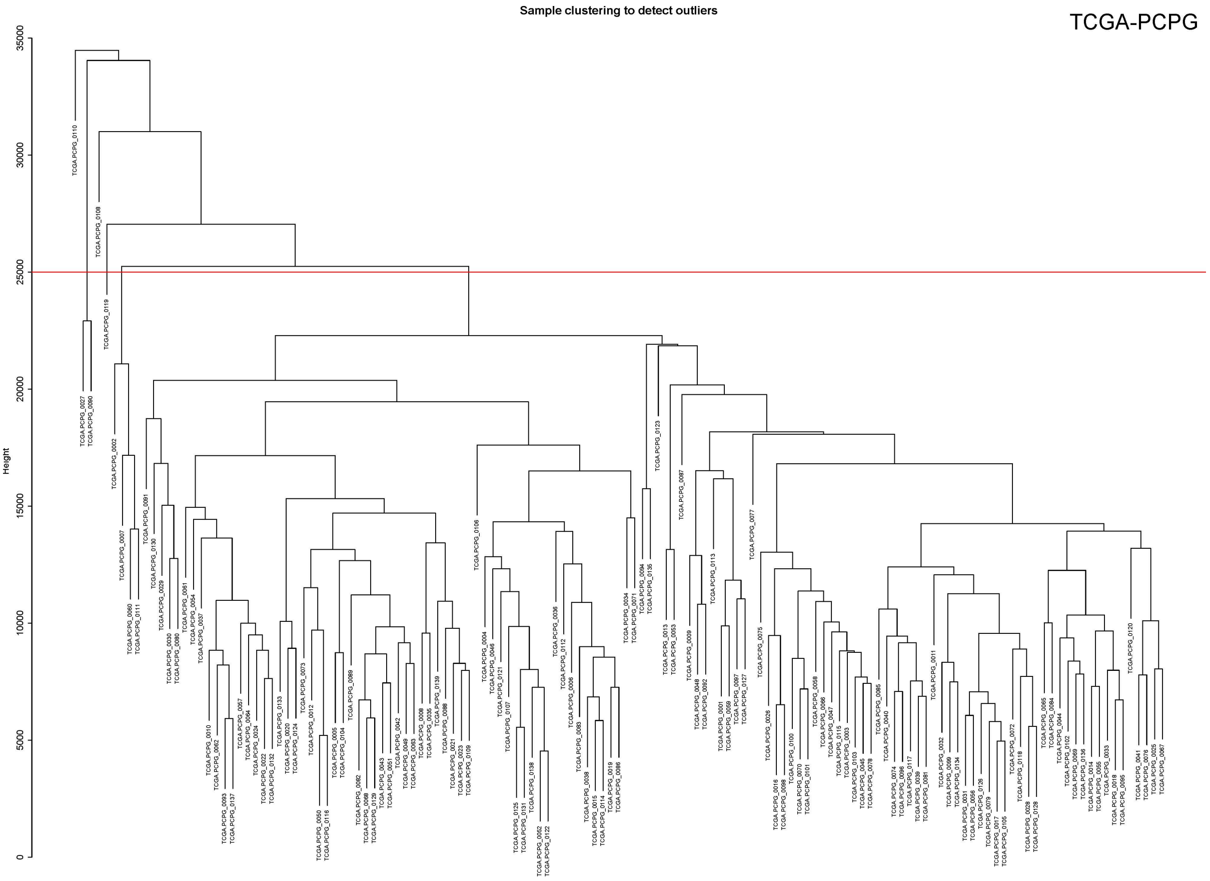

Supplementary Figure S2.22. Sample clustering to detect outliers.

Sample clustering is shown in a dendrogram. Arbitrary cut off value to exclude outlier is shown as red horizontal line. The name of TCGA dataset is located to the right top corner.

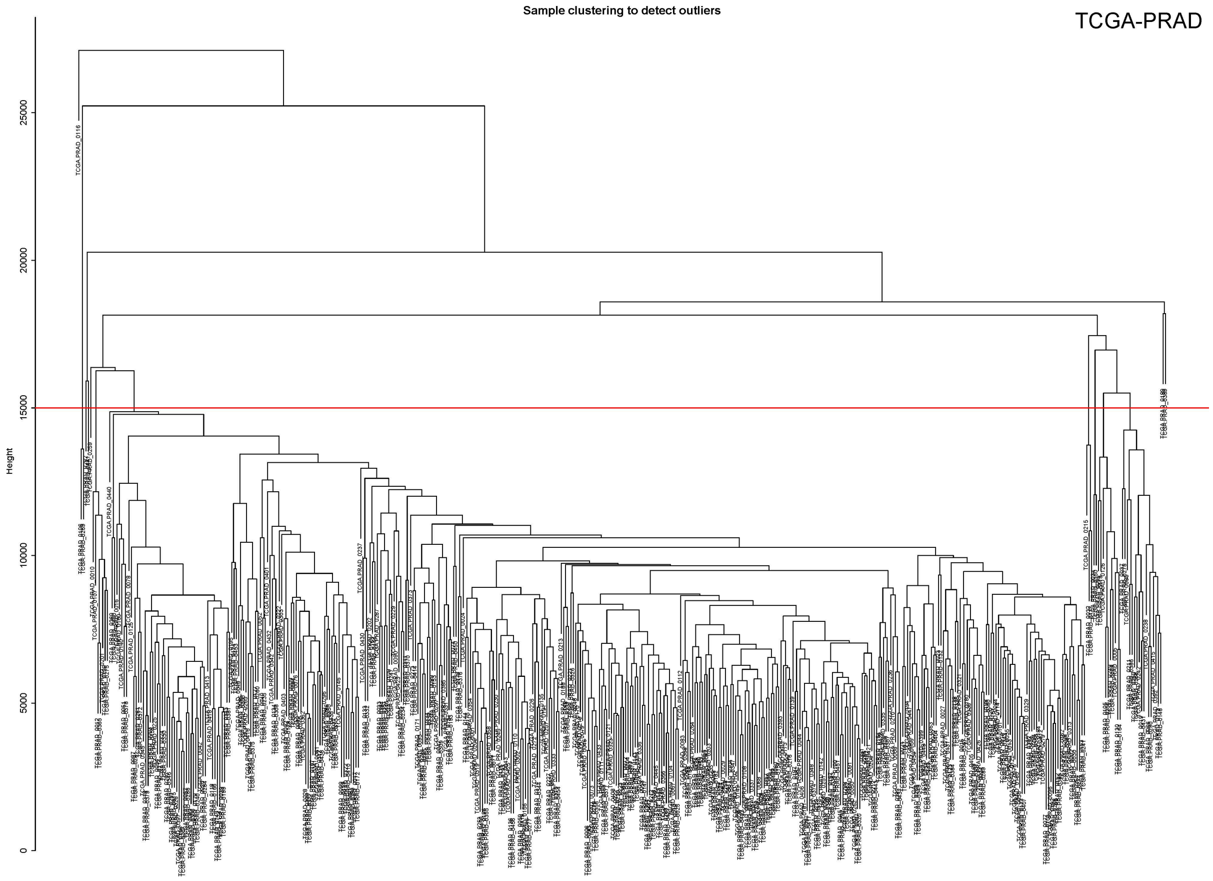

Supplementary Figure S2.23. Sample clustering to detect outliers.

Sample clustering is shown in a dendrogram. Arbitrary cut off value to exclude outlier is shown as red horizontal line. The name of TCGA dataset is located to the right top corner.

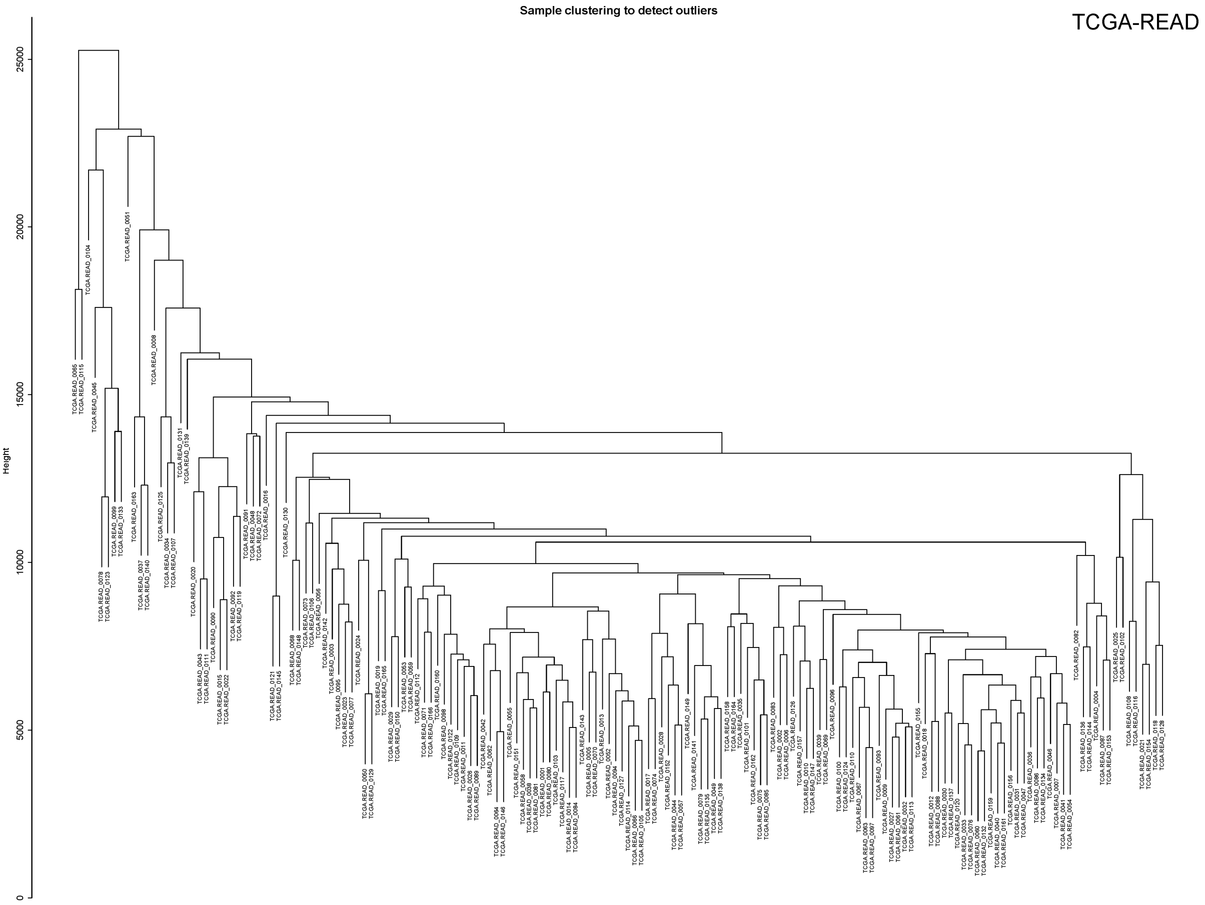

Supplementary Figure S2.24. Sample clustering to detect outliers.

Sample clustering is shown in a dendrogram. Arbitrary cut off value to exclude outlier is shown as red horizontal line. The name of TCGA dataset is located to the right top corner.

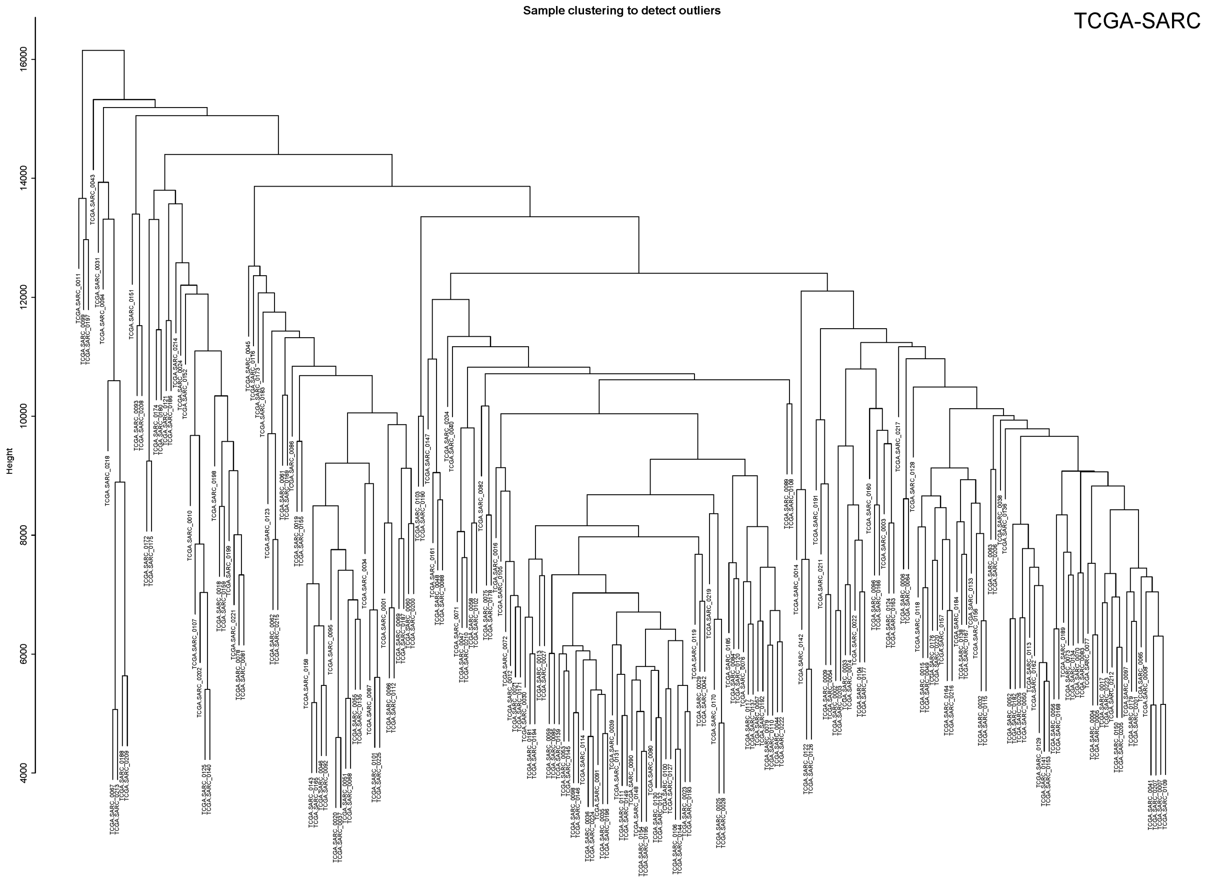

Supplementary Figure S2.25. Sample clustering to detect outliers.

Sample clustering is shown in a dendrogram. Arbitrary cut off value to exclude outlier is shown as red horizontal line. The name of TCGA dataset is located to the right top corner.

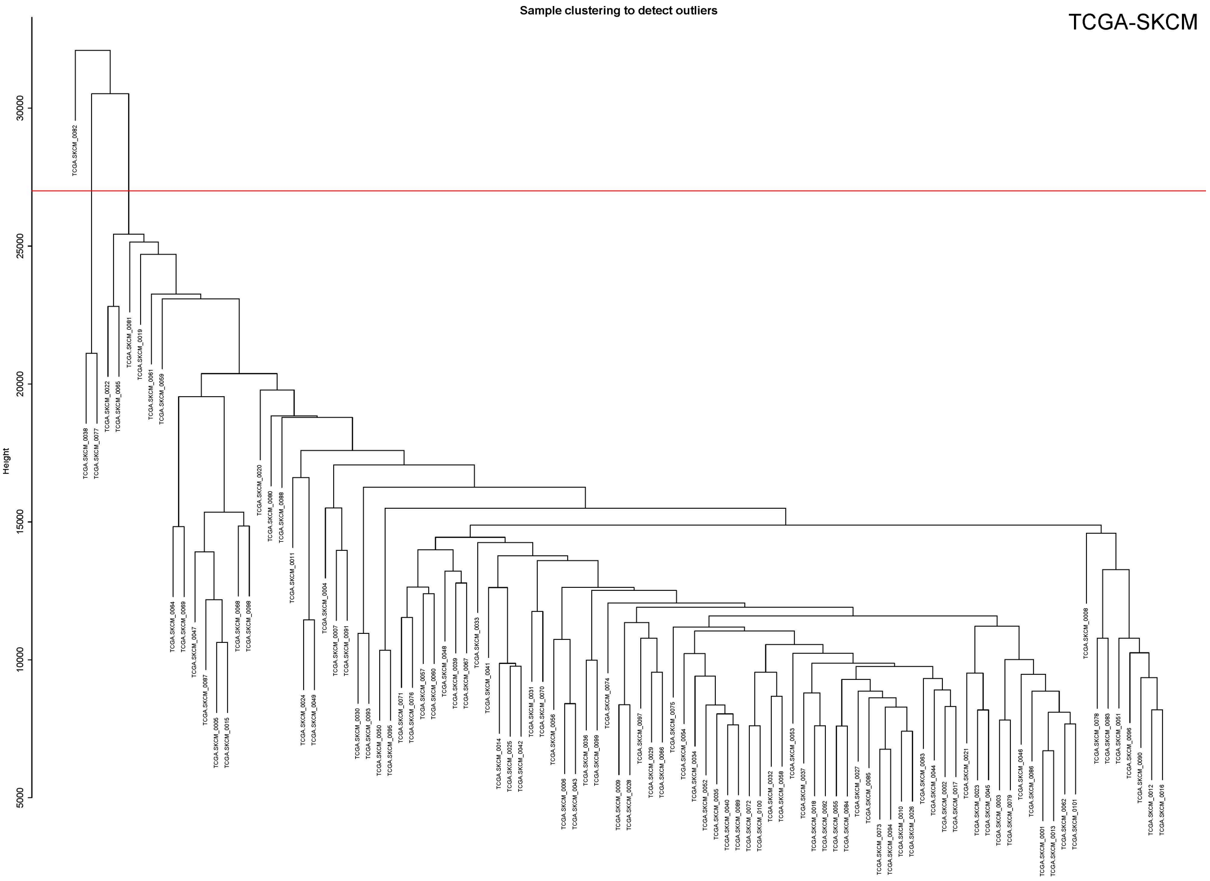

Supplementary Figure S2.26. Sample clustering to detect outliers.

Sample clustering is shown in a dendrogram. Arbitrary cut off value to exclude outlier is shown as red horizontal line. The name of TCGA dataset is located to the right top corner.

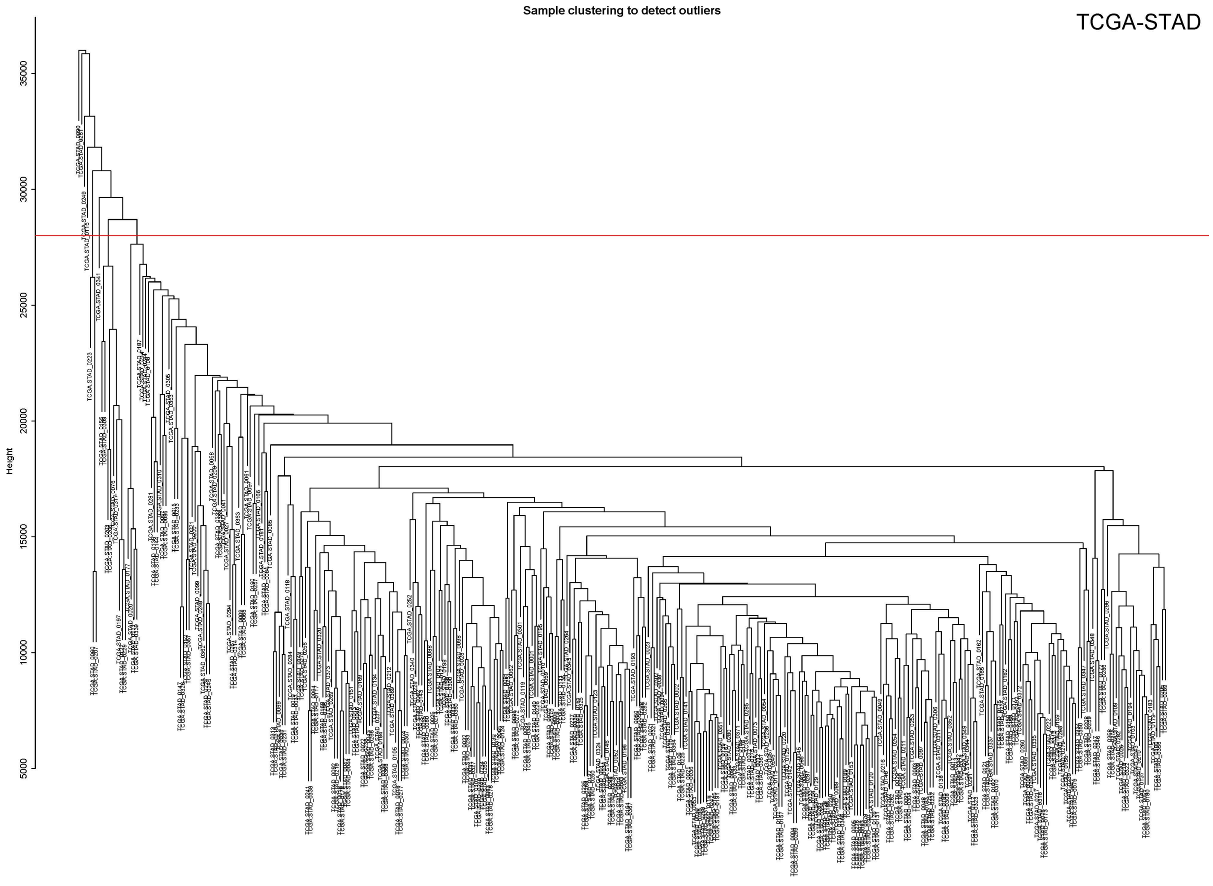

Supplementary Figure S2.27. Sample clustering to detect outliers.

Sample clustering is shown in a dendrogram. Arbitrary cut off value to exclude outlier is shown as red horizontal line. The name of TCGA dataset is located to the right top corner.

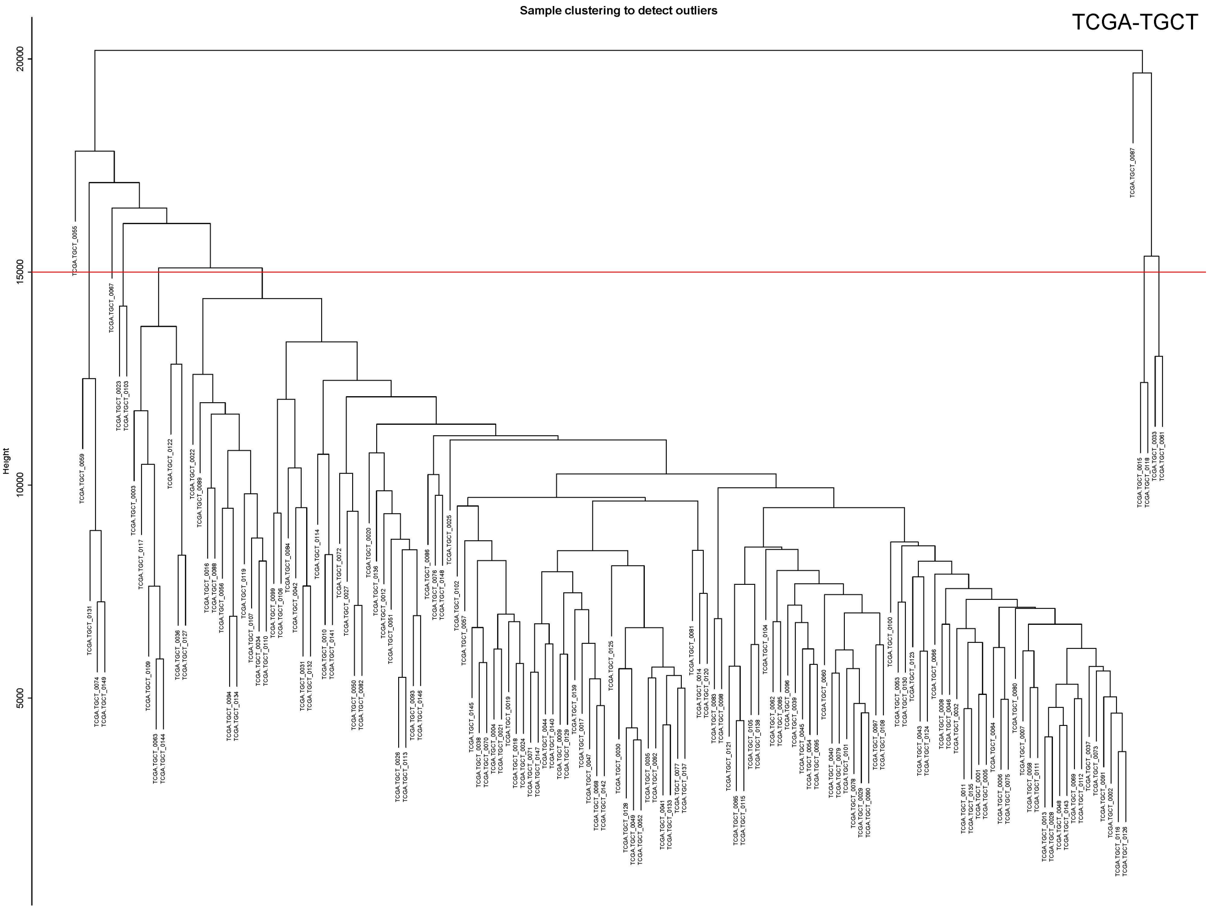

Supplementary Figure S2.28. Sample clustering to detect outliers.

Sample clustering is shown in a dendrogram. Arbitrary cut off value to exclude outlier is shown as red horizontal line. The name of TCGA dataset is located to the right top corner.

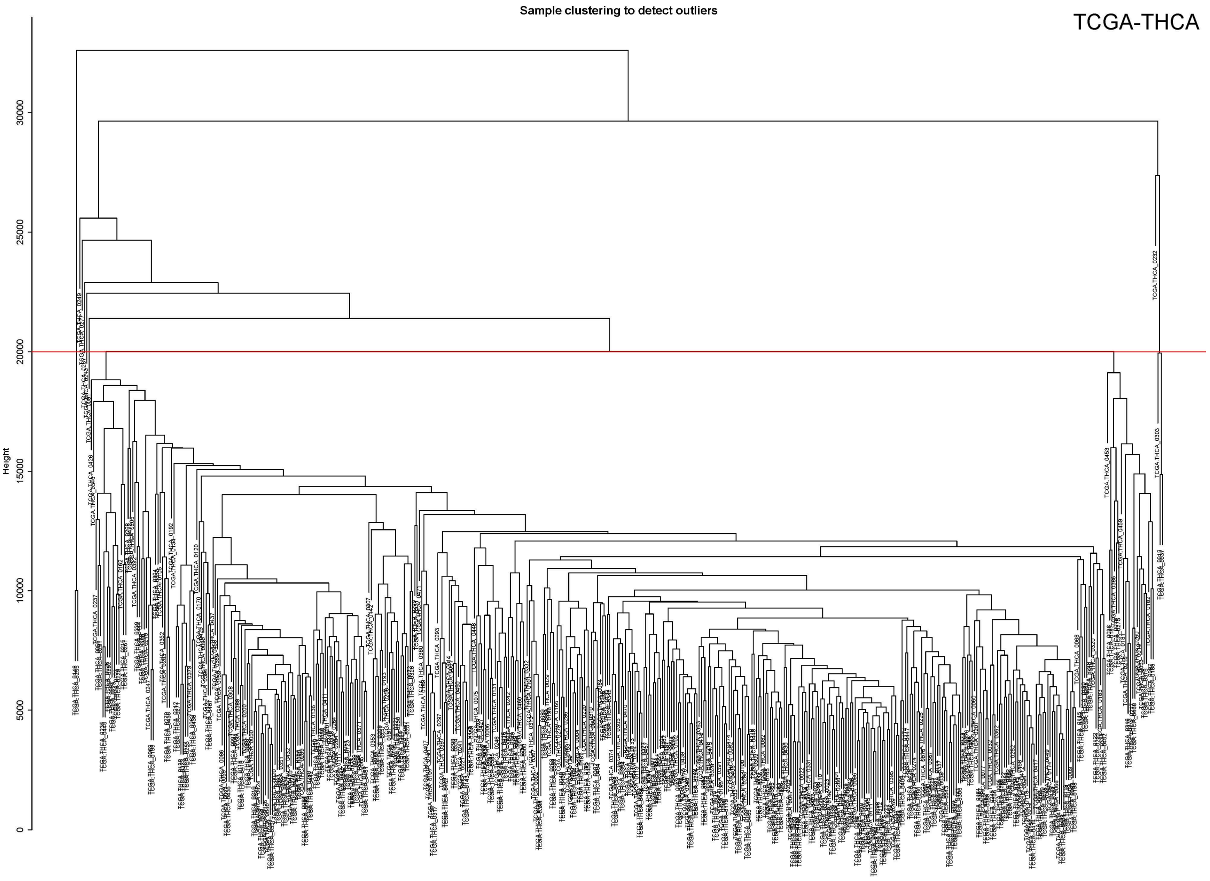

Supplementary Figure S2.29. Sample clustering to detect outliers.

Sample clustering is shown in a dendrogram. Arbitrary cut off value to exclude outlier is shown as red horizontal line. The name of TCGA dataset is located to the right top corner.

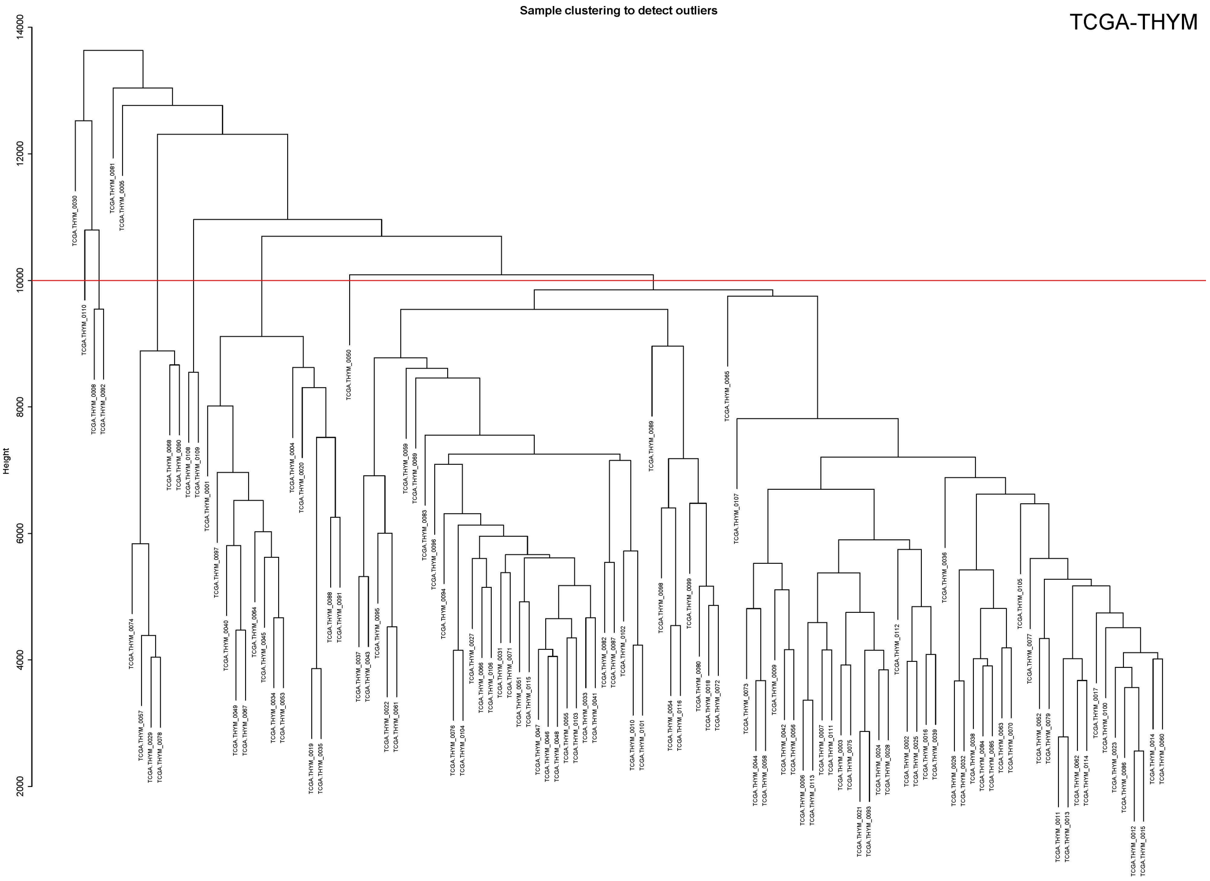

Supplementary Figure S2.30. Sample clustering to detect outliers.

Sample clustering is shown in a dendrogram. Arbitrary cut off value to exclude outlier is shown as red horizontal line. The name of TCGA dataset is located to the right top corner.

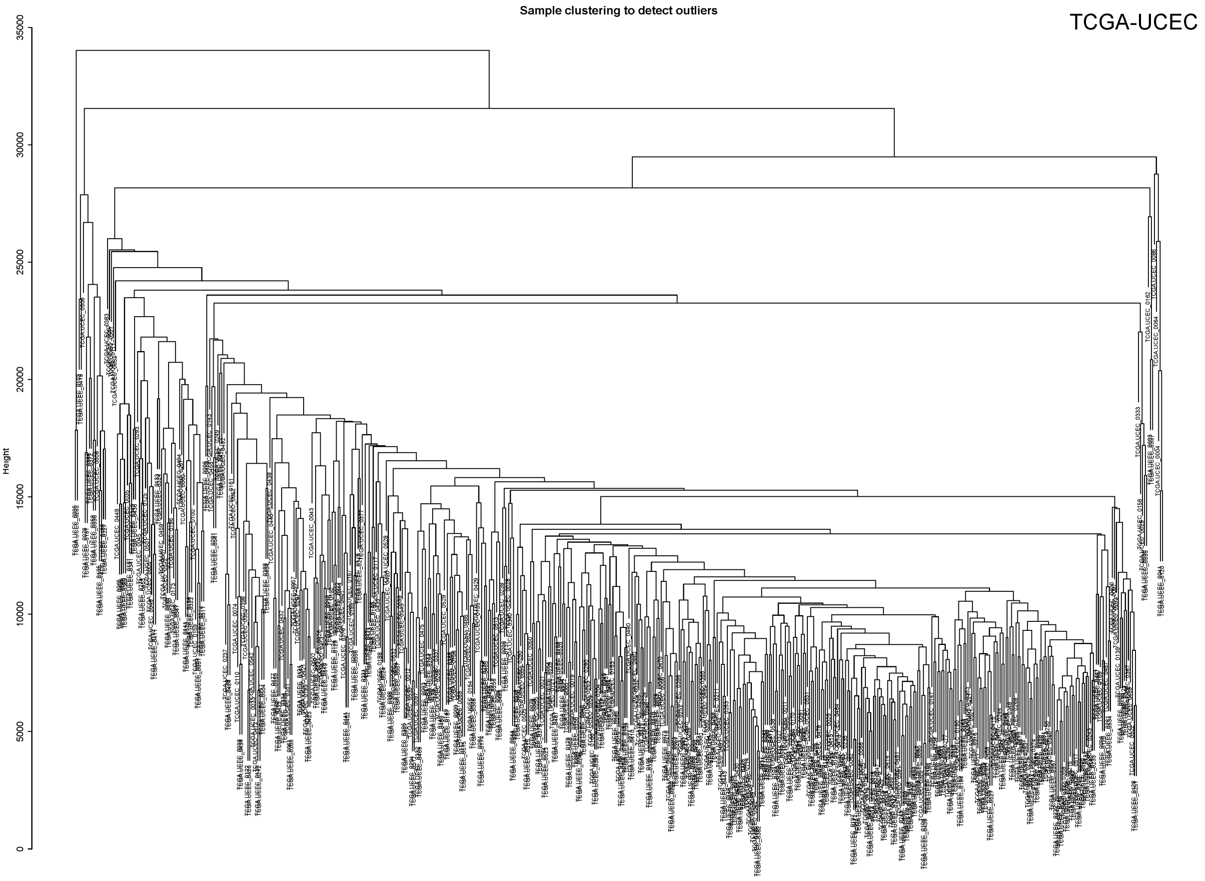

Supplementary Figure S2.31. Sample clustering to detect outliers.

Sample clustering is shown in a dendrogram. Arbitrary cut off value to exclude outlier is shown as red horizontal line. The name of TCGA dataset is located to the right top corner.

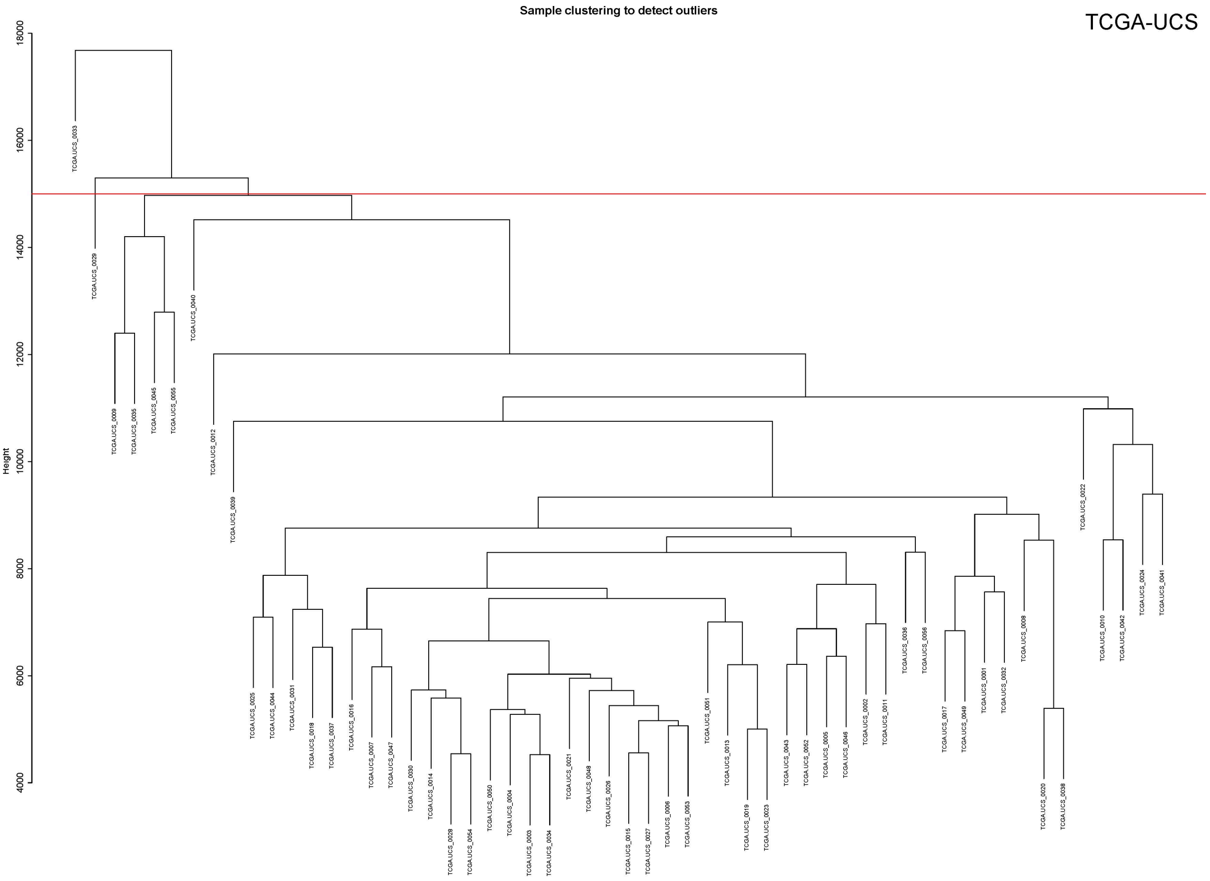

Supplementary Figure S2.32. Sample clustering to detect outliers.

Sample clustering is shown in a dendrogram. Arbitrary cut off value to exclude outlier is shown as red horizontal line. The name of TCGA dataset is located to the right top corner.

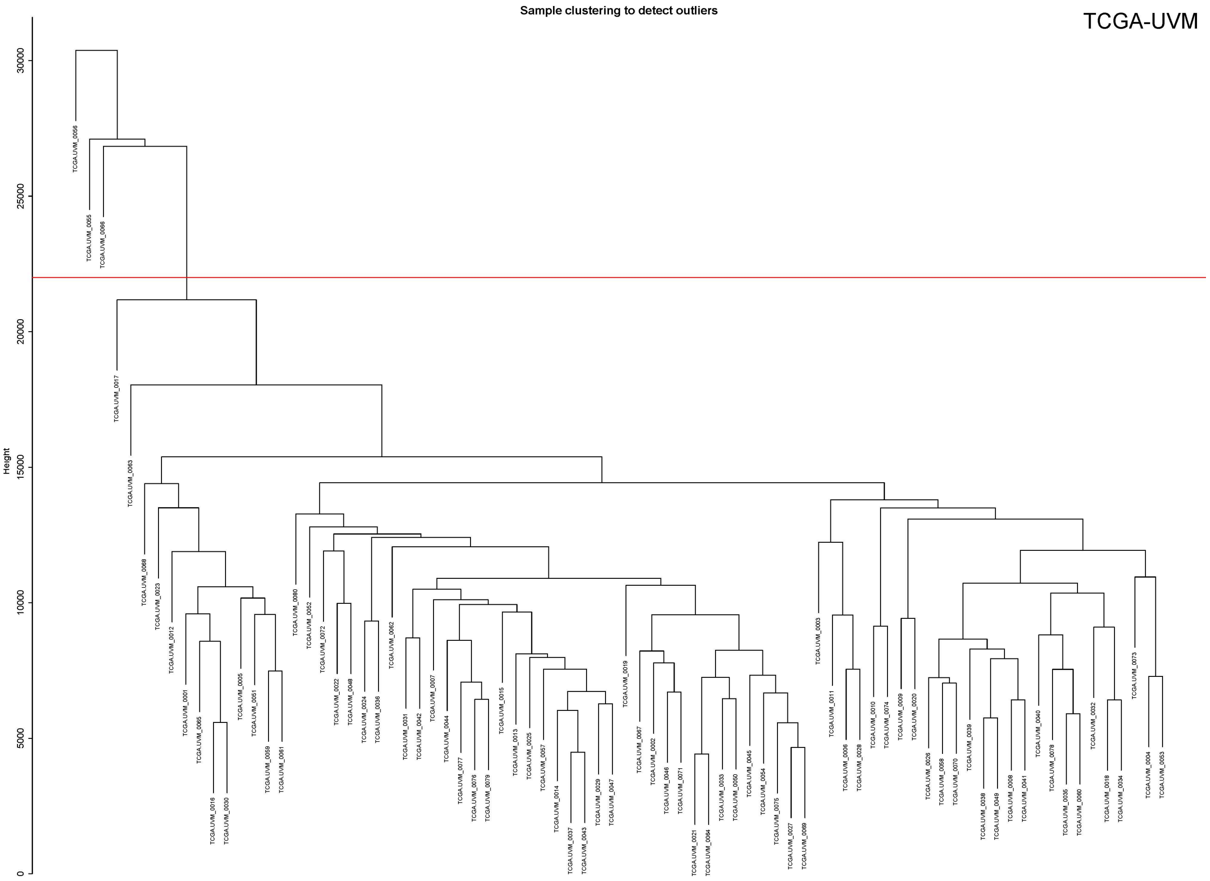

Supplementary Figure S2.33. Sample clustering to detect outliers.

Sample clustering is shown in a dendrogram. Arbitrary cut off value to exclude outlier is shown as red horizontal line. The name of TCGA dataset is located to the right top corner.

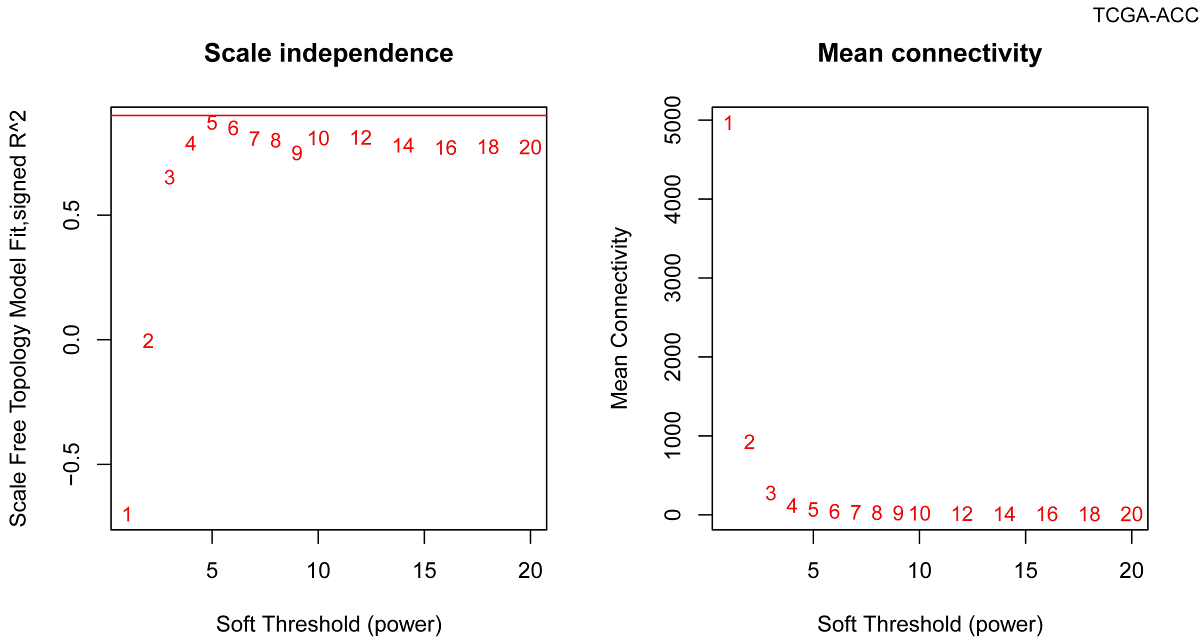

Supplementary Figure S3.1. Network topology analysis of soft-thresholding powers.

The scale-free fit (y-axis) against the soft-thresholding power (x-axis) is shown in the left panel, and the fit value (0.9) is shown as red horizontal lines. The mean connectivity (y-axis) against the soft-thresholding power (x-axis) is shown in the right panel. The name of TCGA dataset is located to the right top corner.

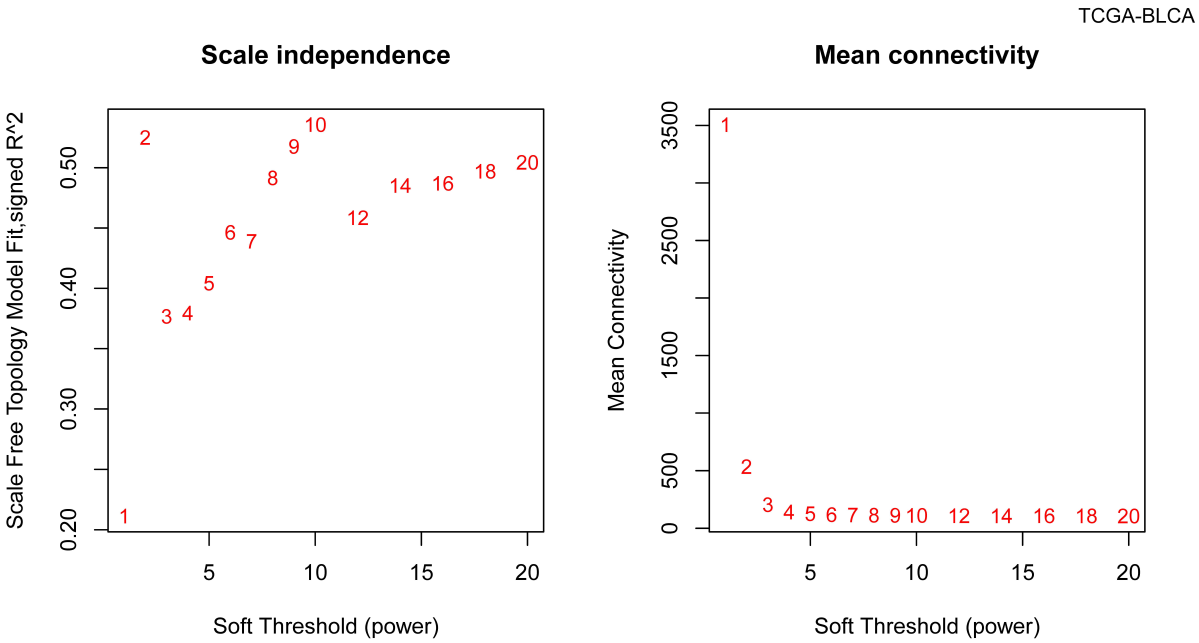

Supplementary Figure S3.2. Network topology analysis of soft-thresholding powers.

The scale-free fit (y-axis) against the soft-thresholding power (x-axis) is shown in the left panel, and the fit value (0.9) is shown as red horizontal lines. The mean connectivity (y-axis) against the soft-thresholding power (x-axis) is shown in the right panel. The name of TCGA dataset is located to the right top corner.

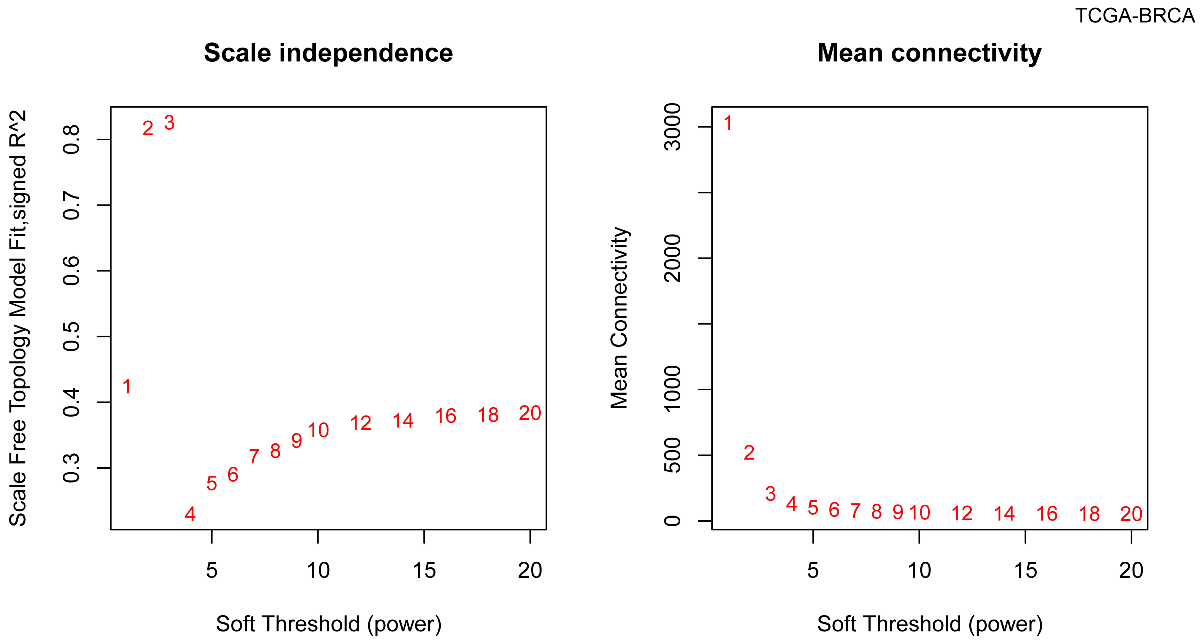

Supplementary Figure S3.3. Network topology analysis of soft-thresholding powers.

The scale-free fit (y-axis) against the soft-thresholding power (x-axis) is shown in the left panel, and the fit value (0.9) is shown as red horizontal lines. The mean connectivity (y-axis) against the soft-thresholding power (x-axis) is shown in the right panel. The name of TCGA dataset is located to the right top corner.

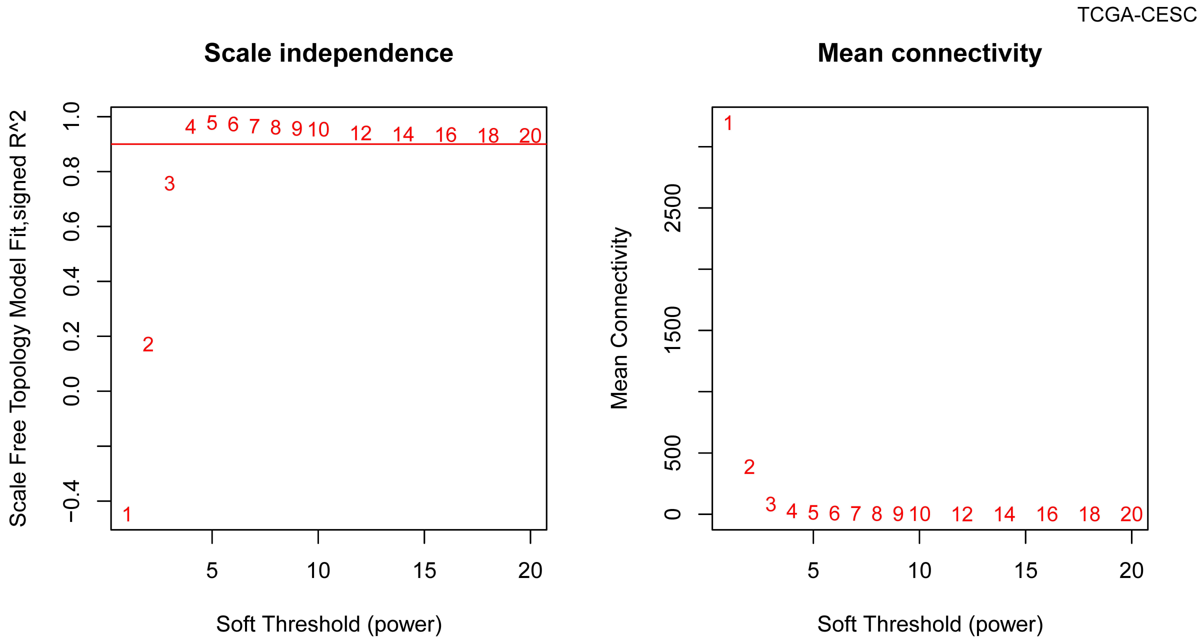

Supplementary Figure S3.4. Network topology analysis of soft-thresholding powers.

The scale-free fit (y-axis) against the soft-thresholding power (x-axis) is shown in the left panel, and the fit value (0.9) is shown as red horizontal lines. The mean connectivity (y-axis) against the soft-thresholding power (x-axis) is shown in the right panel. The name of TCGA dataset is located to the right top corner.

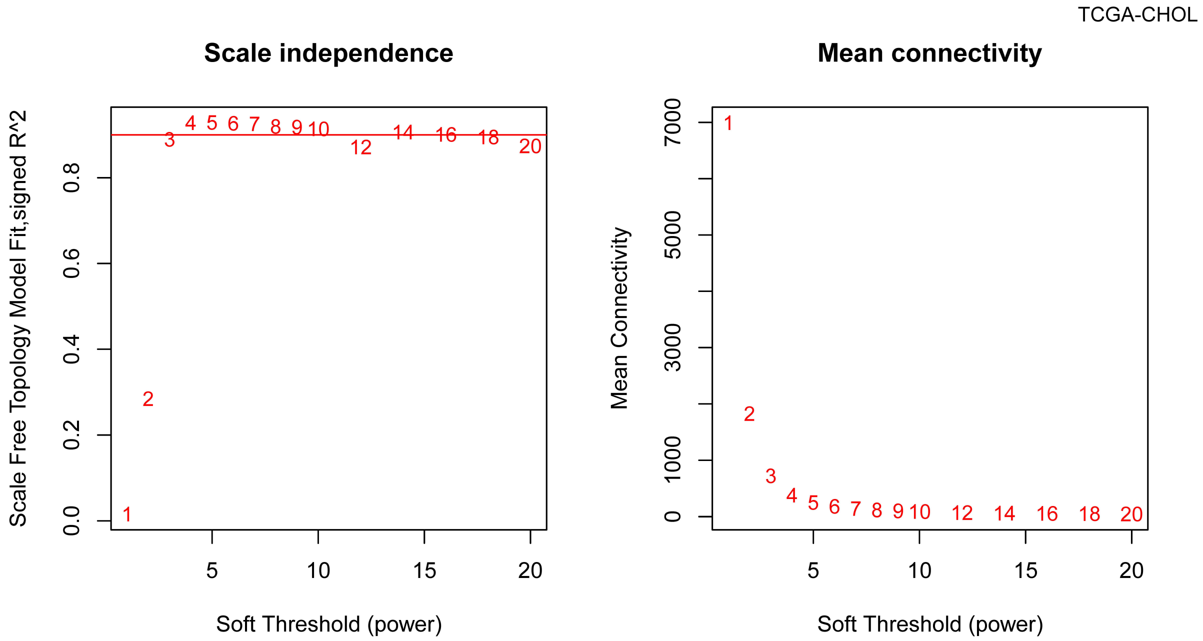

Supplementary Figure S3.5. Network topology analysis of soft-thresholding powers.

The scale-free fit (y-axis) against the soft-thresholding power (x-axis) is shown in the left panel, and the fit value (0.9) is shown as red horizontal lines. The mean connectivity (y-axis) against the soft-thresholding power (x-axis) is shown in the right panel. The name of TCGA dataset is located to the right top corner.

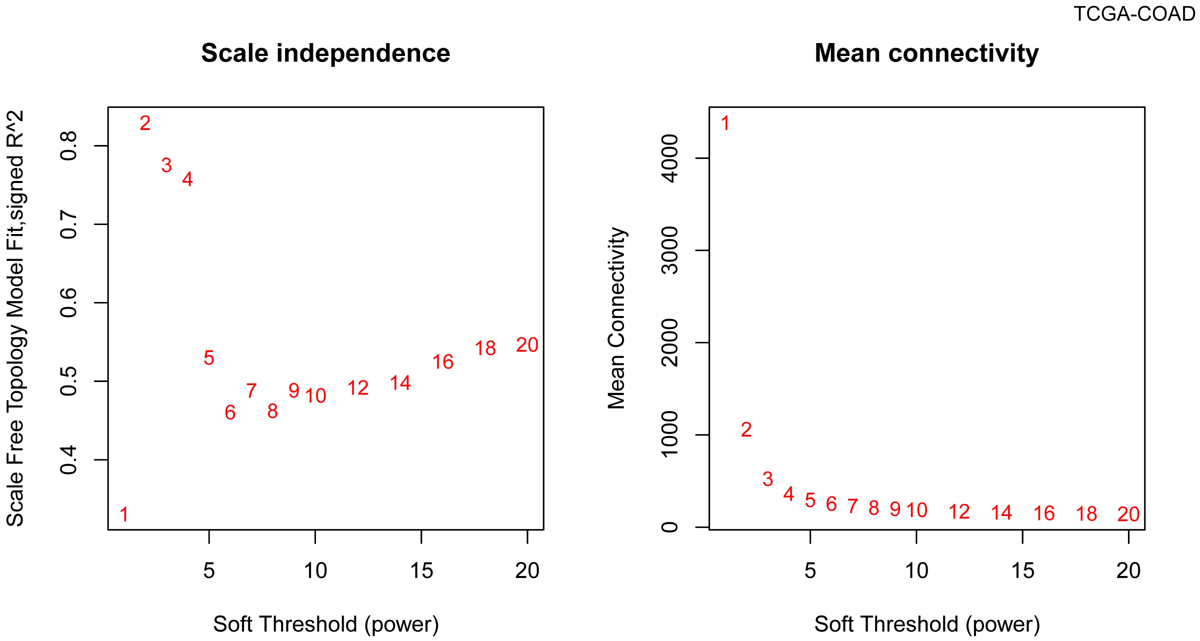

Supplementary Figure S3.6. Network topology analysis of soft-thresholding powers.

The scale-free fit (y-axis) against the soft-thresholding power (x-axis) is shown in the left panel, and the fit value (0.9) is shown as red horizontal lines. The mean connectivity (y-axis) against the soft-thresholding power (x-axis) is shown in the right panel. The name of TCGA dataset is located to the right top corner.

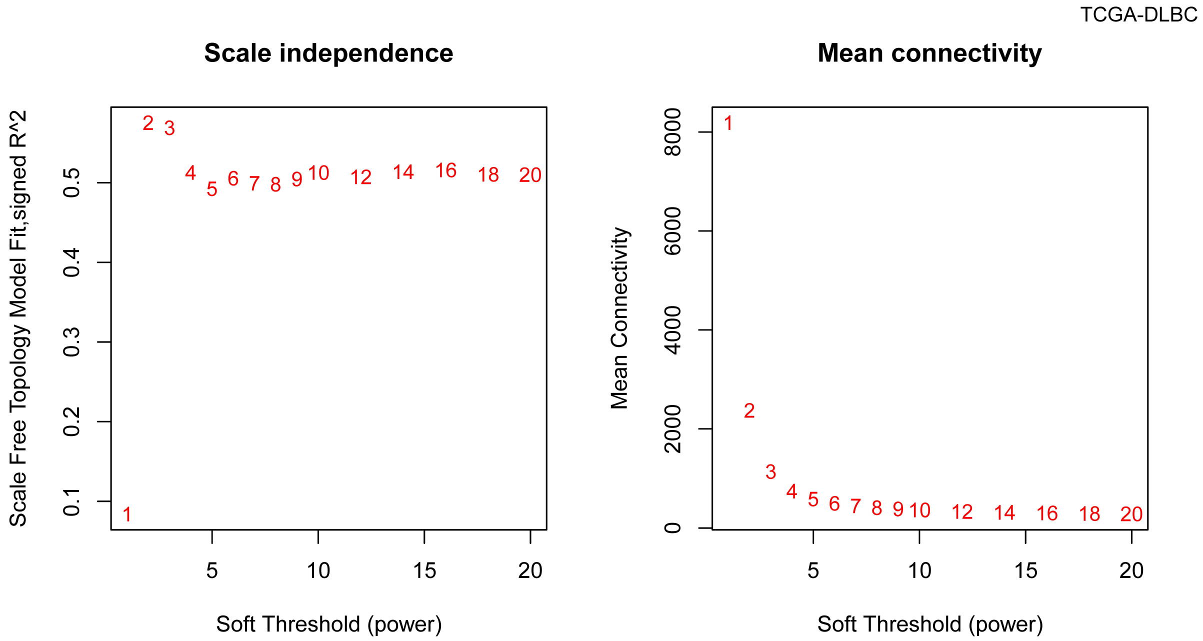

Supplementary Figure S3.7. Network topology analysis of soft-thresholding powers.

The scale-free fit (y-axis) against the soft-thresholding power (x-axis) is shown in the left panel, and the fit value (0.9) is shown as red horizontal lines. The mean connectivity (y-axis) against the soft-thresholding power (x-axis) is shown in the right panel. The name of TCGA dataset is located to the right top corner.

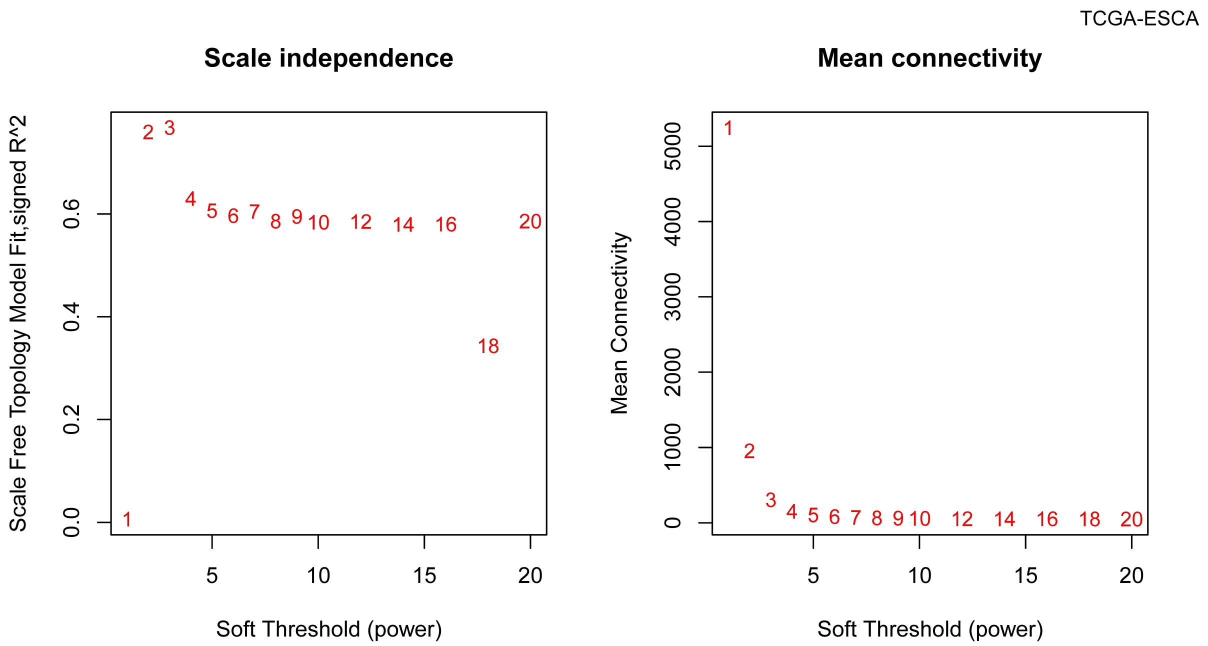

Supplementary Figure S3.8. Network topology analysis of soft-thresholding powers.

The scale-free fit (y-axis) against the soft-thresholding power (x-axis) is shown in the left panel, and the fit value (0.9) is shown as red horizontal lines. The mean connectivity (y-axis) against the soft-thresholding power (x-axis) is shown in the right panel. The name of TCGA dataset is located to the right top corner.

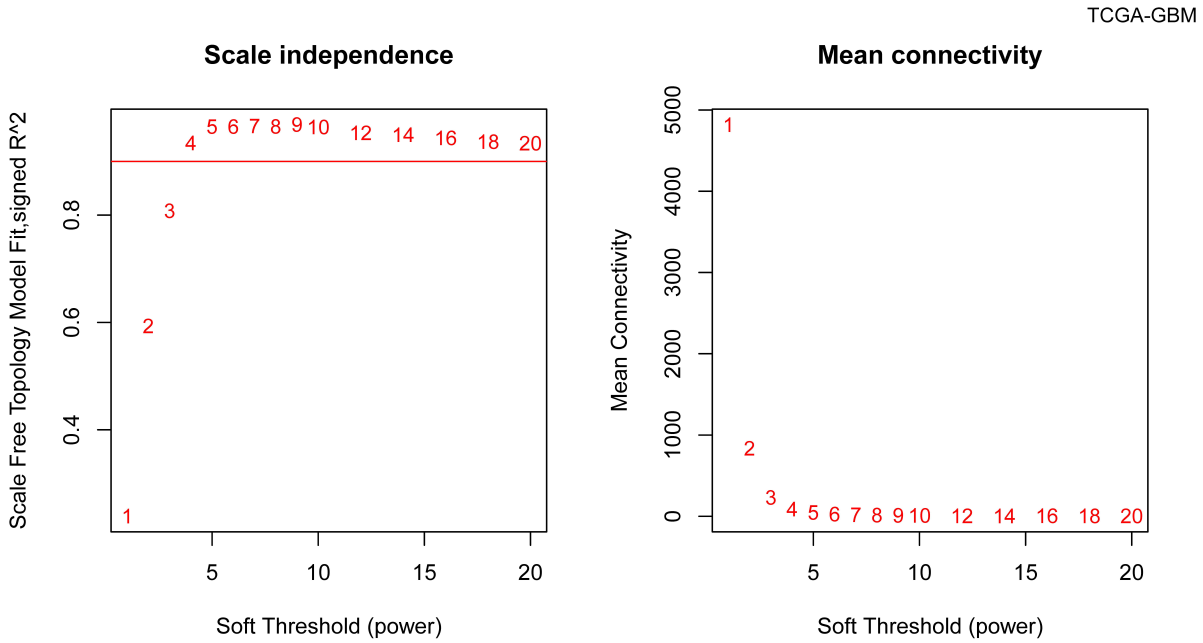

Supplementary Figure S3.9. Network topology analysis of soft-thresholding powers.

The scale-free fit (y-axis) against the soft-thresholding power (x-axis) is shown in the left panel, and the fit value (0.9) is shown as red horizontal lines. The mean connectivity (y-axis) against the soft-thresholding power (x-axis) is shown in the right panel. The name of TCGA dataset is located to the right top corner.

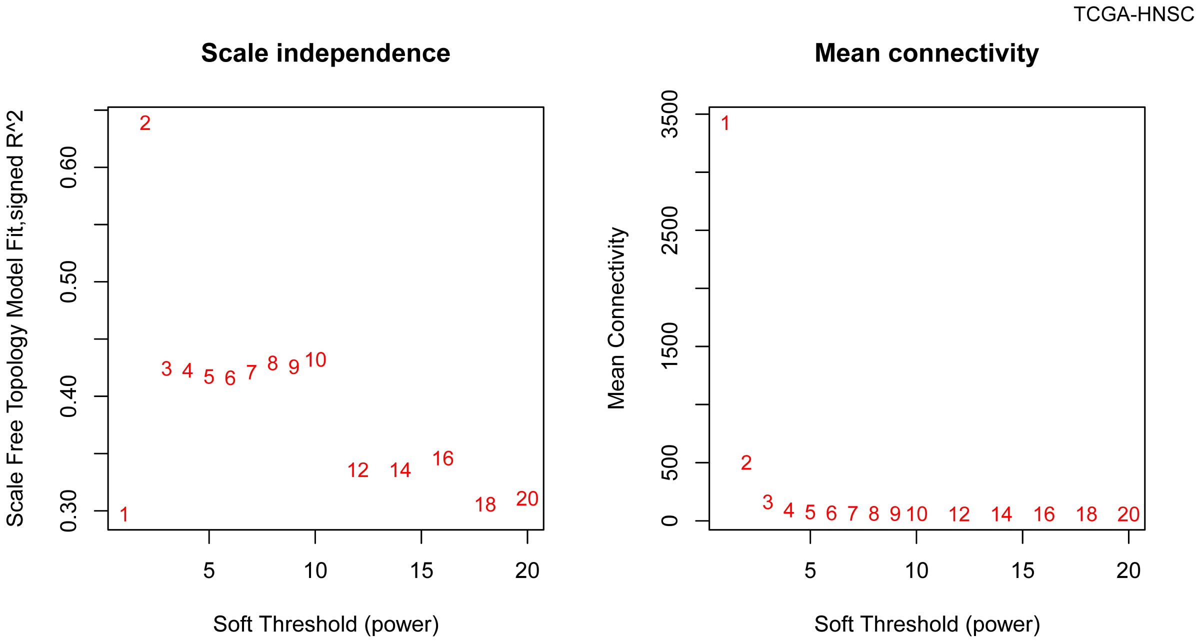

Supplementary Figure S3.10. Network topology analysis of soft-thresholding powers.

The scale-free fit (y-axis) against the soft-thresholding power (x-axis) is shown in the left panel, and the fit value (0.9) is shown as red horizontal lines. The mean connectivity (y-axis) against the soft-thresholding power (x-axis) is shown in the right panel. The name of TCGA dataset is located to the right top corner.

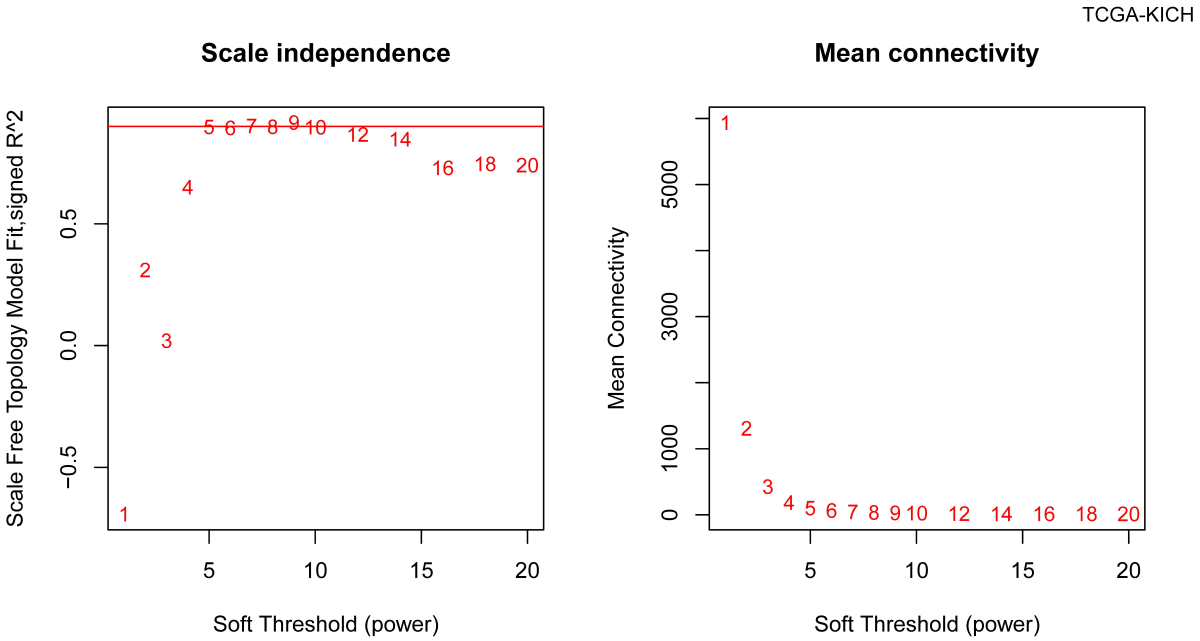

Supplementary Figure S3.11. Network topology analysis of soft-thresholding powers.

The scale-free fit (y-axis) against the soft-thresholding power (x-axis) is shown in the left panel, and the fit value (0.9) is shown as red horizontal lines. The mean connectivity (y-axis) against the soft-thresholding power (x-axis) is shown in the right panel. The name of TCGA dataset is located to the right top corner.

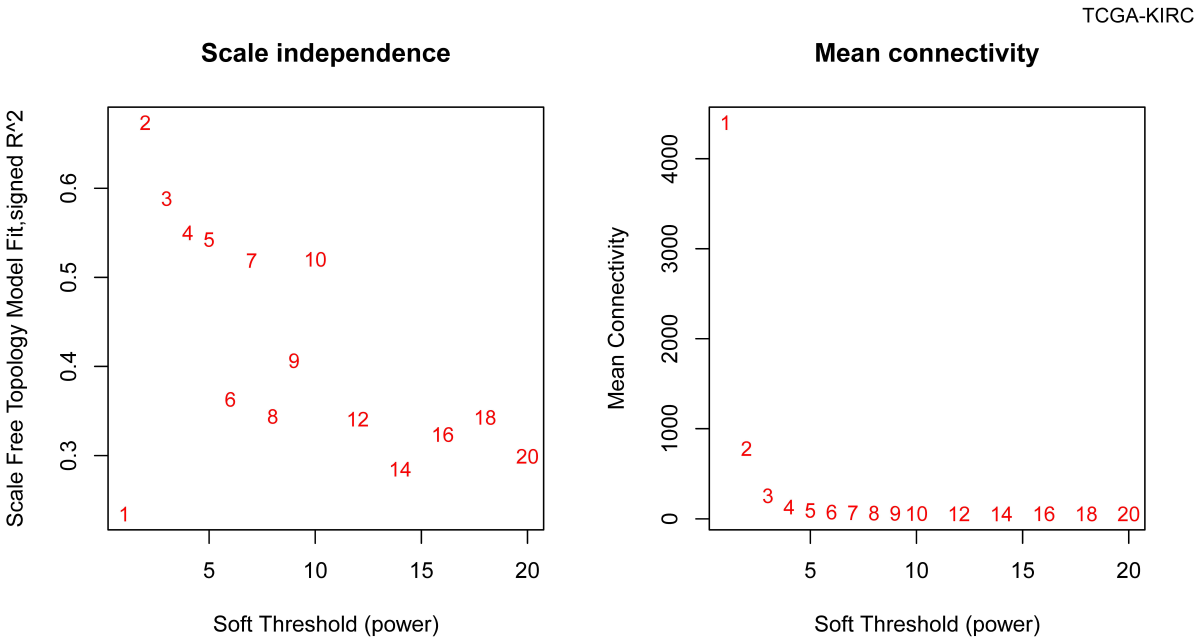

Supplementary Figure S3.12. Network topology analysis of soft-thresholding powers.

The scale-free fit (y-axis) against the soft-thresholding power (x-axis) is shown in the left panel, and the fit value (0.9) is shown as red horizontal lines. The mean connectivity (y-axis) against the soft-thresholding power (x-axis) is shown in the right panel. The name of TCGA dataset is located to the right top corner.

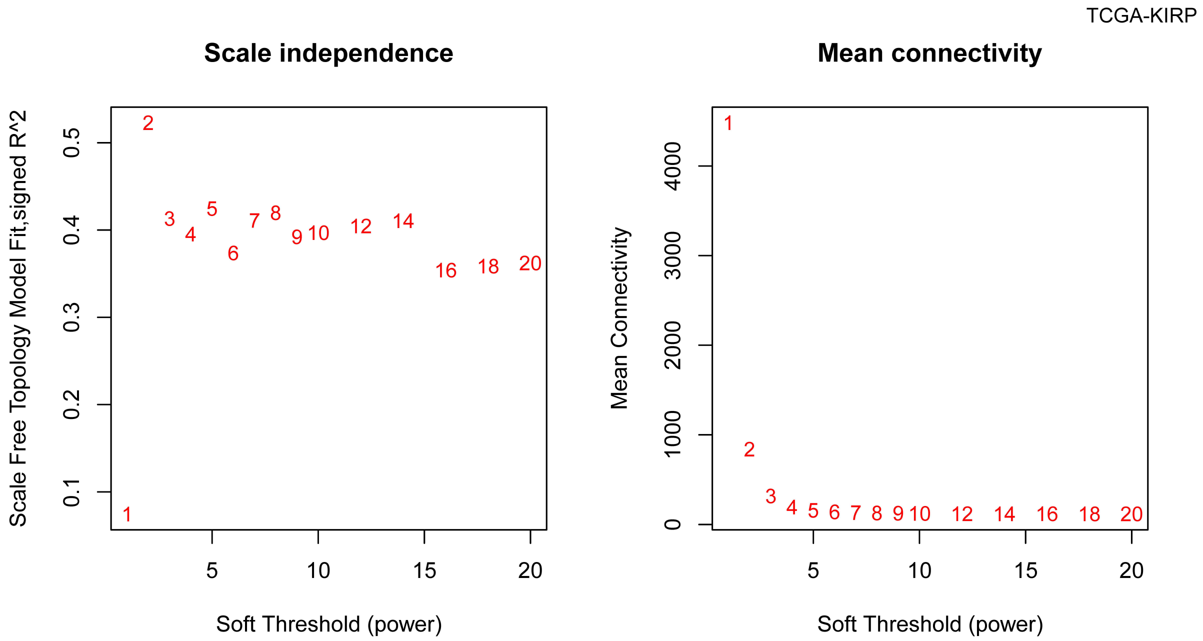

Supplementary Figure S3.13. Network topology analysis of soft-thresholding powers.

The scale-free fit (y-axis) against the soft-thresholding power (x-axis) is shown in the left panel, and the fit value (0.9) is shown as red horizontal lines. The mean connectivity (y-axis) against the soft-thresholding power (x-axis) is shown in the right panel. The name of TCGA dataset is located to the right top corner.

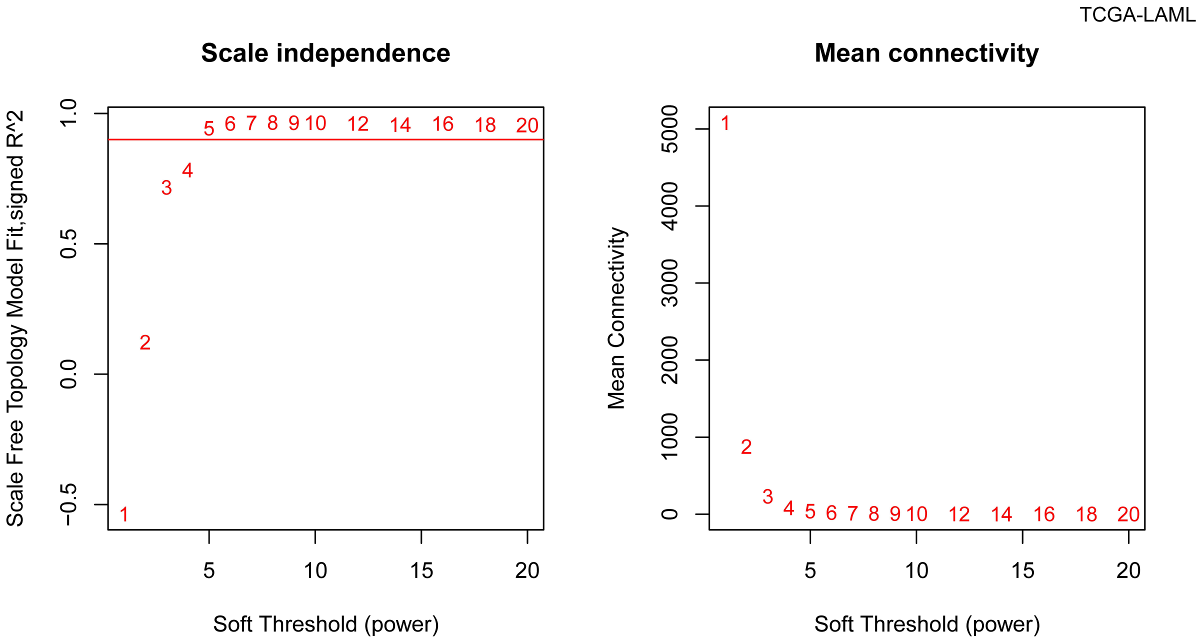

Supplementary Figure S3.14. Network topology analysis of soft-thresholding powers.

The scale-free fit (y-axis) against the soft-thresholding power (x-axis) is shown in the left panel, and the fit value (0.9) is shown as red horizontal lines. The mean connectivity (y-axis) against the soft-thresholding power (x-axis) is shown in the right panel. The name of TCGA dataset is located to the right top corner.

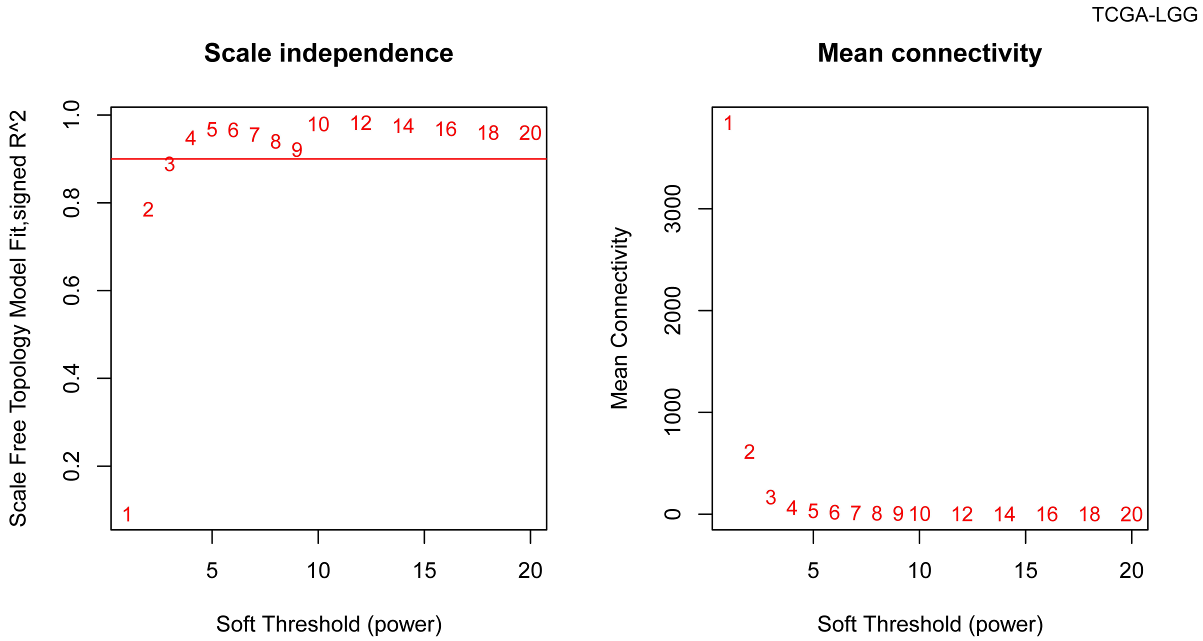

Supplementary Figure S3.15. Network topology analysis of soft-thresholding powers.

The scale-free fit (y-axis) against the soft-thresholding power (x-axis) is shown in the left panel, and the fit value (0.9) is shown as red horizontal lines. The mean connectivity (y-axis) against the soft-thresholding power (x-axis) is shown in the right panel. The name of TCGA dataset is located to the right top corner.

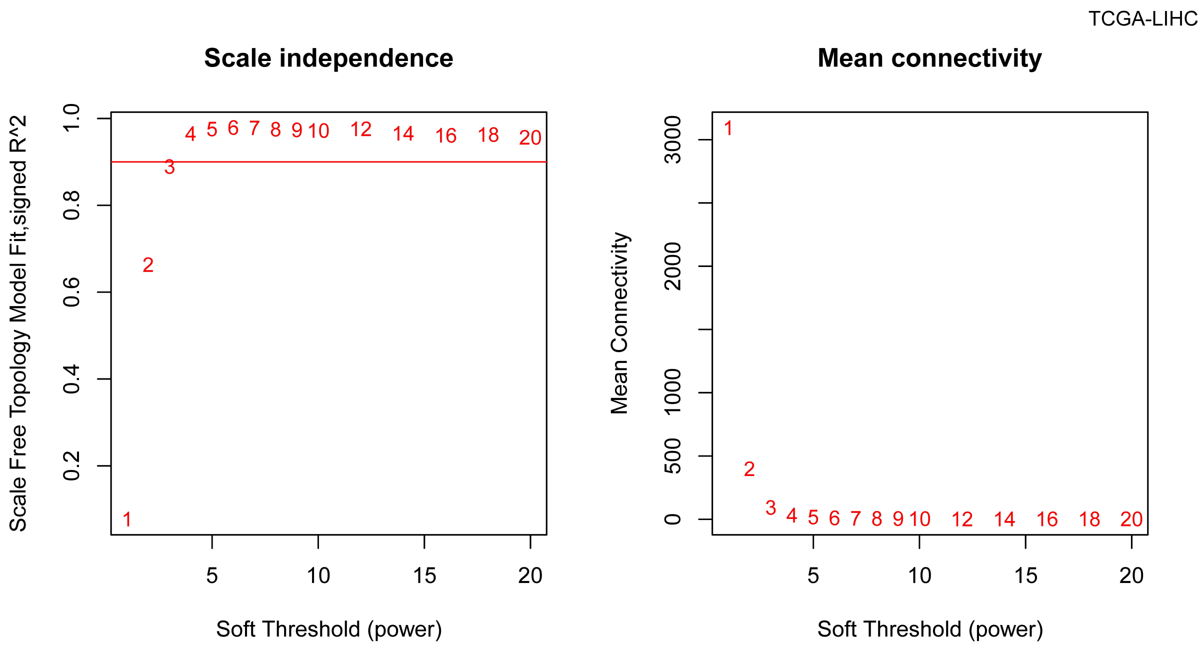

Supplementary Figure S3.16. Network topology analysis of soft-thresholding powers.

The scale-free fit (y-axis) against the soft-thresholding power (x-axis) is shown in the left panel, and the fit value (0.9) is shown as red horizontal lines. The mean connectivity (y-axis) against the soft-thresholding power (x-axis) is shown in the right panel. The name of TCGA dataset is located to the right top corner.

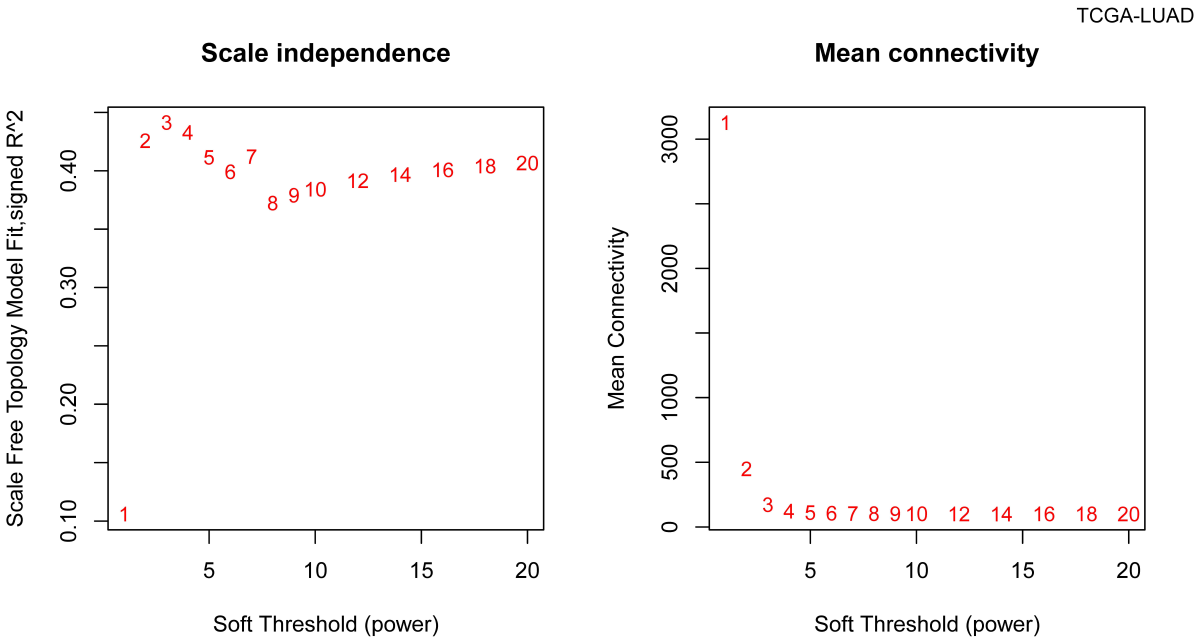

Supplementary Figure S3.17. Network topology analysis of soft-thresholding powers.

The scale-free fit (y-axis) against the soft-thresholding power (x-axis) is shown in the left panel, and the fit value (0.9) is shown as red horizontal lines. The mean connectivity (y-axis) against the soft-thresholding power (x-axis) is shown in the right panel. The name of TCGA dataset is located to the right top corner.

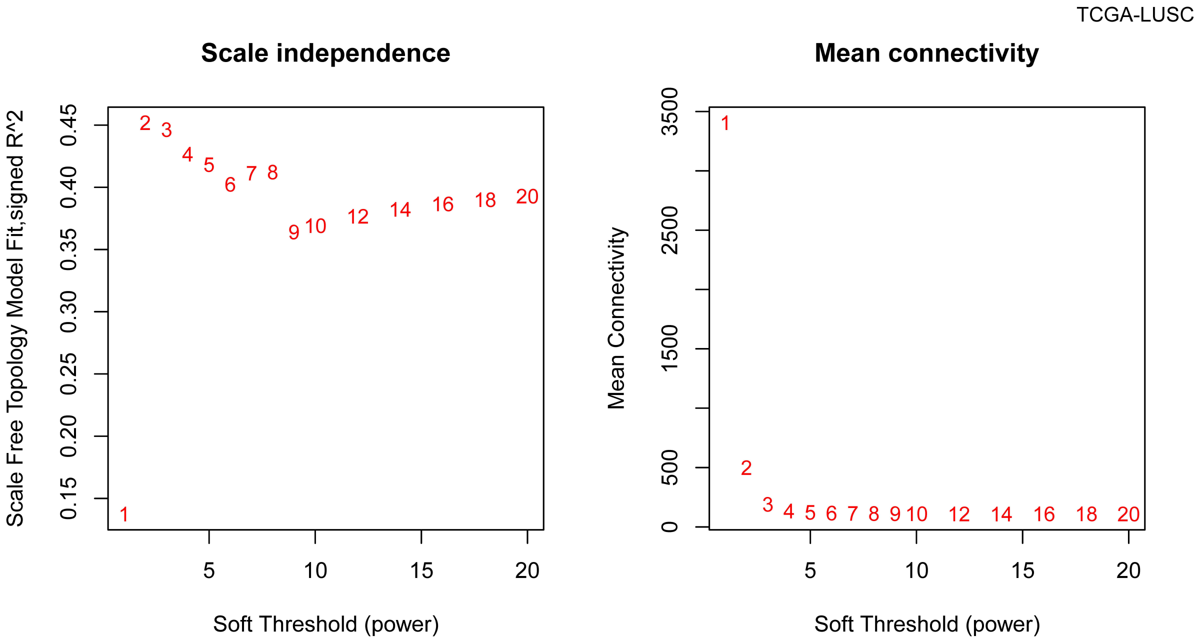

Supplementary Figure S3.18. Network topology analysis of soft-thresholding powers.

The scale-free fit (y-axis) against the soft-thresholding power (x-axis) is shown in the left panel, and the fit value (0.9) is shown as red horizontal lines. The mean connectivity (y-axis) against the soft-thresholding power (x-axis) is shown in the right panel. The name of TCGA dataset is located to the right top corner.

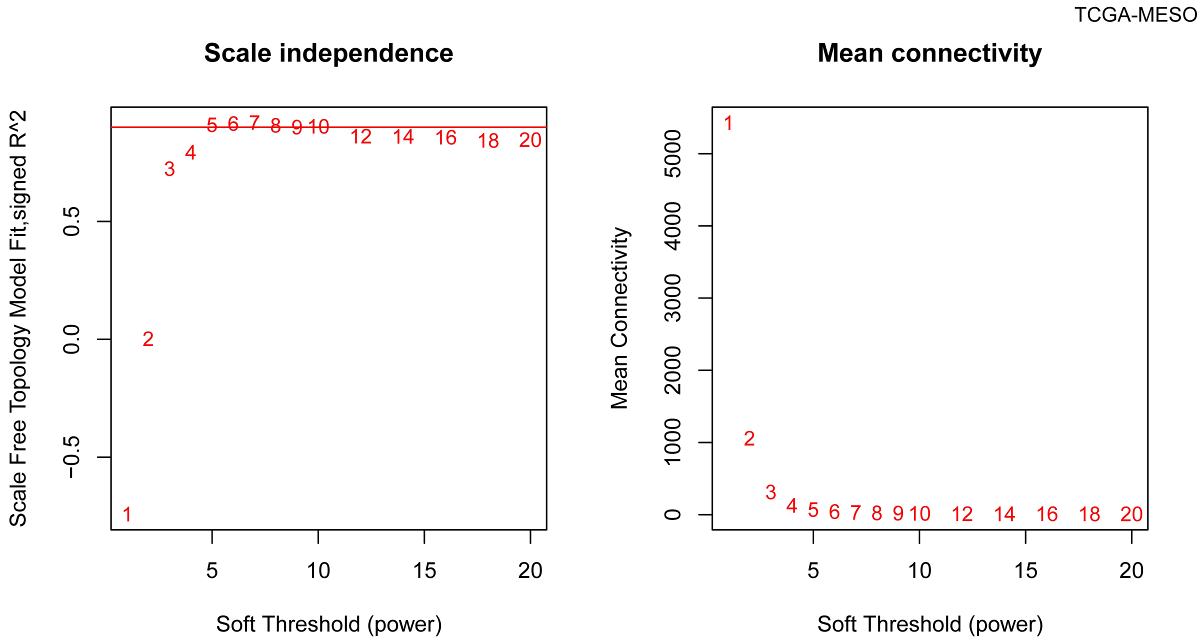

Supplementary Figure S3.19. Network topology analysis of soft-thresholding powers.

The scale-free fit (y-axis) against the soft-thresholding power (x-axis) is shown in the left panel, and the fit value (0.9) is shown as red horizontal lines. The mean connectivity (y-axis) against the soft-thresholding power (x-axis) is shown in the right panel. The name of TCGA dataset is located to the right top corner.

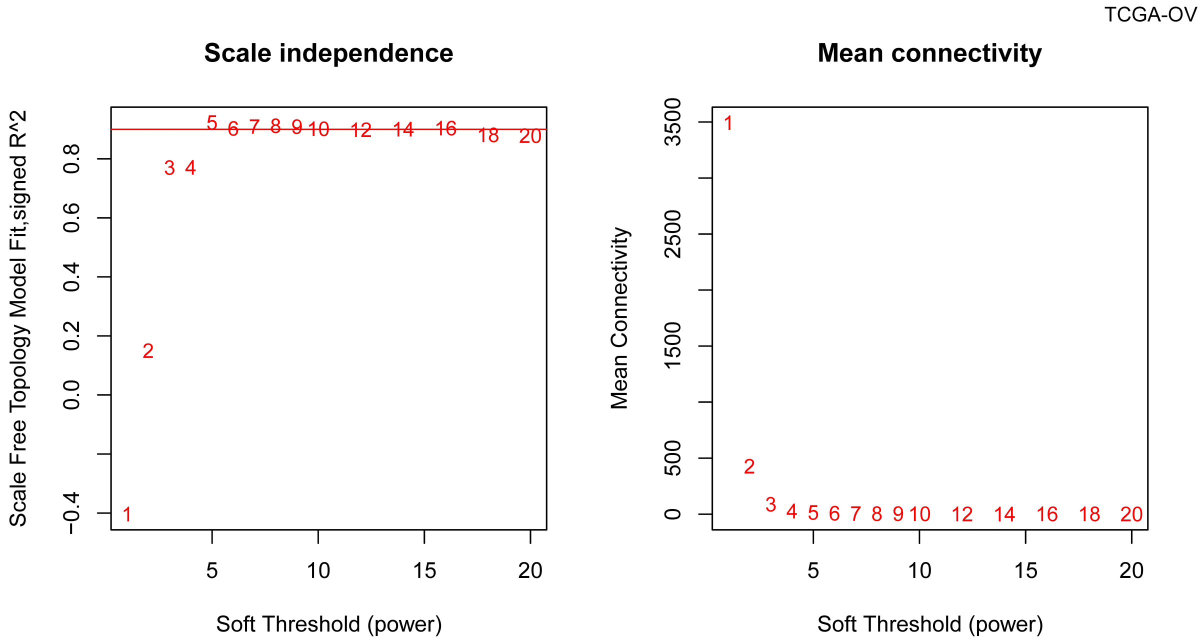

Supplementary Figure S3.20. Network topology analysis of soft-thresholding powers.

The scale-free fit (y-axis) against the soft-thresholding power (x-axis) is shown in the left panel, and the fit value (0.9) is shown as red horizontal lines. The mean connectivity (y-axis) against the soft-thresholding power (x-axis) is shown in the right panel. The name of TCGA dataset is located to the right top corner.

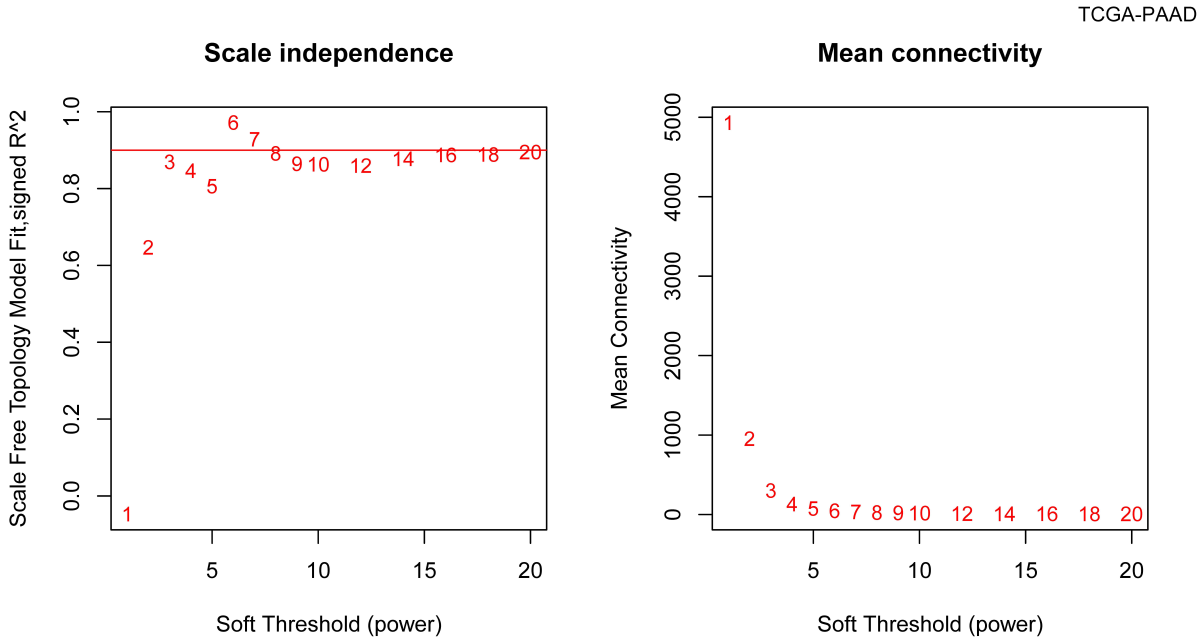

Supplementary Figure S3.21. Network topology analysis of soft-thresholding powers.

The scale-free fit (y-axis) against the soft-thresholding power (x-axis) is shown in the left panel, and the fit value (0.9) is shown as red horizontal lines. The mean connectivity (y-axis) against the soft-thresholding power (x-axis) is shown in the right panel. The name of TCGA dataset is located to the right top corner.

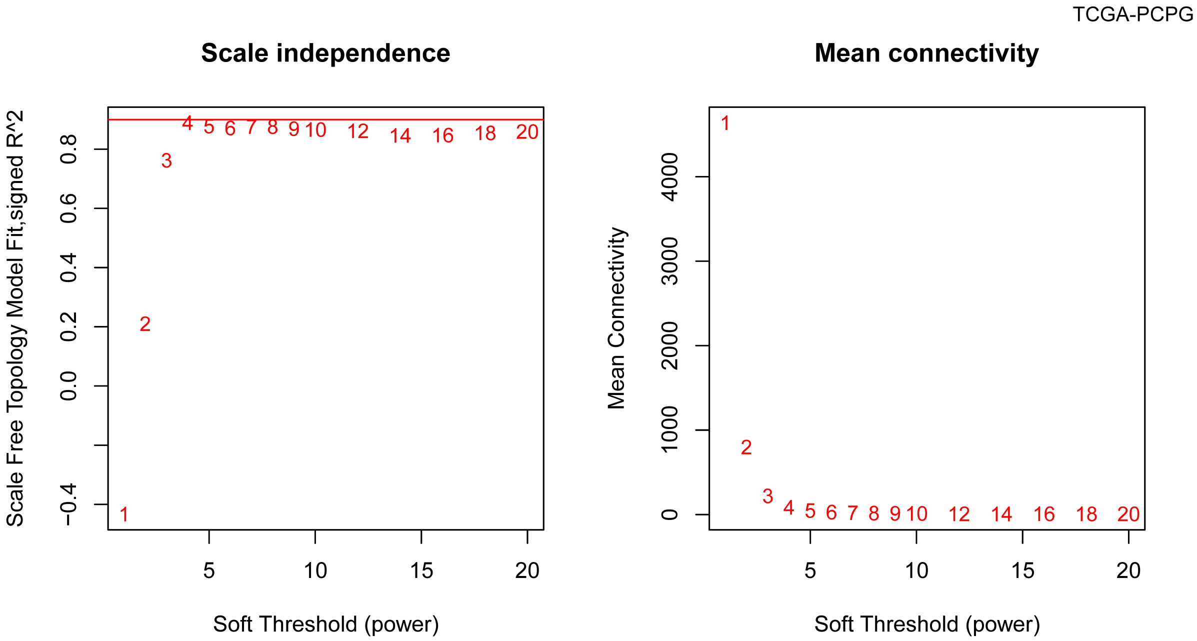

Supplementary Figure S3.22. Network topology analysis of soft-thresholding powers.

The scale-free fit (y-axis) against the soft-thresholding power (x-axis) is shown in the left panel, and the fit value (0.9) is shown as red horizontal lines. The mean connectivity (y-axis) against the soft-thresholding power (x-axis) is shown in the right panel. The name of TCGA dataset is located to the right top corner.

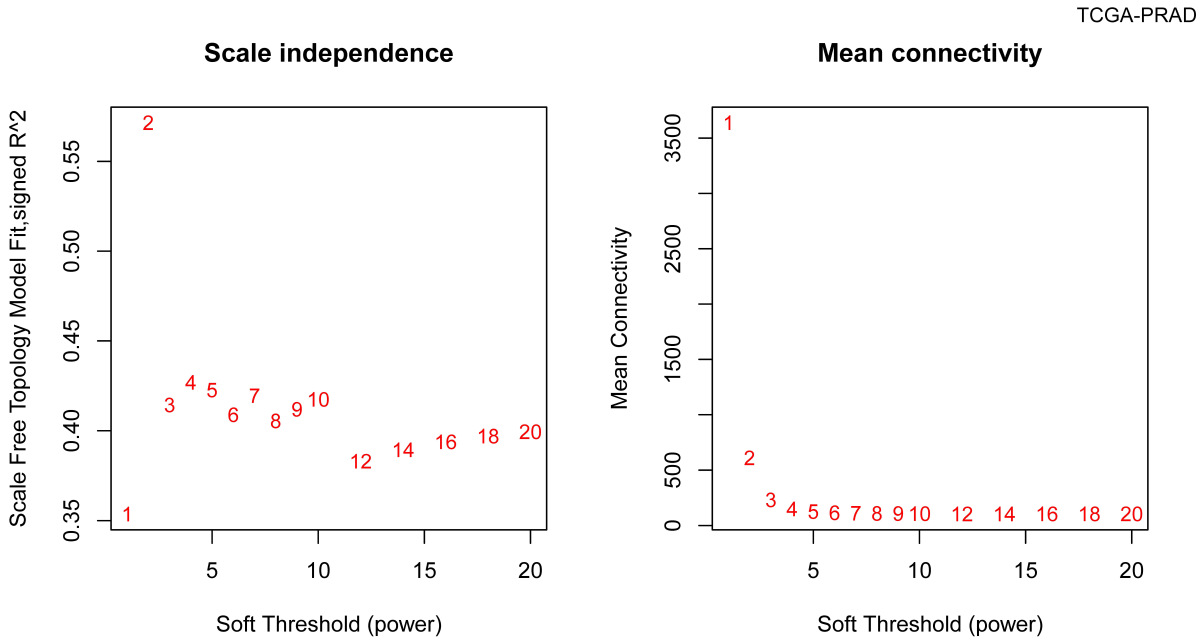

Supplementary Figure S3.23. Network topology analysis of soft-thresholding powers.

The scale-free fit (y-axis) against the soft-thresholding power (x-axis) is shown in the left panel, and the fit value (0.9) is shown as red horizontal lines. The mean connectivity (y-axis) against the soft-thresholding power (x-axis) is shown in the right panel. The name of TCGA dataset is located to the right top corner.

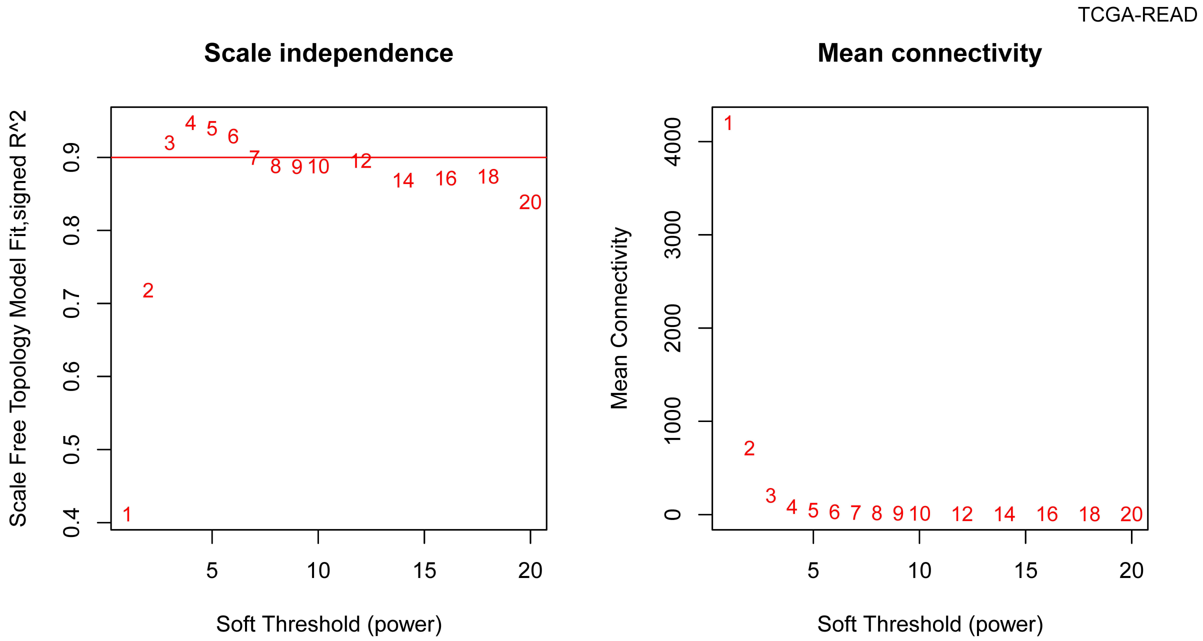

Supplementary Figure S3.24. Network topology analysis of soft-thresholding powers.

The scale-free fit (y-axis) against the soft-thresholding power (x-axis) is shown in the left panel, and the fit value (0.9) is shown as red horizontal lines. The mean connectivity (y-axis) against the soft-thresholding power (x-axis) is shown in the right panel. The name of TCGA dataset is located to the right top corner.

TCGA-SARC

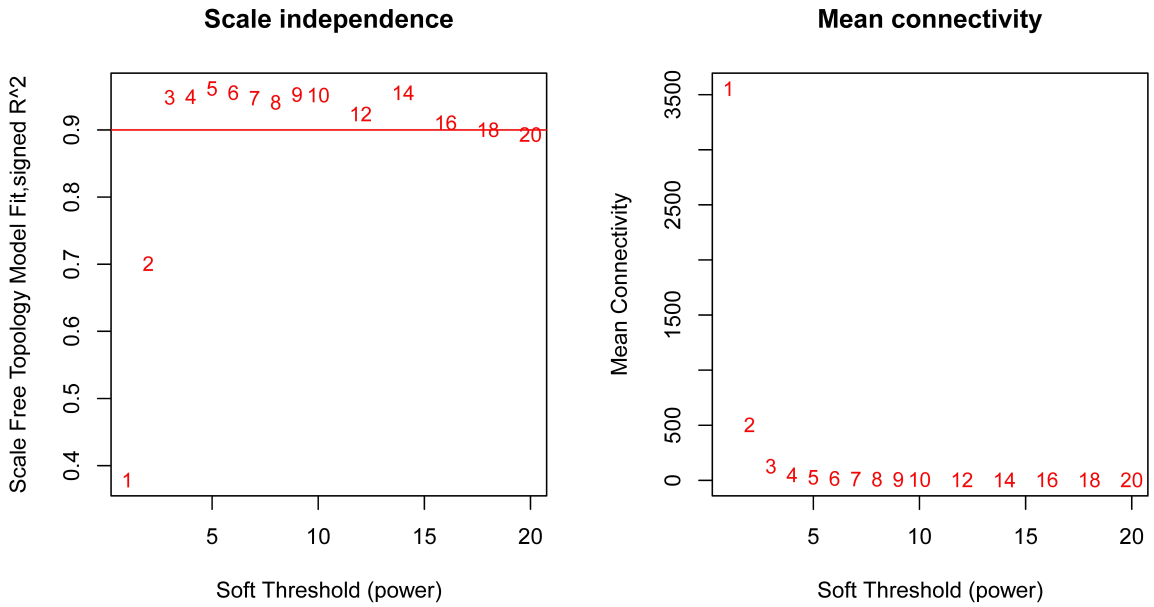

Supplementary Figure S3.25. Network topology analysis of soft-thresholding powers.

The scale-free fit (y-axis) against the soft-thresholding power (x-axis) is shown in the left panel, and the fit value (0.9) is shown as red horizontal lines. The mean connectivity (y-axis) against the soft-thresholding power (x-axis) is shown in the right panel. The name of TCGA dataset is located to the right top corner.

TCGA-SKCM

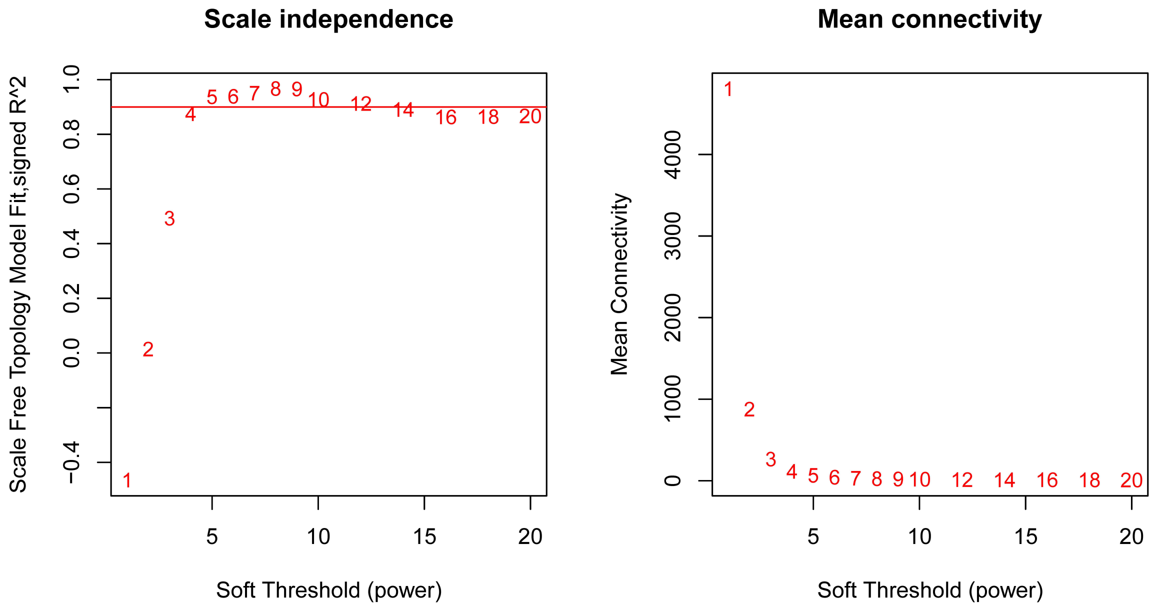

Supplementary Figure S3.26. Network topology analysis of soft-thresholding powers.

The scale-free fit (y-axis) against the soft-thresholding power (x-axis) is shown in the left panel, and the fit value (0.9) is shown as red horizontal lines. The mean connectivity (y-axis) against the soft-thresholding power (x-axis) is shown in the right panel. The name of TCGA dataset is located to the right top corner.

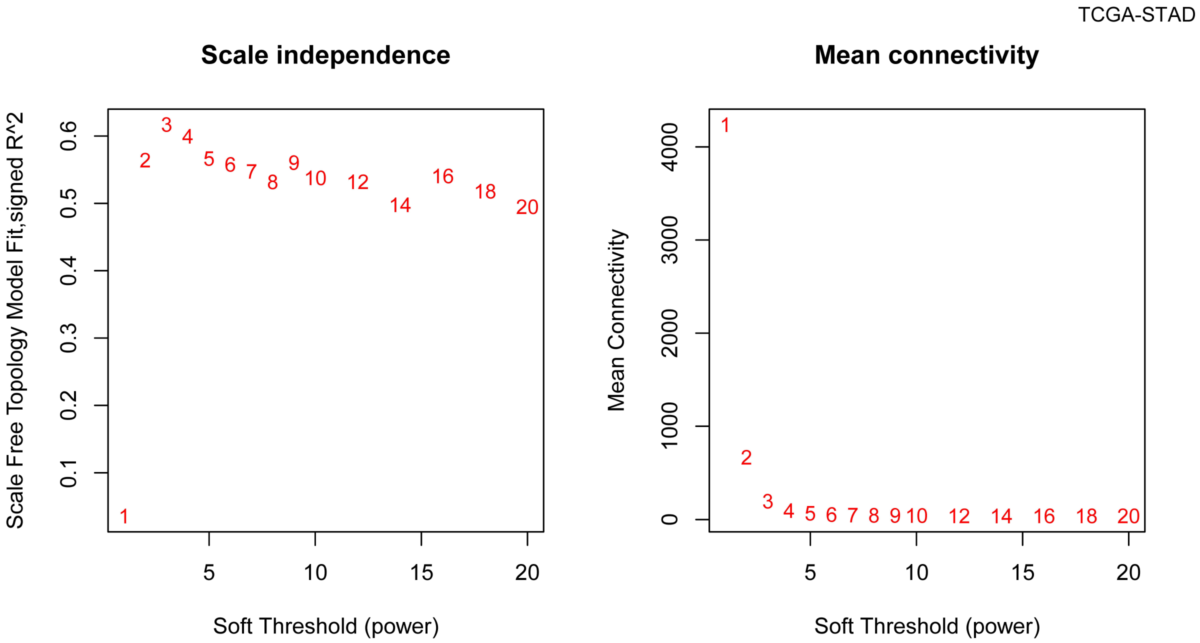

Supplementary Figure S3.27. Network topology analysis of soft-thresholding powers.

The scale-free fit (y-axis) against the soft-thresholding power (x-axis) is shown in the left panel, and the fit value (0.9) is shown as red horizontal lines. The mean connectivity (y-axis) against the soft-thresholding power (x-axis) is shown in the right panel. The name of TCGA dataset is located to the right top corner.

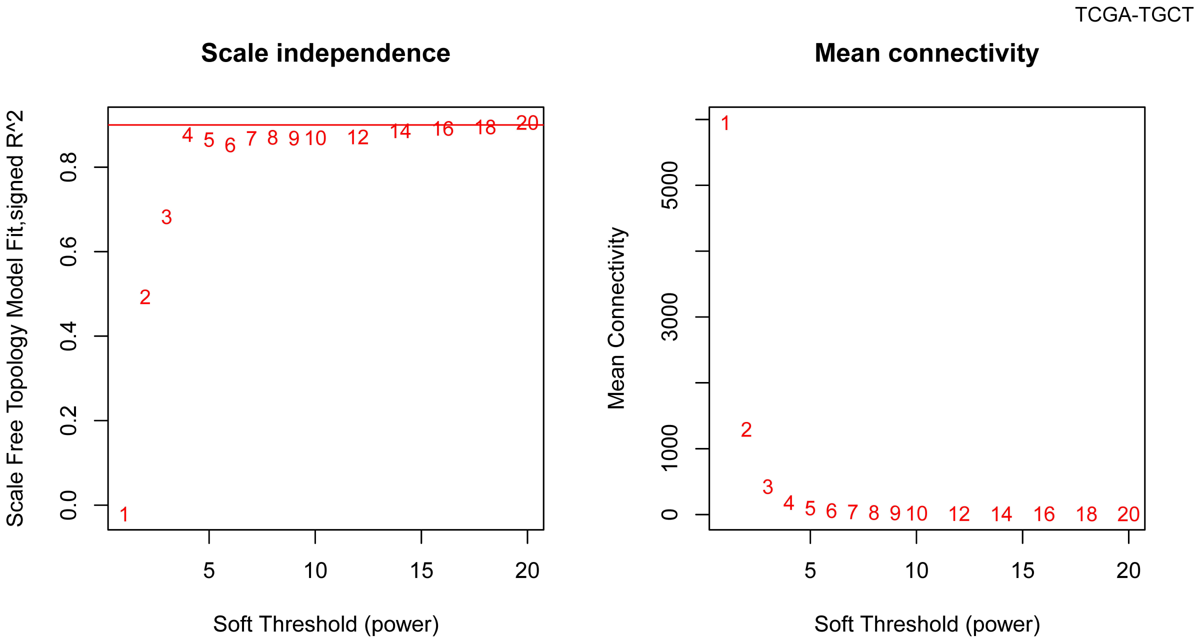

Supplementary Figure S3.28. Network topology analysis of soft-thresholding powers.

The scale-free fit (y-axis) against the soft-thresholding power (x-axis) is shown in the left panel, and the fit value (0.9) is shown as red horizontal lines. The mean connectivity (y-axis) against the soft-thresholding power (x-axis) is shown in the right panel. The name of TCGA dataset is located to the right top corner.

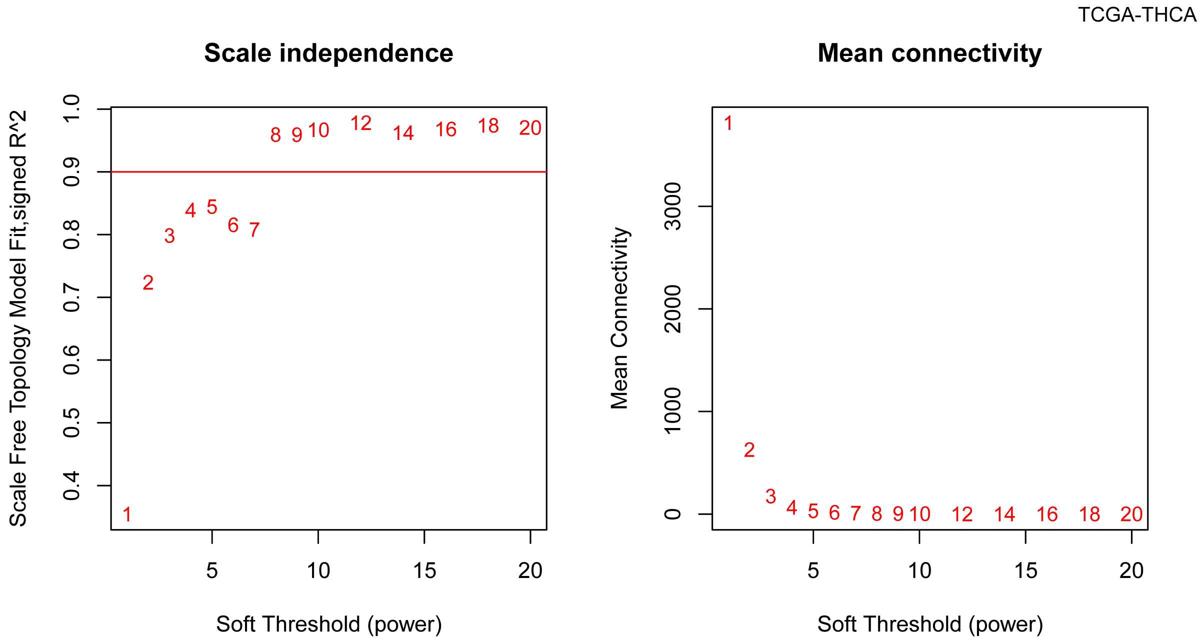

Supplementary Figure S3.29. Network topology analysis of soft-thresholding powers.

The scale-free fit (y-axis) against the soft-thresholding power (x-axis) is shown in the left panel, and the fit value (0.9) is shown as red horizontal lines. The mean connectivity (y-axis) against the soft-thresholding power (x-axis) is shown in the right panel. The name of TCGA dataset is located to the right top corner.

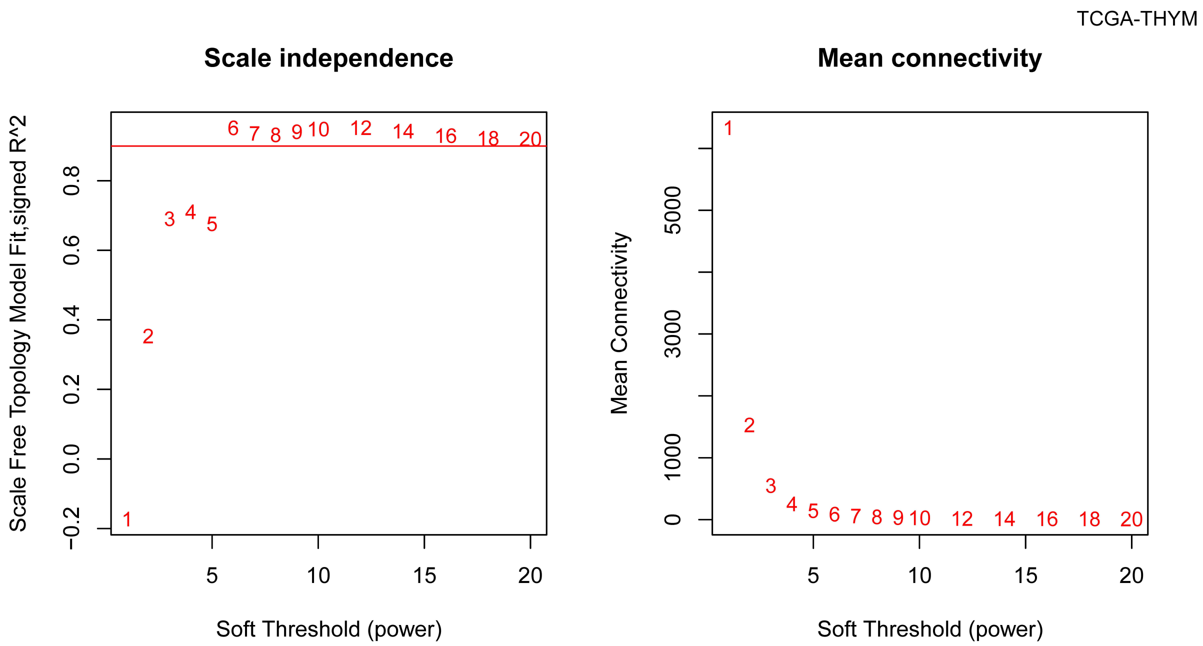

Supplementary Figure S3.30. Network topology analysis of soft-thresholding powers.

The scale-free fit (y-axis) against the soft-thresholding power (x-axis) is shown in the left panel, and the fit value (0.9) is shown as red horizontal lines. The mean connectivity (y-axis) against the soft-thresholding power (x-axis) is shown in the right panel. The name of TCGA dataset is located to the right top corner.

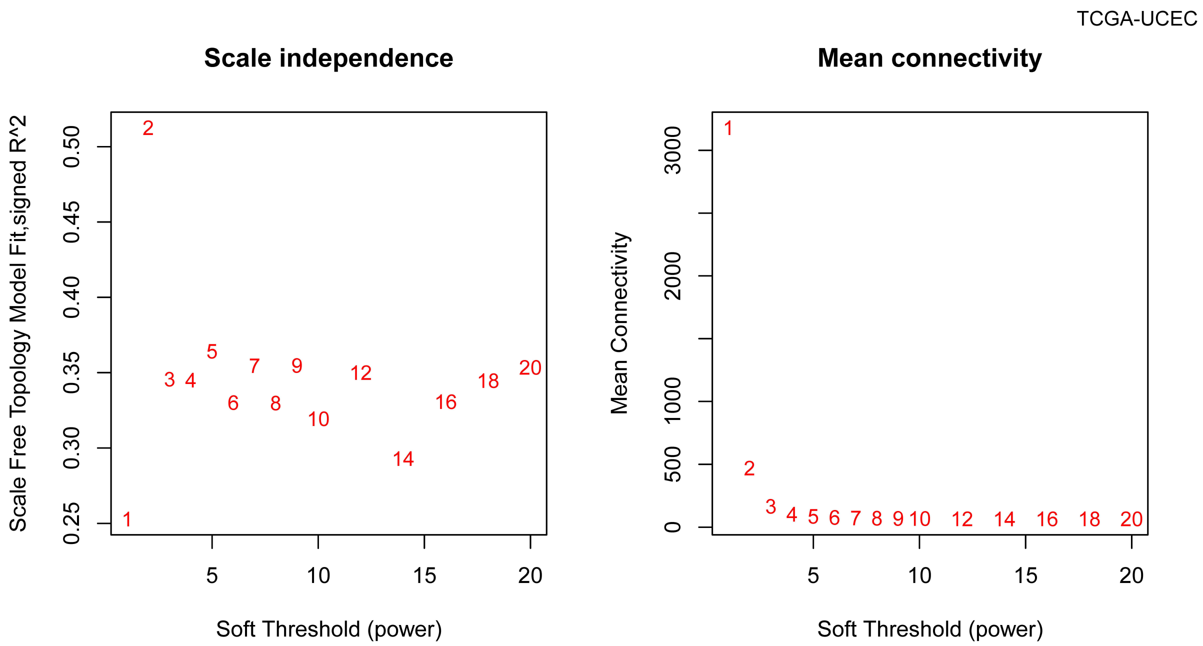

Supplementary Figure S3.31. Network topology analysis of soft-thresholding powers.

The scale-free fit (y-axis) against the soft-thresholding power (x-axis) is shown in the left panel, and the fit value (0.9) is shown as red horizontal lines. The mean connectivity (y-axis) against the soft-thresholding power (x-axis) is shown in the right panel. The name of TCGA dataset is located to the right top corner.

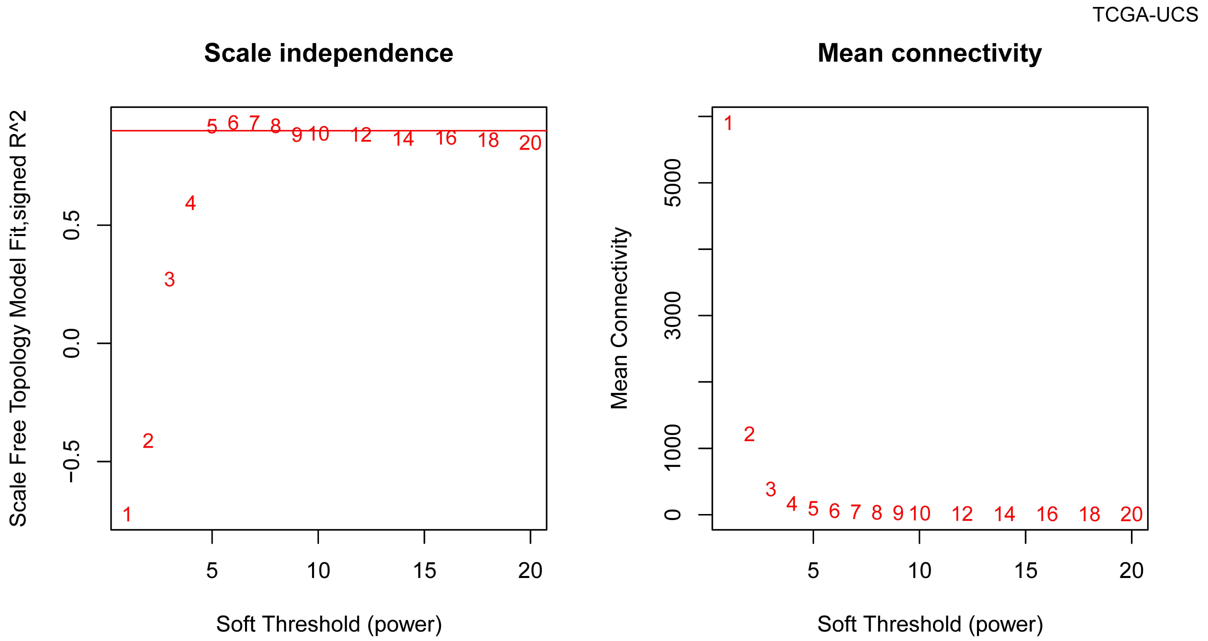

Supplementary Figure S3.32. Network topology analysis of soft-thresholding powers.

The scale-free fit (y-axis) against the soft-thresholding power (x-axis) is shown in the left panel, and the fit value (0.9) is shown as red horizontal lines. The mean connectivity (y-axis) against the soft-thresholding power (x-axis) is shown in the right panel. The name of TCGA dataset is located to the right top corner.

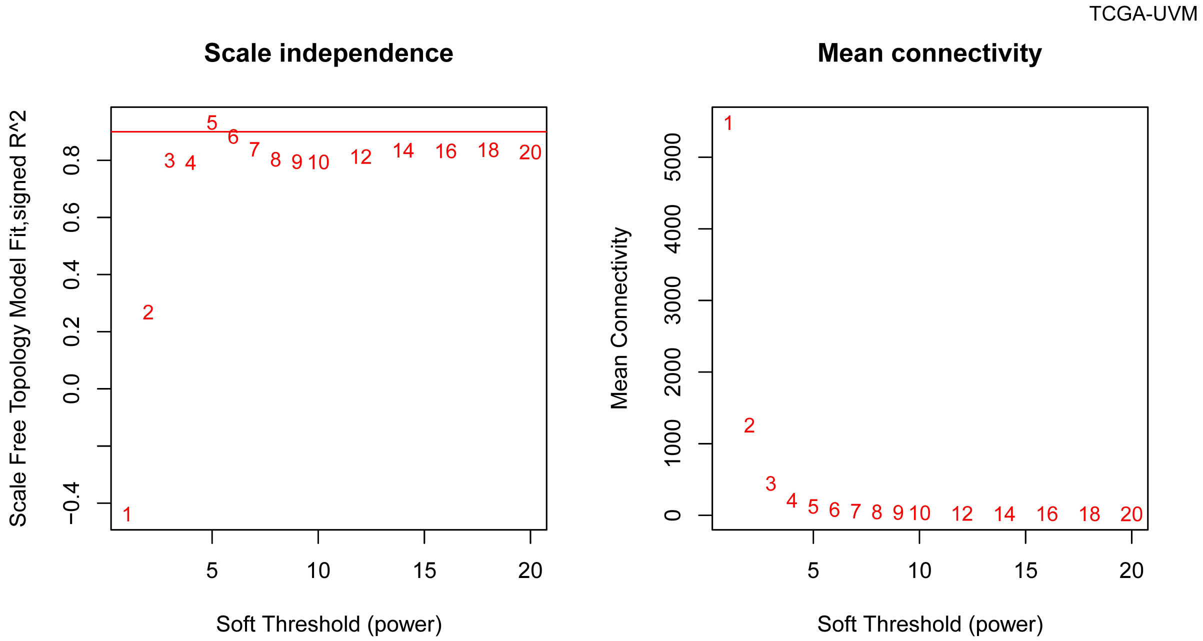

Supplementary Figure S3.33. Network topology analysis of soft-thresholding powers.

The scale-free fit (y-axis) against the soft-thresholding power (x-axis) is shown in the left panel, and the fit value (0.9) is shown as red horizontal lines. The mean connectivity (y-axis) against the soft-thresholding power (x-axis) is shown in the right panel. The name of TCGA dataset is located to the right top corner.

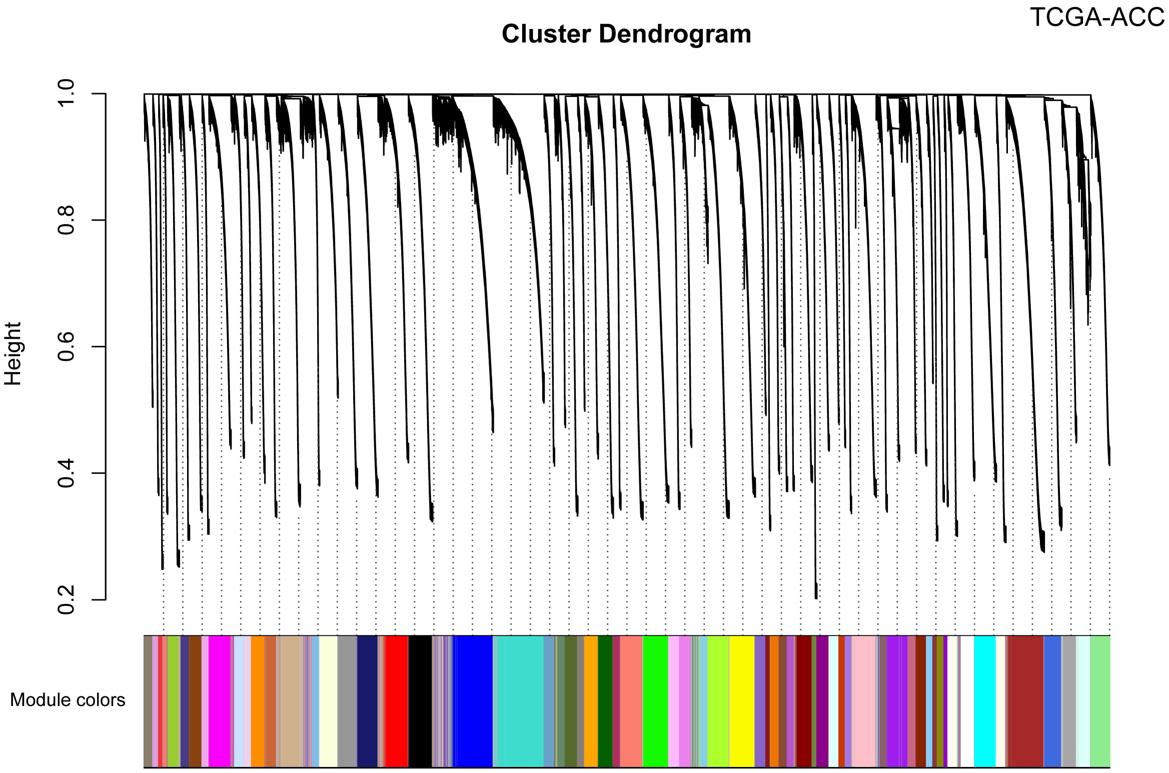

Supplementary Figure S4.1. Dendrogram of weighted gene co-expression network and module colors.

The genes clustered on the topological overlap matrix (TOM) based on dissimilarity, formed a branch-like shape, and the modules of those genes with high inter-connectivity clustered at the same module. The genes of grey module color are not assigned to any modules. The name of TCGA dataset is located to the right top corner.

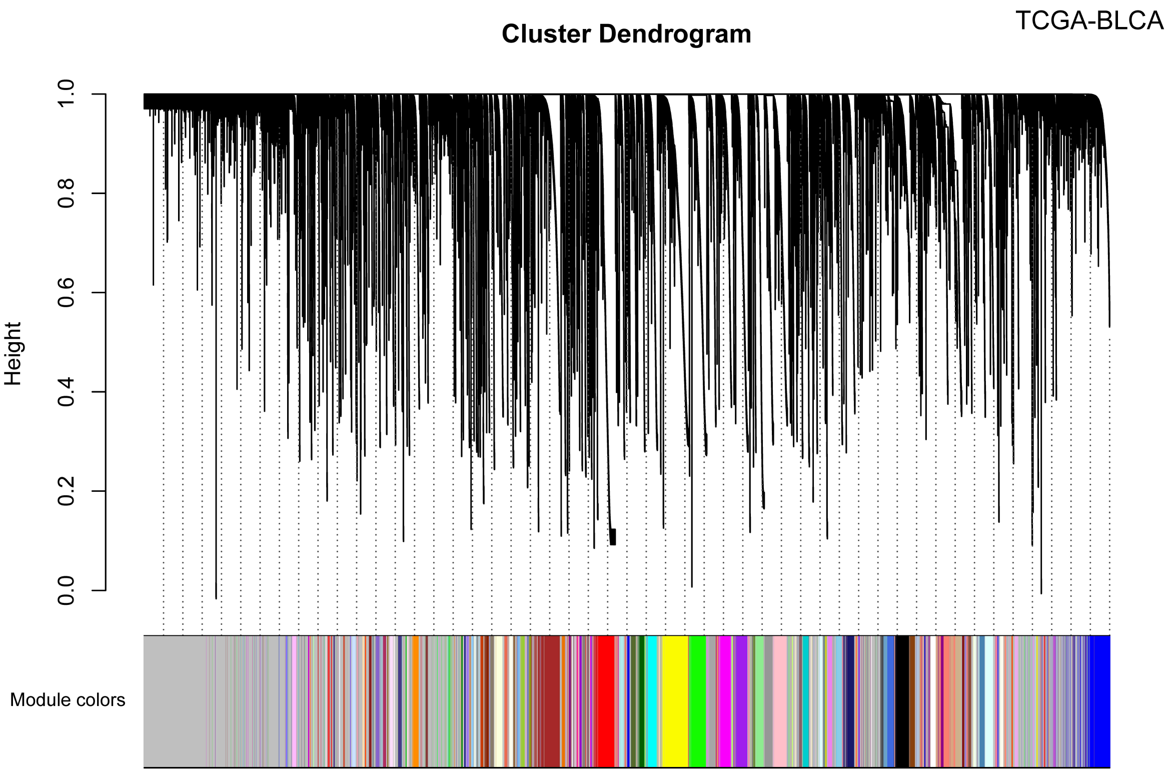

Supplementary Figure S4.2. Dendrogram of weighted gene co-expression network and module colors.

The genes clustered on the topological overlap matrix (TOM) based on dissimilarity, formed a branch-like shape, and the modules of those genes with high inter-connectivity clustered at the same module. The genes of grey module color are not assigned to any modules. The name of TCGA dataset is located to the right top corner.

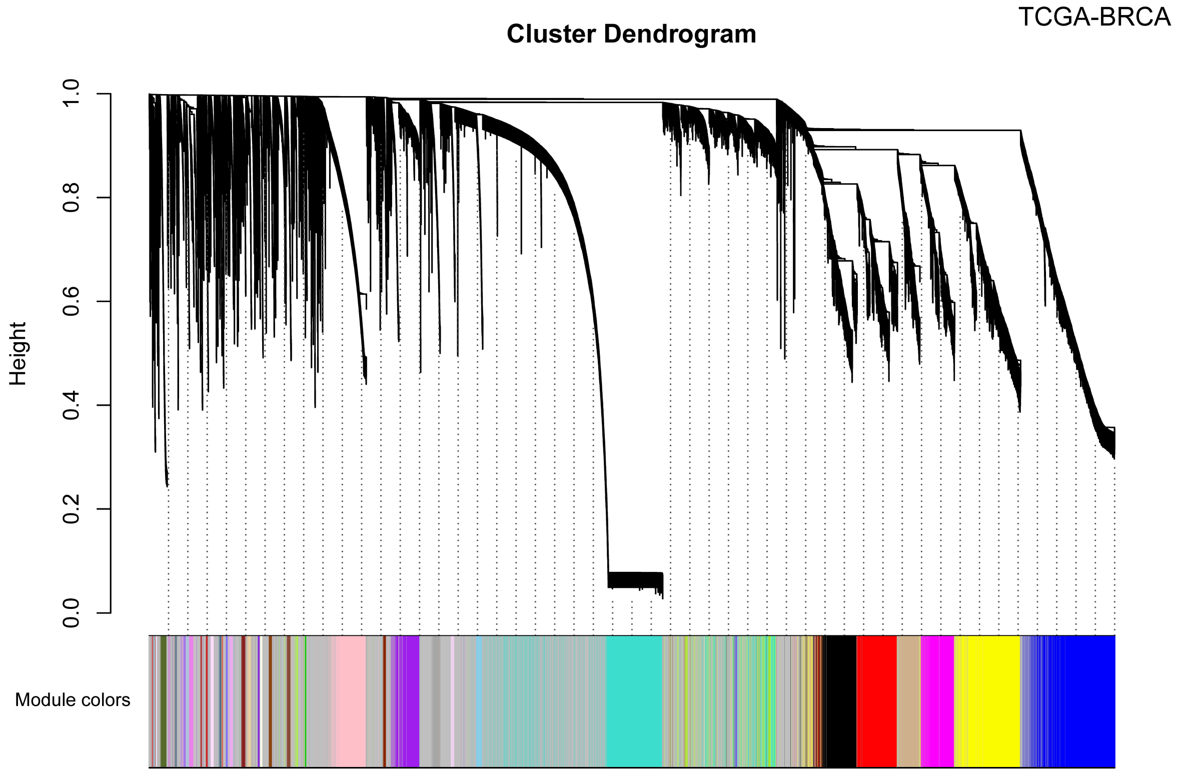

Supplementary Figure S4.3. Dendrogram of weighted gene co-expression network and module colors.

The genes clustered on the topological overlap matrix (TOM) based on dissimilarity, formed a branch-like shape, and the modules of those genes with high inter-connectivity clustered at the same module. The genes of grey module color are not assigned to any modules. The name of TCGA dataset is located to the right top corner.

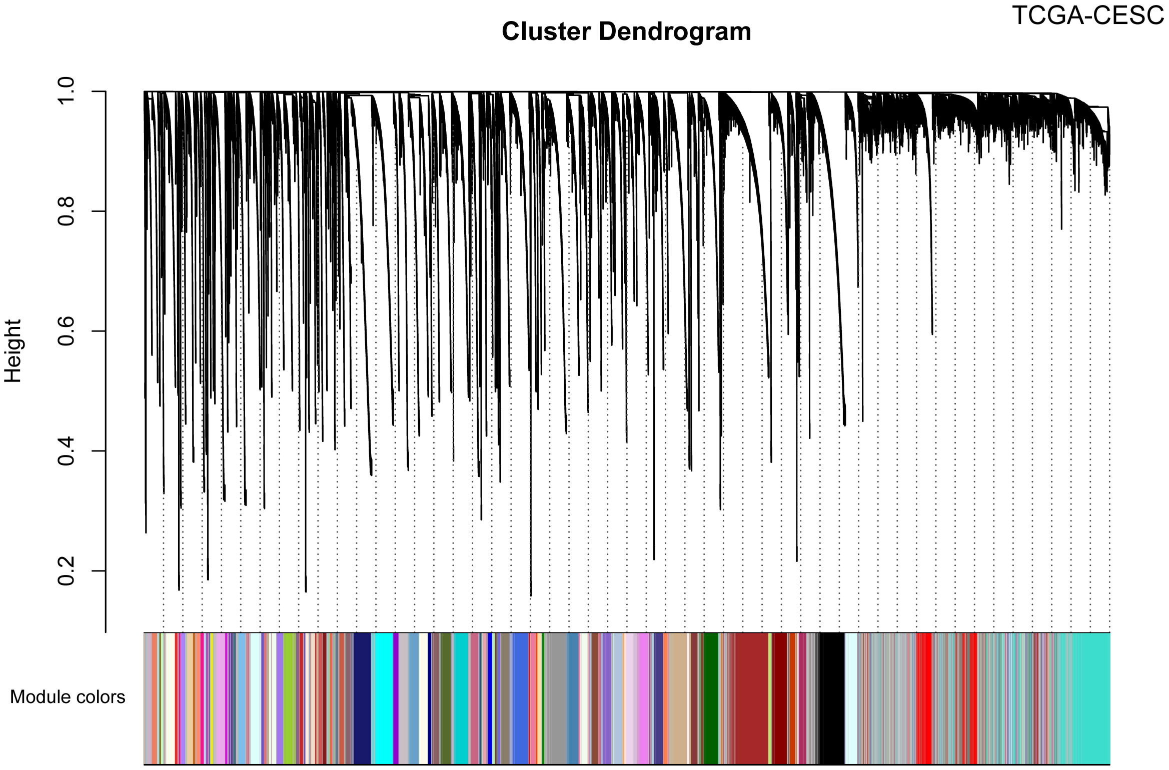

Supplementary Figure S4.4. Dendrogram of weighted gene co-expression network and module colors.

The genes clustered on the topological overlap matrix (TOM) based on dissimilarity, formed a branch-like shape, and the modules of those genes with high inter-connectivity clustered at the same module. The genes of grey module color are not assigned to any modules. The name of TCGA dataset is located to the right top corner.

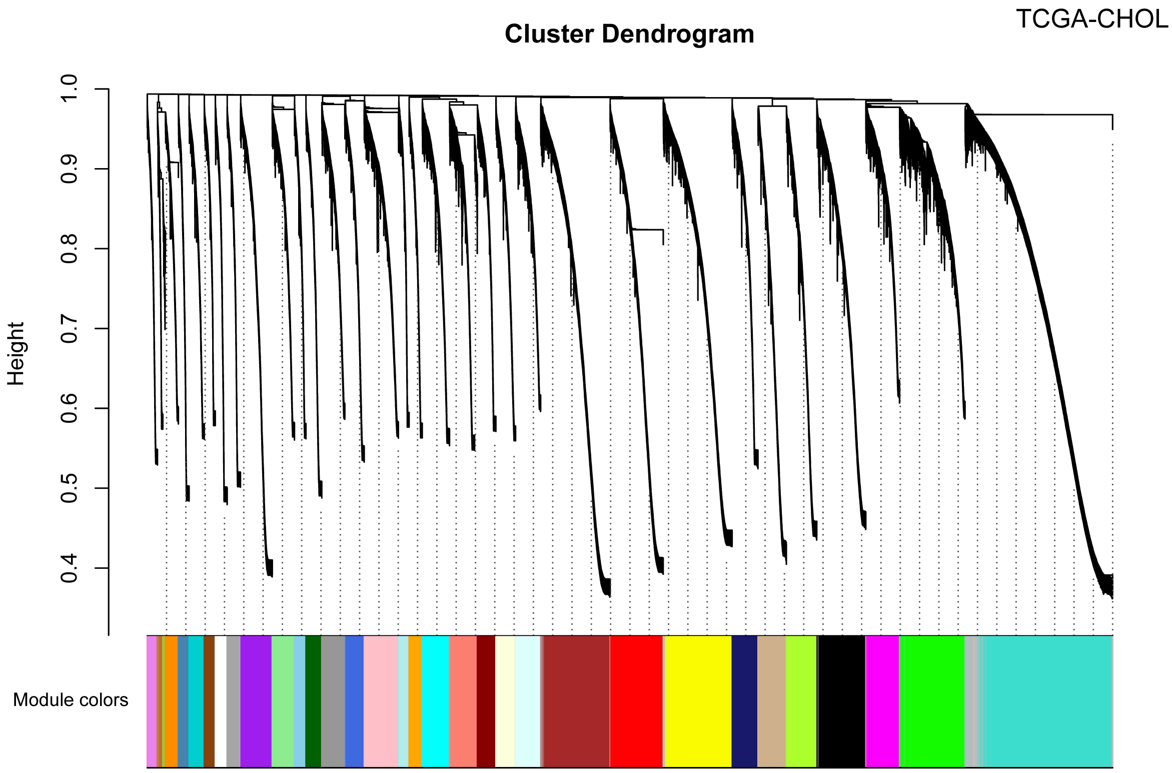

Supplementary Figure S4.5. Dendrogram of weighted gene co-expression network and module colors.

The genes clustered on the topological overlap matrix (TOM) based on dissimilarity, formed a branch-like shape, and the modules of those genes with high inter-connectivity clustered at the same module. The genes of grey module color are not assigned to any modules. The name of TCGA dataset is located to the right top corner.

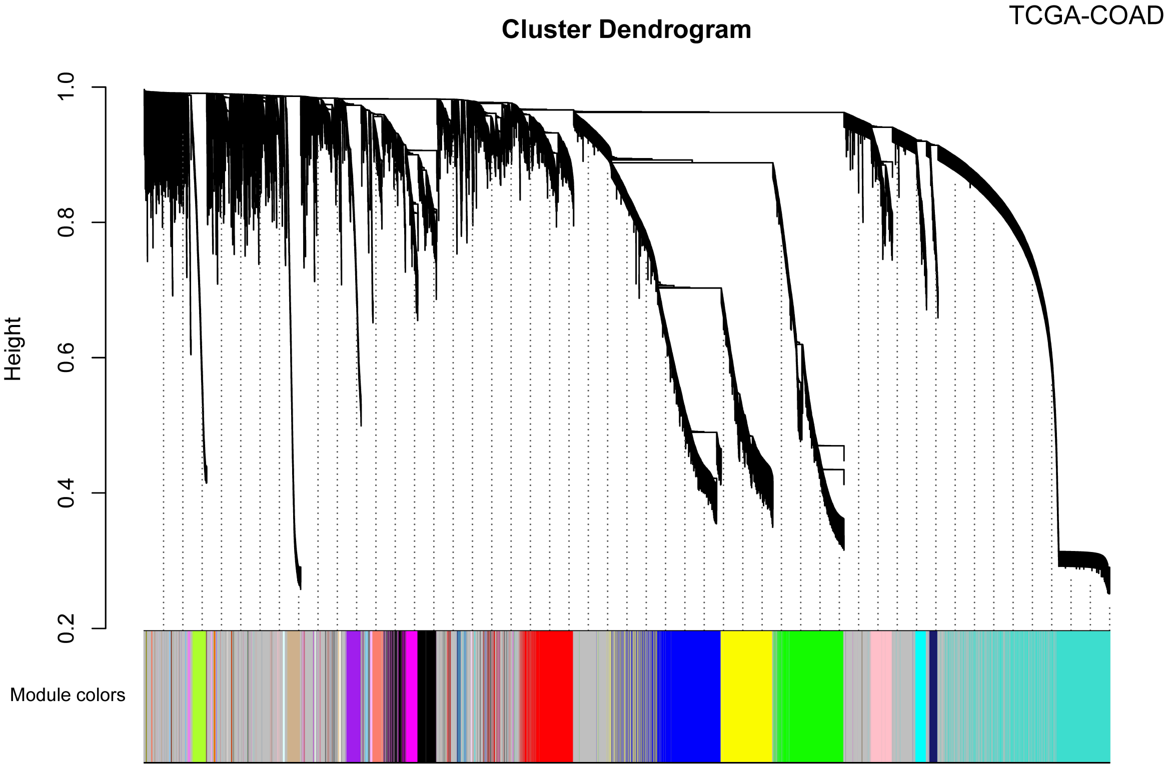

Supplementary Figure S4.6. Dendrogram of weighted gene co-expression network and module colors.

The genes clustered on the topological overlap matrix (TOM) based on dissimilarity, formed a branch-like shape, and the modules of those genes with high inter-connectivity clustered at the same module. The genes of grey module color are not assigned to any modules. The name of TCGA dataset is located to the right top corner.

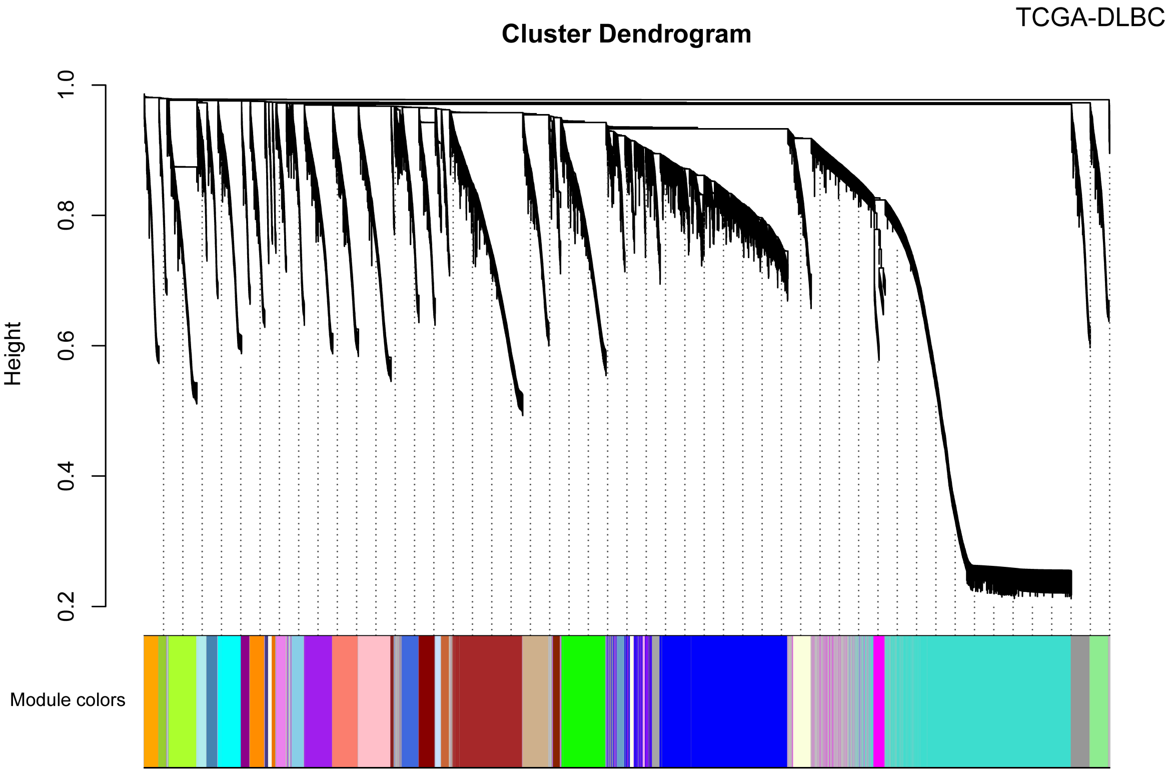

Supplementary Figure S4.7. Dendrogram of weighted gene co-expression network and module colors.

The genes clustered on the topological overlap matrix (TOM) based on dissimilarity, formed a branch-like shape, and the modules of those genes with high inter-connectivity clustered at the same module. The genes of grey module color are not assigned to any modules. The name of TCGA dataset is located to the right top corner.

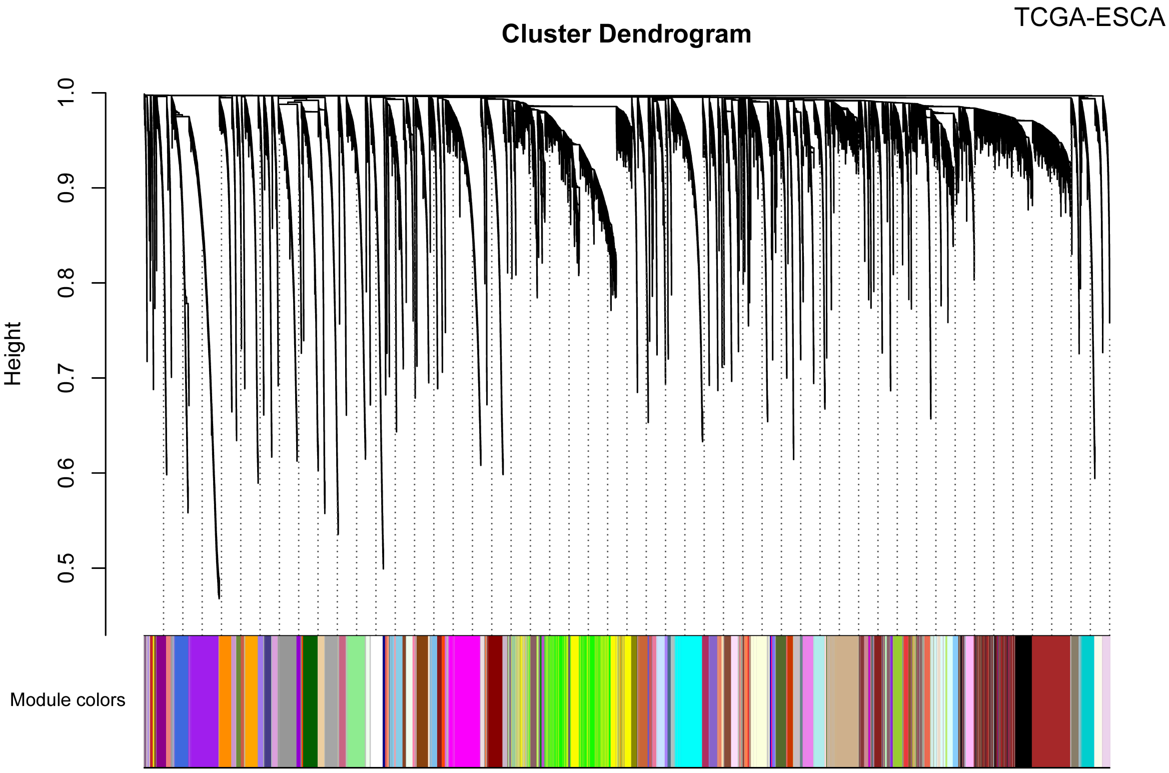

Supplementary Figure S4.8. Dendrogram of weighted gene co-expression network and module colors.

The genes clustered on the topological overlap matrix (TOM) based on dissimilarity, formed a branch-like shape, and the modules of those genes with high inter-connectivity clustered at the same module. The genes of grey module color are not assigned to any modules. The name of TCGA dataset is located to the right top corner.

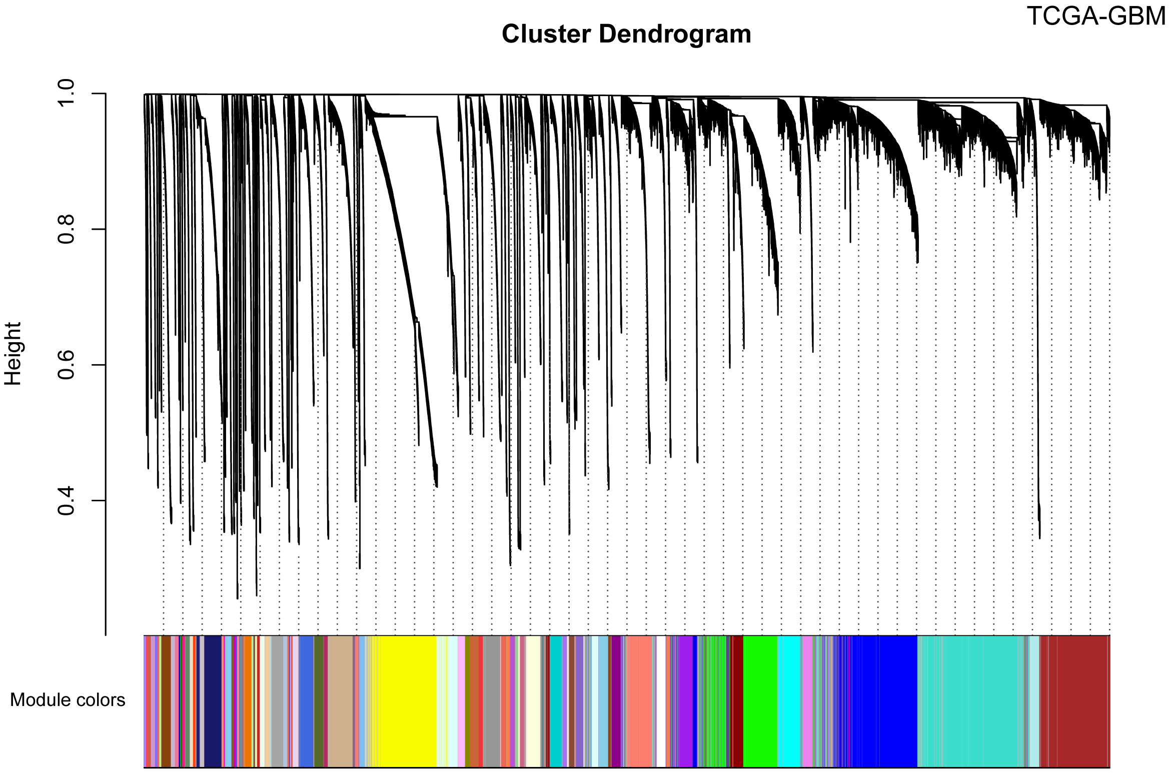

Supplementary Figure S4.9. Dendrogram of weighted gene co-expression network and module colors.

The genes clustered on the topological overlap matrix (TOM) based on dissimilarity, formed a branch-like shape, and the modules of those genes with high inter-connectivity clustered at the same module. The genes of grey module color are not assigned to any modules. The name of TCGA dataset is located to the right top corner.

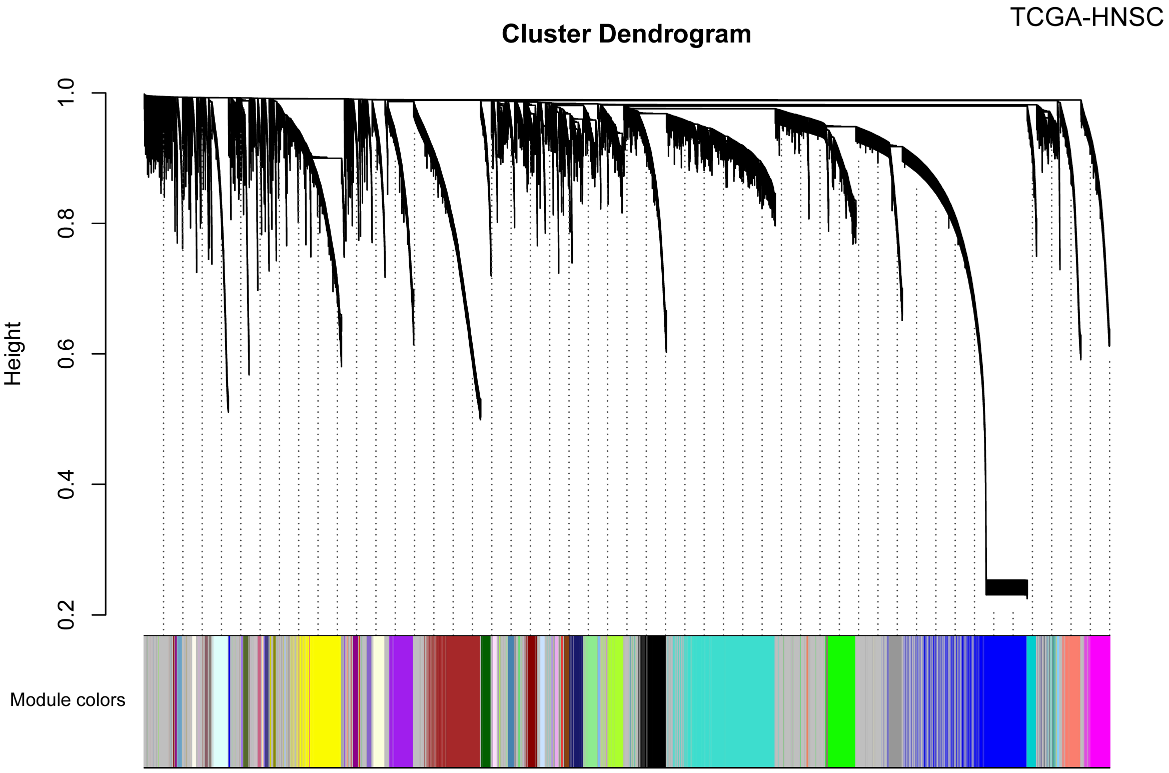

Supplementary Figure S4.10. Dendrogram of weighted gene co-expression network and module colors.

The genes clustered on the topological overlap matrix (TOM) based on dissimilarity, formed a branch-like shape, and the modules of those genes with high inter-connectivity clustered at the same module. The genes of grey module color are not assigned to any modules. The name of TCGA dataset is located to the right top corner.

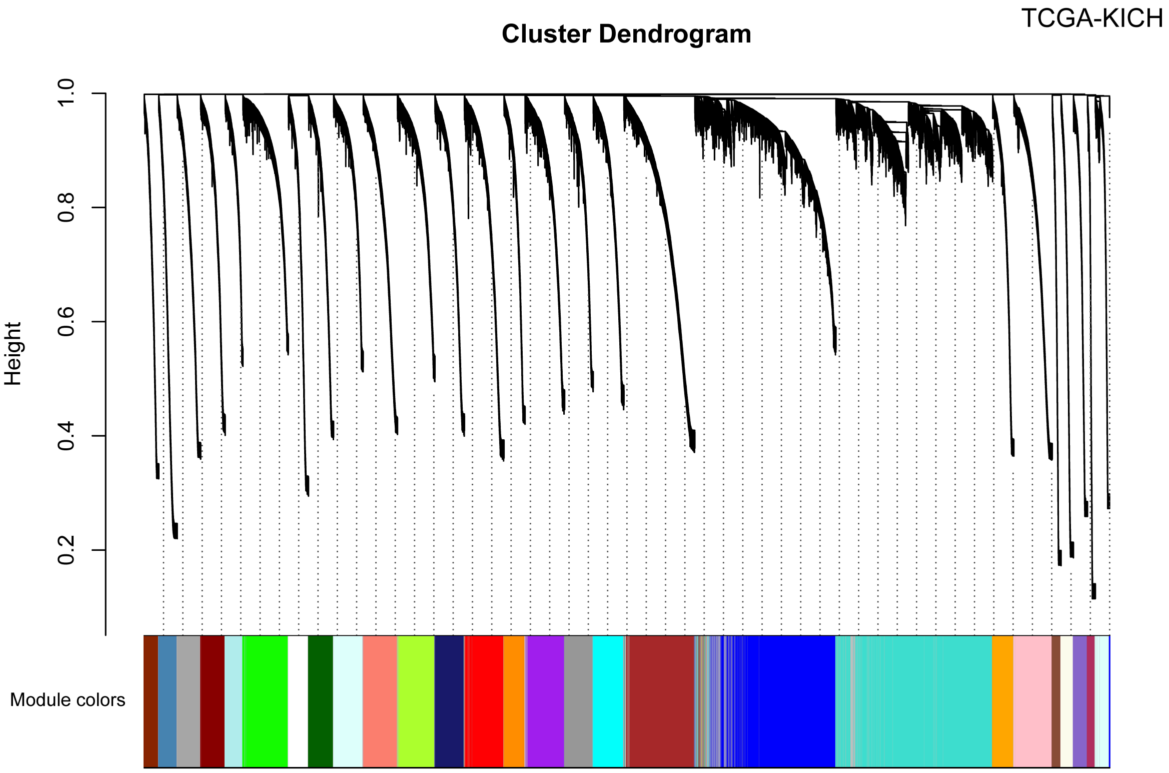

Supplementary Figure S4.11. Dendrogram of weighted gene co-expression network and module colors.

The genes clustered on the topological overlap matrix (TOM) based on dissimilarity, formed a branch-like shape, and the modules of those genes with high inter-connectivity clustered at the same module. The genes of grey module color are not assigned to any modules. The name of TCGA dataset is located to the right top corner.

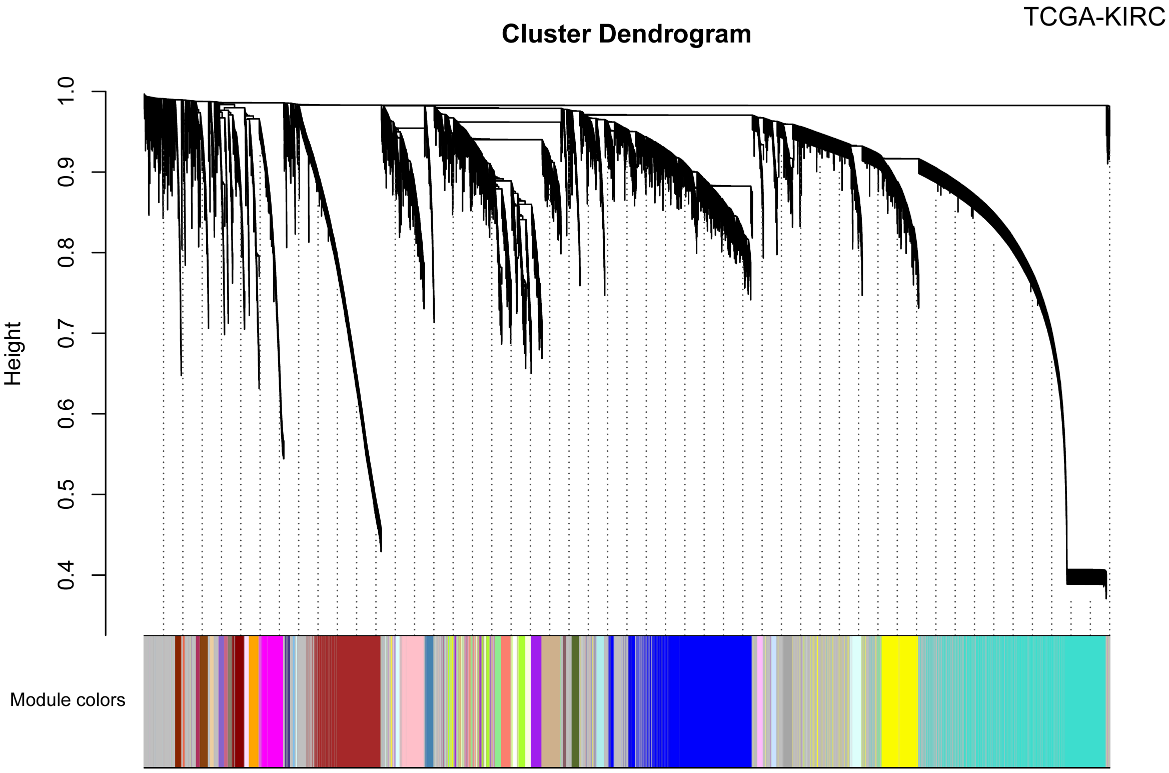

Supplementary Figure S4.12. Dendrogram of weighted gene co-expression network and module colors.

The genes clustered on the topological overlap matrix (TOM) based on dissimilarity, formed a branch-like shape, and the modules of those genes with high inter-connectivity clustered at the same module. The genes of grey module color are not assigned to any modules. The name of TCGA dataset is located to the right top corner.

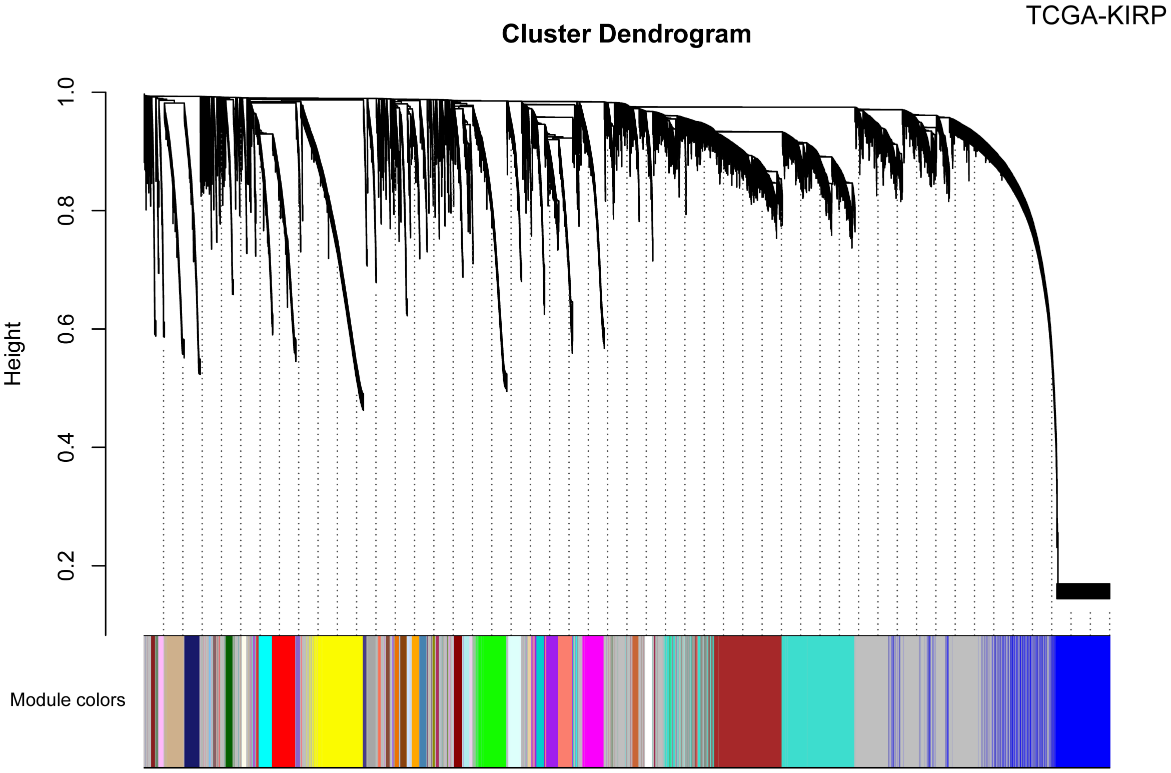

Supplementary Figure S4.13. Dendrogram of weighted gene co-expression network and module colors.

The genes clustered on the topological overlap matrix (TOM) based on dissimilarity, formed a branch-like shape, and the modules of those genes with high inter-connectivity clustered at the same module. The genes of grey module color are not assigned to any modules. The name of TCGA dataset is located to the right top corner.

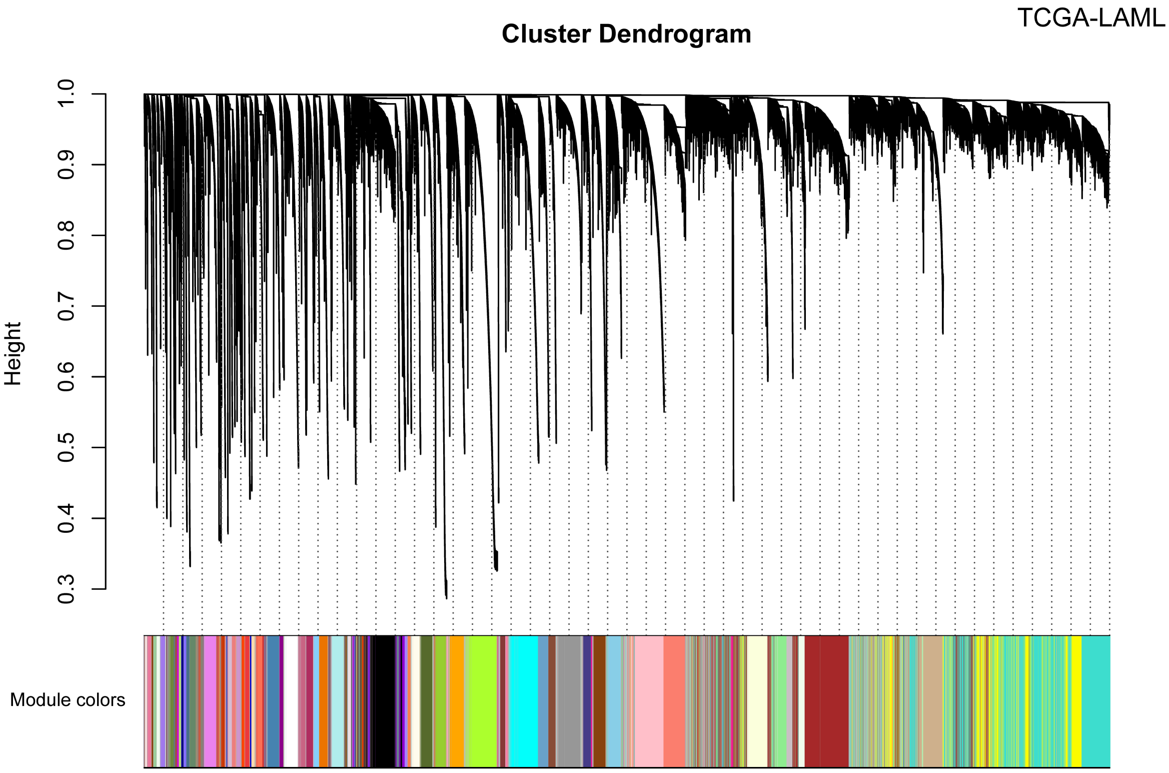

Supplementary Figure S4.14. Dendrogram of weighted gene co-expression network and module colors.

The genes clustered on the topological overlap matrix (TOM) based on dissimilarity, formed a branch-like shape, and the modules of those genes with high inter-connectivity clustered at the same module. The genes of grey module color are not assigned to any modules. The name of TCGA dataset is located to the right top corner.

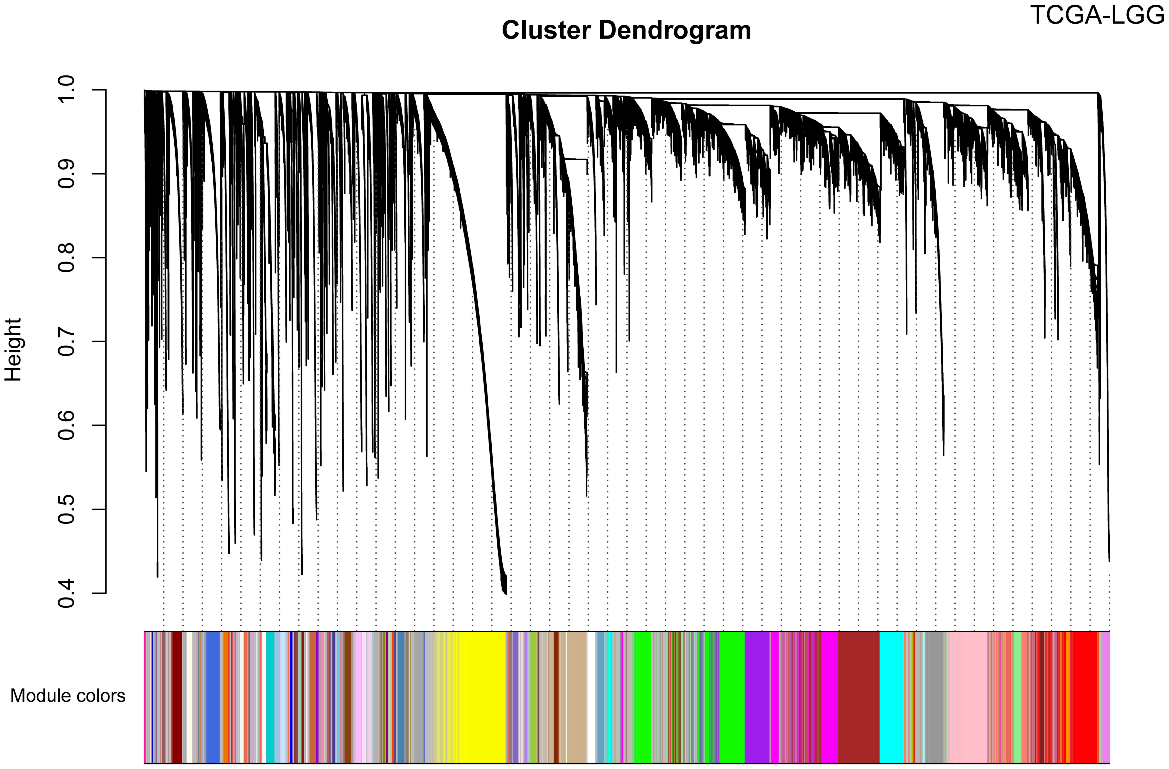

Supplementary Figure S4.15. Dendrogram of weighted gene co-expression network and module colors.

The genes clustered on the topological overlap matrix (TOM) based on dissimilarity, formed a branch-like shape, and the modules of those genes with high inter-connectivity clustered at the same module. The genes of grey module color are not assigned to any modules. The name of TCGA dataset is located to the right top corner.

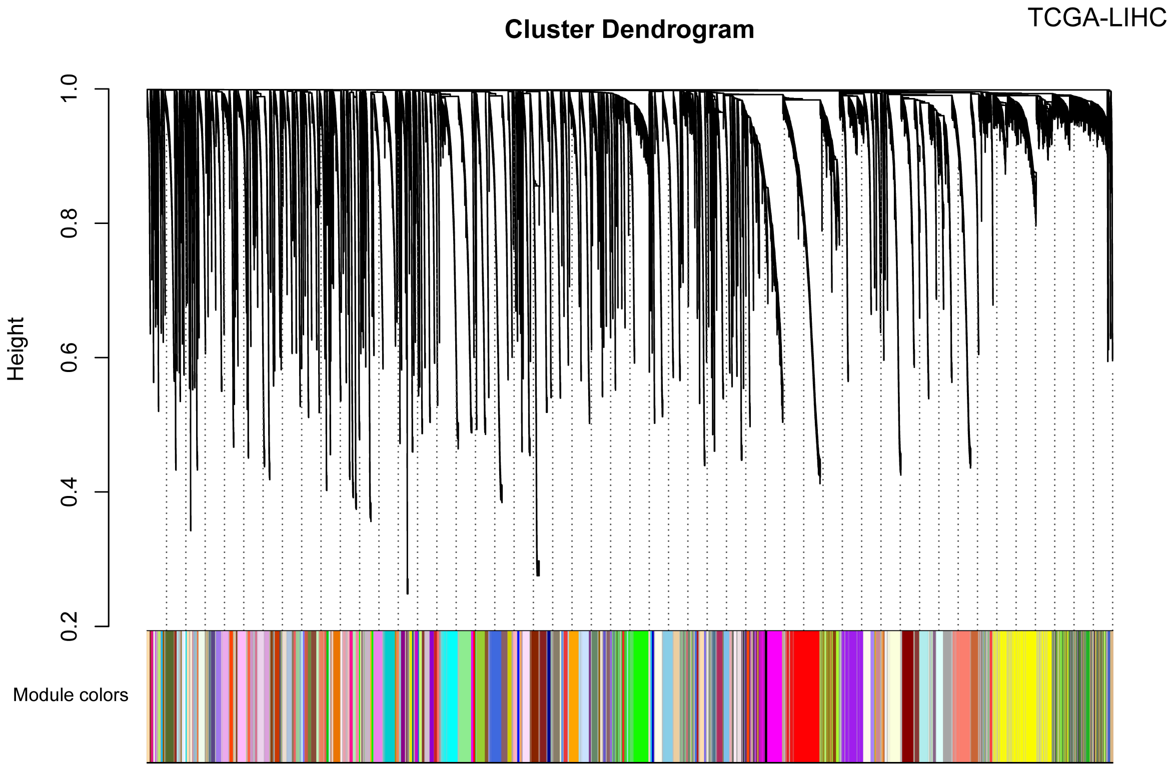

Supplementary Figure S4.16. Dendrogram of weighted gene co-expression network and module colors.

The genes clustered on the topological overlap matrix (TOM) based on dissimilarity, formed a branch-like shape, and the modules of those genes with high inter-connectivity clustered at the same module. The genes of grey module color are not assigned to any modules. The name of TCGA dataset is located to the right top corner.

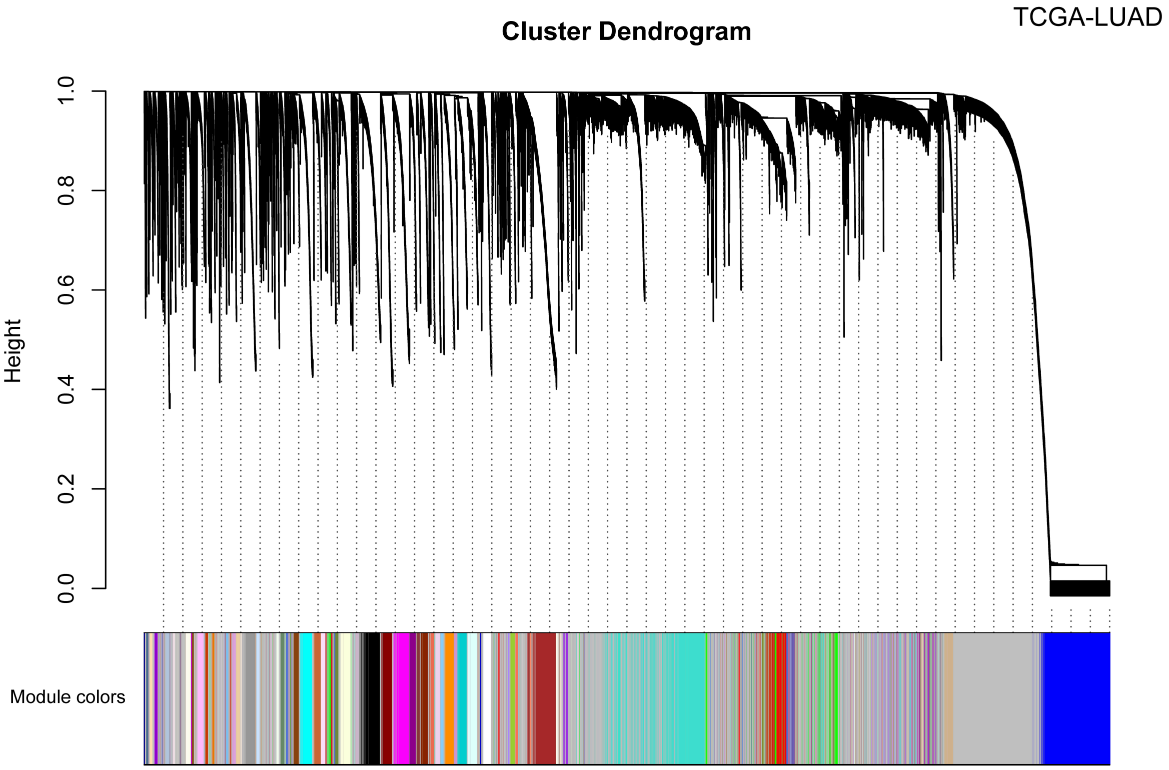

Supplementary Figure S4.17. Dendrogram of weighted gene co-expression network and module colors.

The genes clustered on the topological overlap matrix (TOM) based on dissimilarity, formed a branch-like shape, and the modules of those genes with high inter-connectivity clustered at the same module. The genes of grey module color are not assigned to any modules. The name of TCGA dataset is located to the right top corner.

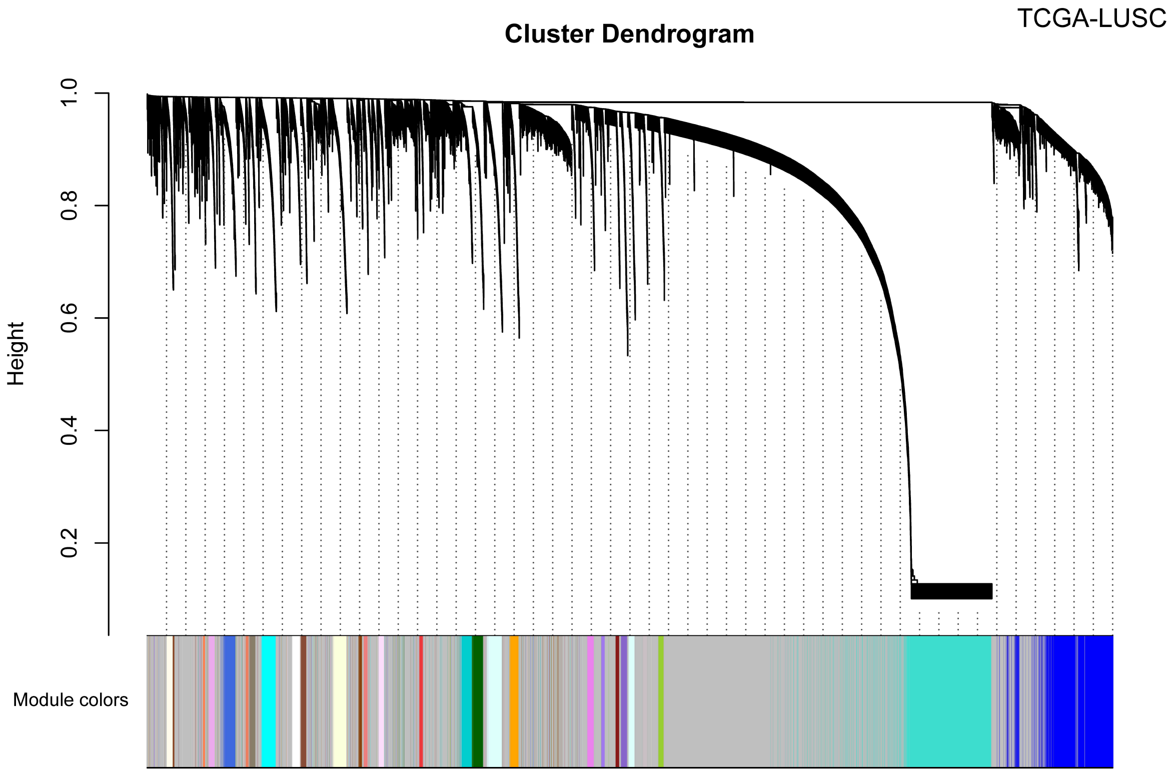

Supplementary Figure S4.18. Dendrogram of weighted gene co-expression network and module colors.

The genes clustered on the topological overlap matrix (TOM) based on dissimilarity, formed a branch-like shape, and the modules of those genes with high inter-connectivity clustered at the same module. The genes of grey module color are not assigned to any modules. The name of TCGA dataset is located to the right top corner.

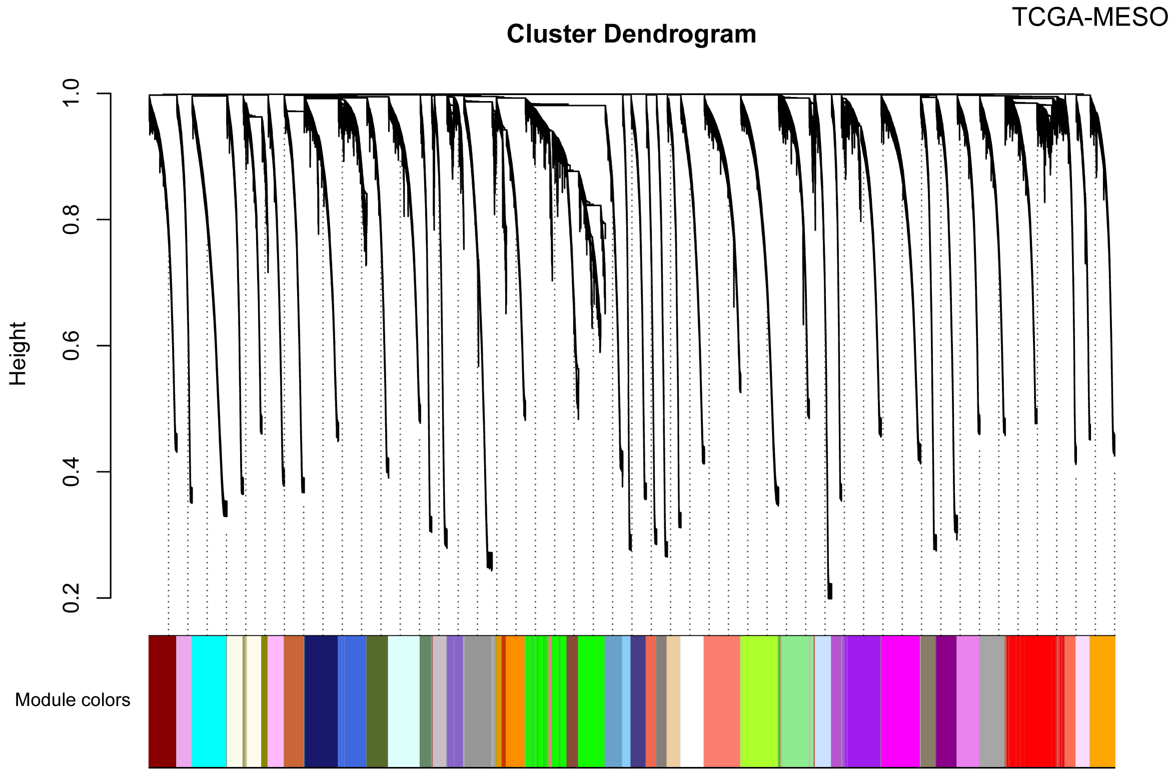

Supplementary Figure S4.19. Dendrogram of weighted gene co-expression network and module colors.

The genes clustered on the topological overlap matrix (TOM) based on dissimilarity, formed a branch-like shape, and the modules of those genes with high inter-connectivity clustered at the same module. The genes of grey module color are not assigned to any modules. The name of TCGA dataset is located to the right top corner.

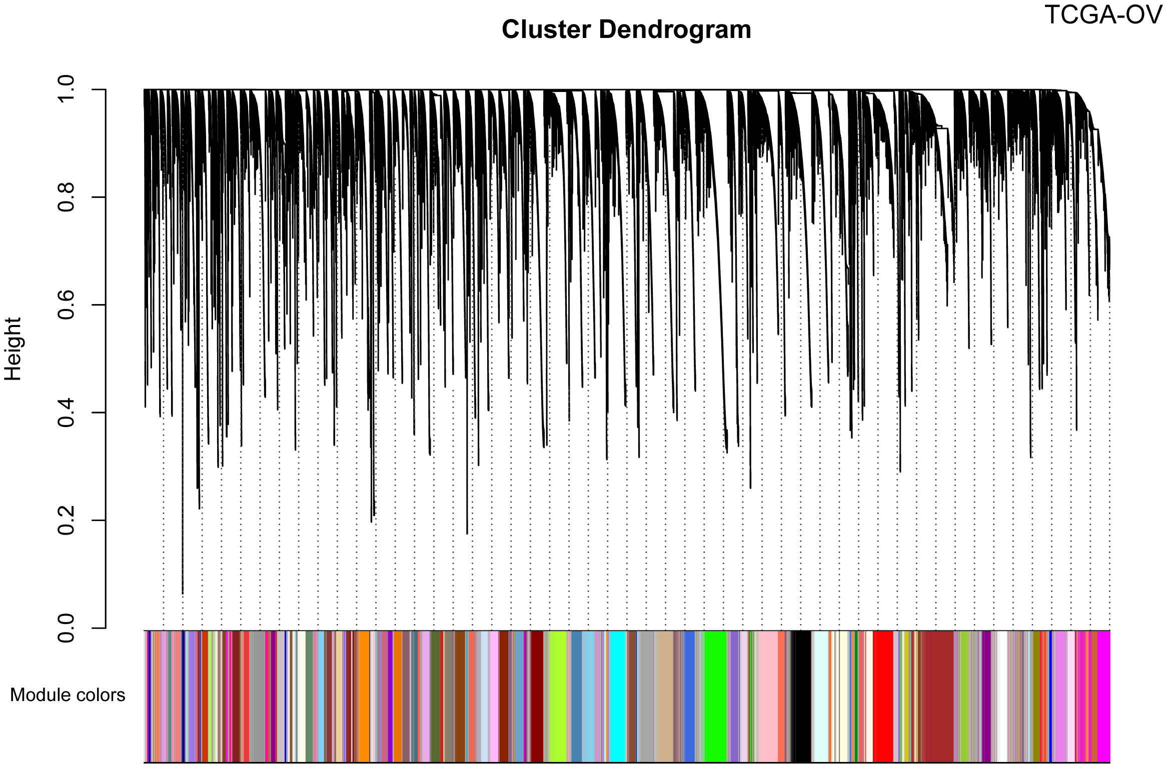

Supplementary Figure S4.20. Dendrogram of weighted gene co-expression network and module colors.

The genes clustered on the topological overlap matrix (TOM) based on dissimilarity, formed a branch-like shape, and the modules of those genes with high inter-connectivity clustered at the same module. The genes of grey module color are not assigned to any modules. The name of TCGA dataset is located to the right top corner.

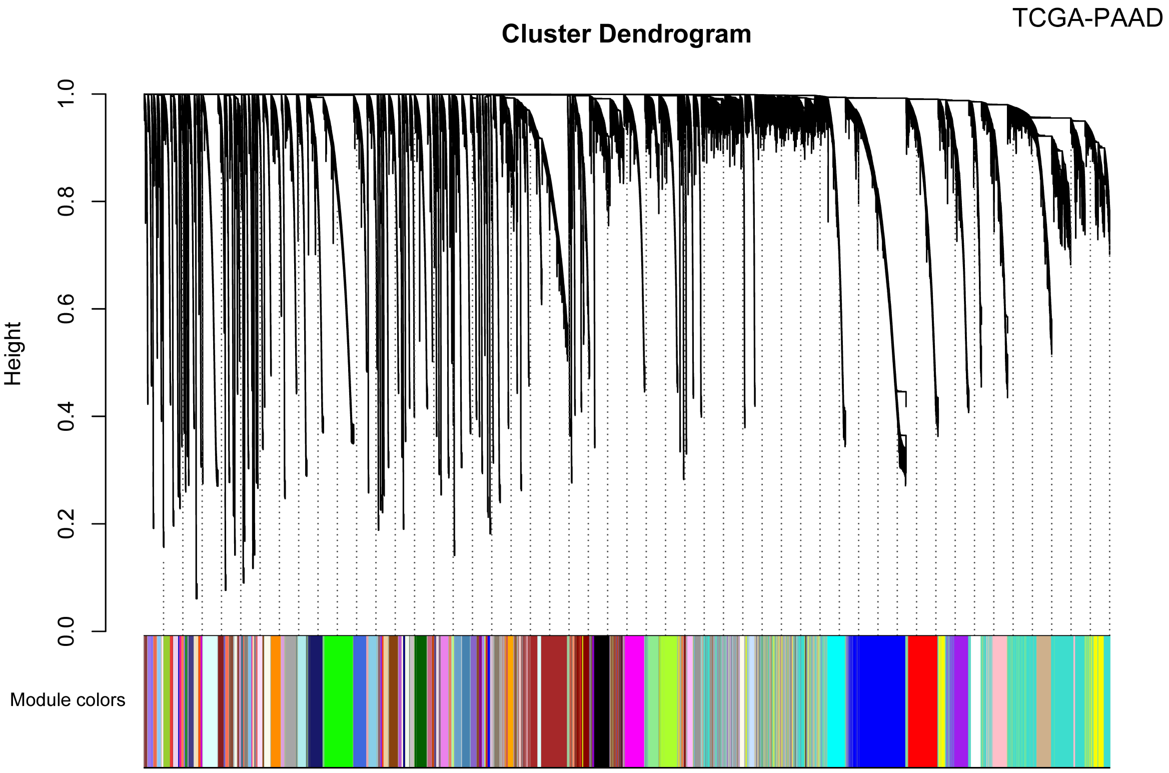

Supplementary Figure S4.21. Dendrogram of weighted gene co-expression network and module colors.

The genes clustered on the topological overlap matrix (TOM) based on dissimilarity, formed a branch-like shape, and the modules of those genes with high inter-connectivity clustered at the same module. The genes of grey module color are not assigned to any modules. The name of TCGA dataset is located to the right top corner.

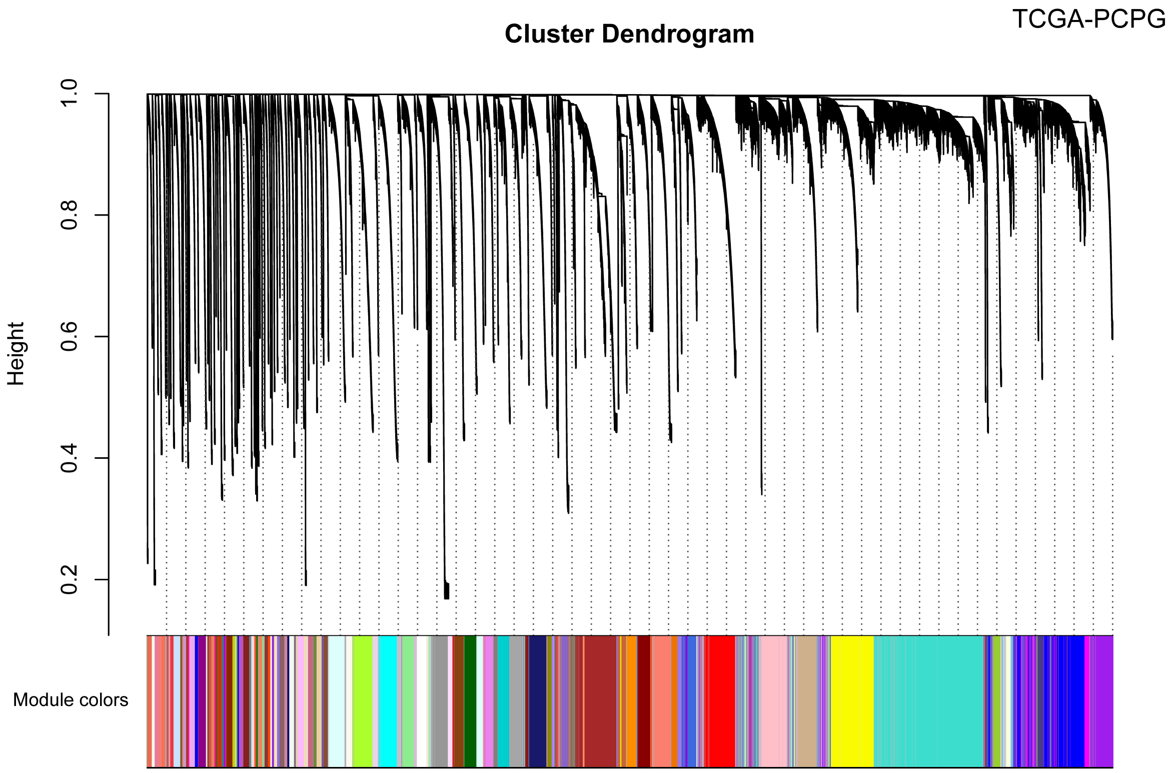

Supplementary Figure S4.22. Dendrogram of weighted gene co-expression network and module colors.

The genes clustered on the topological overlap matrix (TOM) based on dissimilarity, formed a branch-like shape, and the modules of those genes with high inter-connectivity clustered at the same module. The genes of grey module color are not assigned to any modules. The name of TCGA dataset is located to the right top corner.

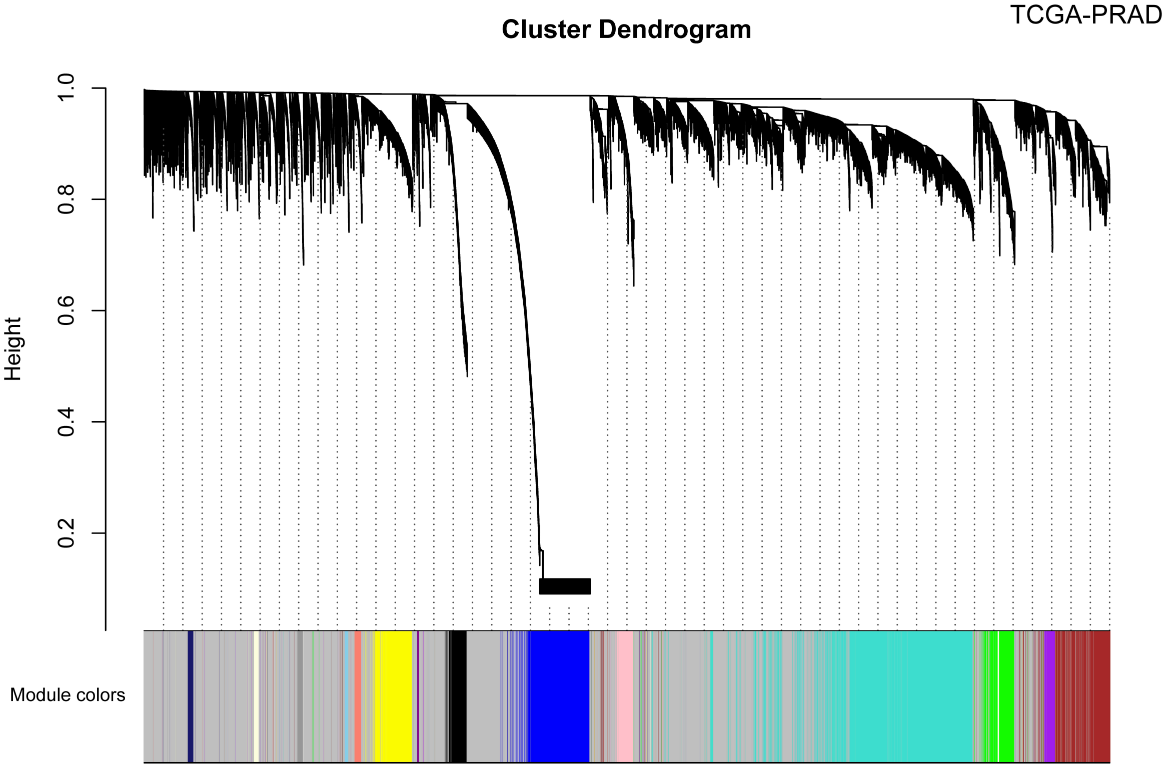

Supplementary Figure S4.23. Dendrogram of weighted gene co-expression network and module colors.

The genes clustered on the topological overlap matrix (TOM) based on dissimilarity, formed a branch-like shape, and the modules of those genes with high inter-connectivity clustered at the same module. The genes of grey module color are not assigned to any modules. The name of TCGA dataset is located to the right top corner.

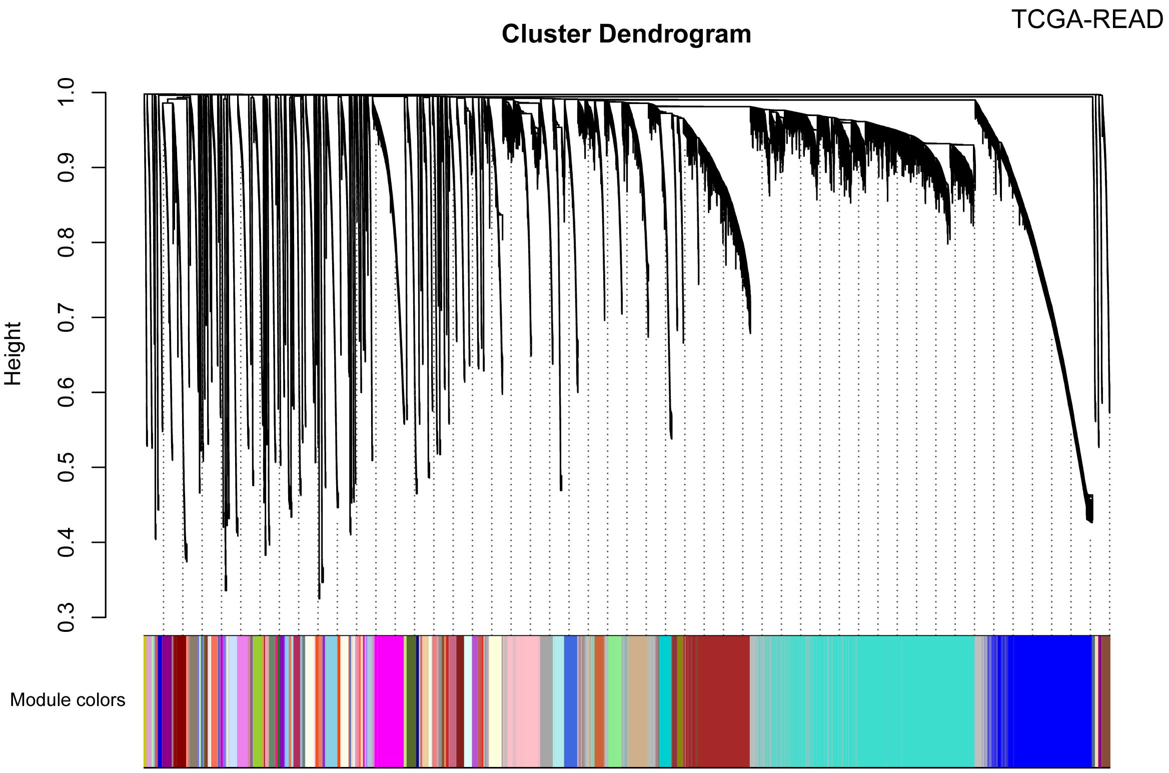

Supplementary Figure S4.24. Dendrogram of weighted gene co-expression network and module colors.

The genes clustered on the topological overlap matrix (TOM) based on dissimilarity, formed a branch-like shape, and the modules of those genes with high inter-connectivity clustered at the same module. The genes of grey module color are not assigned to any modules. The name of TCGA dataset is located to the right top corner.

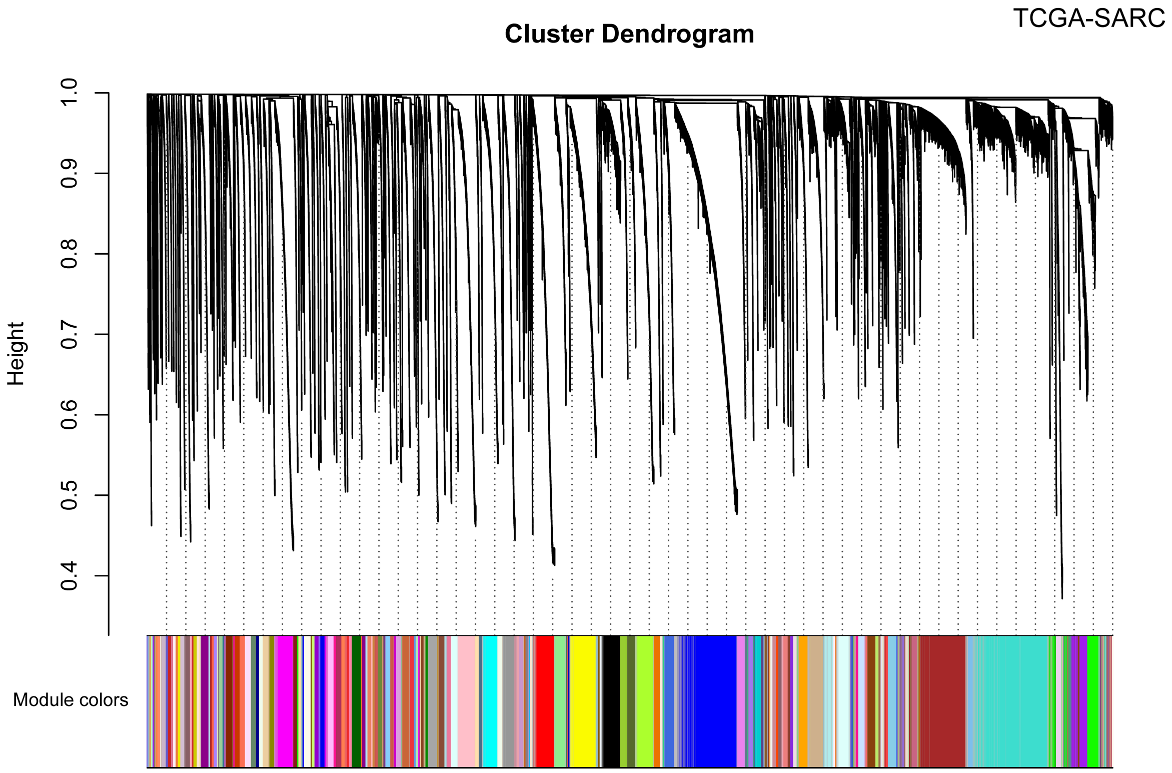

Supplementary Figure S4.25. Dendrogram of weighted gene co-expression network and module colors.

The genes clustered on the topological overlap matrix (TOM) based on dissimilarity, formed a branch-like shape, and the modules of those genes with high inter-connectivity clustered at the same module. The genes of grey module color are not assigned to any modules. The name of TCGA dataset is located to the right top corner.

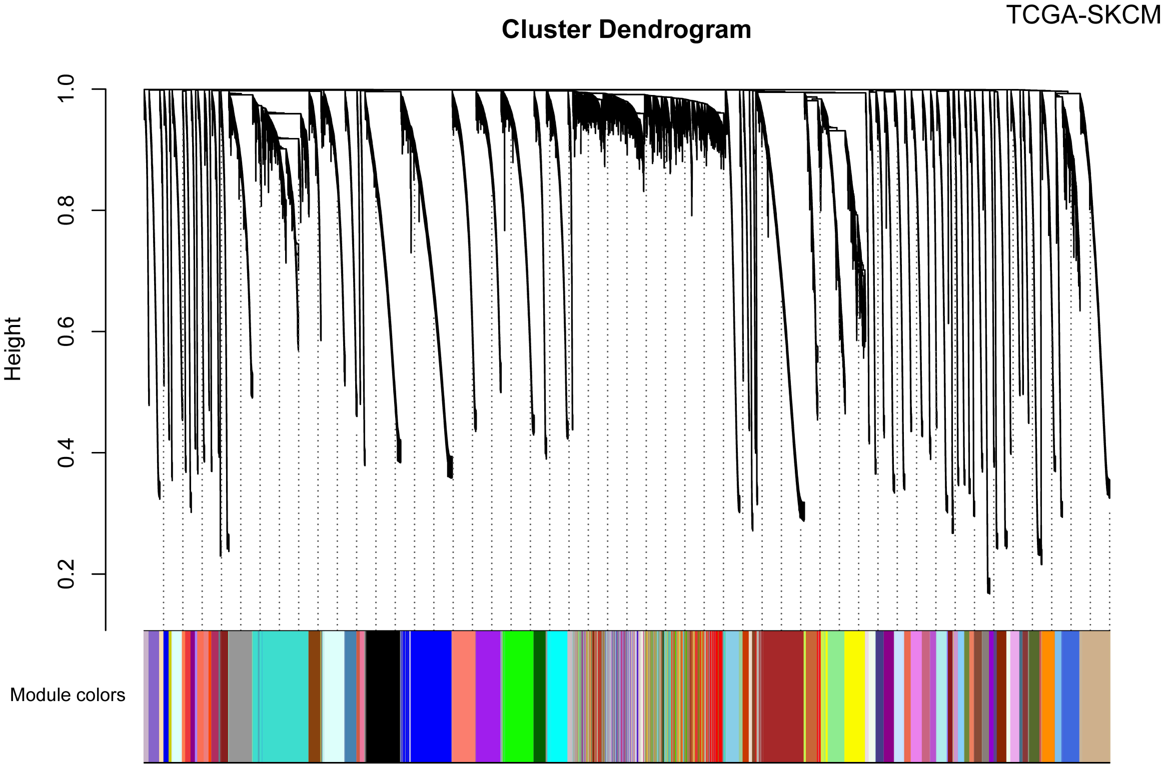

Supplementary Figure S4.26. Dendrogram of weighted gene co-expression network and module colors.

The genes clustered on the topological overlap matrix (TOM) based on dissimilarity, formed a branch-like shape, and the modules of those genes with high inter-connectivity clustered at the same module. The genes of grey module color are not assigned to any modules. The name of TCGA dataset is located to the right top corner.

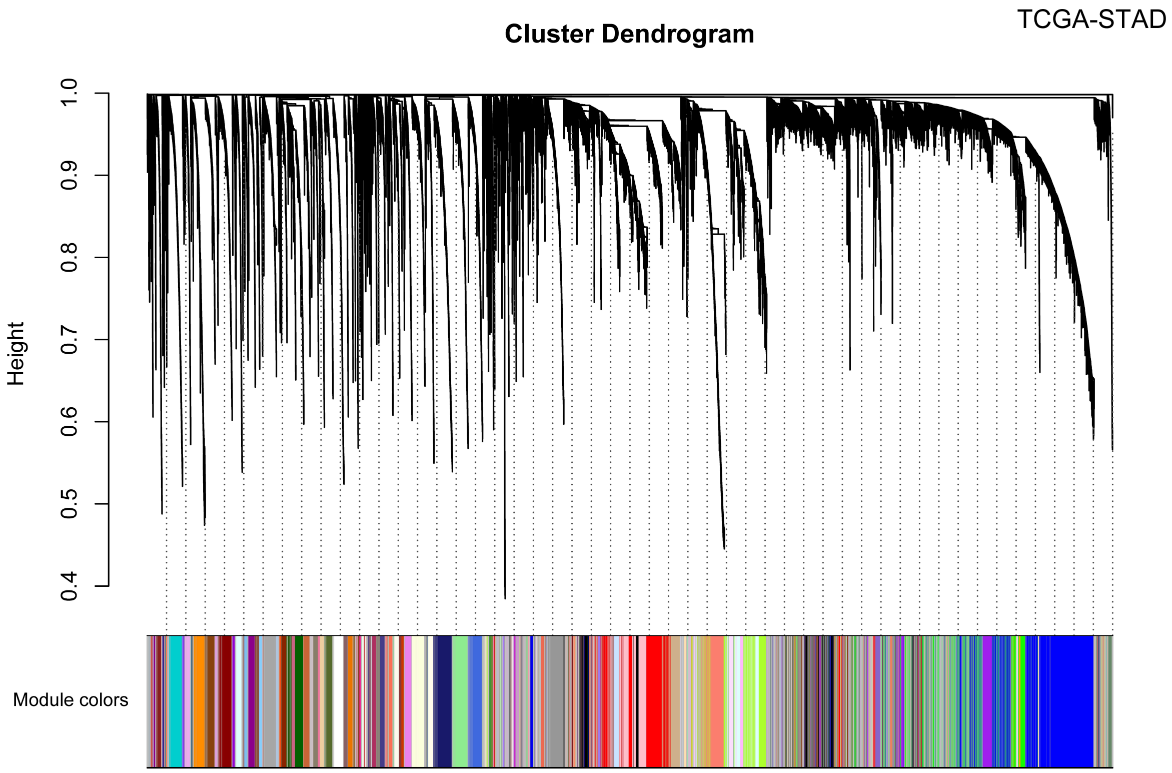

Supplementary Figure S4.27. Dendrogram of weighted gene co-expression network and module colors.

The genes clustered on the topological overlap matrix (TOM) based on dissimilarity, formed a branch-like shape, and the modules of those genes with high inter-connectivity clustered at the same module. The genes of grey module color are not assigned to any modules. The name of TCGA dataset is located to the right top corner.

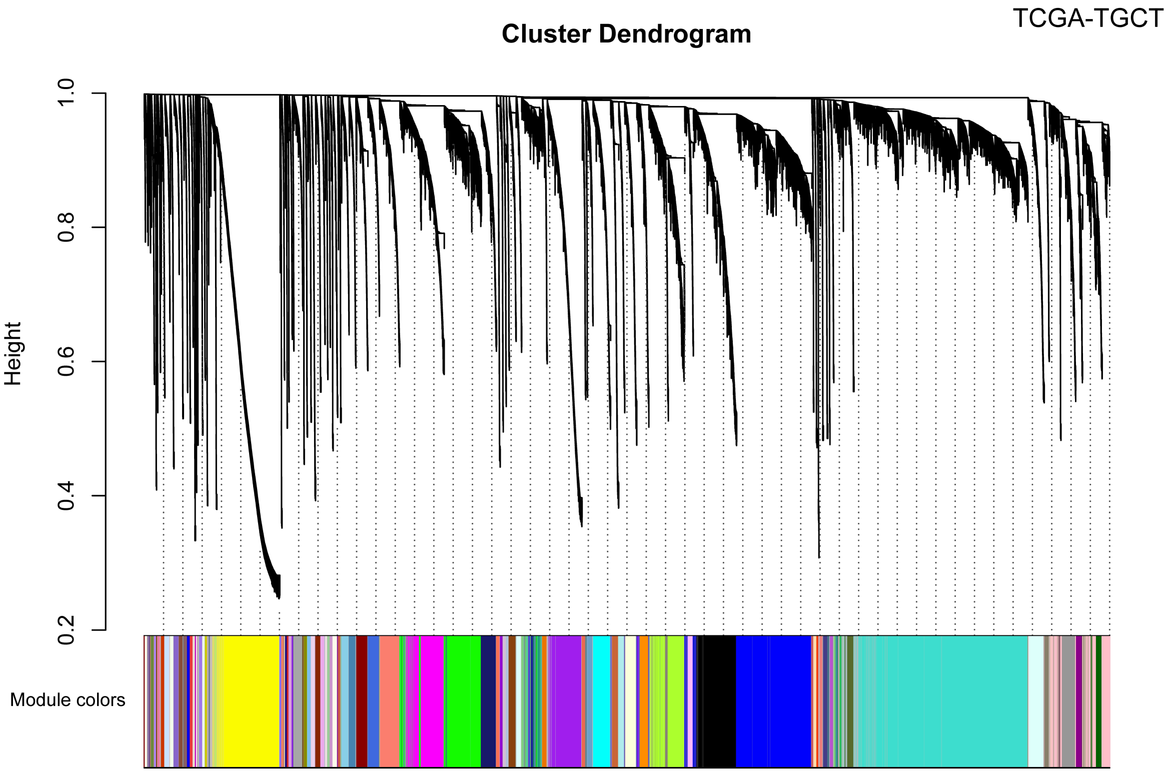

Supplementary Figure S4.28. Dendrogram of weighted gene co-expression network and module colors.

The genes clustered on the topological overlap matrix (TOM) based on dissimilarity, formed a branch-like shape, and the modules of those genes with high inter-connectivity clustered at the same module. The genes of grey module color are not assigned to any modules. The name of TCGA dataset is located to the right top corner.

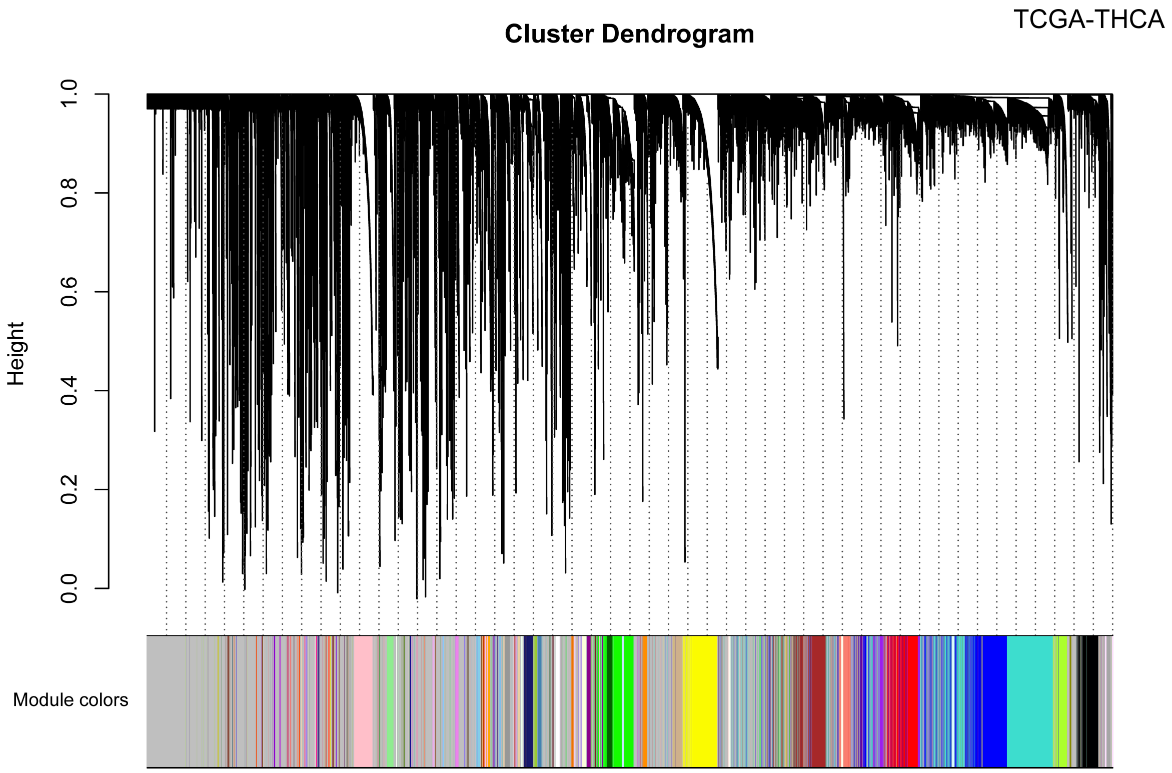

Supplementary Figure S4.29. Dendrogram of weighted gene co-expression network and module colors.

The genes clustered on the topological overlap matrix (TOM) based on dissimilarity, formed a branch-like shape, and the modules of those genes with high inter-connectivity clustered at the same module. The genes of grey module color are not assigned to any modules. The name of TCGA dataset is located to the right top corner.

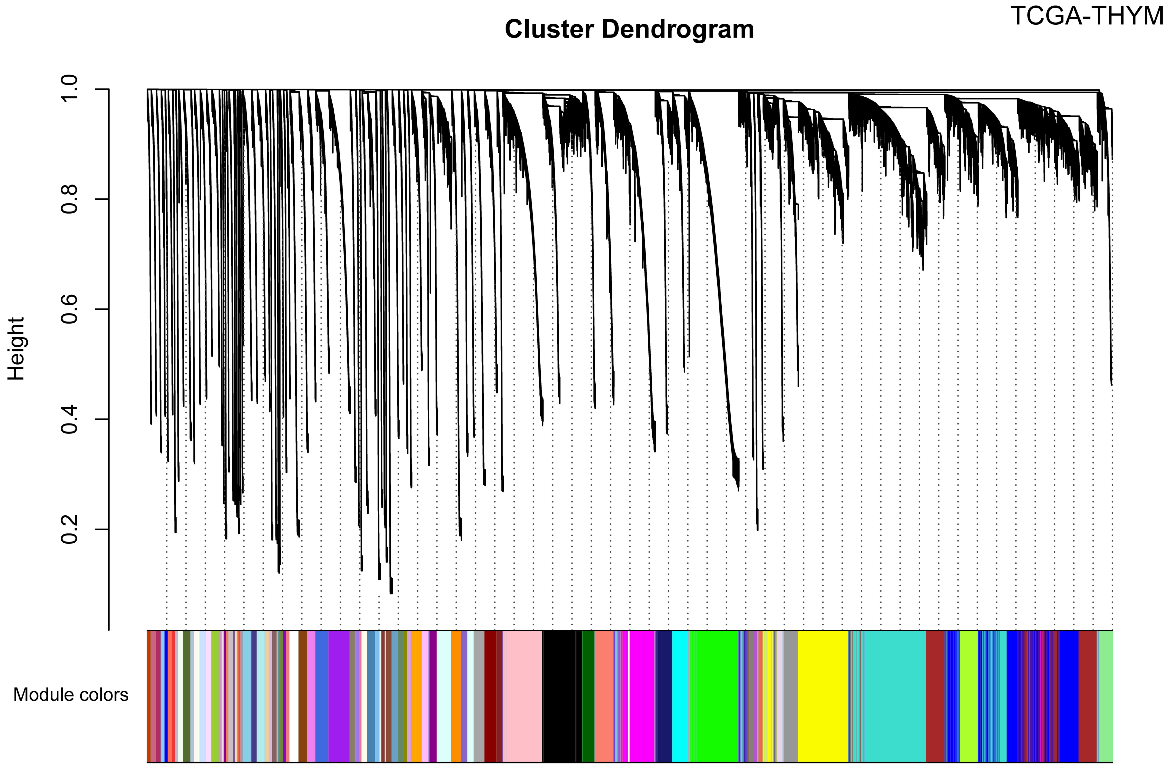

Supplementary Figure S4.30. Dendrogram of weighted gene co-expression network and module colors.

The genes clustered on the topological overlap matrix (TOM) based on dissimilarity, formed a branch-like shape, and the modules of those genes with high inter-connectivity clustered at the same module. The genes of grey module color are not assigned to any modules. The name of TCGA dataset is located to the right top corner.

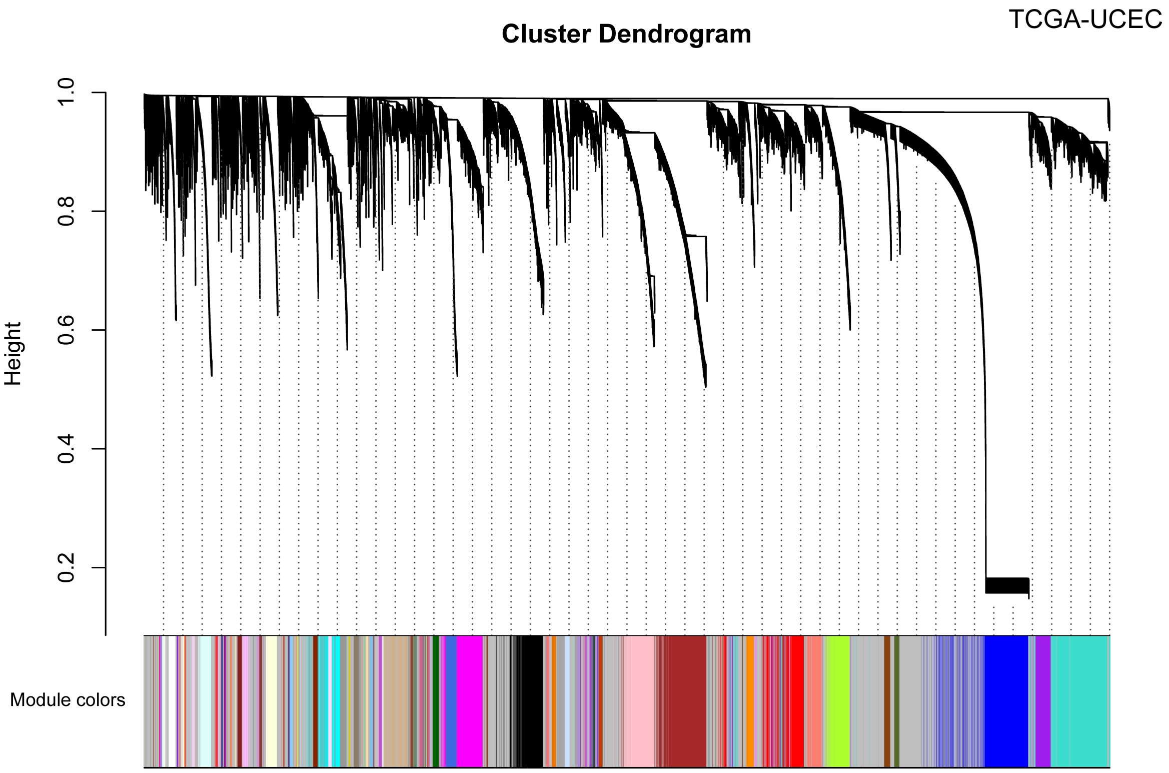

Supplementary Figure S4.31. Dendrogram of weighted gene co-expression network and module colors.

The genes clustered on the topological overlap matrix (TOM) based on dissimilarity, formed a branch-like shape, and the modules of those genes with high inter-connectivity clustered at the same module. The genes of grey module color are not assigned to any modules. The name of TCGA dataset is located to the right top corner.

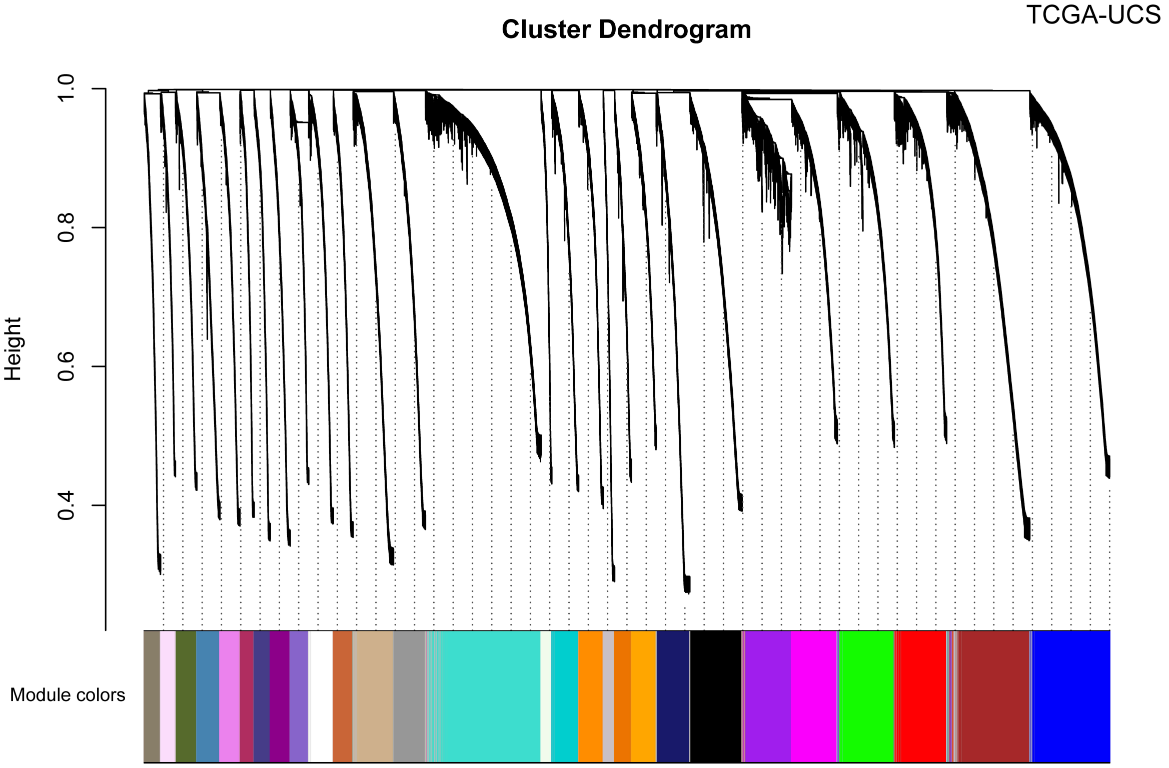

Supplementary Figure S4.32. Dendrogram of weighted gene co-expression network and module colors.

The genes clustered on the topological overlap matrix (TOM) based on dissimilarity, formed a branch-like shape, and the modules of those genes with high inter-connectivity clustered at the same module. The genes of grey module color are not assigned to any modules. The name of TCGA dataset is located to the right top corner.

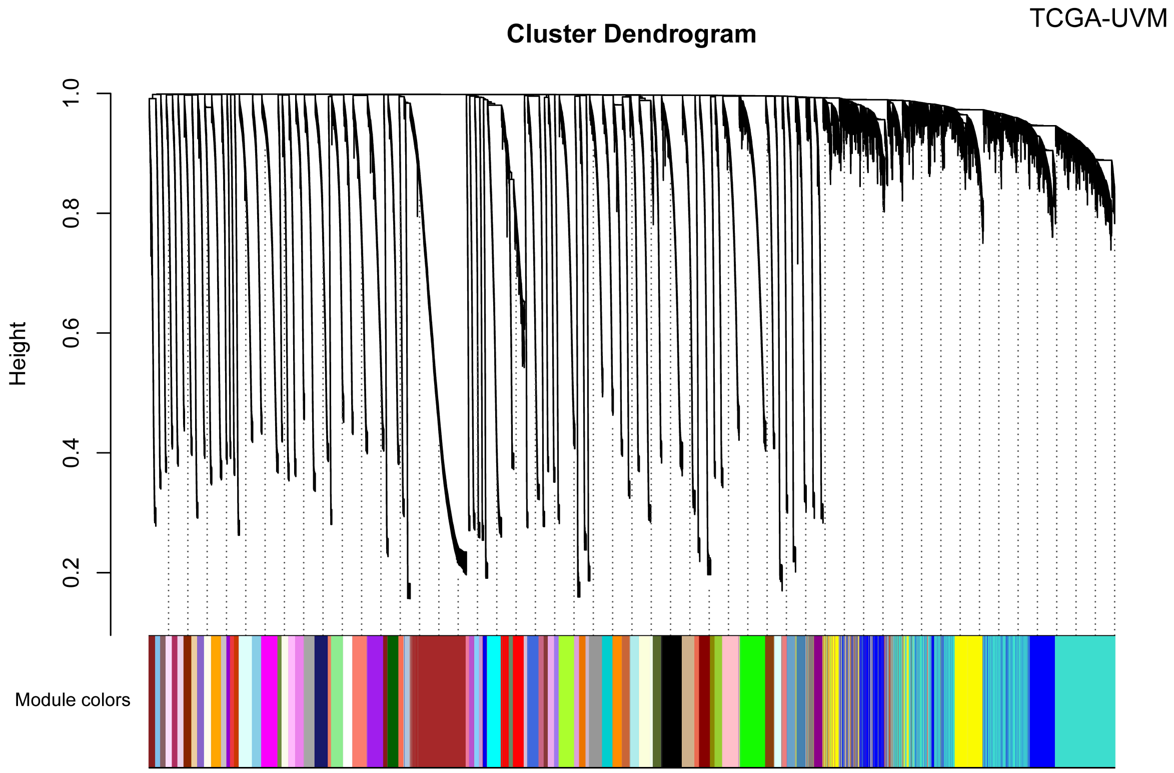

Supplementary Figure S4.33. Dendrogram of weighted gene co-expression network and module colors.

The genes clustered on the topological overlap matrix (TOM) based on dissimilarity, formed a branch-like shape, and the modules of those genes with high inter-connectivity clustered at the same module. The genes of grey module color are not assigned to any modules. The name of TCGA dataset is located to the right top corner.

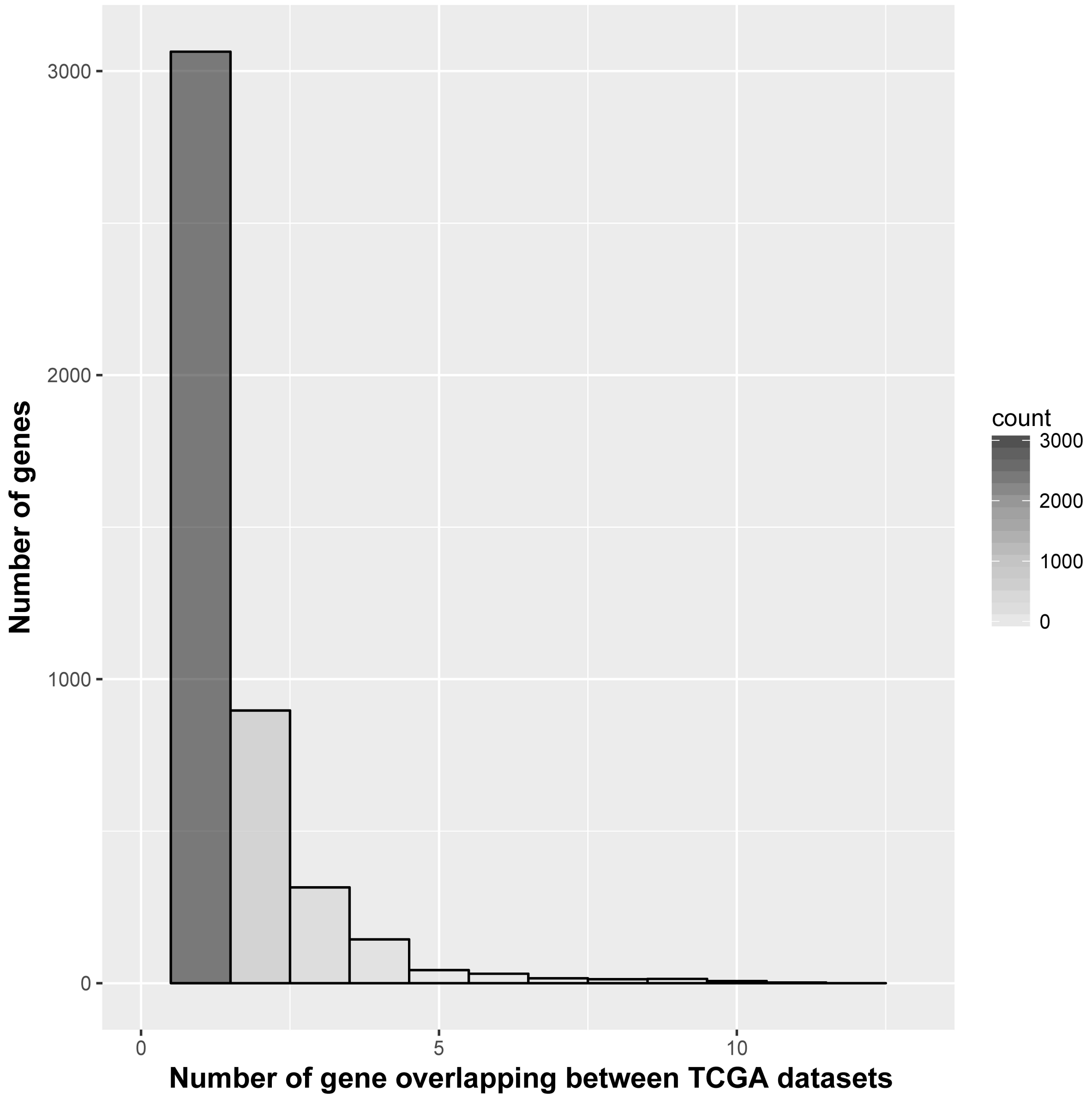

Supplementary Figure S5. Gene overlapping between TCGA datasets.

The x-axis shows the number of TCGA datasets that PSGs belong to, and y-axis shows the number of PSGs. The PSGs overlapped between up to 11 TCGA datasets. From left bar, 3064, 897, 315, 144, 43, 31, 16, 13, 14, 7, and 2 PSGs are presented.

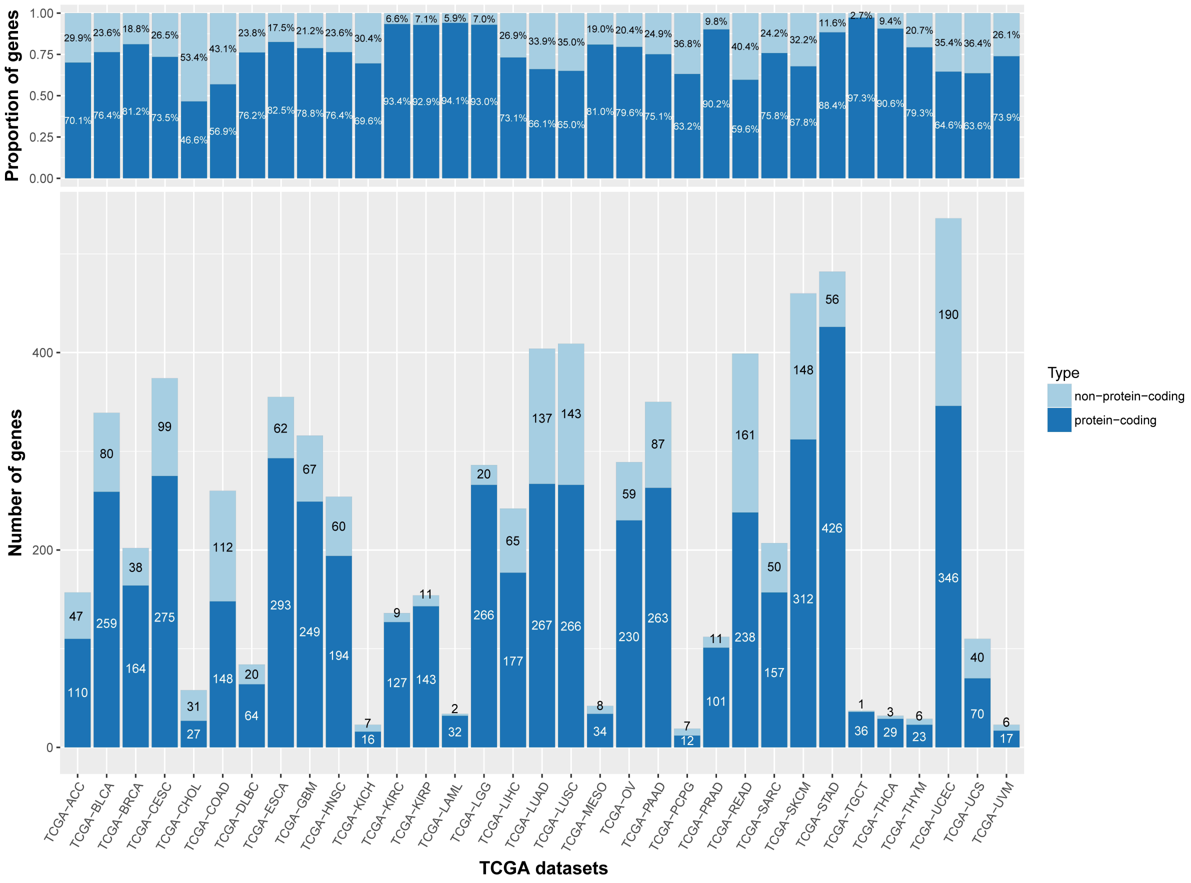

Supplementary Figure S6. Number of protein-coding and non-coding genes in pan-cancer-wide selected genes.

The lower panel shows the number of protein-coding and non-protein-coding PSGs and upper panel shows the proportion of them for each TCGA dataset. Dark blue presents protein-coding PSGs and light blue shows non-protein-coding PSGs.

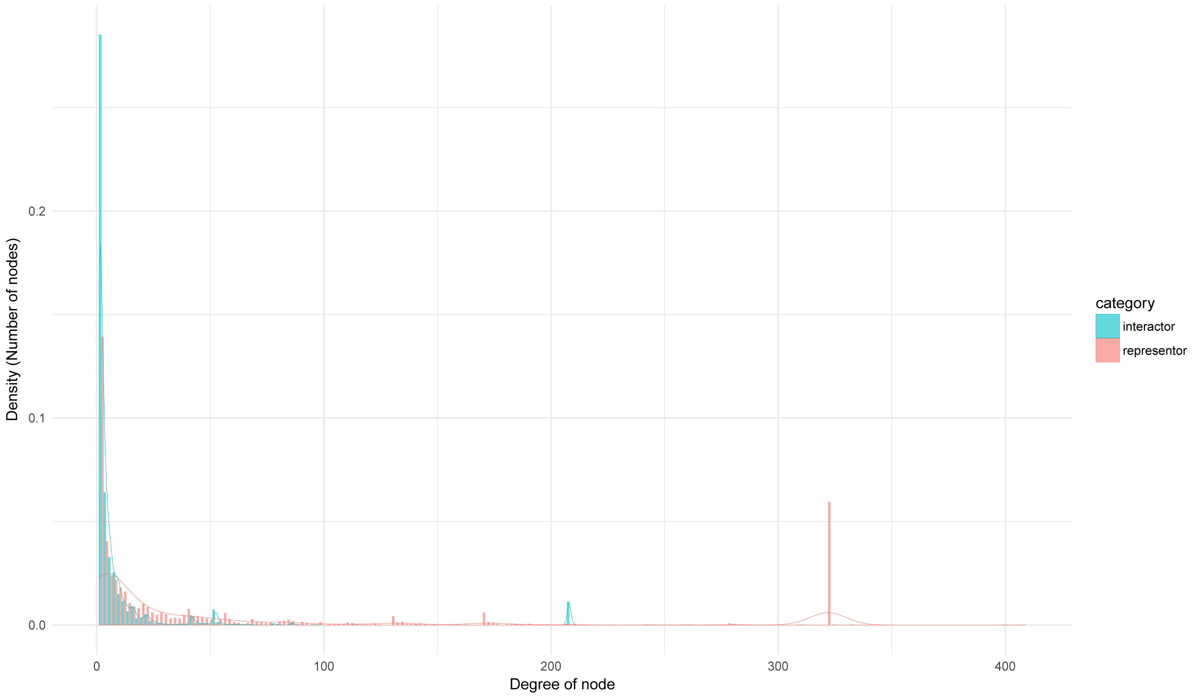

Supplementary Figure S7. Distribution of PPIs for protein-coding PSGs and genes of interaction.

The PSGs that had PPI information were designated as 'representor' (light red), and genes that interacted with representor were designated as 'interactor' (light blue). Density of node (y-axis) against degree of node (x-axis) were plotted.

Cluster 1

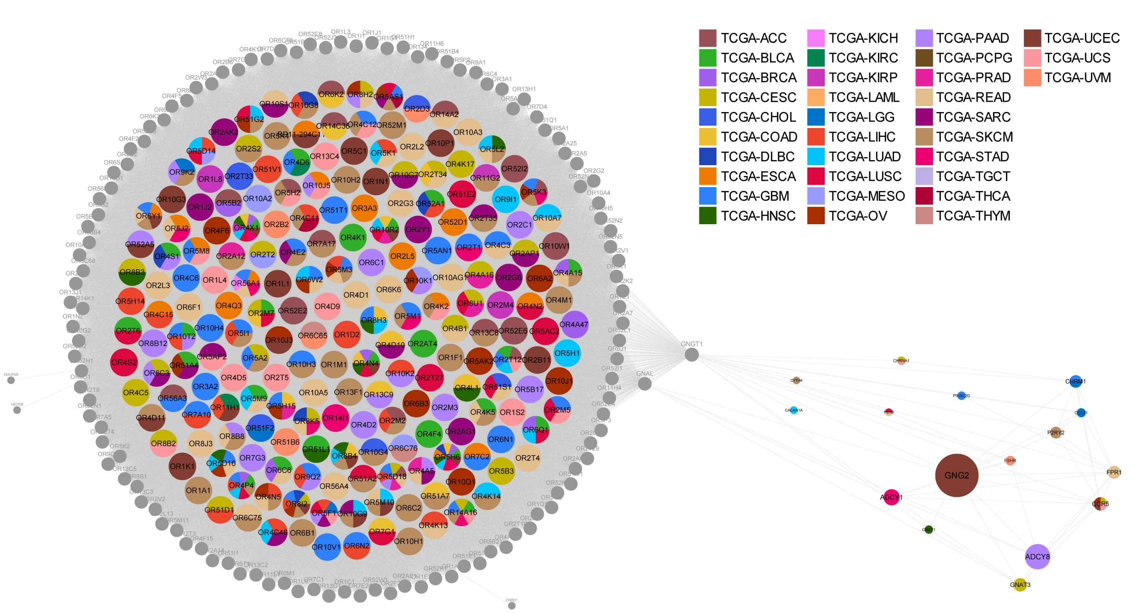

Supplementary Figure S8.1. Subnetwork of single depth PPI network of protein-coding PSGs.

PSGs that have PPI information (representors) are coloured differently according to the TCGA dataset to which they belong. The colour indicator for the TCGA datasets is located to the top right corner. PSGs that belong to more than one TCGA dataset are presented like pie charts. The genes that interact with PSGs (interactors) are presented as grey colour. The name of TCGA dataset is on top of figure.

Cluster 2

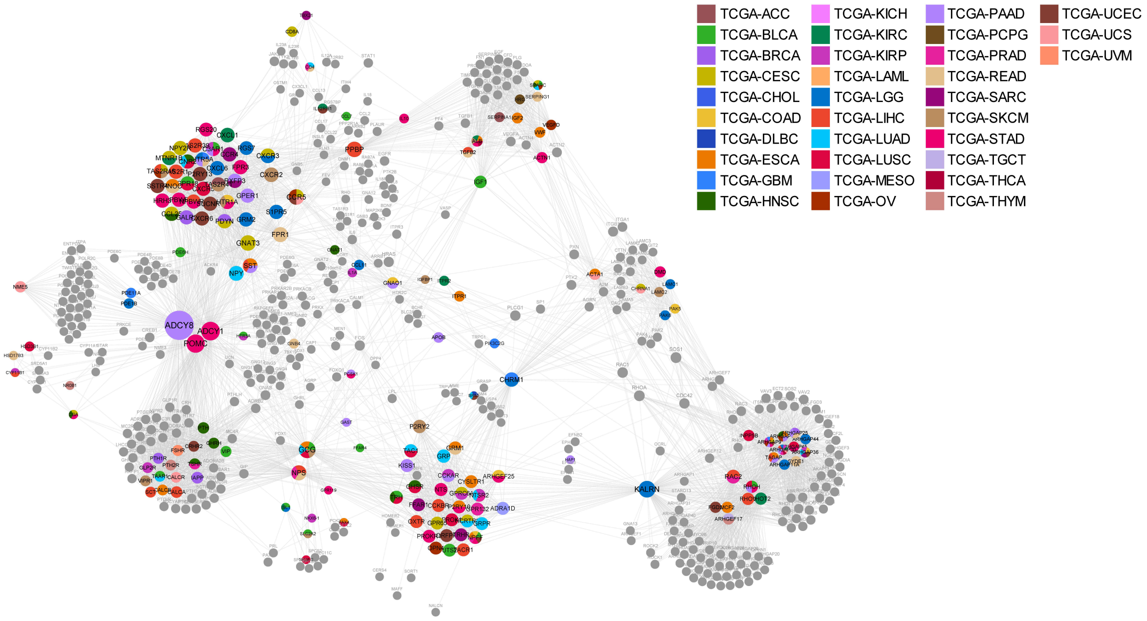

Supplementary Figure S8.2. Subnetwork of single depth PPI network of protein-coding PSGs.

PSGs that have PPI information (representors) are coloured differently according to the TCGA dataset to which they belong. The colour indicator for the TCGA datasets is located to the top right corner. PSGs that belong to more than one TCGA dataset are presented like pie charts. The genes that interact with PSGs (interactors) are presented as grey colour. The name of TCGA dataset is on top of figure.

Cluster 3

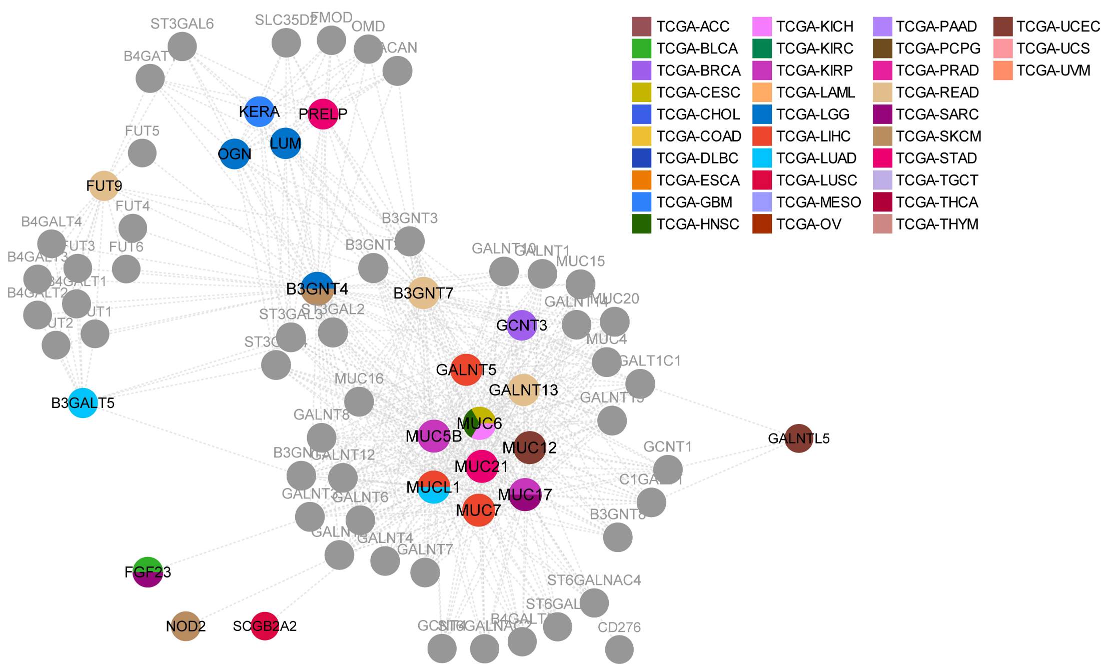

Supplementary Figure S8.3. Subnetwork of single depth PPI network of protein-coding PSGs.

PSGs that have PPI information (representors) are coloured differently according to the TCGA dataset to which they belong. The colour indicator for the TCGA datasets is located to the top right corner. PSGs that belong to more than one TCGA dataset are presented like pie charts. The genes that interact with PSGs (interactors) are presented as grey colour. The name of TCGA dataset is on top of figure.

Cluster 4

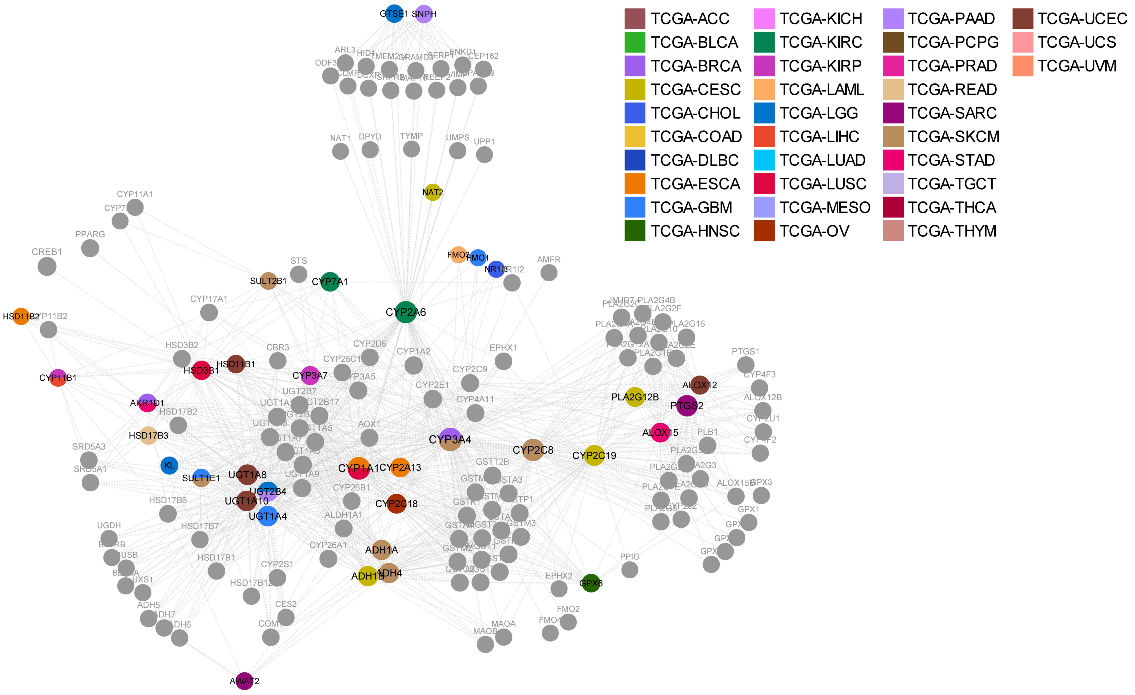

Supplementary Figure S8.4. Subnetwork of single depth PPI network of protein-coding PSGs.

PSGs that have PPI information (representors) are coloured differently according to the TCGA dataset to which they belong. The colour indicator for the TCGA datasets is located to the top right corner. PSGs that belong to more than one TCGA dataset are presented like pie charts. The genes that interact with PSGs (interactors) are presented as grey colour. The name of TCGA dataset is on top of figure.

Cluster 5

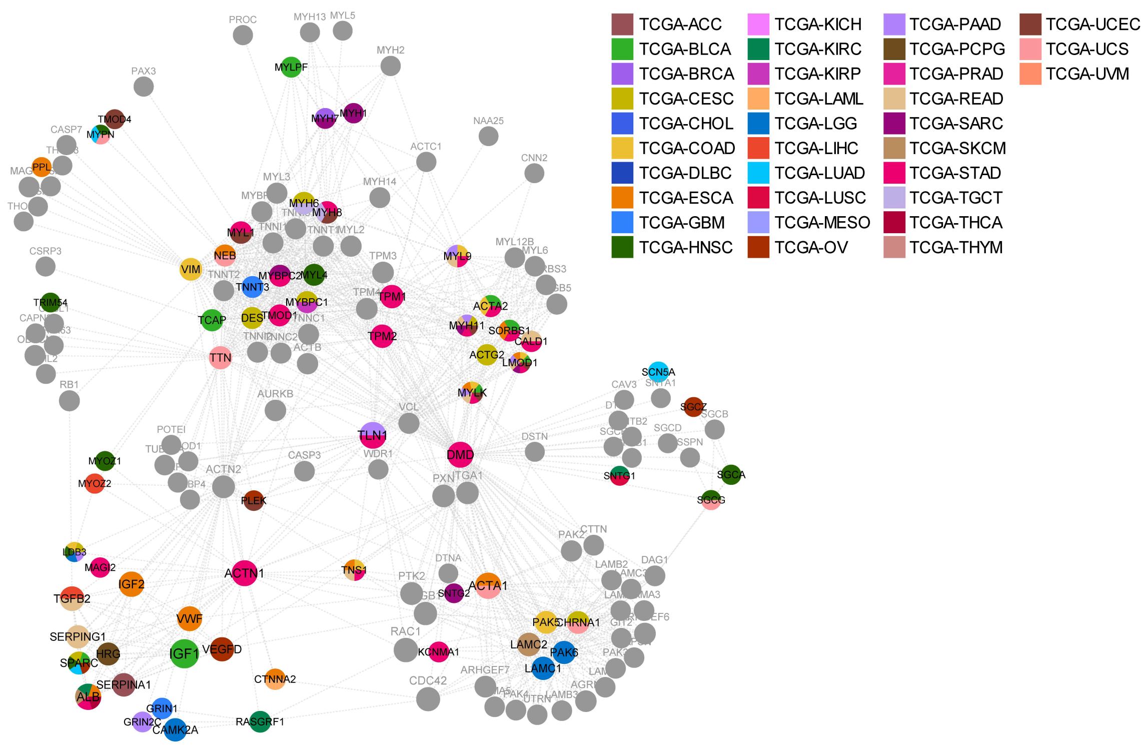

Supplementary Figure S8.5. Subnetwork of single depth PPI network of protein-coding PSGs.

PSGs that have PPI information (representors) are coloured differently according to the TCGA dataset to which they belong. The colour indicator for the TCGA datasets is located to the top right corner. PSGs that belong to more than one TCGA dataset are presented like pie charts. The genes that interact with PSGs (interactors) are presented as grey colour. The name of TCGA dataset is on top of figure.

Cluster 6

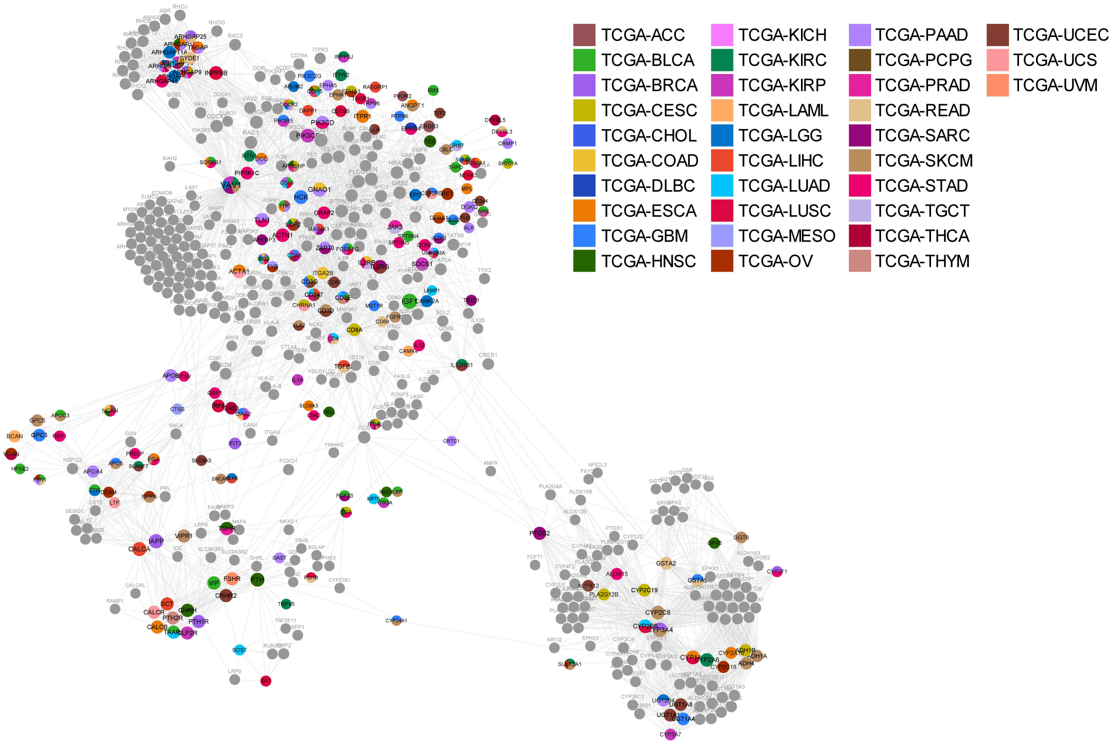

Supplementary Figure S8.6. Subnetwork of single depth PPI network of protein-coding PSGs.

PSGs that have PPI information (representors) are coloured differently according to the TCGA dataset to which they belong. The colour indicator for the TCGA datasets is located to the top right corner. PSGs that belong to more than one TCGA dataset are presented like pie charts. The genes that interact with PSGs (interactors) are presented as grey colour. The name of TCGA dataset is on top of figure.

Cluster 7

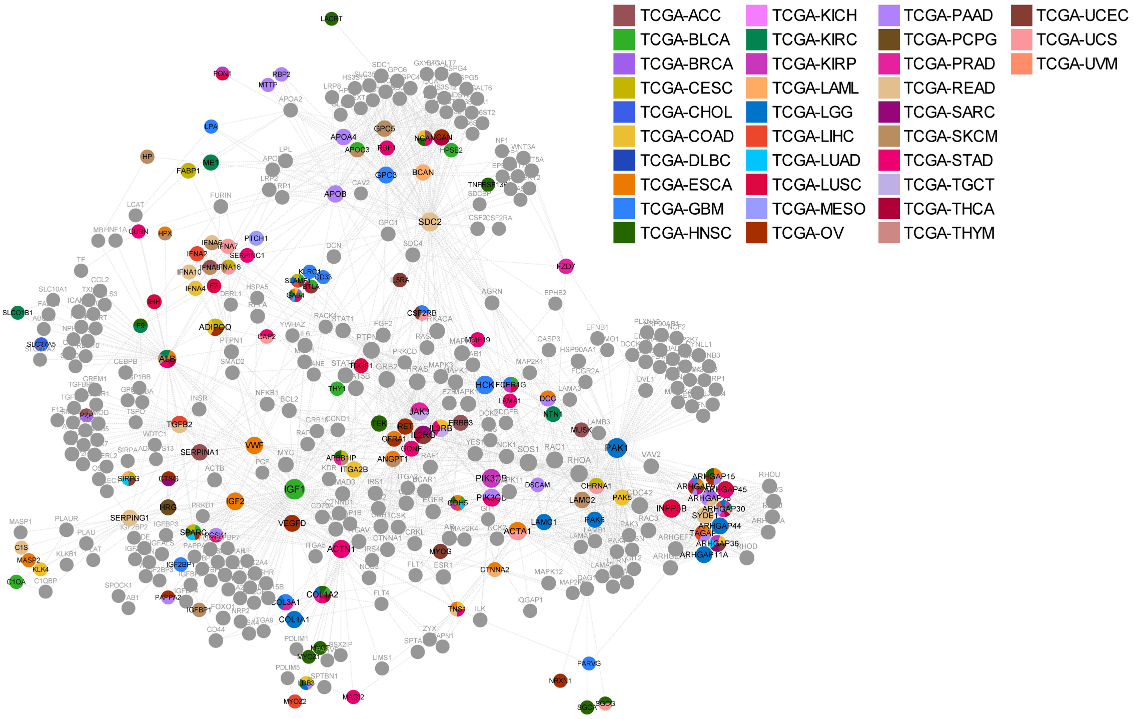

Supplementary Figure S8.7. Subnetwork of single depth PPI network of protein-coding PSGs.

PSGs that have PPI information (representors) are coloured differently according to the TCGA dataset to which they belong. The colour indicator for the TCGA datasets is located to the top right corner. PSGs that belong to more than one TCGA dataset are presented like pie charts. The genes that interact with PSGs (interactors) are presented as grey colour. The name of TCGA dataset is on top of figure.

Cluster 8

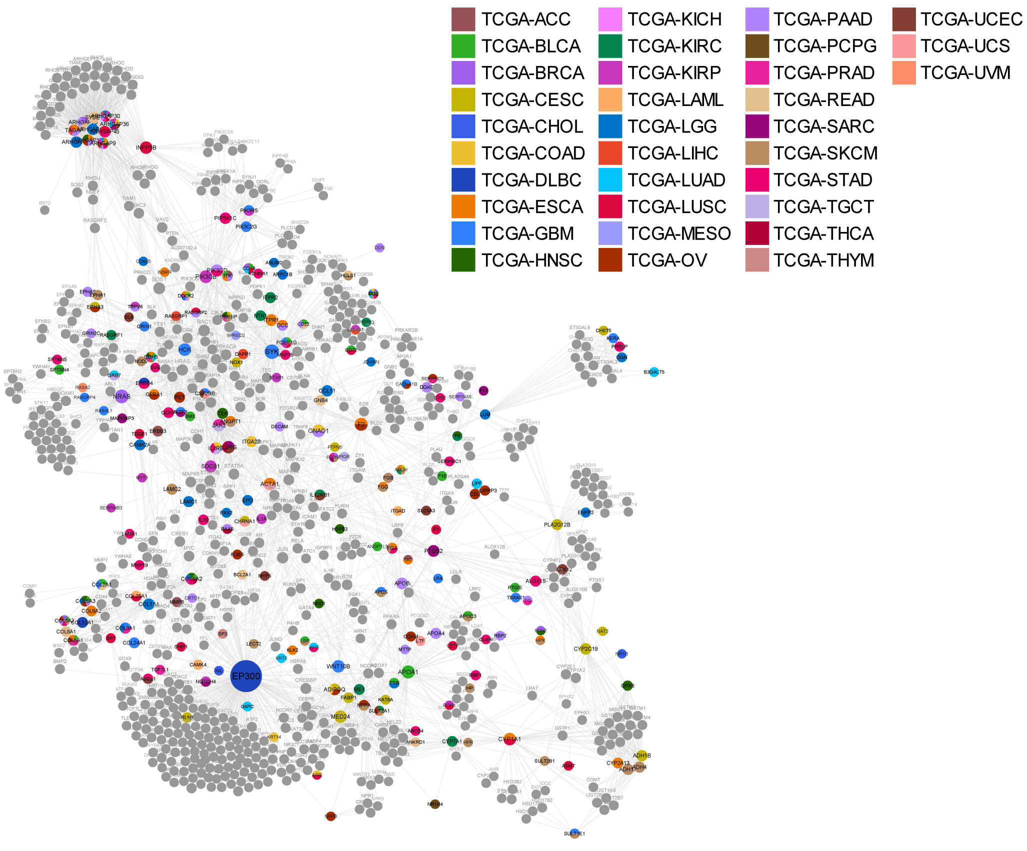

Supplementary Figure S8.8. Subnetwork of single depth PPI network of protein-coding PSGs.

PSGs that have PPI information (representors) are coloured differently according to the TCGA dataset to which they belong. The colour indicator for the TCGA datasets is located to the top right corner. PSGs that belong to more than one TCGA dataset are presented like pie charts. The genes that interact with PSGs (interactors) are presented as grey colour. The name of TCGA dataset is on top of figure.

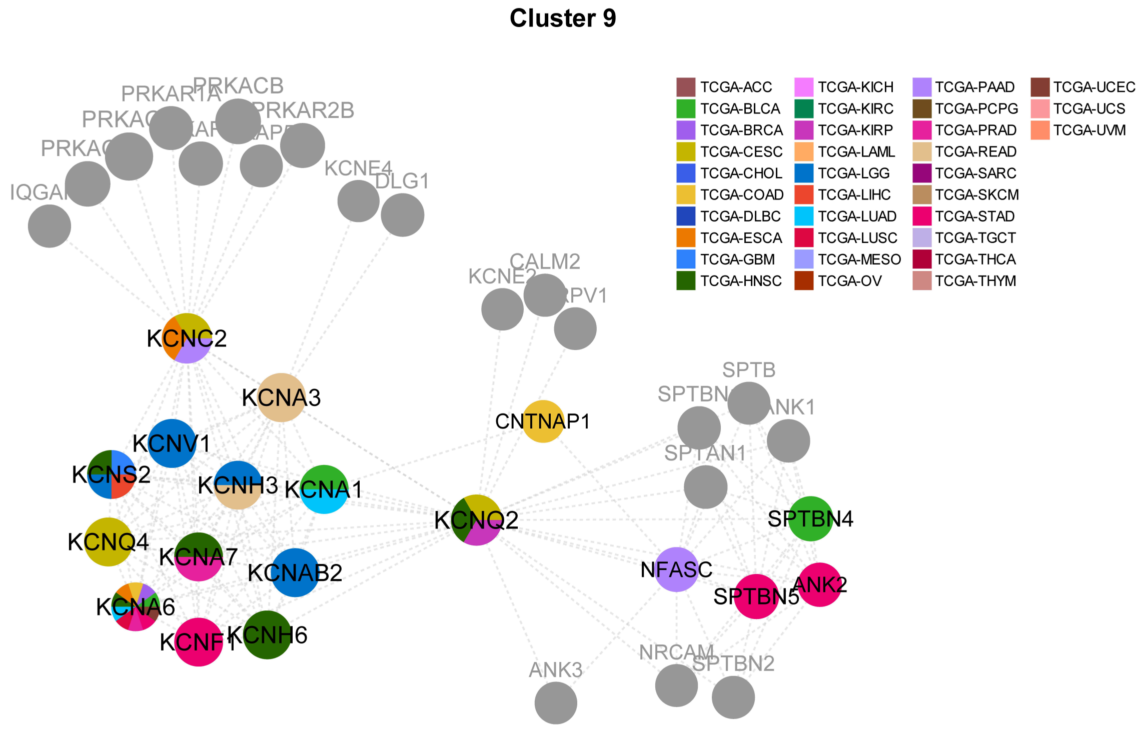

Supplementary Figure S8.9. Subnetwork of single depth PPI network of protein-coding PSGs.

PSGs that have PPI information (representors) are coloured differently according to the TCGA dataset to which they belong. The colour indicator for the TCGA datasets is located to the top right corner. PSGs that belong to more than one TCGA dataset are presented like pie charts. The genes that interact with PSGs (interactors) are presented as grey colour. The name of TCGA dataset is on top of figure.

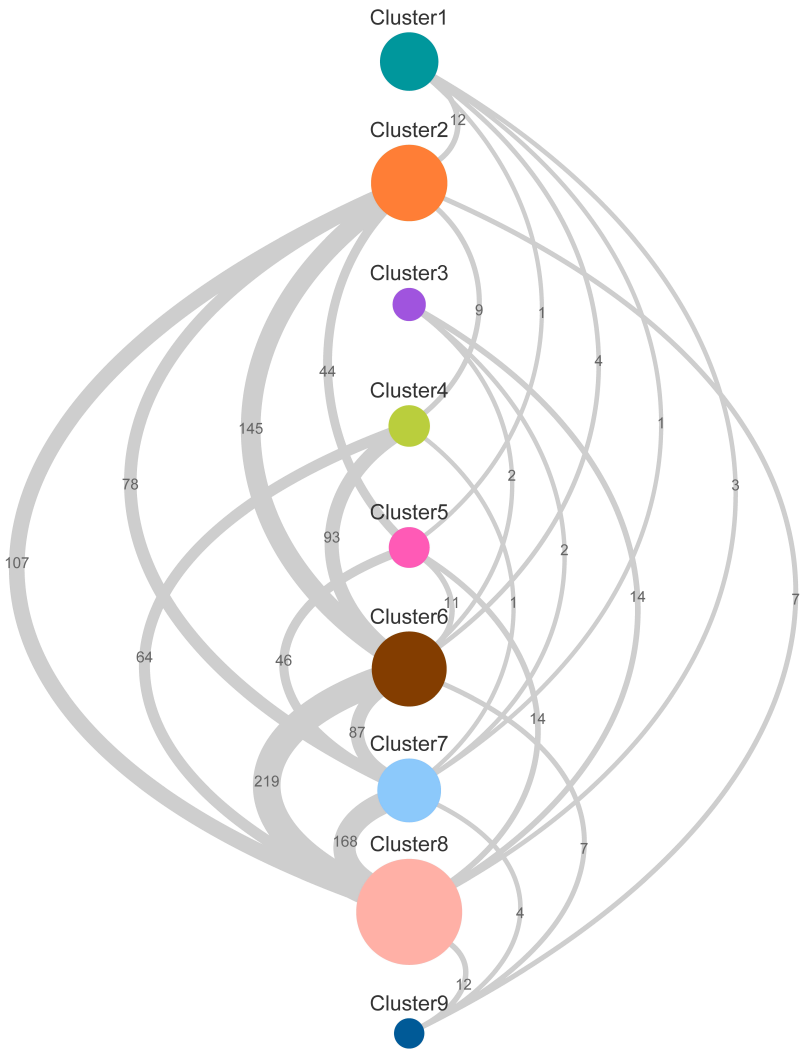

Supplementary Figure S9. The number of shared genes between subnetworks.

The crescent-shaped lines show the number of genes that shared by clusters. Line width and size of cluster reflect the number of genes.

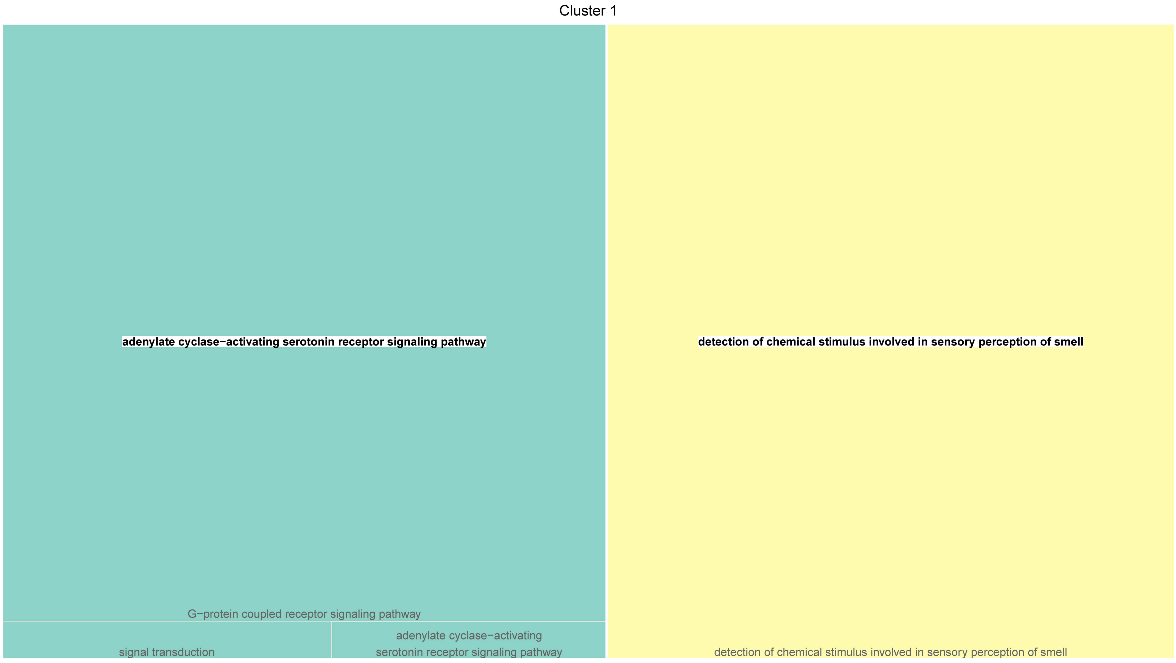

Supplementary Figure S10.1. GO term summarisation of subnetwork of PPI network of protein-coding PSGs.

GO term summary on gene clusters of subnetworks are presented as a tree map. The name of subnetwork is on top of the tree map, and size of blocks shows the significance of enrichment of GO term. Similar GO terms were combined and grouped into a large block of the same colour. The term of most uniqueness in the large block is shown on the white box and located at the centre of the block.

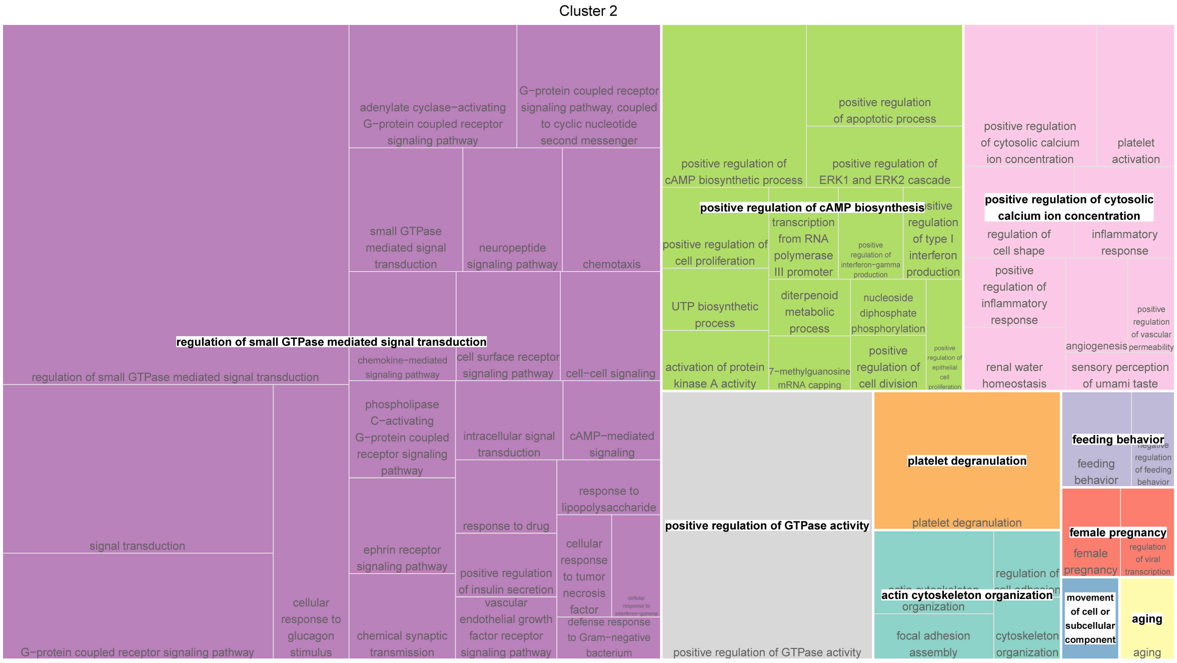

Supplementary Figure S10.2. GO term summarisation of subnetwork of PPI network of protein-coding PSGs.

GO term summary on gene clusters of subnetworks are presented as a tree map. The name of subnetwork is on top of the tree map, and size of blocks shows the significance of enrichment of GO term. Similar GO terms were combined and grouped into a large block of the same colour. The term of most uniqueness in the large block is shown on the white box and located at the centre of the block.

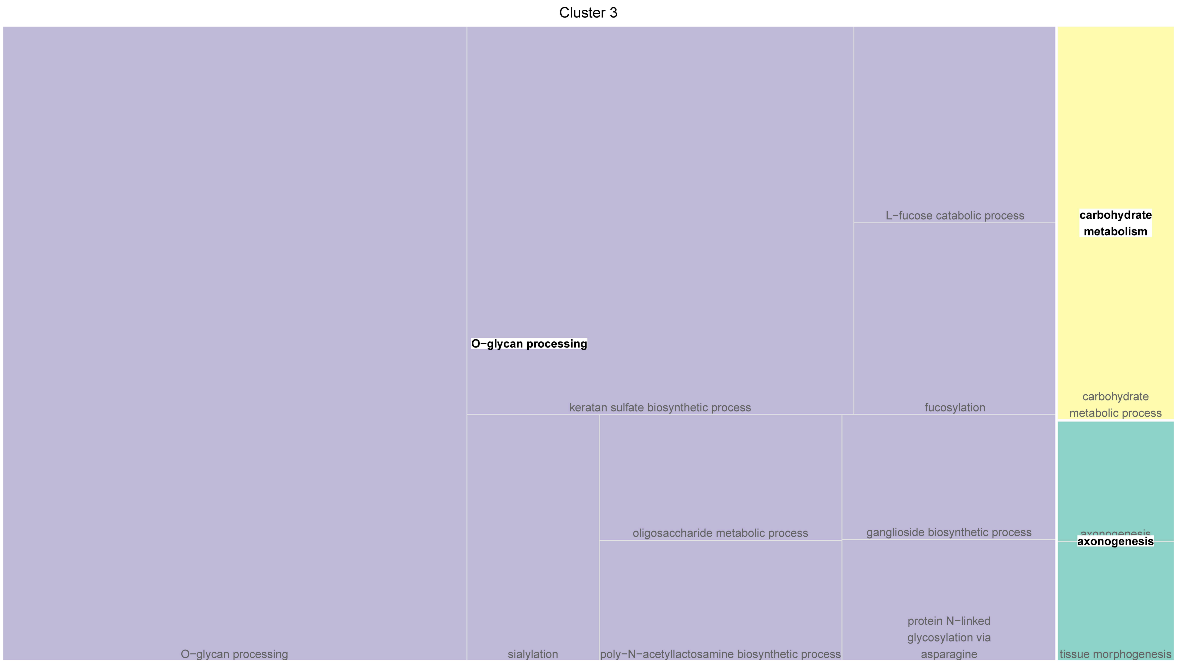

Supplementary Figure S10.3. GO term summarisation of subnetwork of PPI network of protein-coding PSGs.

GO term summary on gene clusters of subnetworks are presented as a tree map. The name of subnetwork is on top of the tree map, and size of blocks shows the significance of enrichment of GO term. Similar GO terms were combined and grouped into a large block of the same colour. The term of most uniqueness in the large block is shown on the white box and located at the centre of the block.

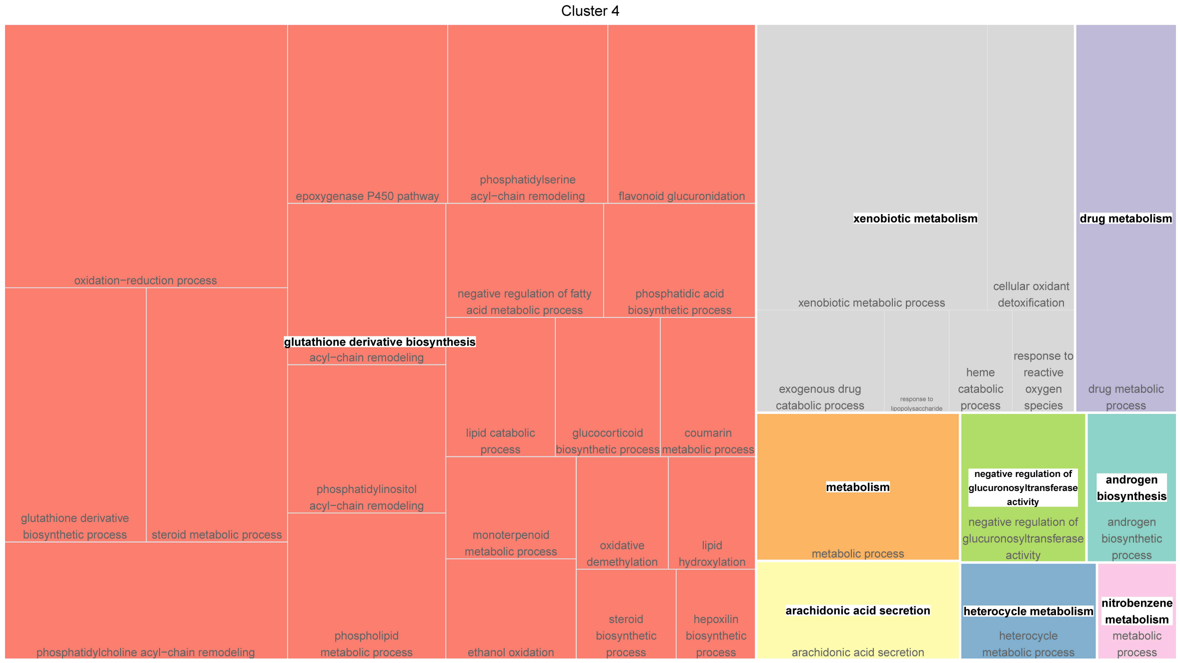

Supplementary Figure S10.4. GO term summarisation of subnetwork of PPI network of protein-coding PSGs.

GO term summary on gene clusters of subnetworks are presented as a tree map. The name of subnetwork is on top of the tree map, and size of blocks shows the significance of enrichment of GO term. Similar GO terms were combined and grouped into a large block of the same colour. The term of most uniqueness in the large block is shown on the white box and located at the centre of the block.

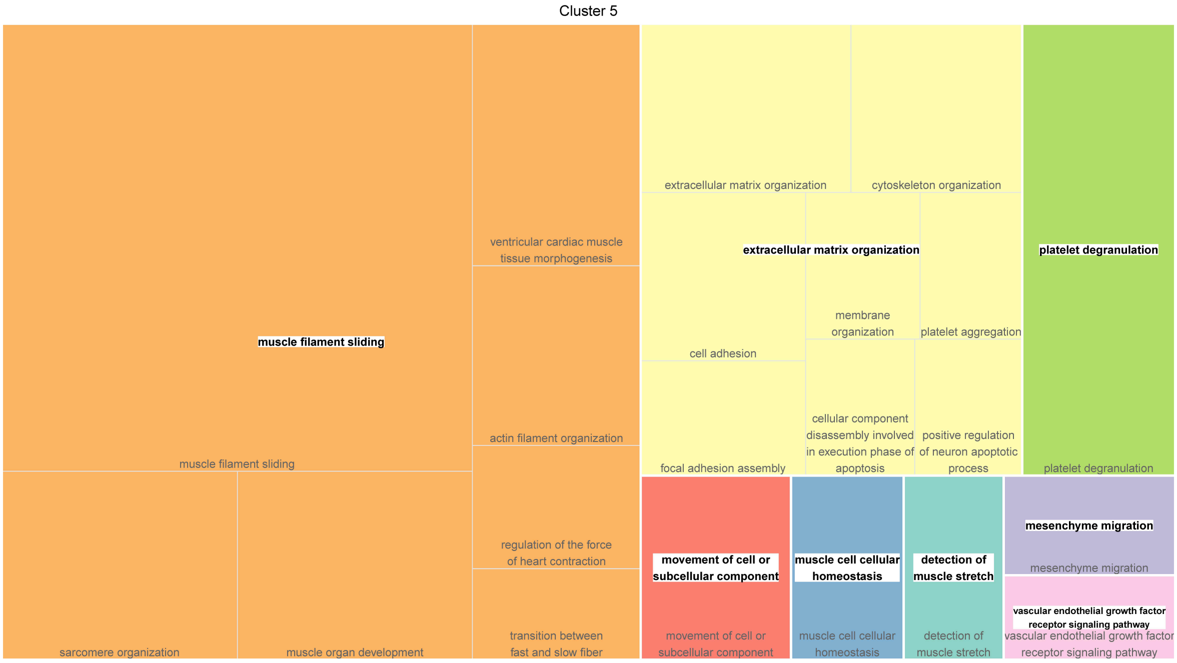

Supplementary Figure S10.5. GO term summarisation of subnetwork of PPI network of protein-coding PSGs.

GO term summary on gene clusters of subnetworks are presented as a tree map. The name of subnetwork is on top of the tree map, and size of blocks shows the significance of enrichment of GO term. Similar GO terms were combined and grouped into a large block of the same colour. The term of most uniqueness in the large block is shown on the white box and located at the centre of the block.

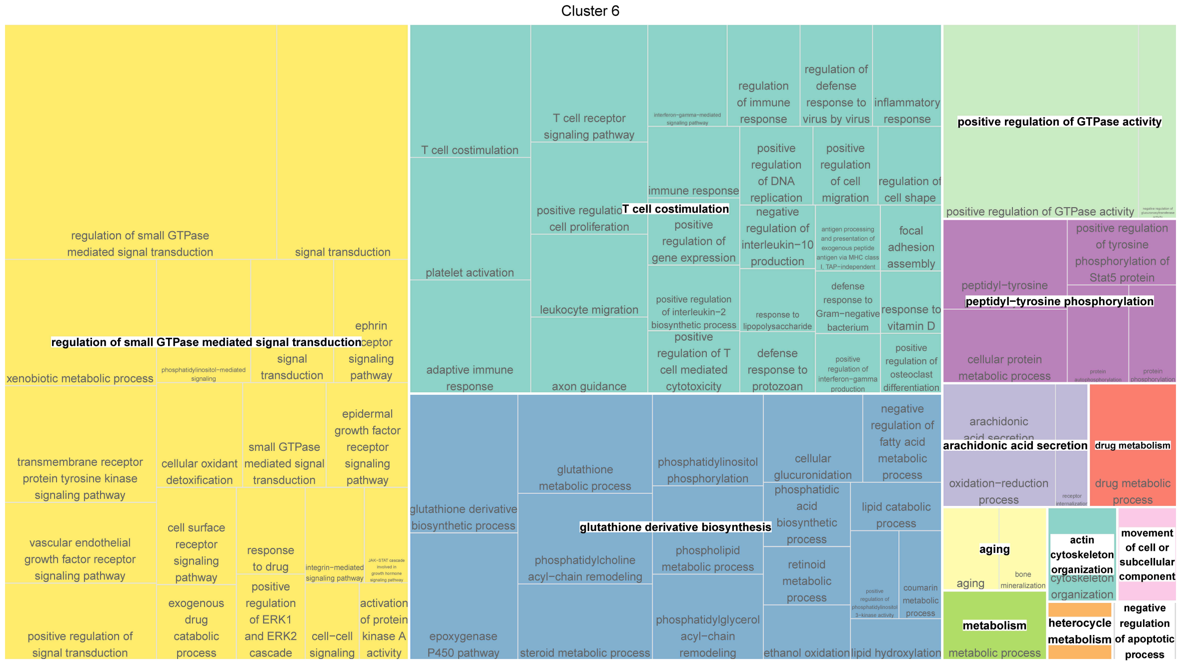

Supplementary Figure S10.6. GO term summarisation of subnetwork of PPI network of protein-coding PSGs.

GO term summary on gene clusters of subnetworks are presented as a tree map. The name of subnetwork is on top of the tree map, and size of blocks shows the significance of enrichment of GO term. Similar GO terms were combined and grouped into a large block of the same colour. The term of most uniqueness in the large block is shown on the white box and located at the centre of the block.

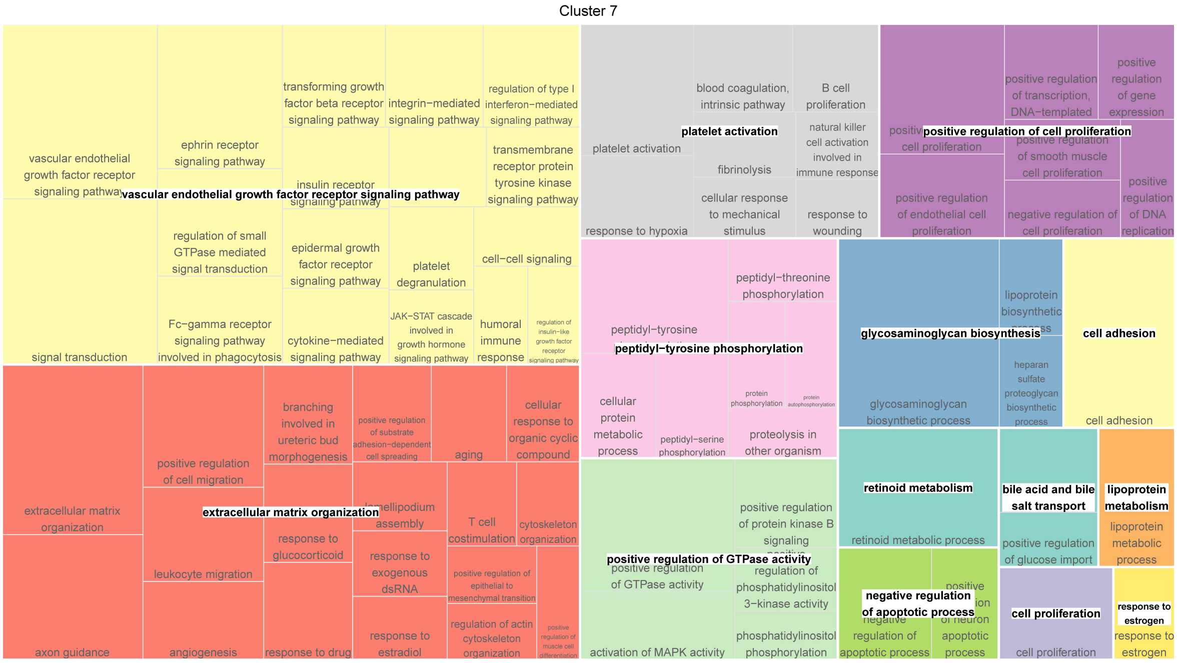

Supplementary Figure S10.7. GO term summarisation of subnetwork of PPI network of protein-coding PSGs.

GO term summary on gene clusters of subnetworks are presented as a tree map. The name of subnetwork is on top of the tree map, and size of blocks shows the significance of enrichment of GO term. Similar GO terms were combined and grouped into a large block of the same colour. The term of most uniqueness in the large block is shown on the white box and located at the centre of the block.

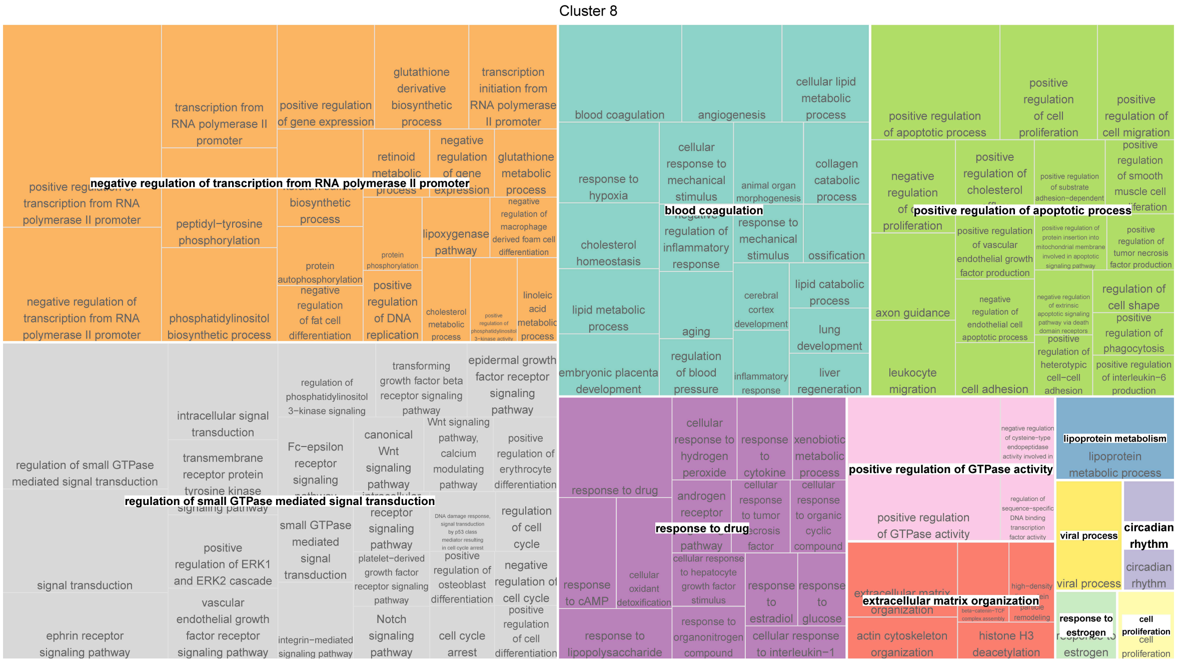

Supplementary Figure S10.8. GO term summarisation of subnetwork of PPI network of protein-coding PSGs.

GO term summary on gene clusters of subnetworks are presented as a tree map. The name of subnetwork is on top of the tree map, and size of blocks shows the significance of enrichment of GO term. Similar GO terms were combined and grouped into a large block of the same colour. The term of most uniqueness in the large block is shown on the white box and located at the centre of the block.

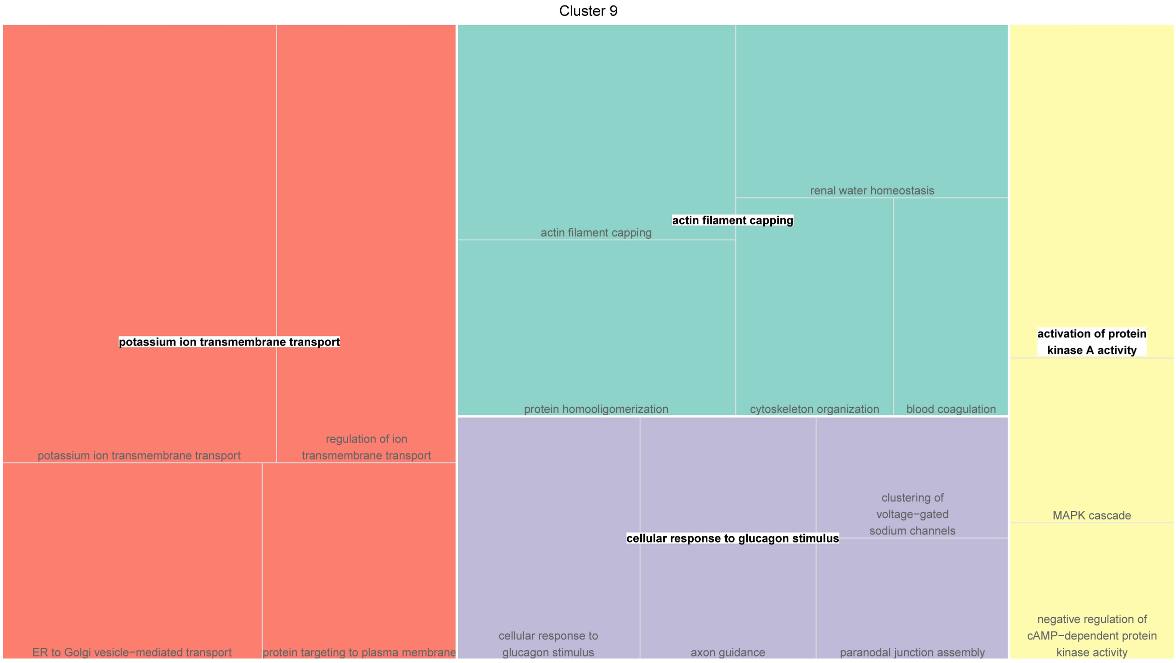

Supplementary Figure S10.9. GO term summarisation of subnetwork of PPI network of protein-coding PSGs.

GO term summary on gene clusters of subnetworks are presented as a tree map. The name of subnetwork is on top of the tree map, and size of blocks shows the significance of enrichment of GO term. Similar GO terms were combined and grouped into a large block of the same colour. The term of most uniqueness in the large block is shown on the white box and located at the centre of the block.

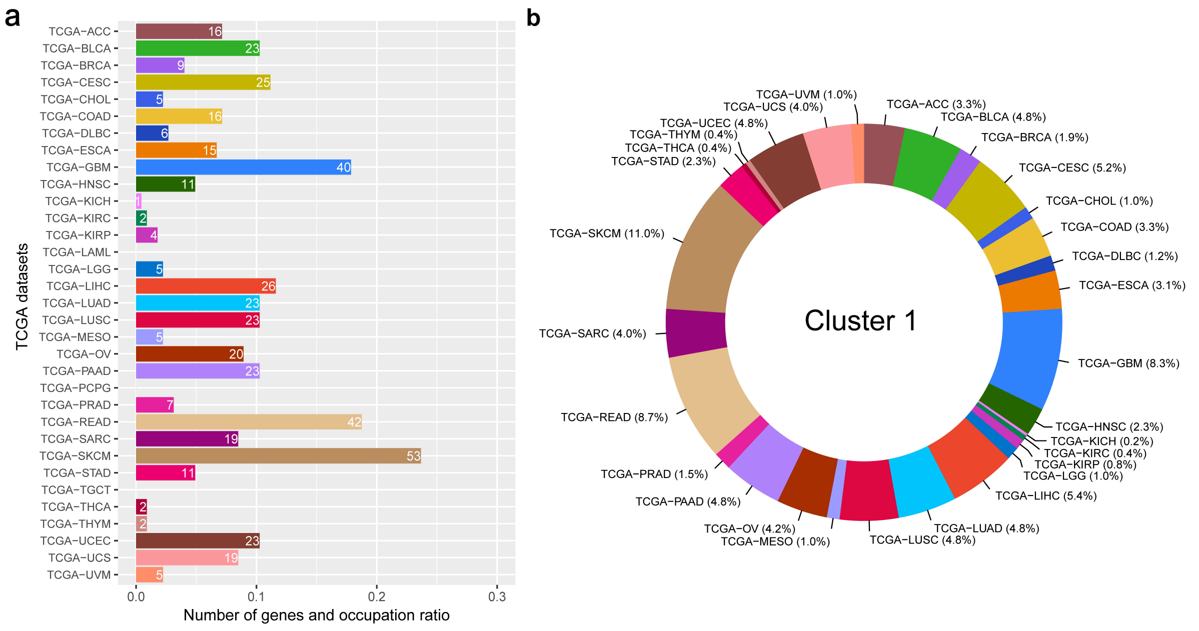

Supplementary Figure S11.1. Cluster occupation of TCGA dataset.

(a) The number of PSGs in the dataset is shown in coloured bar for each TCGA dataset. The length of bar indicates cluster occupation ratio of the dataset, which takes the number of shared genes into account. (b) The percentage value is only based on the number of PSGs in the cluster. The name of subnetwork is on the centre of circle.

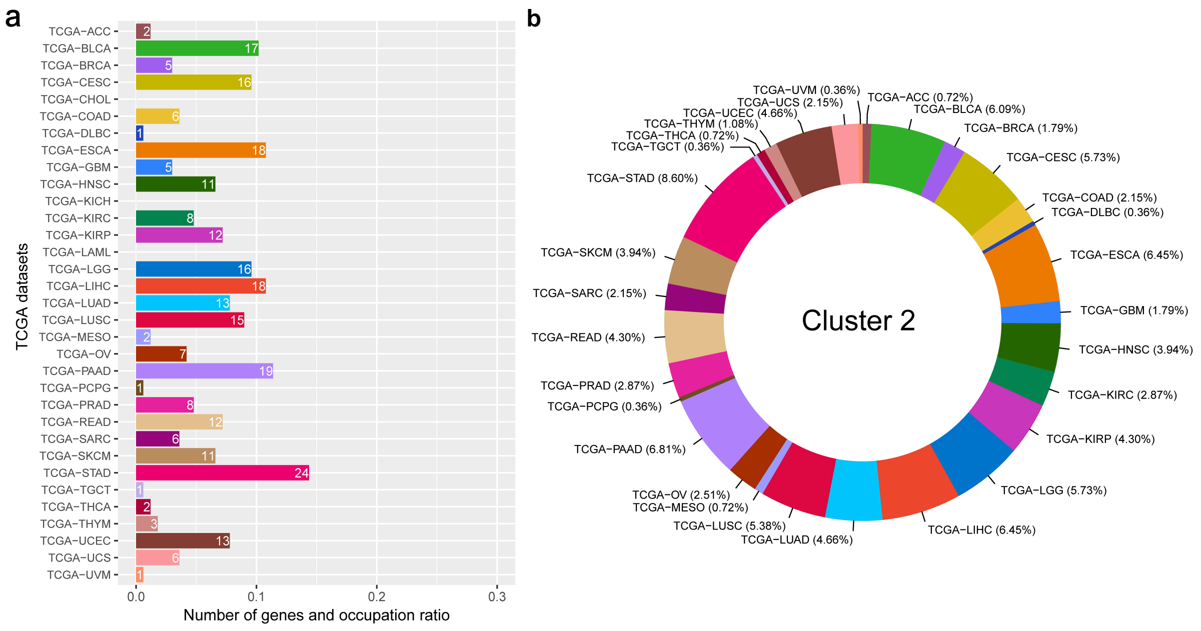

Supplementary Figure S11.2. Cluster occupation of TCGA dataset.

(a) The number of PSGs in the dataset is shown in coloured bar for each TCGA dataset. The length of bar indicates cluster occupation ratio of the dataset, which takes the number of shared genes into account. (b) The percentage value is only based on the number of PSGs in the cluster. The name of subnetwork is on the centre of circle.

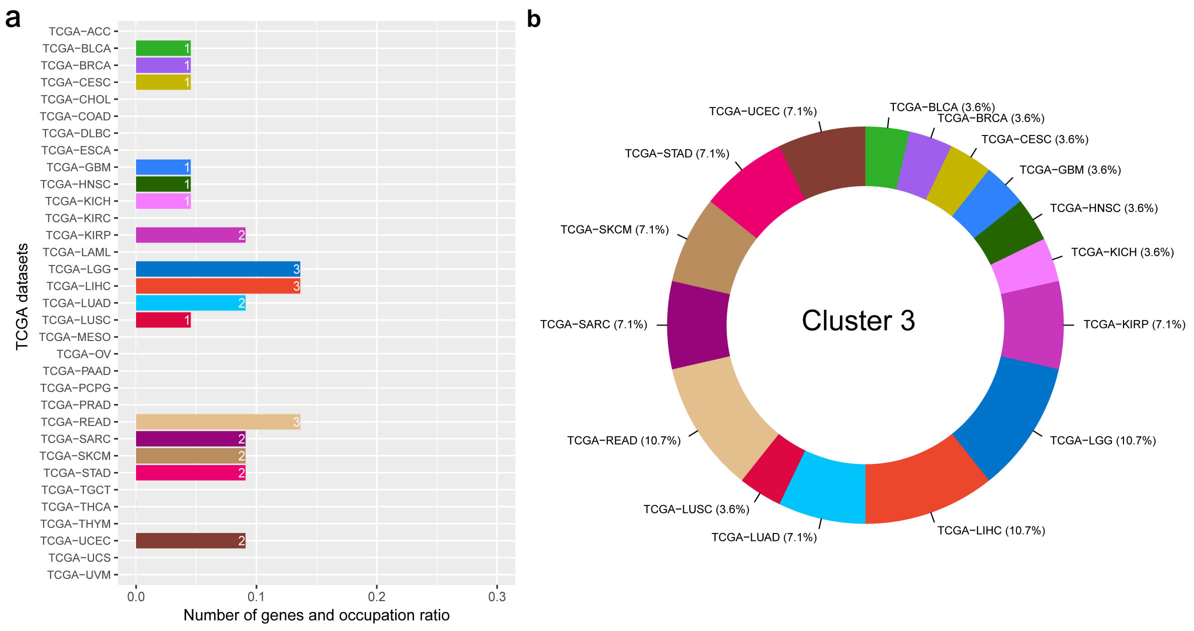

Supplementary Figure S11.3. Cluster occupation of TCGA dataset.

(a) The number of PSGs in the dataset is shown in coloured bar for each TCGA dataset. The length of bar indicates cluster occupation ratio of the dataset, which takes the number of shared genes into account. (b) The percentage value is only based on the number of PSGs in the cluster. The name of subnetwork is on the centre of circle.

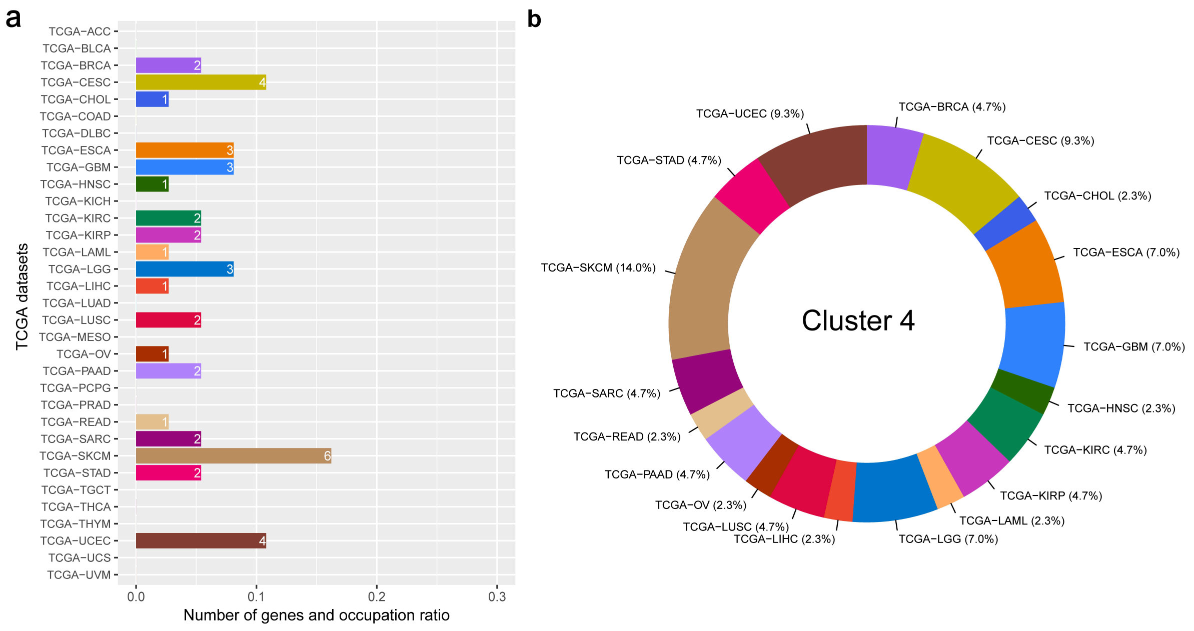

Supplementary Figure S11.4. Cluster occupation of TCGA dataset.

(a) The number of PSGs in the dataset is shown in coloured bar for each TCGA dataset. The length of bar indicates cluster occupation ratio of the dataset, which takes the number of shared genes into account. (b) The percentage value is only based on the number of PSGs in the cluster. The name of subnetwork is on the centre of circle.

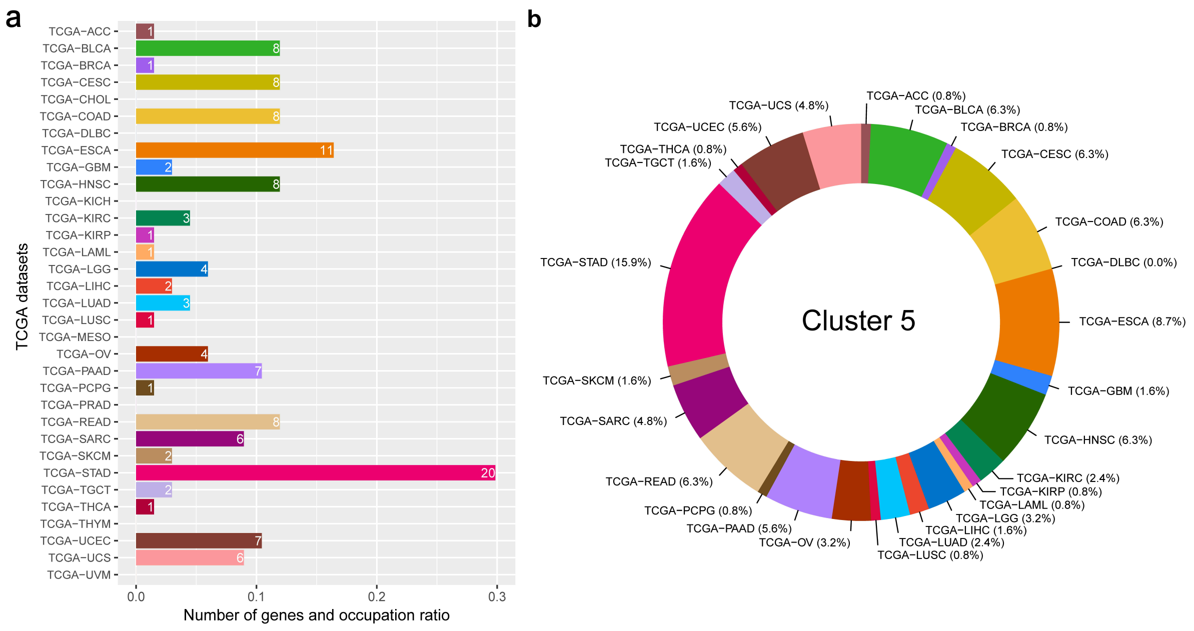

Supplementary Figure S11.5. Cluster occupation of TCGA dataset.

(a) The number of PSGs in the dataset is shown in coloured bar for each TCGA dataset. The length of bar indicates cluster occupation ratio of the dataset, which takes the number of shared genes into account. (b) The percentage value is only based on the number of PSGs in the cluster. The name of subnetwork is on the centre of circle.

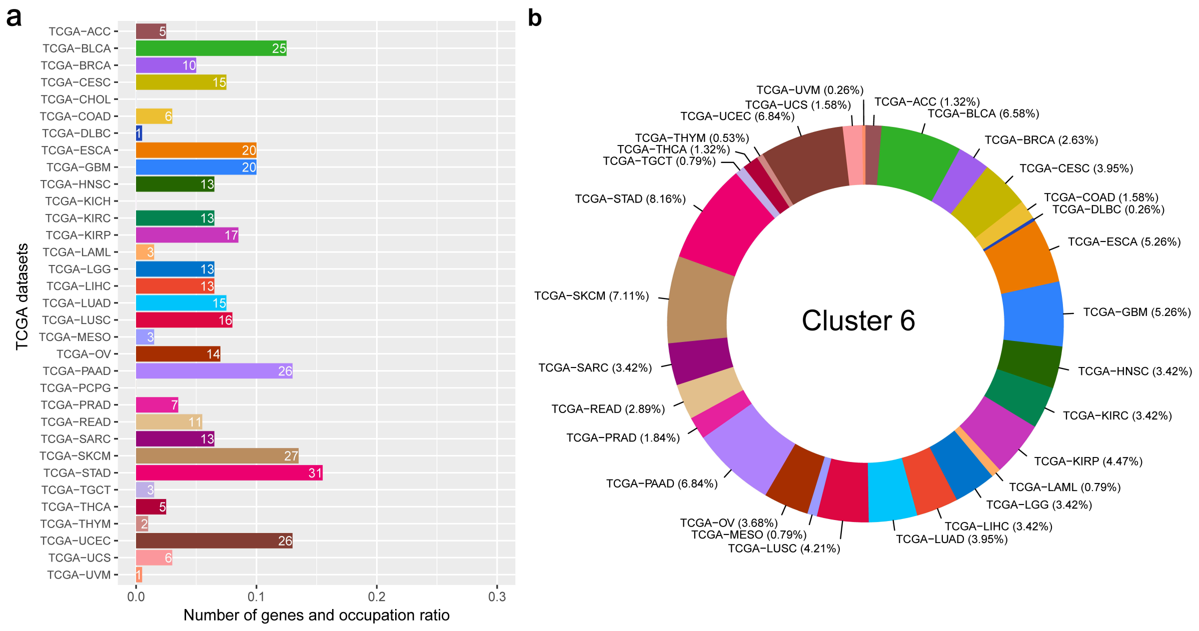

Supplementary Figure S11.6. Cluster occupation of TCGA dataset.

(a) The number of PSGs in the dataset is shown in coloured bar for each TCGA dataset. The length of bar indicates cluster occupation ratio of the dataset, which takes the number of shared genes into account. (b) The percentage value is only based on the number of PSGs in the cluster. The name of subnetwork is on the centre of circle.

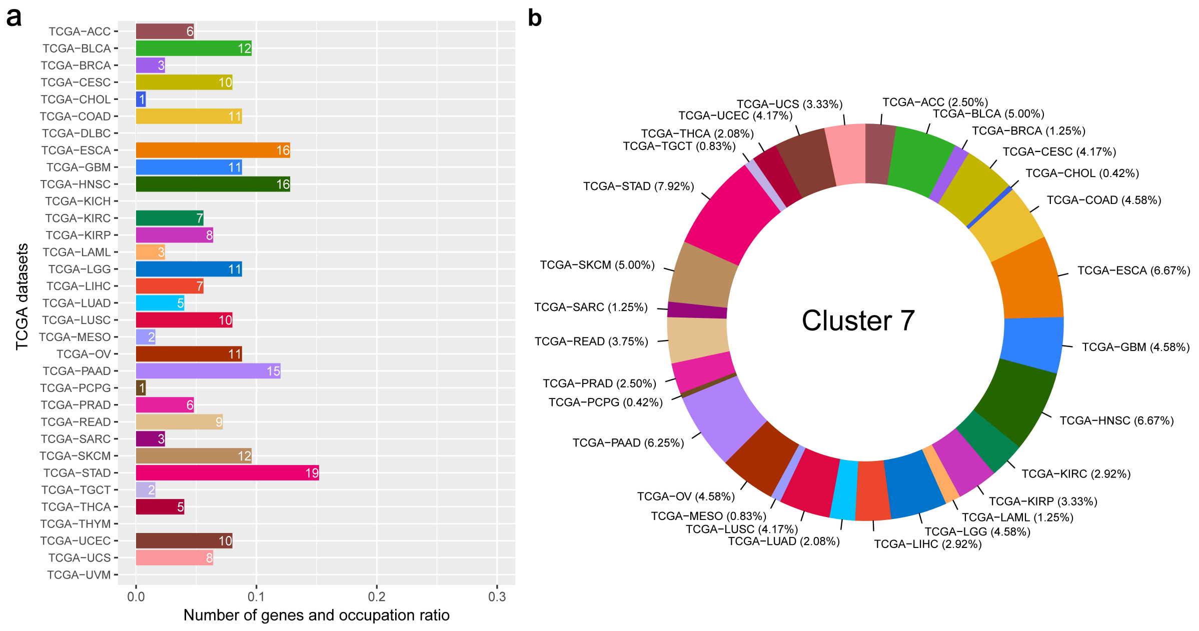

Supplementary Figure S11.7. Cluster occupation of TCGA dataset.

(a) The number of PSGs in the dataset is shown in coloured bar for each TCGA dataset. The length of bar indicates cluster occupation ratio of the dataset, which takes the number of shared genes into account. (b) The percentage value is only based on the number of PSGs in the cluster. The name of subnetwork is on the centre of circle.

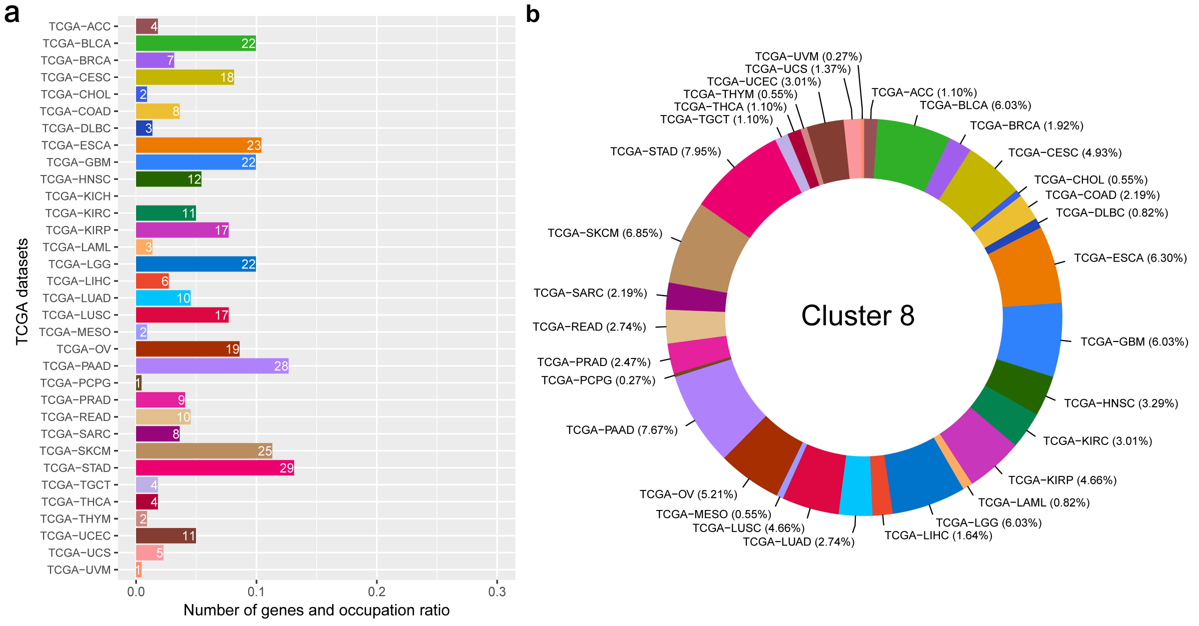

Supplementary Figure S11.8. Cluster occupation of TCGA dataset.

(a) The number of PSGs in the dataset is shown in coloured bar for each TCGA dataset. The length of bar indicates cluster occupation ratio of the dataset, which takes the number of shared genes into account. (b) The percentage value is only based on the number of PSGs in the cluster. The name of subnetwork is on the centre of circle.

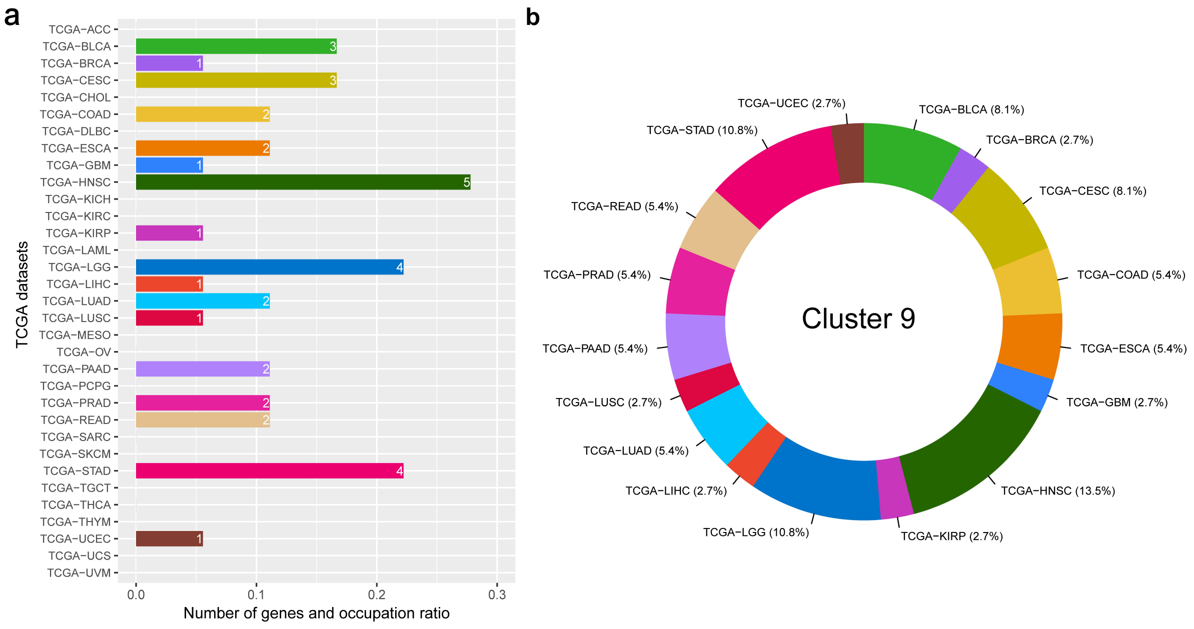

Supplementary Figure S11.9. Cluster occupation of TCGA dataset.

(a) The number of PSGs in the dataset is shown in coloured bar for each TCGA dataset. The length of bar indicates cluster occupation ratio of the dataset, which takes the number of shared genes into account. (b) The percentage value is only based on the number of PSGs in the cluster. The name of subnetwork is on the centre of circle.

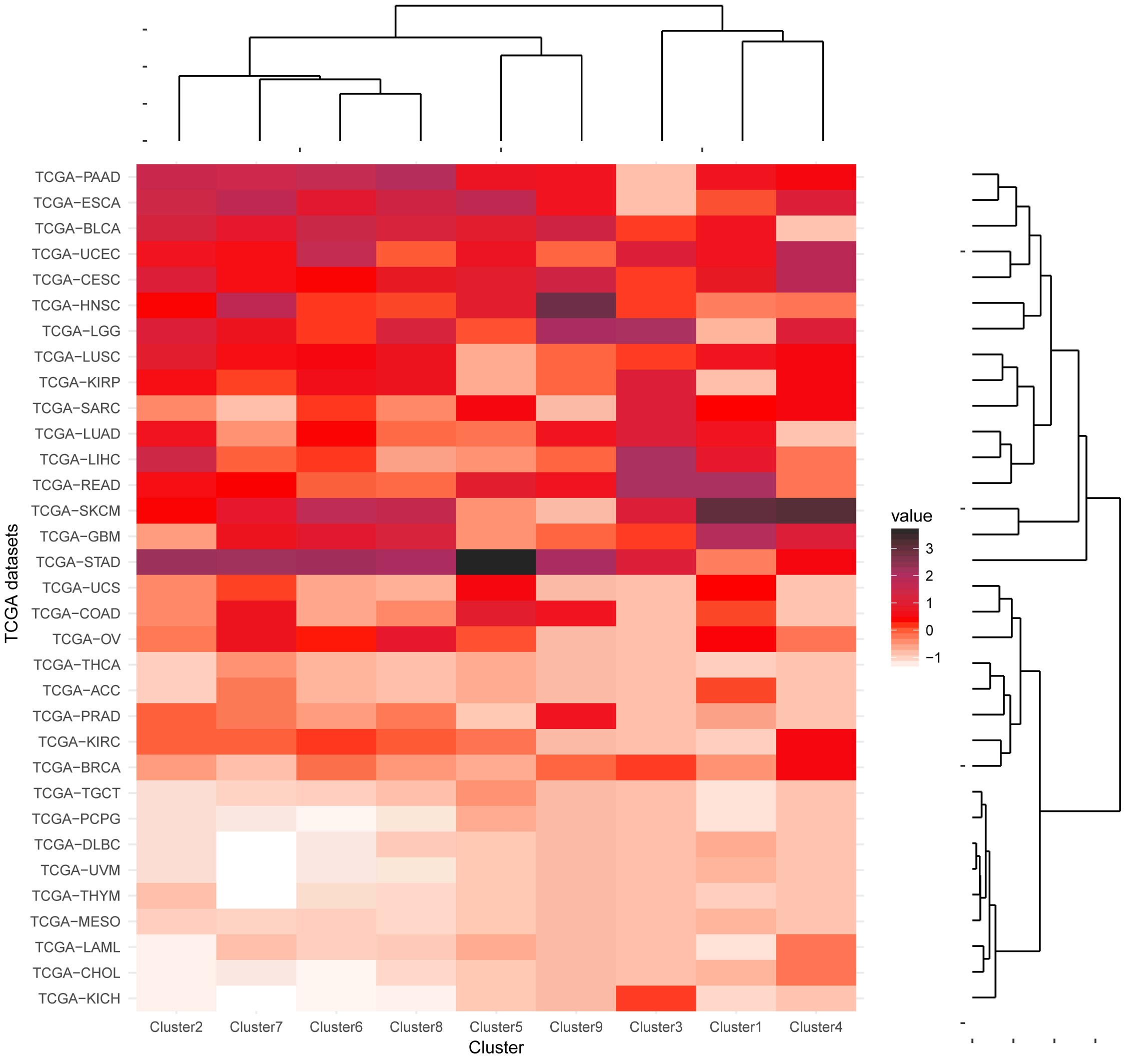

Supplementary Figure S12. Heatmap of cluster-cancer type relationships based on the number of PSGs in clusters.

Heatmap of normalised numbers of PSGs of TCGA dataset in clusters. Clusters are shown in x-axis and TCGA datasets are in y-axis. A dendrogram on top of the heatmap show clustering of the subnetworks. A dendrogram on right of the heatmap show clustering of the TCGA datasets.

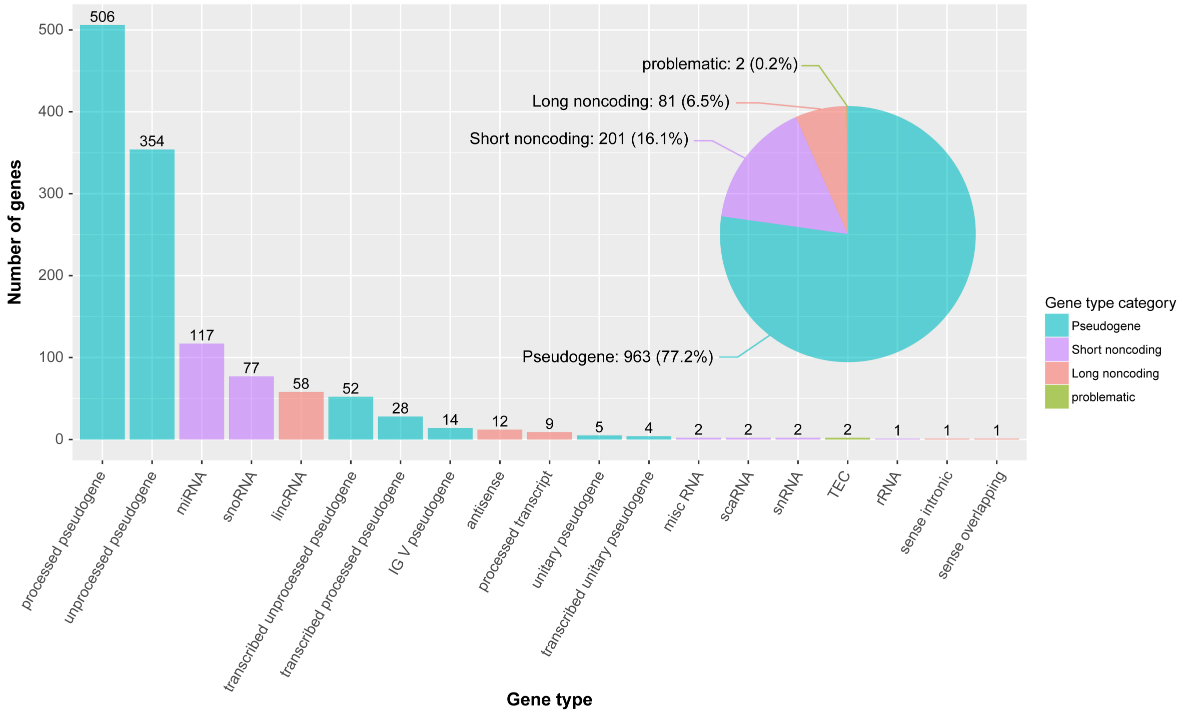

Supplementary Figure S13. Classification of non-coding PSGs (ncPSGs).

The number of ncPSGs (y-axis) for each gene type (x-axis) is shown. Each gene type is coloured according to classification categories, which included pseudogene (light blue), short noncoding (light purple), long noncoding (light red), and problematic (light green). NcPSGs occupation ratio of gene type categories is shown in a piechart. The colour indicator is on the right side of figure.

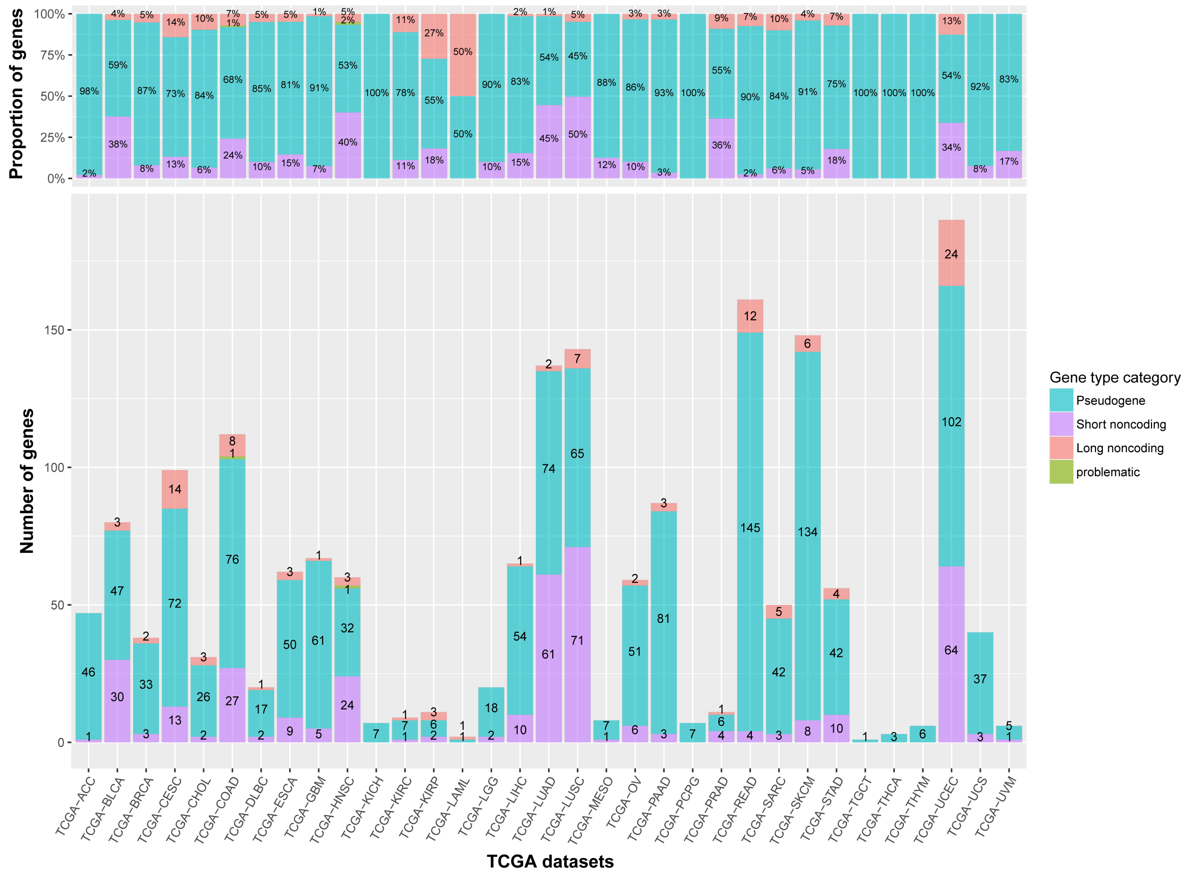

Supplementary Figure S14. Distribution of non-coding gene type of ncPSGs in each TCGA dataset.

In each TCGA dataset, ncPSGs occupation ratio of gene type categories and the number of gene that belong to gene type are shown in coloured bar. The colour indicator for gene type category is on right side.

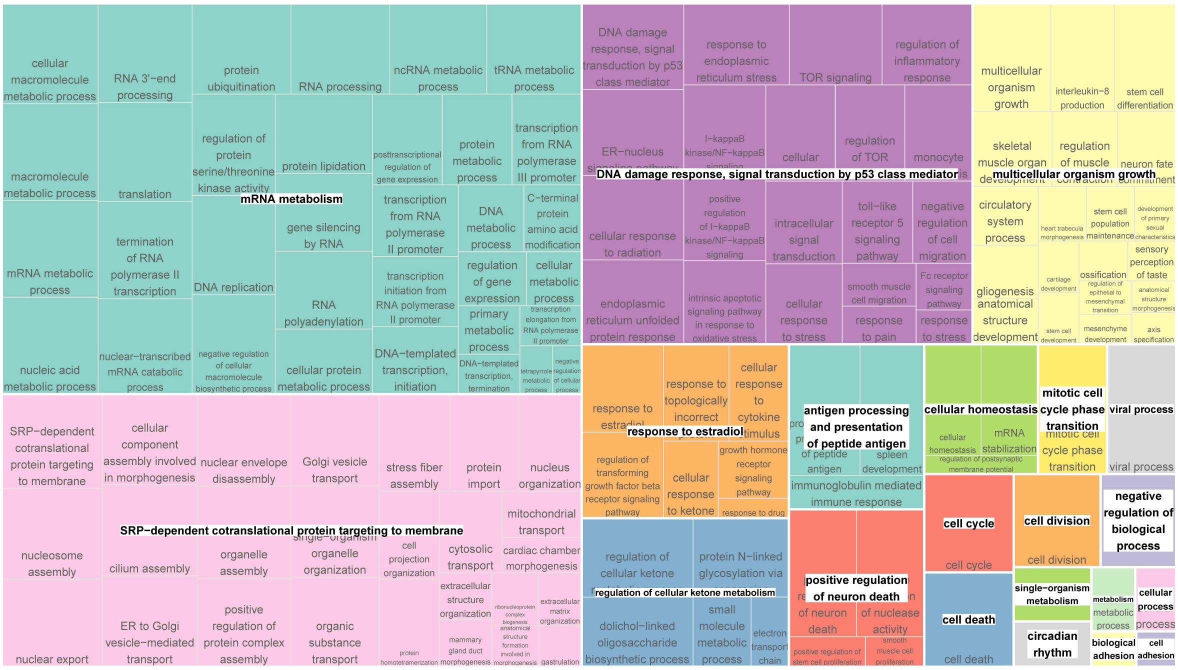

Supplementary Figure S15. GO term summarisation of lncRNA in the ncPSGs group.

GO term summary of lncRNA in the ncPSGs group are presented as a tree map. The frequency of GO terms from the results of LncRNA Ontology was used to determine the size of block. Similar GO terms were combined and grouped into a large block of the same color. The most frequent term in the large block is shown on the white box and located at the centre of the block.

Supplementary Table S1. Primary site and disease type of TCGA datasets.

TCGA dataset	Primary site	Disease type
TCGA-ACC	Adrenal Gland	Adrenocortical Carcinoma
TCGA-BLCA	Bladder	Bladder Urothelial Carcinoma
TCGA-BRCA	Breast	Breast Invasive Carcinoma
TCGA-CESC	Cervix	Cervical Squamous Cell Carcinoma and Endocervical Adenocarcinoma
TCGA-CHOL	Bile Duct	Cholangiocarcinoma
TCGA-COAD	Colorectal	Colon Adenocarcinoma
TCGA-DLBC	Lymph Nodes	Lymphoid Neoplasm Diffuse Large B-cell Lymphoma
TCGA-ESCA	Esophagus	Esophageal Carcinoma
TCGA-GBM	Brain	Glioblastoma Multiforme
TCGA-HNSC	Head and Neck	Head and Neck Squamous Cell Carcinoma
TCGA-KICH	Kidney	Kidney Chromophobe
TCGA-KIRC	Kidney	Kidney Renal Clear Cell Carcinoma
TCGA-KIRP	Kidney	Kidney Renal Papillary Cell Carcinoma
TCGA-LAML	Bone Marrow	Acute Myeloid Leukemia
TCGA-LGG	Brain	Brain Lower Grade Glioma
TCGA-LIHC	Liver	Liver Hepatocellular Carcinoma
TCGA-LUAD	Lung	Lung Adenocarcinoma
TCGA-LUSC	Lung	Lung Squamous Cell Carcinoma
TCGA-MESO	Pleura	Mesothelioma
TCGA-OV	Ovary	Ovarian Serous Cystadenocarcinoma
TCGA-PAAD	Pancreas	Pancreatic Adenocarcinoma
TCGA-PCPG	Adrenal Gland	Pheochromocytoma and Paraganglioma
TCGA-PRAD	Prostate	Prostate Adenocarcinoma
TCGA-READ	Colorectal	Rectum Adenocarcinoma
TCGA-SARC	Soft Tissue	Sarcoma
TCGA-SKCM	Skin	Skin Cutaneous Melanoma
TCGA-STAD	Stomach	Stomach Adenocarcinoma
TCGA-TGCT	Testis	Testicular Germ Cell Tumors
TCGA-THCA	Thyroid	Thyroid Carcinoma
TCGA-THYM	Thymus	Thymoma
TCGA-UCEC	Uterus	Uterine Corpus Endometrial Carcinoma
TCGA-UCS	Uterus	Uterine Carcinosarcoma
TCGA-UVM	Eye	Uveal Melanoma

Supplementary Table S2. Somatic mutation data of TCGA datasets.

TCGA dataset	File name	Data type	Data format	Size	Cases	Annotations
TCGA-ACC	TCGA.ACC.muse.e5858fc5-8298-4fdf-ab81-1e706ed7eef6.somatic.maf.gz	Masked Somatic Mutation	MAF	1.40 MB	92	13
TCGA-BLCA	TCGA.BLCA.muse.1367593a-7e5f-4f0f-a1dc-765edf7174ca.somatic.maf.gz	Masked Somatic Mutation	MAF	19.05 MB	412	52
TCGA-BRCA	TCGA.BRCA.muse.8751a889-cb3e-4487-ba6f-ac91651666e7.somatic.maf.gz	Masked Somatic Mutation	MAF	14.15 MB	1,044	47
TCGA-CESC	TCGA.CESC.muse.bd35e965-971d-4bc6-acf2-99205450be78.somatic.maf.gz	Masked Somatic Mutation	MAF	11.47 MB	305	0
TCGA-CHOL	TCGA.CHOL.muse.e84c7316-e619-4b01-a23e-218ed99cc428.somatic.maf.gz	Masked Somatic Mutation	MAF	530 KB	51	16
TCGA-COAD	TCGA.COAD.muse.7d9db44b-21ee-46e2-8a43-ecf00c295b28.somatic.maf.gz	Masked Somatic Mutation	MAF	26.20 MB	432	0
TCGA-DLBC	TCGA.DLBC.muse.987edefd-0e63-4617-a793-d3c1241de62a.somatic.maf.gz	Masked Somatic Mutation	MAF	1.21 MB	48	1
TCGA-ESCA	TCGA.ESCA.muse.49fc4a97-d38a-463c-95ea-384282d5a8fa.somatic.maf.gz	Masked Somatic Mutation	MAF	4.44 MB	184	0
TCGA-GBM	TCGA.GBM.muse.730ddd72-d57c-4bce-9d3c-c0dd57b03754.somatic.maf.gz	Masked Somatic Mutation	MAF	6.50 MB	396	91
TCGA-HNSC	TCGA.HNSC.muse.2d2f9250-7ba6-48ef-8111-b373c5fffd6f.somatic.maf.gz	Masked Somatic Mutation	MAF	13.75 MB	510	0
TCGA-KICH	TCGA.KICH.muse.a05a7fd4-d309-4b61-9724-a021d6c45ccb.somatic.maf.gz	Masked Somatic Mutation	MAF	267 KB	66	8
TCGA-KIRC	TCGA.KIRC.muse.f7aa19c6-369a-4fca-a0ce-a9142c947020.somatic.maf.gz	Masked Somatic Mutation	MAF	3.39 MB	339	86
TCGA-KIRP	TCGA.KIRP.muse.18188602-bc86-46ff-aff7-baff03114282.somatic.maf.gz	Masked Somatic Mutation	MAF	3.31 MB	288	47
TCGA-LAML	TCGA.LAML.muse.64407a2f-c723-4d31-a21b-d53cc1c570e4.somatic.maf.gz	Masked Somatic Mutation	MAF	365 KB	149	149
TCGA-LGG	TCGA.LGG.muse.1cb98f27-bc2d-4738-8b41-64f3b1055fb2.somatic.maf.gz	Masked Somatic Mutation	MAF	4.23 MB	513	54
TCGA-LIHC	TCGA.LIHC.muse.fae05f3d-ed6a-4f3b-9af0-c675a1a33943.somatic.maf.gz	Masked Somatic Mutation	MAF	7.47 MB	375	20
TCGA-LUAD	TCGA.LUAD.muse.f8f598d1-637a-4be2-82fa-1e4712c701b1.somatic.maf.gz	Masked Somatic Mutation	MAF	29.54 MB	569	84
TCGA-LUSC	TCGA.LUSC.muse.c6828a00-67e5-47e3-9386-a99add1dc181.somatic.maf.gz	Masked Somatic Mutation	MAF	26.45 MB	497	108
TCGA-MESO	TCGA.MESO.muse.e16eb19b-e610-4746-8479-613cf9ed8cf5.somatic.maf.gz	Masked Somatic Mutation	MAF	469 KB	83	4
TCGA-OV	TCGA.OV.muse.10dbdb2f-0444-434e-9520-06bc24d7db20.somatic.maf.gz	Masked Somatic Mutation	MAF	5.76 MB	440	44
TCGA-PAAD	TCGA.PAAD.muse.a8c39fc8-57f1-4409-b8fd-9296a9f7a2c2.somatic.maf.gz	Masked Somatic Mutation	MAF	3.47 MB	183	0
TCGA-PCPG	TCGA.PCPG.muse.7c6b6a28-1e70-4347-91e0-3e7424e2ca02.somatic.maf.gz	Masked Somatic Mutation	MAF	289 KB	179	55
TCGA-PRAD	TCGA.PRAD.muse.a87f6f43-ec67-4f4d-a5e1-997b58eede9a.somatic.maf.gz	Masked Somatic Mutation	MAF	3.05 MB	498	33
TCGA-READ	TCGA.READ.muse.7299e40f-605a-4caf-add2-d7acbd867bc5.somatic.maf.gz	Masked Somatic Mutation	MAF	7.03 MB	158	0
TCGA-SARC	TCGA.SARC.muse.0b124afd-e591-4c63-b2a2-2e16448fd50d.somatic.maf.gz	Masked Somatic Mutation	MAF	2.89 MB	255	26

TCGA-SKCM	TCGA.SKCM.muse.49540f53-3228-4a65-8854-ebd2f4a8f5ce.somatic.maf.gz	Masked Somatic Mutation	MAF	52.39 MB	470	48
TCGA-STAD	TCGA.STAD.muse.73ce7ac0-ad88-4471-8cf1-4cb5d3d0efee.somatic.maf.gz	Masked Somatic Mutation	MAF	21.18 MB	441	0
TCGA-TGCT	TCGA.TGCT.muse.1d8baa25-7aef-4c68-8c2b-de00f4c99e8f.somatic.maf.gz	Masked Somatic Mutation	MAF	416 KB	150	1
TCGA-THCA	TCGA.THCA.muse.8c58393b-584b-44c8-8cc8-c610670a03f3.somatic.maf.gz	Masked Somatic Mutation	MAF	879 KB	496	23
TCGA-THYM	TCGA.THYM.muse.0c7ebf88-9139-44cb-8d5a-00487ecc9cb4.somatic.maf.gz	Masked Somatic Mutation	MAF	284 KB	123	3
TCGA-UCEC	TCGA.UCEC.muse.3317f8f1-02e4-4351-8966-0269adc948e6.somatic.maf.gz	Masked Somatic Mutation	MAF	88.55 MB	542	0
TCGA-UCS	TCGA.UCS.muse.55f34d65-a97c-47e6-a449-24005503ed1c.somatic.maf.gz	Masked Somatic Mutation	MAF	1.29 MB	57	0
TCGA-UVM	TCGA.UVM.muse.6304d4a9-eba8-48b9-bf84-06f505a4cc08.somatic.maf.gz	Masked Somatic Mutation	MAF	195 KB	80	0

Supplementary Table S3. Number of cases in TCGA datasets for gene expression quantification data.

TCGA dataset	Cases
TCGA-ACC	79
TCGA-BLCA	408
TCGA-BRCA	1091
TCGA-CESC	304
TCGA-CHOL	36
TCGA-COAD	456
TCGA-DLBC	48
TCGA-ESCA	161
TCGA-GBM	154
TCGA-HNSC	500
TCGA-KICH	65
TCGA-KIRC	530
TCGA-KIRP	288
TCGA-LAML	151
TCGA-LGG	511
TCGA-LIHC	371
TCGA-LUAD	513
TCGA-LUSC	501
TCGA-MESO	86
TCGA-OV	374
TCGA-PAAD	177
TCGA-PCPG	179
TCGA-PRAD	495
TCGA-READ	166
TCGA-SARC	259
TCGA-SKCM	103
TCGA-STAD	375
TCGA-TGCT	150
TCGA-THCA	502
TCGA-THYM	119
TCGA-UCEC	543
TCGA-UCS	56
TCGA-UVM	80

Supplementary Table S4. Filtering of somatic mutation data.

TCGA dataset	Raw variant count	Variant count after filtering
TCGA-ACC	7,603	7,318
TCGA-BLCA	99,908	88,301
TCGA-BRCA	73,751	68,571
TCGA-CESC	62,234	60,739
TCGA-CHOL	2,742	2,595
TCGA-COAD	142,789	141,295
TCGA-DLBC	6,471	6,243
TCGA-ESCA	24,261	23,582
TCGA-GBM	37,171	34,606
TCGA-HNSC	73,300	71,704
TCGA-KICH	1,299	1,192
TCGA-KIRC	16,586	13,856
TCGA-KIRP	15,962	14,029
TCGA-LAML	1,750	1,704
TCGA-LGG	23,073	19,350
TCGA-LIHC	38,556	36,718
TCGA-LUAD	167,236	143,132
TCGA-LUSC	147,949	124,656
TCGA-MESO	2,262	2,135
TCGA-OV	29,699	26,776
TCGA-PAAD	20,617	20,526
TCGA-PCPG	1,331	1,046
TCGA-PRAD	15,797	14,875
TCGA-READ	41,862	41,212
TCGA-SARC	15,710	13,823
TCGA-SKCM	320,510	50,330
TCGA-STAD	113,098	110,976
TCGA-TGCT	1,894	1,823
TCGA-THCA	4,226	3,872
TCGA-THYM	1,388	1,335
TCGA-UCEC	539,986	530,149
TCGA-UCS	7,023	6,875
TCGA-UVM	946	885

Supplementary Table S5. Number of variants and associated genes after filtering.

TCGA dataset	Number of aliquots	Number of variants per aliquot	Number of genes with variants
TCGA-ACC	91	80.4	5,186
TCGA-BLCA	364	242.6	19,385
TCGA-BRCA	999	68.6	18,716
TCGA-CESC	304	199.8	18,540
TCGA-CHOL	48	54.1	2,267
TCGA-COAD	432	327.1	21,654
TCGA-DLBC	47	132.8	4,384
TCGA-ESCA	183	128.9	11,606
TCGA-GBM	358	96.7	14,496
TCGA-HNSC	507	141.4	18,212
TCGA-KICH	60	19.9	1,121
TCGA-KIRC	282	49.1	8,681
TCGA-KIRP	253	55.5	8,712
TCGA-LAML	116	14.7	1,545
TCGA-LGG	460	42.1	10,625
TCGA-LIHC	357	102.9	14,537
TCGA-LUAD	490	292.1	20,680
TCGA-LUSC	430	289.9	20,399
TCGA-MESO	76	28.1	1,871
TCGA-OV	409	65.5	12,805
TCGA-PAAD	170	120.7	11,246
TCGA-PCPG	142	7.4	954
TCGA-PRAD	457	32.5	8,987
TCGA-READ	157	262.5	15,097
TCGA-SARC	230	60.1	8,407
TCGA-SKCM	102	493.4	15,058
TCGA-STAD	435	255.1	20,273
TCGA-TGCT	153	11.9	1,612
TCGA-THCA	465	8.3	3,031
TCGA-THYM	105	12.7	1,164
TCGA-UCEC	541	979.9	26,042
TCGA-UCS	57	120.6	5,151
TCGA-UVM	80	11.1	780

Supplementary Table S6. Filtering cases of gene expression quantification data.

TCGA dataset	Number of cases	Number of cases after filtering
TCGA-ACC	79	78
TCGA-BLCA	408	356
TCGA-BRCA	1,091	1,027
TCGA-CESC	304	304
TCGA-CHOL	36	35
TCGA-COAD	456	456
TCGA-DLBC	48	47
TCGA-ESCA	161	161
TCGA-GBM	154	138
TCGA-HNSC	500	500
TCGA-KICH	65	59
TCGA-KIRC	530	426
TCGA-KIRP	288	248
TCGA-LAML	151	145
TCGA-LGG	511	461
TCGA-LIHC	371	347
TCGA-LUAD	513	438
TCGA-LUSC	501	435
TCGA-MESO	86	80
TCGA-OV	374	249
TCGA-PAAD	177	177
TCGA-PCPG	179	139
TCGA-PRAD	495	456
TCGA-READ	166	166
TCGA-SARC	259	225
TCGA-SKCM	103	101
TCGA-STAD	375	375
TCGA-TGCT	150	149
TCGA-THCA	502	475
TCGA-THYM	119	116
TCGA-UCEC	543	543
TCGA-UCS	56	56
TCGA-UVM	80	80

Supplementary Table S7. Soft-thresholding powers.

TCGA dataset	Soft-thresholding power
TCGA-KICH	5
TCGA-STAD	3
TCGA-SARC	3
TCGA-UCS	5
TCGA-BLCA	10
TCGA-SKCM	5
TCGA-LUSC	2
TCGA-THYM	6
TCGA-TGCT	4
TCGA-PRAD	2
TCGA-COAD	2
TCGA-CHOL	3
TCGA-HNSC	2
TCGA-READ	3
TCGA-GBM	4
TCGA-LAML	5
TCGA-ACC	5
TCGA-PCPG	4
TCGA-CESC	4
TCGA-LUAD	3
TCGA-THCA	8
TCGA-KIRC	2
TCGA-OV	5
TCGA-UCEC	2
TCGA-PAAD	6
TCGA-UVM	5
TCGA-KIRP	2
TCGA-DLBC	2
TCGA-LGG	3
TCGA-MESO	5
TCGA-ESCA	3
TCGA-BRCA	3
TCGA-LIHC	3

Supplementary Table S8. Selection of WGCNA module hub genes.

TCGA dataset	Total number of genes in WGCNA analysis	Number of modules	Total number of genes in all module (not grey)	Total number of module hub genes
TCGA-ACC	50,761	78	49,462	3,765
TCGA-BLCA	57,288	170	28,550	3,400
TCGA-BRCA	57,288	175	35,296	2,577
TCGA-CESC	54,452	215	44,426	1,891
TCGA-CHOL	48,243	37	46,836	4,015
TCGA-COAD	57,288	72	36,880	3,400
TCGA-DLBC	57,288	51	53,086	4,938
TCGA-ESCA	57,288	138	50,991	3,099
TCGA-GBM	53,754	150	49,973	2,439
TCGA-HNSC	57,288	117	37,310	2,614
TCGA-KICH	50,163	57	47,586	3,870
TCGA-KIRC	57,288	69	41,080	2,543
TCGA-KIRP	57,288	79	41,048	3,652
TCGA-LAML	55,256	146	50,504	1,463
TCGA-LGG	55,261	143	39,973	1,110
TCGA-LIHC	54,099	243	45,888	1,924
TCGA-LUAD	57,288	161	36,525	3,276
TCGA-LUSC	57,288	117	32,665	3,415
TCGA-MESO	51,301	72	48,788	3,431
TCGA-OV	55,148	209	45,620	1,720
TCGA-PAAD	53,218	138	48,528	2,772
TCGA-PCPG	52,781	139	49,846	2,717
TCGA-PRAD	57,288	54	29,596	2,934
TCGA-READ	52,304	170	47,023	2,994
TCGA-SARC	54,678	211	50,727	2,180
TCGA-SKCM	52,935	103	50,424	3,210
TCGA-STAD	57,288	122	41,575	2,390
TCGA-TGCT	53,625	135	50,734	2,198
TCGA-THCA	55,049	128	30,556	885
TCGA-THYM	52,458	94	50,936	3,276
TCGA-UCEC	57,288	117	38,990	2,938
TCGA-UCS	51,897	59	50,548	3,232
TCGA-UVM	47,536	83	46,445	3,861

Supplementary Table S9. Number of genes at the intersection between genes associated with somatic mutations and WGCNA module hub genes.

TCGA dataset	Number of genes of intersection
TCGA-ACC	157
TCGA-BLCA	339
TCGA-BRCA	202
TCGA-CESC	374
TCGA-CHOL	58
TCGA-COAD	260
TCGA-DLBC	84
TCGA-ESCA	355
TCGA-GBM	316
TCGA-HNSC	254
TCGA-KICH	23
TCGA-KIRC	136
TCGA-KIRP	154
TCGA-LAML	34
TCGA-LGG	286
TCGA-LIHC	242
TCGA-LUAD	404
TCGA-LUSC	409
TCGA-MESO	42
TCGA-OV	289
TCGA-PAAD	350
TCGA-PCPG	19
TCGA-PRAD	112
TCGA-READ	399
TCGA-SARC	207
TCGA-SKCM	460
TCGA-STAD	482
TCGA-TGCT	37
TCGA-THCA	32
TCGA-THYM	29
TCGA-UCEC	536
TCGA-UCS	110
TCGA-UVM	23

Supplementary Table S10. Number of genes in subnetworks and identification in DAVID Bioinformatics Resources 6.8.

Subnetwork	Number of genes	Number of mapped genes	Number of unmapped genes	Unmapped genes
Cluster1	342	341	1	RP11-294C11.1
Cluster2	536	535	1	RP11-577H5.5
Cluster3	69	69	0	
Cluster4	158	157	1	VIMP
Cluster5	151	151	0	
Cluster6	521	520	1	AC007192.4
Cluster7	399	399	0	
Cluster8	857	853	4	RP4-576H24.4<br>AC007192.4<br>RP11-330H6.5<br>RP11-548K23.11
Cluster9	38	38	0	

Supplementary Table S11. The number of known and candidate cancer genes and enrichment analysis.

		No. of genes	No. of known cancer genes	No. of candidate cancer genes
**Pan-cancer-wide selected genes**	**Network**	1,740	51 *	172 *
	**Not in network**	2,806	16	106
**Not pan-cancer-wide selected genes**	**Interactor in network**	5,076	281 *	318 *
	**Not interactor**	47,666	160	438
**Network**	**Representor**	1,740	51	172 *
	**Interactor in network**	5,076	281 **	318

* Known and candidate cancer genes were enriched in PSGs in network, interactors in network, and representors in network compared to PSGs not in network, non-interactor, or interactors in network.

** Known and candidate cancer genes were enriched in interactor group compared to representor group.

Supplementary Table S12. Glossary of non-coding gene type.

Gene type category	Gene type	Description	Description source
pseudogene	processed pseudogene	Pseudogene that appears to have been produced by integration of a reverse transcribed mRNA into the genome.	http://www.ensembl.org
pseudogene	unprocessed pseudogene	Pseudogenes that can contain introns since they are produced by gene duplication.	http://www.ensembl.org
Short noncoding	miRNA	It stands for micro RNA, a small RNA molecule (ca. 22 nucleotides) that functions in the post-transcriptional regulation of gene expression.	http://www.ensembl.org
Short noncoding	snoRNA	Small nucleolar RNA genes which can be involved in chemical modifications of other RNA genes.	http://www.ensembl.org
Long noncoding	lincRNA	Large intergenic non-coding RNAs, usually associated with open chromatin signatures such as histone modification sites.	http://www.ensembl.org
pseudogene	transcribed unprocessed pseudogene	Unprocessed pseudogenes that have evidence of transcription through the presence of locus-specific mRNAs and/or ESTs.	http://www.ensembl.org
pseudogene	transcribed processed pseudogene	Processed pseudogenes that have evidence of transcription through the presence of locus-specific mRNAs and/or ESTs.	http://www.ensembl.org
pseudogene	IG V pseudogene	Locus that shares an evolutionary history with the Ig V gene but it has been mutated through frameshift and/or stop codon(s) that disrupt the open reading frame.	http://www.ensembl.org
Long noncoding	antisense	Genes or transcripts that overlap any coding exon of a locus on the opposite strand. It also encompasses antisense regulation of a coding gene that have been reported in the literature.	http://www.ensembl.org
Long noncoding	processed transcript	Transcripts that don't contain an open reading frame (ORF) and cannot be placed in one of the other categories.	http://www.ensembl.org
pseudogene	unitary pseudogene	A species specific unprocessed pseudogene without a parent gene in the same species but with an active orthologue in another species.	http://www.ensembl.org
pseudogene	transcribed unitary pseudogene	Unitary pseudogene that have evidence of transcription through the presence of locus-specific mRNAs and/or ESTs.	-
Short noncoding	misc RNA	Short non coding RNA genes that have not been classified into the other short noncoding RNA biotypes such as scRNA, tRNA, rRNA, among others.	http://www.ensembl.org
Short noncoding	scaRNA	Small Cajal body-specific RNAs (scaRNAs) are a class of small nucleolar RNAs (snoRNAs) that specifically localise to the Cajal body, a nuclear organelle involved in the biogenesis of small nuclear ribonucleoproteins (snRNPs or snurps).	https://en.wikipedia.org
Short noncoding	snRNA	Small nuclear RNA.	http://www.ensembl.org
problematic	TEC	(To be Experimentally Confirmed). This is used for non-spliced EST clusters that have polyA features. This category has been specifically created for the ENCODE project to highlight regions that could indicate the presence of protein coding genes that require experimental validation, either by 5' RACE or RT-PCR to extend the transcripts, or by confirming expression of the putatively-encoded peptide with specific antibodies.	http://vega.archive.ensembl.org
Short noncoding	rRNA	The RNA component of the ribosome.	http://www.ensembl.org
Long noncoding	sense intronic	Long non-coding transcript that lies within the intron (does not overlap any exons) of a coding gene on the same strand.	http://www.ensembl.org
Long noncoding	sense overlapping	Long non-coding transcript that contains a coding gene within one of its introns and on the same strand.	http://www.ensembl.org

Supplementary Table S13. Enriched KEGG pathway term and associated ncPSGs.

KEGG pathway	Gene name
MicroRNAs in cancer	microRNA 1-1(MIR1-1)<br>microRNA 124-2(MIR124-2)<br>microRNA 124-3(MIR124-3)<br>microRNA 125a(MIR125A)<br>microRNA 129-1(MIR129-1)<br>microRNA 129-2(MIR129-2)<br>microRNA 133b(MIR133B)<br>microRNA 135a-2(MIR135A2)<br>microRNA 181a-1(MIR181A1)<br>microRNA 205(MIR205)<br>microRNA 206(MIR206)<br>microRNA 31(MIR31)<br>microRNA 494(MIR494)<br>microRNA 520a(MIR520A)<br>microRNA 520c(MIR520C)<br>microRNA 520g(MIR520G)<br>microRNA 7-3(MIR7-3)
